# Light restores sporulation in *Rhizopus microsporus* cured of its endosymbionts, unveiling their role in fitness and virulence

**DOI:** 10.1093/ismejo/wrag047

**Published:** 2026-04-08

**Authors:** Youssef Ahmiane, Carlos Lax, Sonia Béjar-González, Francisco Esteban Nicolás, Laura Camuña-Pardo, Maria José Figueras, Ana Fernández-Bravo, Victoriano Garre, Javier Capilla, Marta Sanchis

**Affiliations:** Unitat de Microbiologia, Facultat de Medicina i Ciències de la Salut, Universitat Rovira i Virgili, 43003 Reus, Tarragona, Spain; Departamento de Genética y Microbiología, Facultad de Biología, Universidad de Murcia, 30100 Murcia, Murcia, Spain; Unitat de Microbiologia, Facultat de Medicina i Ciències de la Salut, Universitat Rovira i Virgili, 43003 Reus, Tarragona, Spain; Departamento de Genética y Microbiología, Facultad de Biología, Universidad de Murcia, 30100 Murcia, Murcia, Spain; Unitat de Microbiologia, Facultat de Medicina i Ciències de la Salut, Universitat Rovira i Virgili, 43003 Reus, Tarragona, Spain; Unitat de Microbiologia, Facultat de Medicina i Ciències de la Salut, Universitat Rovira i Virgili, 43003 Reus, Tarragona, Spain; University Research Institute for Sustainability, Climate Change and Energy Transition (IU-RESCAT), 43480 Vila-seca, Tarragona, Spain; Unitat de Microbiologia, Facultat de Medicina i Ciències de la Salut, Universitat Rovira i Virgili, 43003 Reus, Tarragona, Spain; University Research Institute for Sustainability, Climate Change and Energy Transition (IU-RESCAT), 43480 Vila-seca, Tarragona, Spain; Departamento de Genética y Microbiología, Facultad de Biología, Universidad de Murcia, 30100 Murcia, Murcia, Spain; Unitat de Microbiologia, Facultat de Medicina i Ciències de la Salut, Universitat Rovira i Virgili, 43003 Reus, Tarragona, Spain; University Research Institute for Sustainability, Climate Change and Energy Transition (IU-RESCAT), 43480 Vila-seca, Tarragona, Spain; Unitat de Microbiologia, Facultat de Medicina i Ciències de la Salut, Universitat Rovira i Virgili, 43003 Reus, Tarragona, Spain; University Research Institute for Sustainability, Climate Change and Energy Transition (IU-RESCAT), 43480 Vila-seca, Tarragona, Spain; Serra Húnter Fellow, Unitat de Microbiologia i Microbiologia Ambiental, Universitat Rovira i Virgili, 43003 Reus, Tarragona, Spain

**Keywords:** light-induced sporulation, endosymbiosis, *Rhizopus microsporus*, endobacteria, fungal fitness, virulence

## Abstract

*Rhizopus microsporus* is a major cause of mucormycosis, an infection caused by Mucorales that increasingly affects immunocompromised individuals. Certain isolates of *R. microsporus* harbor bacterial endosymbionts that regulate key fungal functions, particularly asexual and sexual reproduction, but these effects have been explored exclusively in environmental isolates. Although some clinical isolates contain *Mycetohabitans* endosymbionts, their influence on fungal reproduction remains unknown. This dependence on endosymbionts for asexual spore formation in environmental isolates has established the *Rhizopus–Mycetohabitans* association as a model for studying fungal-bacterial endosymbiosis, but it has also constrained comparative studies across environmental and clinical backgrounds. We show that light exposure partially restores asexual sporulation in endosymbiont-cured environmental strains, enabling the generation of isogenic sporulating lines. Transcriptomic analyses revealed that both light and endobacteria modulate overlapping signal transduction pathways, regulating the expression of conserved genes involved in asexual development in Mucorales and other fungi*.* Functional assays demonstrated that asexual spores from cured strains are viable; however, the presence of endosymbionts accelerates spore formation, enhances osmotic stress tolerance, and helps maintain cell-wall integrity. Cured strains exhibit altered membrane composition, including reduced ergosterol levels, which may contribute to their resistance to macrophage phagocytosis. Despite these compensatory adaptations, cured strains showed attenuated virulence in a murine mucormycosis model, highlighting the role of bacterial endosymbionts in fungal pathogenicity. The discovery of light-induced sporulation in cured strains provides a valuable experimental framework for future comparative studies requiring asexual spores, offering new opportunities to explore the role of fungal-bacterial endosymbiosis in fungal biology and human disease.

## Introduction

Endosymbiosis is a powerful evolutionary strategy that enables microorganisms to adapt to diverse environments by sharing or extending metabolic functions, defense mechanisms, and developmental programs [1–3]. In fungi, intracellular bacterial symbionts are increasingly recognized as major drivers of ecological versatility, shaping host fitness, nutrient acquisition, reproduction, and virulence [4–7]. These associations, often vertically transmitted through spores, can persist over evolutionary timescales and lead to deep metabolic integration, with the symbiotic unit functioning as a single level of selection [4, 6, 8]. Despite their ecological and evolutionary importance, the mechanisms by which endosymbionts manipulate fungal physiology and how hosts accommodate or resist such control remain only partially understood, particularly in early-diverging fungal lineages such as the *Mucoromycota.* This phylum includes both the arbuscular mycorrhizal fungi members of *Glomeromycota* and the order Mucorales [5, 8, 9].

One of the best characterized examples of fungal-bacterial symbiosis occurs in *Rhizopus microsporus*, a member of the order Mucorales. In this species, some strains are unable to establish symbiosis with bacteria and are therefore considered non-host, whereas host strains harbor intracellular bacteria of the genus *Mycetohabitans* (formerly *Burkholderia*), which are transmitted vertically by fungal reproduction [10, 11]. This association causes rice seedling blight, mediated by rhizoxin, a bacterial toxin used by the fungus as a phytopathogenicity factor [12–14] and protection against micropredators [15]. In recent years, the *Rhizopus-Mycetohabitans* system has become a model for exploring molecular dynamics of fungal-bacterial endosymbiosis, revealing bacterial contributions to fungal morphogenesis, metabolism, and environmental interactions [8, 10, 11, 16]. Endosymbionts have been also detected in some clinical Mucorales isolates, yet there is no evidence currently associating them to mucormycosis pathogenesis [17–19]. *Rhizopus* spp., and other Mucorales, cause mucormycosis, a life-threatening fungal infection whose incidence has increased in immunocompromised populations and showed a marked rise during the recent SARS-CoV-2 pandemic [20–22]. Although the infection process is not yet fully understood, studies indicate that host tissue invasion begins with the recognition of specific host receptors by fungal CotH proteins, which mediate adhesion and invasion [23, 24]. This process is further enhanced by the release of the potent mycotoxin mucoricin, which plays a central role in tissue necrosis [25]. Moreover, interactions between fungal cells and host immune cells trigger major remodeling of gene expression networks governing survival and germination within phagosomes, nutrient acquisition, and oxidative stress responses [26]. Changes in cell wall structure or composition may also influence virulence; e.g. avirulent strains of *Mucor lusitanicus* display reduced tolerance to cell wall stressors such as calcofluor white and sodium dodecyl sulfate compared with virulent strains [27]. The contribution of endosymbiotic bacteria to enhanced fungal pathogenesis remains a controversial issue that requires further investigation to elucidate their biological role.

In *R. microsporus*, elimination of *Mycetohabitans* results in a failure to produce sporangiospores and a reduction of zygospore formation [10, 11, 28], restricting comparative studies that depend on spore-derived inocula [10, 29]. This dependence highlights the importance of elucidating host and bacterial regulatory mechanisms. Asexual reproduction, essential for fungal propagation and survival under diverse environmental conditions, is controlled by a complex interplay of genetic and environmental factors such as nutrient availability, pH, humidity, host interactions, and light [30, 31]. Although sporulation is well characterized in fungi such as *Aspergillus nidulans* [32, 33] and *Neurospora crassa* [33–35], the genetic mechanisms governing this process in Mucorales remain poorly defined [36–38]. In fact, only a few transcriptional regulators, most notably those linked to the white-collar complex (WCC), have been identified in this early-diverging fungi [39, 40]. Light acts as a key regulator of fungal development [40], not only in Mucorales but also in arbuscular mycorrhizal fungi where it interacts with root exudates to influence hyphal branching [41]. In addition, recent studies have identified bacterial-derived factors, such as TALE-like effectors and secondary metabolites, that modulate host sporulation [42–46], suggesting a dominant contribution of the bacterial partner to this developmental process [42–45, 47].

To further elucidate the role of endosymbionts in *R. microsporus* biology, we sought to determine how symbiosis controls asexual sporulation and the subsequent consequences for fungal fitness and virulence. To address this question, we developed a reliable method to induce sporulation in environmental strains cured of their endosymbionts, thereby enabling direct, controlled comparisons between symbiotic and aposymbiotic fungal states. The use of standardized spore suspensions rather than mycelial fragments allows for greater precision and reproducibility in experiments. Our findings reveal that bacterial endosymbionts control key genes in sporulation and profoundly influence the fitness and pathogenic potential of *R. microsporus*, providing a robust framework for dissecting the molecular basis of this facultative mutualistic relationship and its broader implications for fungal biology, ecology, and potential pathogenesis.

## Materials and methods

### Fungal strains and generation of cured *Rhizopus* strains

Environmental *R. microsporus* strains ATCC 52813 and ATCC 52814, both harboring the endobacteria *Mycetohabitans endofungorum* (formerly *Burkholderia endofungorum*; isolates HKI 0402/B4/B13 and HKI 0403/B7/B14*,* respectively), were used. The naturally endobacteria-free strain ATCC 11559 was included as a control. Endobacteria from both strains were eradicate after treating fresh sporangiospores with 20–40 μg/ml of ciprofloxacin (Acros Organics, China) [10], thereafter referred as cured strains or ATCC 52813 (−) and ATCC 52814 (−) in contrast to non-cured or ATCC 52813 (+) and ATCC 52814 (+). Successful endobacteria curation was validated by (i) PCR amplification of the 23S rRNA gene with GlomGiGf [48] and LSU483r [49] primers, (ii) absence of rhizoxin production by high-performance liquid chromatography-mass spectrometry [15, 50], (iii) lack of SYTO9-stained endobacteria under confocal microscopy using the Live/Dead BacLight Bacterial Viability Kit (Invitrogen, #L7012) [51, 52], and (iv) absence of bacterial reads in RNA-seq datasets (see Supplementary File 1 for details).

### Sporulation stimulation in cured strains

In order to explore different nutritive media as inductors of sporulation, potato dextrose agar (PDA), half-diluted PDA (½PDA), potato carrot agar (PCA), yeast peptone glucose agar (YPG), oatmeal agar (OAT), and malt extract agar (MEA) were used. All plates were incubated at room temperature in darkness, 12 h light-cycles or under continuous light [Osram Led White Light bulb (LED CLA100 13 W)] for 5–10 days. Sporulation was monitored periodically by stereoscopic microscope at 10x (LEICA EZ4, Wetzlar, Germany).

Sporangiospores were collected by flooding grown mycelium with 0.1% Tween-20 in phosphate-buffered saline (PBS; OXOID, Hampshire, England), scraping with a loop, and washing twice with PBS. The suspension was filtered through sterile gauze, centrifuged at 5000 × g for 5 min, and washed with distilled sterile water. The resulting spore pellet was used for subsequent assays.

### Phenotypic characterization of *R. microsporus* strains

For radial growth, a 10 μl drop of spore suspension containing 3–5 × 10^3^ sporangiospores was spotted at the center of PDA, ½ PDA, and PCA plates in duplicate. Plates were incubated at room temperature under dark and continuous light exposure. After 48 and 72 h, colony diameters were determined from scanned images using ImageJ/FIJI software version 1.53t [53].

Sporulation rate was quantified by harvesting sporangiospores after measuring mycelial growth and counted using a hemocytometer. The total spore number was normalized to the mycelial area to determine the number of spores per cm^2^ [36]. For determination of time of germination, 1 ml of a 5 × 10^5^ sporangiospores/ml suspension was incubated at 30°C in RPMI 1640 medium (buffered with MOPS, pH 7.0) with constant shaking at 125 rpm. Germ tube emergence was monitored hourly for up to 6 h using optical microscopy. Germination rates were calculated as the number of sporangiospores showing a germ tube out of 100 cells per sample [54], and viability by colony-forming unit (CFU) counting on PDA plates.

Sensitivity to different stress conditions was evaluated by radial growth after seeding 10 μl of sporangiospore suspension (3–5 × 10^3^ sporangiospores/plate) onto the center of PDA plates supplemented with sorbitol (3 M), NaCl (0.5 M), KCl (0.5 M), H_2_O_2_ (200 mM), sodium dodecyl sulfate (SDS; 50 μg/ml), calcofluor white (CFW; 250 μg/ml) or congo red (CR; 1 mg/ml) [27]. Plates were incubated at 30°C for 48 h in darkness, and colony diameters measured using ImageJ/FIJI software. Non-supplemented PDA medium was used as the control.

For melanin and ergosterol quantification, 1 × 10^4^ sporangiospores were inoculated in 200 ml of potato dextrose broth (BD Difco Sparks, MD, USA) and incubated at 30°C and 250 rpm under laboratory conditions (12 h light-cycles), darkness and continuous light exposure for 5 days. The resulting biomass was lyophilized and ground to a fine powder for subsequent analyses. For total melanin quantification, 20 mg of lyophilized biomass, were resuspended in 0.5 ml of 1 N NaOH solution with 10% DMSO (dimethyl sulfoxide) and quantified spectrophotometrically at 405 nm using a NanoDrop 2000 spectrophotometer (Thermo Fisher Scientific, Waltham, MA, USA) [55]. For ergosterol quantification, 60 mg of lyophilized biomass were extracted with 25% (w/v) KOH/EtOH solution prepared by dissolving 25 g of KOH in 35 ml of sterile distilled water and bringing the final volume to 100 ml with 100% ethanol. Then absorbance was measured spectrophotometrically at 230 and 300 nm (Thermo Fisher Scientific, Waltham, MA, USA) [56].

In all assays, spore viability was confirmed by performing serial dilutions of spore suspensions, plating 100 μl onto PDA plates, and incubating at 30°C for 2–3 days prior to colony counting.

### Sexual mating assay

Sexual crosses were performed by placing a mycelial agar plug from the “minus” mating type strain (ATCC 52814) and the “plus” mating type strain (ATCC 52813) on opposite sides of PDA, ½ PDA, and PCA plates in duplicate. Plates were incubated at 30°C in darkness or under continuous light exposure for 10 days. Successful sexual interaction was confirmed by the appearance of a yellow pigmentation line, resulting from β-carotene accumulation, and the formation of zygospores at the contact zone.

### Scanning and transmission electron microscopy

For scanning electron microscopy, mycelia from 7-days PDA cultures were fixed in 2.5% glutaraldehyde for 24 h, post-fixed with 2% osmium tetroxide at 4°C, dehydrated in a graded ethanol series, and infiltrated with amyl acetate. Samples were dried using a critical point dryer (CPD 030, BAL-TEC) and sputter-coated with gold before observation in a Quanta 600 scanning electron microscope (FEI, Eindhoven, Netherlands).

For transmission electron microscopy (TEM), mycelia were embedded in 0.6% agar, cut into 2 × 2 mm cubes, and processed as described previously [57]. Samples were fixed in 2.5% glutaraldehyde—2% paraformaldehyde, post-fixed in 2% osmium tetroxide, dehydrated through an ethanol series, stained with uranyl acetate, and embedded in Spurr’s resin [57]. Ultrathin sections were examined under a transmission electron microscope (JEM 1011, JEOL, Japan) at 80 kV at the Scientific and Technical Resources Service (SRCiT) of the Rovira i Virgili University (see Supplementary File 1 for details).

### Antifungal susceptibility testing

Minimal inhibitory concentrations (MICs) of amphotericin B, voriconazole, posaconazole and itraconazole, all purchased from Sigma–Aldrich (St. Louis, MO, USA) were determined using the Clinical and Laboratory Standards Institute (CLSI) M38-A2 broth microdilution method [58]. The MIC was defined as the lowest antifungal concentration that resulted in complete inhibition of visible growth (100% inhibition for all drugs). *Aspergillus flavus* ATCC 204304 was included as a quality control strain in all assays.

### Transcriptomic analysis

*R. microsporus* strains ATCC 52814 (+) and ATCC 52814 (−) were seeded on the center of ½ PDA plates (3–5 × 10^3^ sporangiospores/plate) and incubated at 30°C in dark or continuous light for 72 h. Mycelia were harvested, immediately frozen in liquid nitrogen, and ground to a fine powder. Total RNA was extracted from 50 mg of mycelia using the NZY Total RNA Isolation kit (NZYtech, Portugal) according to the manufacturer’s instructions, and genomic DNA was removed with DNase I. Samples were obtained from three different sets of plates for each growth condition. RNA integrity was quality-checked using the Bioanalyzer 2100 (Agilent Technologies, Santa Clara, CA, USA). RNA-seq libraries were prepared and sequenced by Novogene using NovaSeq 6000 System (Illumina). Raw reads were quality-checked with FastQC (v0.12.1) and trimmed using Trimmomatic (v0.30) [59]. Reads with Phred quality <33 or length < 20 bp were discarded. Filtered reads were aligned to the *R. microsporus* ATCC 52814 reference genome (available at JGI Mycoscosm Portal: https://mycocosm.jgi.doe.gov/Rhimi_ATCC52814_1) using HISAT2 (v2.2.0) [60]. Raw counts were calculated with featureCounts (v2.0.3) [61] and differential gene expression analysis was performed using DESeq2 (v1.30.0) [62] (*P* value <.05 and |logFC| > 1 as cutoff). Coverage bigWigs were generated with deepTools (v3.1) [63] using 1x coverage normalization for all samples and visualized using Integrative Genomic Viewer (IGV, v.2.17.0) [64]. Heatmaps were produced using the pheatmap R package (v1.0.12; Canberra distance, Ward clustering). GO annotations were retrieved from the *R. microsporus* ATCC 52814 annotation generated in this work and available at Mycocosm (https://mycocosm.jgi.doe.gov/ Rhimi_ATCC52814_1) and evaluated using 2x2 contingency tables for compare the number of genes with/without GO the specific annotation in the gene set of interest versus the background/reference genome. Fisher’s exact test and False Discovery Rate were calculated using R built-in functions. A *P* value of .05 was used as a cutoff for the identification of enriched terms (Fisher’s exact test). IGV (v2.17.0) [64] was used for manual quality control of the data and snapshot generation of selected genes.

The presence of bacterial reads in RNA-seq datasets generated from total RNA isolation from cured and non-cured ATCC 52814 strains were analyzed using Kraken2 software (v2.1.3) [65], which assigns taxonomic labels to every sequencing read in the dataset. Results were inspected for *Mycetohabitans*-derived reads and taxonomic distribution was visualized using Krona (v 2.7.1) [66].

### *In vitro* and *in vivo* virulence assays

The murine macrophage-like cell line J774A.1 was maintained at 37°C with 5% CO_2_ in Dulbecco’s Modified Eagle’s Medium (DMEM; Biowest, Nuaillé, France) supplemented with 10% heat-inactivated fetal bovine serum and 1% penicillin/streptomycin (Biowest, Nuaillé, France). For infection assays, 5 × 10^4^ cells/well were seeded in 24-well plates with serum-free DMEM (sfDMEM) and incubated overnight. Cells were infected with resting or swollen spores (previously incubated for 3 h in DMEM) from cured and non-cured strains of *R. microsporus* at 2.5 × 10^5^ sporangiospores/ml for a multiplicity of infection of 1:5. After 1 h of incubation, the supernatant containing non-phagocytized spores was removed and cultures were incubated with fresh medium for an additional 4 h. Then, macrophages were lysed with sterile cold distilled water, the lysates collected, and plated on PDA to determine the number of intracellular surviving spores, expressed as CFU/ml.

For *in vivo* assay, 1-month-old male Swiss mice weighing 30 g (Animal Facilities Services, University of Murcia, Spain) were housed in groups of ten per cage with food and water *ad libitum*. Mice were challenged intravenously by retroorbital injection of 1 × 10^6^ sporangiospores/animal [67, 68]. *R. microsporus* strain UM1, which is auxotrophic for uracil (*pyrF*^−^), was used as an avirulent control strain [67]. Prior to infection, animals were anesthetized with isoflurane and monitored until full recovery. Mice were checked twice daily for 15 days.

All animal procedures complied with the European Union Council (Directive 2010/63/EU) and Spanish legislation (RD 53/2013) and protocols were approved by the Animal Welfare and Ethics Committee of the University of Murcia and the Department of Water, Agriculture, Farming, Fishing, Environment, and Emergencies of the Government of Murcia (Consejería de Agua, Agricultura, Ganadería, Pesca, Medio Ambiente y Emergencias de la Comunidad Autónoma de la Región de Murcia), Spain (authorization number A13180108).

### Detection of lipase activity

Lipase activity was assessed after 3 days of growth at 30°C in peptone agar medium (peptone 10 g; NaCl 5 g; CaCl_2_·2H_2_O 0.1 g; agar 20 g; distilled water 1 L; phenol red 0.1 g; pH = 6.0). Enzymatic activity was evidenced either by the formation of calcium salt precipitates or by the appearance of a clear yellow halo around the colony, indicating complete degradation of fatty acid salts [69].

### Statistical analysis

All data were analyzed using GraphPad Prism software, Version 8.2.0 for Windows (GraphPad Inc., San Diego, CA USA). Depending on the dataset, nonparametric tests, unpaired *t*-tests, or One/Two-way ANOVA followed by Tukey’s multiple comparisons test were applied, as indicated in the figure legends. All measurements were performed with at least two technical and three biological replicates. Survival rates were plotted in Kaplan–Meier curves, and differences in pathogenicity between fungal strains were analyzed using the log-rank (Mantel–Cox) test. *P* values ≤.05 were considered statistically significant in all assays.

## Results

### Light induces sporulation in endosymbiont-free strains

**Figure 1:**
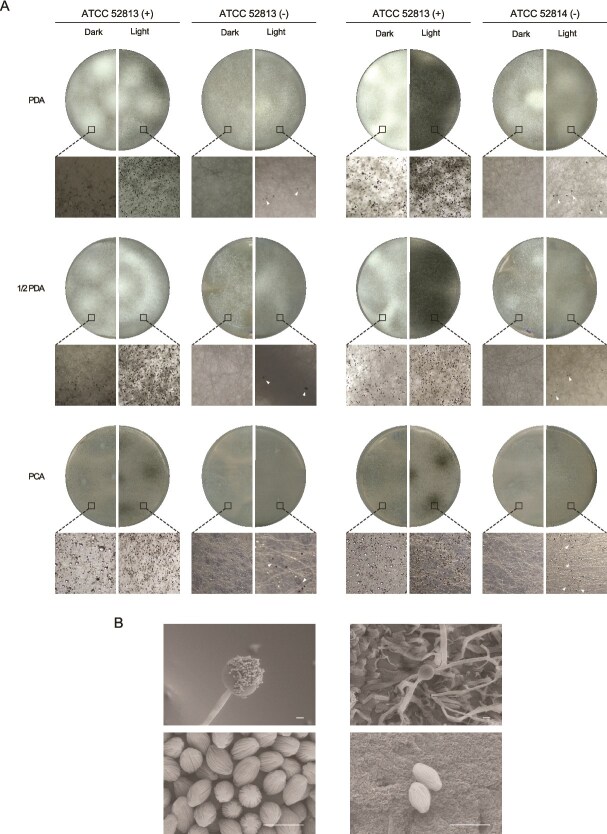
Sporulation induction in cured *R. microsporus* strains by light. (A) Vegetative growth of strains ATCC 52813 and ATCC 52814 with (+) and without (−) endobacteria on different media after 10 days of incubation at 30°C under continuous darkness or light exposure. Representative sporangia and mycelia observed in the stereomicroscope (10x) are shown below each growth plate. The white arrowhead indicates sporangia formation in cured strains. White dots visible in some images correspond to water droplets. (B) Scanning electron microscopy images of sporangium (top) and sporangiospores (bottom) of the strain ATCC 52814 with (left) and without (right) endobacteria (scale bar: 10 *μ*m).

**Figure 2:**
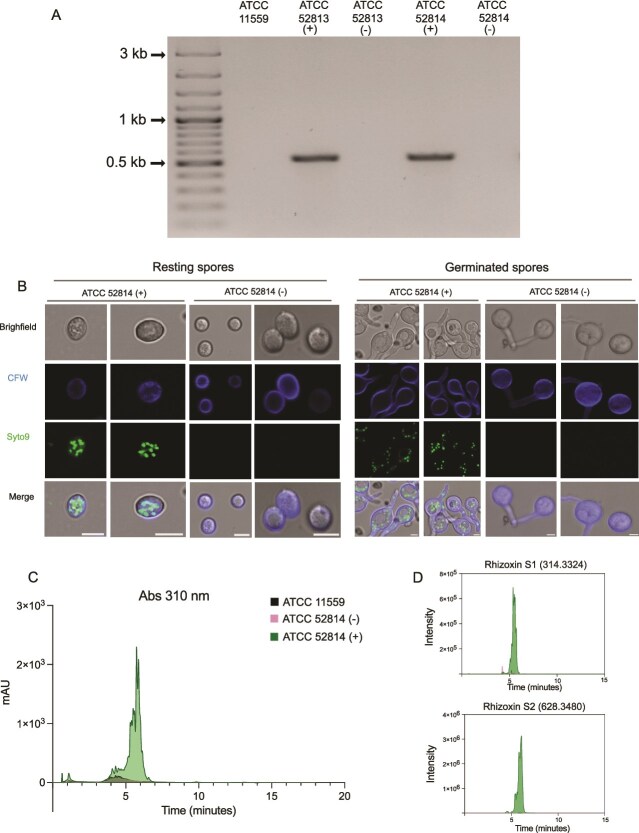
Validation of endobacteria elimination in *R. microsporus* host strains. (A) PCR validation using endobacteria-specific primers for the 23S rRNA gene. (B) *Mycetohabitans* detection in resting and germinated sporangiospores of ATCC 52814 (+) and ATCC 52814 (−). Using confocal microscopy. Endobacteria was visualized with SYTO 9, and fungal cell wall was stained with calcofluor white (CFW). Scale bar = 5 μm. (C) HPLC chromatogram with detection at 310 nm of crude extracts obtained from 4-day-old cultures of *R. microsporus* ATCC 11559, ATCC 52814 (+), and ATCC 52814 (−). ATCC 11559 was used as a negative control, while the *Mycetohabitans*-harboring strain ATCC 52814 (+) served as a positive control. (D) Ion chromatograms for Rhizoxin S1 (m/z 314.3324, top) and Rhizoxin S2 (m/z 628.3480, bottom).

Environmental host strains of *R. microsporus* harboring endosymbionts, such as ATCC 52813 and ATCC 52814, both used in the present study, are unable to sporulate in their absence, whereas non-host strains such as ATCC 11559 do not harbor endobacteria and can sporulate normally [10, 11]. Studying the role of endobacteria in fungal cellular functions and broader phenotypic traits, such as virulence, requires the generation of endobacteria-free sporangiospores. Antibiotic treatment completely eradicated the endosymbionts from both strains as neither 23S rRNA amplification nor rhizoxin production was detected. Confocal microscopy confirmed the absence of bacterial cells in both resting and germinated spores. To evaluate whether nutrient composition could induce sporulation, the cured (−) and non-cured (+) strains, as well as the control strain ATCC 11559, were grown on different media (PDA, ½ PDA, PCA, YPG, MEA 1%, and OAT) under continuous darkness. As expected, control and non-cured strains sporulated abundantly on all tested media (Supplementary Fig. 1). In contrast, cured strains failed to produce sporangia in darkness, confirming that endobacteria are essential for asexual sporulation when cultivated under continuous darkness. Only when exposed to continuous light, sporulation was induced in cured strains in all media, being such effect more pronounced on PCA, ½ PDA, and PDA, where the reduced hyphal density facilitated visualization of sporangia after 10 days (Fig. 1A). Light exposure also enhanced sporulation in non-cured and control strains. Scanning electron microscopy analysis revealed that the sporangia and sporangiospores of cured strains were morphologically indistinguishable from those of non-cured strains (Fig. 1B). To confirm that asexual sporulation observed in cured strains was not due to the presence of residual endobacteria, several complementary analyses were performed. No amplification of the endobacterial 23S rRNA gene was detected in DNA samples from cured strains (Fig. 2A). Likewise, confocal microscopy revealed no bacterial cells in either resting or germinated spores of the cured strains (Fig. 2B). In addition, rhizoxin, a hallmark metabolite synthesized by *Mycetohabitans* endobacteria [70], was undetectable in the cured strains (Fig. 2C and D). Taken together, these results strongly support that the cured *R. microsporus* strains can undergo asexual sporulation when exposed to continuous light.

**Figure 3:**
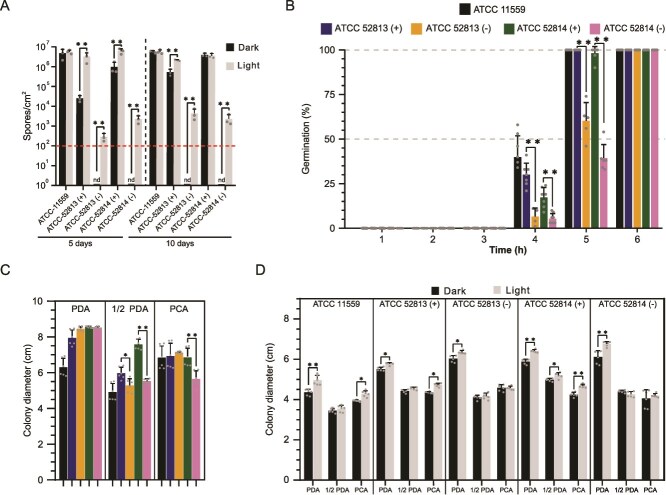
Impact of endobacteria and light on phenotypic changes in *R. microsporus*. (A) Asexual sporulation rate under dark and light conditions on PCA media after 5 and 10 days of growth. The dashed red lines represent the limit for spore counting using a Neubauer chamber. (B) Percentage of germinated spores in RPMI media over 6 h, normalized by spore viability. nd: Indicates not determined values. (C) Effect of different media on mycelial radial growth under dark conditions at 72 h. color legend as in (B). (D) Effect of light and dark on mycelial radial growth on PDA, ½ PDA, and PCA agar plates at 48 h. Bar plots represent the mean ± standard deviation of at least three replicates. Significant differences were determined using two-way ANOVA followed by *post hoc* Tukey’s test; (*) *P* ≤ .05; (**) *P* ≤ .001.

Quantification of sporulation on PCA medium in cured strains, which failed to sporulate in darkness, showed that light exposure induced sporangia formation. This effect became noticeable at shorter incubation times (Fig. 3A), confirming the role of light as a sporulation inducer. Sporangiospore yields in both cured strains (≈10^3^ sporangiospores/cm^2^) remained markedly lower than those of non-cured and control strains (≈10^6^ sporangiospores/cm^2^), indicating that light cannot fully compensate for the absence of the endobacteria-driven regulatory mechanism. Germination assays showed that sporangiospores from cured strains were fully viable, although complete germination was delayed in comparison to their non-cured counterpart (Fig. 3B). Together with scanning electron microscopy observations, these results indicate that sporangia and sporangiospore development proceed normally, both structurally and functionally, in the absence of endobacteria. Furthermore, under 72 h of dark incubation, the presence of endosymbionts significantly enhanced radial growth in ½ PDA and PCA (Fig. 3C), with ATCC 52814 (+) exceeding 2.6 cm/day, whereas its cured counterpart did not surpass 1.85 cm/day under either dark or light conditions (Supplementary Fig. 2). In particular, light markedly increased radial expansion in control and uncured strains, but had no significant effect on the cured strains at 48 h (Fig. 3D).

Endobacteria not only control asexual sporulation but also sexual reproduction [11]. To investigate the role of light in sexual reproduction, sex-compatible strains ATCC 52813 and ATCC 52814 were crossed under continuous light exposure. Non-cured strains mated successfully, whereas cured strains failed (Supplementary Fig. 3), indicating that light cannot substitute endobacterial role in regulating sexual reproduction.

### Sporulation induction by light and endosymbionts share regulatory pathways

**Figure 4:**
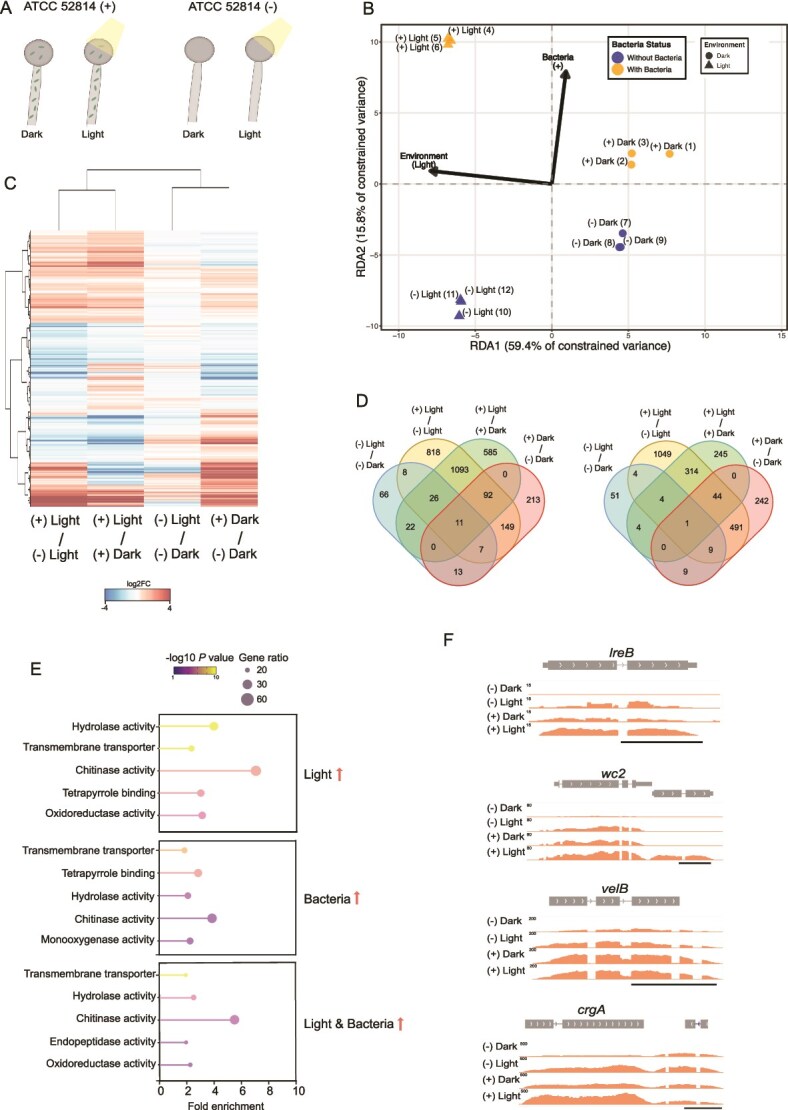
Environmental regulatory mechanisms in response to light exposure and endobacteria presence. (A) Schematic representation of the growth conditions used in this assay. Mycelium from *R. microsporus* cured (−) and non-cured (+) strains grown under dark and light conditions was collected for RNA-seq. (B) RDA plot for multivariance analysis of environmental conditions (light/dark) and bacteria presence or absence. The effect of each component is indicated in the axis. (C) Heatmap of log2FC values for all genes across all comparisons. Clustering was performed using Canberra distance and Ward’s method. (D) Venn diagrams showing intersections between upregulated (left) and downregulated (right) genes. (E) GO enrichment (molecular function) for upregulated genes in light, bacteria, and light + bacteria conditions. (F) Genome browser snapshots showing RNA coverage for the three indicated genes (scale bar = 500 bp). Data represents the analysis of sample triplicates.

To gain insight into the regulatory mechanism governing asexual sporulation in *R. microsporus*, we performed a comparative transcriptomic analysis between ATCC 52814 (+) and ATCC 52814 (−) under continuous dark and light conditions (Fig. 4A). Sequencing reads confirmed the presence of *Mycetohabitans* endosymbionts in non-cured strain, whereas no bacterial reads were detected in the cured strain (Table S1 and Supplementary File 2), validating the successful removal of endosymbionts.

Transcriptomic analysis revealed that the presence of endobacteria was the primary driver of gene expression differences overshadowing the influence of light exposure, with a significant higher number of differentially expressed genes (DEGs) between the cured and non-cured strain in the light than in the dark (Supplementary Fig. 4A). Specifically, bacterial presence explained 59.4% of the constrained variance in gene expression (*F* = 21.4, RDA analysis, *P* < .001), while light conditions accounted for an additional 15.8% (*F* = 6.0, *P* = .002) (Fig. 4B). In addition, endosymbiont enhances the transcriptional response to light as 2441 DEGs were found when comparing light vs. dark conditions in the non-cured strain, whereas only 253 DEGs were detected when comparing light vs. dark conditions in the cured strain (Supplementary Fig. 4A). Interestingly, comparisons of gene expression regulated solely by light or solely by the endosymbiont revealed similar expression patterns. This was particularly evident in the comparison of the non-cured vs. cured strain under light exposure [(+) Light / (−) Light] and non-cured strain in light vs. dark conditions [(+) Light / (+) Dark], which shared the most similar transcriptional profiles (Fig. 4C). These similarities suggest that the signal transduction pathways activated by each stimulus converge on some common regulatory mechanisms. Additionally, several genes shared a common expression pattern between conditions promoting sporulation, either via light in the cured strain [(−) Light / (−) Dark] or via endosymbionts in the dark [(+) Dark / (−) Dark] (Fig. 4C).

To characterize the regulatory pathways underlying environmental responses to light exposure and endosymbiont presence, we analyzed the functional classification of genes regulated by signaling pathways responding to each stimulus. In total, 2608 genes (1923 upregulated and 685 downregulated) responded to light in the cured strain (comparison: (−) Light / (−) Dark) and non-cured strain (comparison: (+) Light / (+) Dark) (Fig. 4D). Analogously, 4697 genes (2430 upregulated and 2167 downregulated) changed their expression in response to the endosymbiont presence (comparisons: (+) Light / (−) Light and (+) Dark / (−) Dark) (Fig. 4D). Functional enrichment analysis revealed that both stimuli, light and endobacteria presence, activated similar biological processes, including transmembrane transport, tetrapyrrole binding, and chitinase and cell wall remodeling activities (Fig. 4E). This suggests that *R. microsporus* activates similar molecular pathways in response to both light exposure and the presence of endosymbionts. The HOG pathway has been reported to be upregulated during the establishment of these symbiotic relationships [71]. We investigated how the components of this regulatory pathway behaved in our expression data. In addition to confirming the activation of the orthologs of Hog1, Ssk1, Gdp1, and diacylglycerol kinases (DGKs) in the comparisons of endobacteria presence/absence, we detected stronger activation of these genes under light conditions compared to dark conditions, as well as the activation of some components, such as Gdp1 and DGKs, in the presence of light even in the absence of bacteria (Supplementary Fig. 4B).

We focused on the genes shared by the (−) Light / (−) Dark and (+) Dark / (−) Dark comparisons, which we considered the core genes necessary for sporulation, we identified 31 upregulated and 19 downregulated genes (Fig. 4D). Among those DEG (Fig. 4F), four genes encoded proteins involved in regulating sporulation in fungi: *cyclin1, wc-2, velB,* and *crgA*. Their encoded proteins were similar to cyclins *Pcl1p* and *Pcl2p*, a White-collar 2 component of a blue light photoreceptor, a regulatory protein VelB, and a RING-finger ubiquitin ligase, respectively. All these genes were significantly upregulated under growth conditions that promoted sporulation, i.e., in the presence of light or endosymbionts, suggesting their importance in this process also in this species.

### Endosymbiont mediates fungal stress resistance

**Figure 5:**
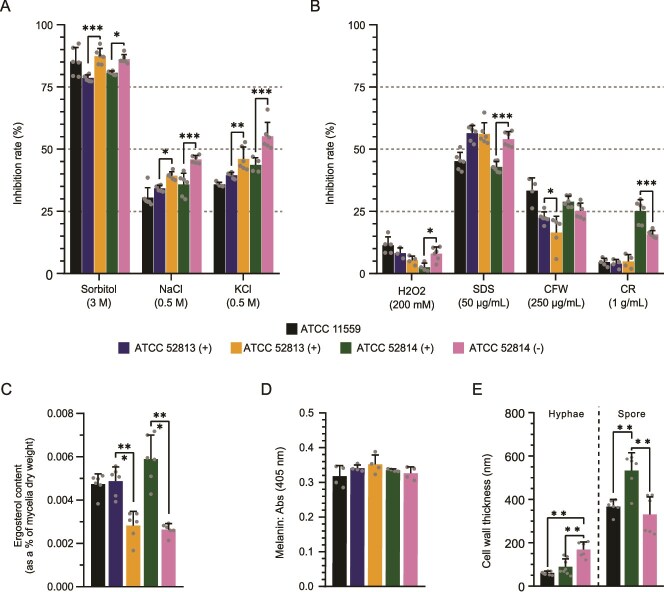
Impact of the endosymbiont on the fungal cell wall and stress tolerance *R. microsporus*. (A) Mycelial growth of *R. microsporus* under osmotic stress. Sporangiospores were seeded and exposed to osmotic stress, and growth rates were measured after incubation at 30°C for 2 days in the dark. (B) Mycelial growth under plasma membrane and cell wall stress. Sporangiospores were subjected to different stress conditions under the same incubation conditions. (C) Quantification of ergosterol content, expressed as a percentage of mycelial dry weight, under 12-h light/dark incubation cycles. (D) Measurement of melanin content, determined by absorbance at 405 nm, under 12-h light/dark incubation cycles. (E) Cell wall thickness analysis using TEM after 7 days on PCA medium. Bar plots represent the mean ± standard deviation of at least three replicates. Statistical significance is indicated as “*” (light vs. dark) and “#” (non-cured vs. cured) and determined using 2-way ANOVA with Tukey’s *post hoc* test: (*) *P* ≤ .05; (**) *P* ≤ .001; (***) *P* ≤ .0001. Student’s t-test was used in (D & E) (*) *P* ≤ .05; (**) *P* ≤ .001; (***) *P* ≤ .0001.

To determine whether endobacteria provides additional advantages beyond sporulation and sexual reproduction that allow the host fungus to outcompete others in its natural niche, we tested the responses of cured strains under various stress conditions. Under osmotic stress (sorbitol, NaCl, and KCl), cured strains displayed significantly reduced growth compared with their corresponding non-cured counterparts (*P* ≤ .032) (Fig. 5A), suggesting that endosymbionts regulate cellular mechanisms that enhance osmotic stress tolerance. Similarly, ATCC 52814 (−) strain, but not ATCC 52813 (−), exhibited increased sensitivity to plasma membrane stressor SDS and oxidative stress by H_2_O_2_ (Fig. 5B). In contrast, exposure to cell wall stressors that interact with chitin, such as CFW and CR, revealed a trend toward increased resistance in cured strains. Accordingly, ATCC 52813 (−) showed significantly lower radial growth than ATCC 52813 (+) under CFW exposure (*P* = .0247), whereas ATCC 52814 (−) displayed significantly lower radial growth than ATCC 52814 (+) under CR exposure (*P* < .001) (Fig. 5B). These results suggest that endobacteria modulate fungal responses to membrane and cell wall stress in a strain- and stress-specific manner, likely to reflect underlying physiological and genetic differences in stress adaptation.

To understand the basis of these altered responses, we analyzed ergosterol content, melanin content, and cell wall structure. Cured strains displayed a significant reduction in ergosterol content (*P* < .0006) compared to their non-cured counterparts, whereas no significant changes were detected in melanin content (Fig. 5C and D), suggesting that decreased ergosterol levels could contribute to their increased sensitivity to membrane stress. Light exposure increased ergosterol and melanin content in all strains, although no significant differences were detected between them (Supplementary Fig. 5A and B). TEM analysis revealed that the sporangiospore wall of the ATCC 52814 (−) strain was significantly thinner than that of the non-cured strain, whereas its hyphal wall was significantly thicker (Fig. 5E, Supplementary Fig. 5C), confirming the presence of structural changes in the cell wall associated with the presence of the endosymbiont.

Despite physiological differences, all strains exhibited similar susceptibility to antifungal drugs targeting ergosterol, including amphotericin B and azoles (voriconazole, posaconazole, itraconazole), showing at most a one-fold change in MIC values between cured, non-cured, and control strains. Even when the spore concentration was reduced to increase sensitivity in MIC detection, no significant changes in antifungal susceptibility were observed (Table S2), suggesting that the reduced ergosterol content observed in cured strains did not markedly increase resistance to ergosterol-targeting drugs. This virulence difference is not due to spore size among ATCC 11559, ATCC 52814 (−), and ATCC 52814 (−).

### Endosymbionts modulate virulence in a murine model

**Figure 6:**
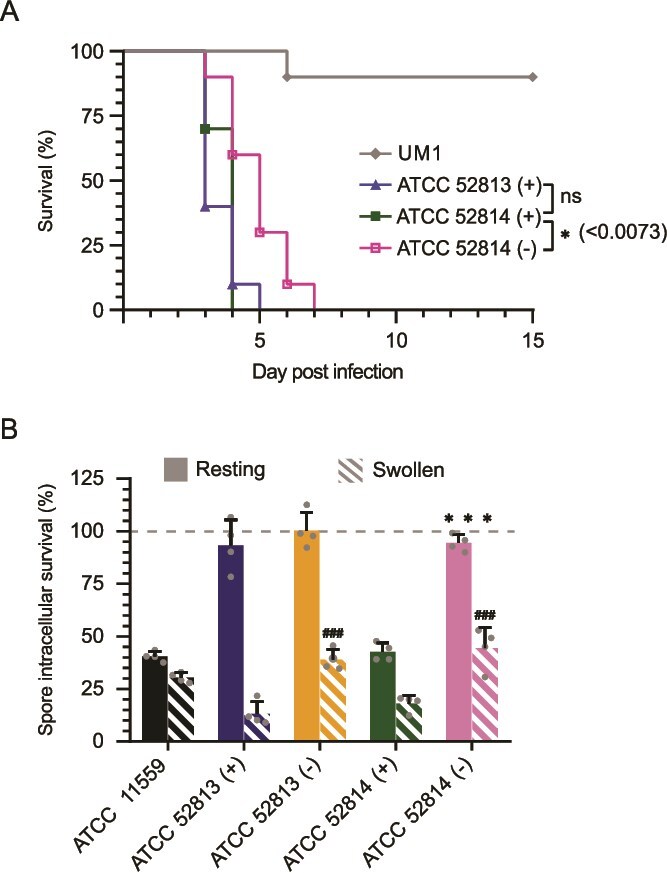
Impact of endosymbiosis on fungal virulence. (A) Survival rates of mice (10 per group) immunosuppressed with cyclophosphamide and infected with cured and non-cured strains of *R. Microsporus.* Strains ATCC 11559 and UM1 were used as the virulent and avirulent controls, respectively. Statistical significance in survival assay was analyzed by a Mantel–Cox test and indicated by *P* values. *P* ≤ .05 (*), ≤ .001 (**) and *P* ≤ .0001 (***). (B) Intracellular survival of *R. microsporus* strains ATCC 11559, ATCC 52813(+), ATCC 52814(+), ATCC 52813(−), and ATCC 52814(−) in J774A.1 macrophages. Intracellular spore viability was determined by quantifying the number of CFUs after 4 h of incubation. Data were normalized to the initial inoculum of spores and represented as the mean ± standard deviation of two independent experiments. Statistically significant differences between groups are indicated by (“*” for resting spores or “#” for swollen (pre-germinating) spores) comparing strain with and without endosymbiont using one-way ANOVA followed by *post hoc* Tukey’s test. (*/#) *P* ≤ .05; (**/##) *P* ≤ .001; (***/###) *P* ≤ .0001.

The plasma membrane and cell wall are critical determinants of pathogenicity, influencing interactions with host immune defenses, tissue adhesion, and stress tolerance [72, 73]. Therefore, the altered plasma membrane composition and cell wall structure observed in cured strains could impact their ability to cause infection in mammals, as previously reported for Mucorales [24] and other fungal pathogens [73]. Moreover, *Mycetohabitans* isolates (formerly *Burkholderia*) are known to produce a diverse family of rhixozin congeners, including numerous structural variants with potential antimitotic activity [70], providing a plausible basis for endobacteria-associated virulence. In our mouse model, the ATCC 52814 (−) exhibited significantly reduced virulence compared to the non-cured counterpart strain (*P* < .007) at 7 days post-infection (Fig. 6A), indicating that the endosymbiont contributes substantially to virulence in mammals. Importantly, this virulence difference is not due to spore size among ATCC 11559, ATCC 52814 (−), and ATCC 52814 (−), with mean ± standard deviation values of 4.57 ± 0.31 μm, 4.41 ± 0.25 μm, and 4.56 ± 0.19 μm, respectively. Although all strains used in the murine model are from environmental origin, they display clear virulence in immunosuppressed mice, consistent with the fact that mucormycosis is mostly acquired from environmental exposure rather than through person-to-person or classical nosocomial transmission.

The observed reduction in virulence is likely linked to the increased sensitivity of cured strains to stressors such as SDS and H_2_O_2_, which could result in lower resistance to macrophage phagocytosis, which plays a crucial role in host defense during Mucorales infections [74, 75]. Consistently, *in vitro* macrophage assays showed that macrophages were less efficient at clearing and killing both resting and swollen sporangiospores from cured strains (Fig. 6B). The most pronounced differences were observed in ATCC 52814 (−), which exhibited 51% lower clearance of resting spores and 26% lower clearance of swollen spores compared with the non-cured strain (*P* < .0001) (Fig. 6B).

**Figure 7:**
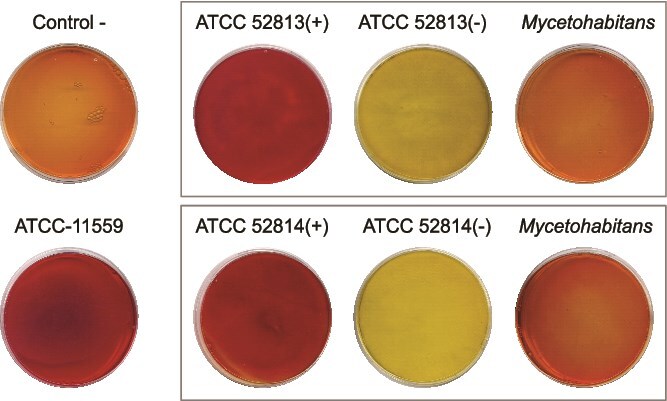
Qualitative extracellular lipase production with Tween-20 and phenol red as pH indicator. The sporangiospores or the endobacteria were inoculated at the center of plate and incubated at 30°C for 72 h. the bacterial strains of *M. endofungorum* used in this study were HKI 0402/B4/B13 and HKI 0403/B7/B14, which were isolated from *R. microsporus* strains ATCC 52813 and ATCC 52814, respectively. Plate without fungi inoculation was used as a negative control (pH = 6). Yellow color surrounding the colonies, resulting from pH reduction due to substrate hydrolysis, indicates lipase activity. Yellow color: Indicates medium acidification (pH < 7); orange color: pH ≈ 7; dark red color: Medium basification (pH >8).

Cured strains exhibited higher lipase secretion compared to non-cured strains or axenic cultures of the endosymbionts (Fig. 7). Given that lipase is a known fungal virulence factor [76–78], these results suggest that the reduced virulence of cured strains is not attributable to decreased lipase production or impaired macrophage interaction. Rather, it likely results from endobacteria-dependent physiological alterations, including changes in membrane composition and delayed spore germination.

## Discussion

Asexual reproduction in environmental isolates of *R. microsporus* that host endobacteria was previously thought to be possible only in the presence of endobacteria [10, 11, 50]. However, in this study, we demonstrate that light can stimulate sporangia development and viable sporangiospores in *R. microsporus* after elimination of endobacteria. This finding indicates that light is an important external factor promoting sporulation in these strains, although its effect remains weaker than that exerted of endobacteria. In addition, it suggests that genes involved in sporulation are likely regulated by both light and the presence of endobacteria. Using a transcriptomic approach, we identified 50 DEGs in response to light and the endosymbiont. Notably, the list of 31 upregulated genes includes *cyclin1*, *wc-2*, *velB*, and *crgA*, all of which regulate sexual and asexual development in fungi [33, 38]. Our findings suggest that these genes are key regulatory elements of asexual sporulation in *R. microsporus* and are likely targets of the signal transduction pathways activated by each stimulus. Given that light-responsive pathways are ancestral in fungi, it is plausible that light originally served as a primary cue regulating sporulation and that endosymbionts may have subsequently evolved to hijack these preexisting light-regulated networks to modulate host sporulation.

VelB is a key component of the Velvet complex, a regulatory system that coordinates fungal development and secondary metabolism in ascomycetes [32, 79, 80]. In darkness, VelB interacts with VeA or VosA to form a nuclear complex that repress conidiation [79–82]. However, exposure to light enhances *velB* expression, promoting VelB-VelB dimerization and consequently activating sporulation [80–82]. The presence of several putative *vosA* orthologs in the *R. microsporus* genome and the upregulation of *velB* by both light and endosymbionts suggest that these genes are likely targets of the signaling pathway trigged by each stimulus to regulate asexual sporulation. Similarly, WC-2 forms together with WC-1 the WCC, a blue light photoreceptor that regulates sporulation in ascomycetes and Mucorales [35, 37, 38, 83]. In *Mucor circinelloides*, light activates the WCC via WC-1 homologs, triggering sporulation, aerial mycelian development, and carotenogenesis. Simultaneously, the RING-finger ubiquitin ligase CrgA represses this pathway in the dark by ubiquitylating the WC-1 homologs (Mcwc-1b). The inactivation of CrgA under illumination thus triggers WCC activity and facilitates developmental gene expression [36, 37]. The gene *crgA* also activates aerial mycelial development in *R. microsporus* non-host strains, but its role in regulating sporulation is not conserved [67]. However, this does not preclude its involvement in sporulation in host strains, as non-host strains have evolved to sporulate independently of endobacteria, potentially leading to divergence in *crgA* function between host and non-host strains.

In addition to regulatory genes, *cyclin1* was also upregulated in response to light and endosymbionts. This gene encodes a protein similar to cyclins Pcl1p and Pcl2p, which are associated with the Pho85 kinase complex, i.e. implicated not only in cell cycle regulation [84, 85] but also in sporulation [84, 86]. Overall, the functions of these four genes in other fungi strongly support their role in sporulation in *R. microsporus* host strains. However, conclusive proof of their involvement in sporulation, as well as their interactions, will require the development of genetic modification techniques for host strains.

According to the mutualism theory [87], the presence of the *Mycetohabitans* endosymbionts can induce extensive physiological changes in their fungal hosts, extending beyond effects on reproduction [4, 6]. Consistent with previous studies [11, 29], we found that removal of the endobacteria significantly reduced radial growth when cultures are initiated with spores and delayed spore germination. Furthermore, the elimination of the endosymbiont reduced resistance to osmotic and oxidative stress, possibly due to alterations in cell wall structure and decreased ergosterol content in the cured strains, reflecting a broader reprogramming of lipid metabolism in the absence of endosymbiont. Endobacteria are known to modulate glycerol and fatty acid pathways, which contribute to membrane integrity and stress responses via the HOG pathway [71]. Their absence may therefore disrupt these pathways, diminishing ergosterol synthesis, impairing membrane fluidity, and altering signaling. Loss of endobacterial chitinolytic activity may also trigger compensatory remodeling of the fungal cell wall and membrane [16]. Notably, this metabolic reprogramming coincides with the observed increase in lipase production in cured strains, suggesting that the fungus adjusts lipid processing and secretion pathways in response to the absence of endobacteria. These findings are consistent with previous reports in *Rhizopus* [52, 71] and in related *Mortierellomycota* and *Glomeromycota* lineages [88–90], where endobacteria influence both cell wall and lipid metabolism, highlighting the evolutionary and ecological significance of these symbiotic interactions. In this context, it is important to note that Carter *et al.* [91] identified a TAL-like effector produced by *Mycetohabitans* that contributes to resistance to the membrane stressor SDS in *R. microsporus* ATCC 52813. In contrast, under our experimental conditions, the cured ATCC 52813 strain did not exhibit increased sensitivity to SDS, whereas cured ATCC 52814 strain showed reduction in SDS tolerance. This apparent discrepancy may reflect strain-specific interactions between particular *Mycetohabitans* lineages and their fungal hosts, differences in effector repertoires. Alternatively, methodological and growth conditions differences between our study and that of Carter *et al.* may also contribute to the distinct SDS phenotypes reported.

Previous studies have reported decreased sensitivity to amphotericin B in cured spores of *R. microsporus* containing *Ralstonia pickettii* [52]. However, the cured strains generated in this work show sensitivity to antifungal agents targeting ergosterol comparable to that of non-cured counterparts, despite their lower ergosterol content. Other factors besides cell wall or membrane composition have been identified as conferring antifungal resistance [92, 93]. Discrepancies with our observations may be attributed to methodological differences, as our study employed the standardized CLSI broth microdilution method, or to differences in the fungus-bacteria system. Despite the documented presence of endosymbionts in some clinical isolates of *R. microsporus* or other Mucorales [17, 18], their contribution to animal pathogenesis remains a largely unexplored yet exciting topic. Recent studies have shown macrophage-mediated killing resistance in *R. microsporus* harboring endobacteria and higher virulence in zebrafish [52]. However, no correlation has been established in humans or mice models, and the involvement of endosymbionts in fungal disease has not yet been demonstrated [94]. In our study, the cured strains exhibit reduced virulence in a mouse model, consistent with previous results in zebrafish and mice infected with spores pretreated with ciprofloxacin before infection [52]. The reduced virulence of the cured strains may be attributed to delayed germination and increased susceptibility to cell membrane stressors. High germination rates have been correlated with enhanced virulence in a pulmonary mucormycosis model of neutropenic rabbits infected with different Mucorales species [95], whereas delayed germination has been associated with attenuated virulence of *M. lusitanicus* in both *G. mellonella* and murine infection models [96, 97]. Similarly, in *M. lusitanicus*, increased sensitivity to SDS has been associated with reduced virulence [27]. Furthermore, the reduced virulence of the cured strains is not linked to lower resistance of resting or swollen (pre-germinating) sporangiospores to macrophage-mediated phagocytosis, the primary host defense mechanism against mucormycosis [74, 75, 98]. On the contrary, our *in vitro* co-culture experiments showed that the absence of the endosymbionts conferred resistance to macrophage-mediated killing. These observations contrast with previous reports of *R. microsporus* strains harboring *R. pickettii* [52], which exhibited higher resistance to macrophages, suggesting that distinct endobacteria may differentially modulate host-pathogen interactions. The increased resistance of cured sporangiospores to phagocytosis-mediated killing may be linked to their reduced ergosterol content, as elevated ergosterol levels can potentiate macrophage-mediated pyroptosis [99–101]. Alternatively, the increased lipase production, observed in the cured strains, may facilitate intracellular adaptation within macrophages without necessarily enhancing systemic virulence in animal models. Furthermore, cured strains displayed lower expression of *wc-2* and *velB* compared to strains harboring endobacteria, and both WCC and VelB have been implicated in the virulence of several filamentous fungi, including Mucorales [102–105]. In addition to these physiological alterations, the increased virulence of strains harboring endobacteria may also be partly explained by the production of rhizoxin congeners by *Mycetohabitans*, which can disrupt host cell microtubule dynamics and contribute to tissue damage during infection [70]. Although the contribution of rhizoxins to mammalian pathogenesis has not been directly demonstrated, their cytotoxic properties provide a plausible basis for the enhanced virulence in endosymbiont-containing strains. Our results, although obtained from environmental isolates that display pathogenic ability in our immunosuppressed mouse model, highlight the need to investigate clinical strains to elucidate potential mechanisms of endobacteria-associated pathogenesis. Such mechanisms remain speculative as both *in vitro* and *in vivo* studies in clinical isolates are still lacking to reach a robust conclusion.

Overall, this study reveals the complex interplay between light, endosymbionts, and gene regulation in *R. microsporus*, providing new insights into the mechanisms driving sporulation and demonstrating the influence of endosymbionts on fungal fitness and virulence, with potential implications for understanding pathogenicity in Mucorales.

## Supplementary Material

nSupp_Fig_1_wrag047

nSupp_Fig_2_wrag047

nSupp_Fig_3_wrag047

nSupp_Fig_4_wrag047

nSupp_Fig_5_wrag047

nSupp_Table_1_wrag047

nSupp_Table_2_wrag047

Supplementary_File_1_wrag047

Supplementary_File_2_wrag047

## Data Availability

Raw data files generated by this work are deposited in the NCBI Gene Expression Omnibus (GEO) repository and are publicly available through the project accession number GSE289616. The accession numbers of each assay condition used in this study are as follows: GSM8795093, GSM8795094, GSM8795095 (cured strain dark-cultured); GSM87950956, GSM8795097, GSM8795098 (cured strain light-cultured); GSM8795099, GSM8795100, GSM8795101 (host strain dark-cultured); and GSM8795102, GSM8795103, GSM8795104 (host strain light-cultured).
