# Supplementary material for: Light restores sporulation in Rhizopus microsporus cured of its endosymbionts, unveiling their role in fitness and virulence
Source: ISME J. 2026 Apr 8;20(1):wrag047. doi: 10.1093/ismejo/wrag047 (PMC13143264; doi:10.1093/ismejo/wrag047)

Supplementary Figure 1. Growth of *R. microsporus* on different media incubated in the dark at 30 °C for 5 days. Media included PDA (Potato Dextrose Agar), YPG (Yeast Extract Peptone Glucose), ½ PDA (half concentration of PDA), PCA (Potato Carrot Agar), MEA (Malt Extract Agar), and OAT (Oatmeal Agar). Sporulation is shown by dark spots or grains on the agar plate, with complete darkening of the plate indicating heavy sporulation.

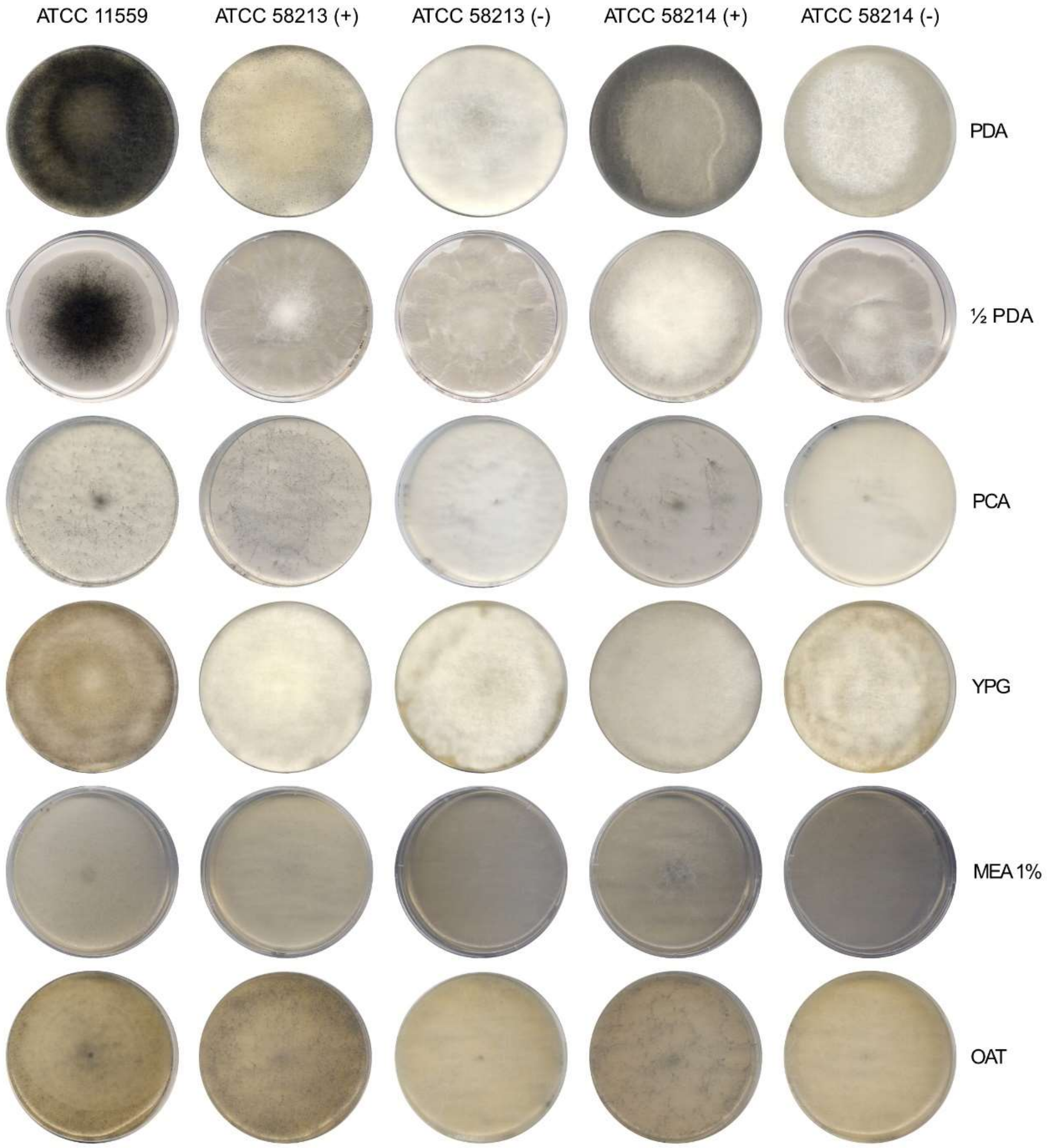

Supplement: nSupp_Fig_1_wrag047 [file nsupp_fig_1_wrag047.pdf]
