# Supplementary material for: Light restores sporulation in Rhizopus microsporus cured of its endosymbionts, unveiling their role in fitness and virulence
Source: ISME J. 2026 Apr 8;20(1):wrag047. doi: 10.1093/ismejo/wrag047 (PMC13143264; doi:10.1093/ismejo/wrag047)

Supplementary Figure 2. Radial growth rate depending on media and light conditions for 72h. (A) Radial fungal growth per day incubating media in dark conditions. **(B)** Radial fungal growth per day incubating media in light exposure. Box and whisker blots represent the median ± maximum/minimum colony diameter of strains grown on PDA, ½PDA and PCA, incubating at 30 °C. Box plots represent the mean ± standard deviation of at least three replicates. Statistical significance was determined using two-way ANOVA followed by Tukey's tests (*) $P \leq 0.05$; (**) $P \leq 0.001$; (***) $P \leq 0.0001$.

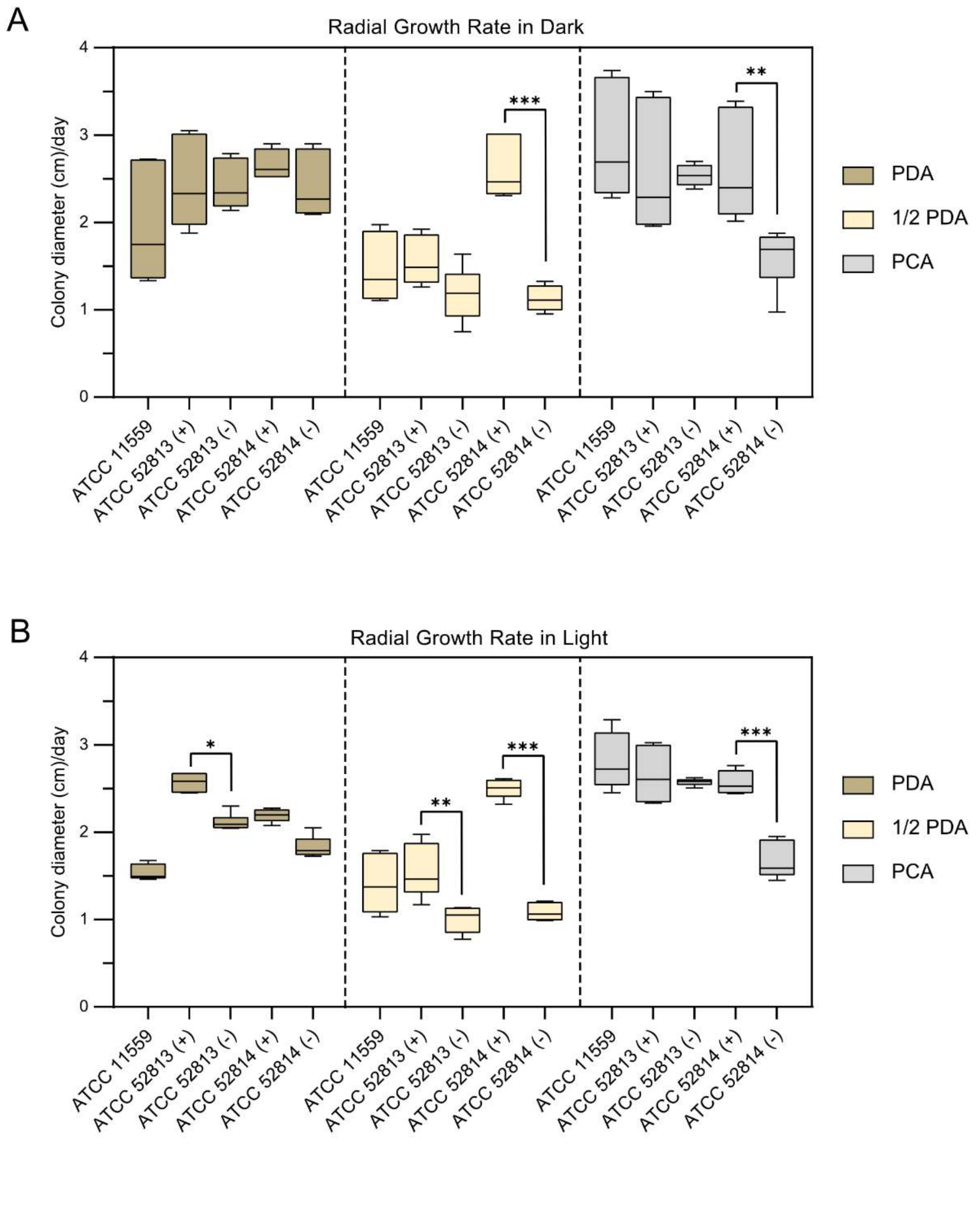

Supplement: nSupp_Fig_2_wrag047 [file nsupp_fig_2_wrag047.pdf]
