# Supplementary material for: Light restores sporulation in Rhizopus microsporus cured of its endosymbionts, unveiling their role in fitness and virulence
Source: ISME J. 2026 Apr 8;20(1):wrag047. doi: 10.1093/ismejo/wrag047 (PMC13143264; doi:10.1093/ismejo/wrag047)

Supplementary Figure 3. Impact of *Mycetohabitans* and light on sexual development and β-carotene production in sex-compatible *R. microsporus*. (**A**) Schematic representation of the mating assay using PDA medium. (**B**) Successful mating and β-carotene accumulation between non-cured strains under dark and light conditions after 10 days of culture. The presence of an orange halo at the interaction zone indicates β-carotene production. **(C)** Mating interactions between sex-compatible cured strains.

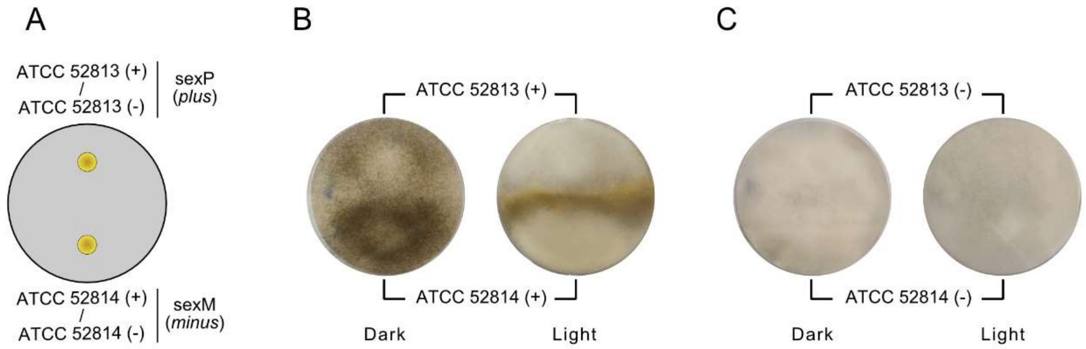

Supplement: nSupp_Fig_3_wrag047 [file nsupp_fig_3_wrag047.pdf]
