# Supplementary material for: Light restores sporulation in Rhizopus microsporus cured of its endosymbionts, unveiling their role in fitness and virulence
Source: ISME J. 2026 Apr 8;20(1):wrag047. doi: 10.1093/ismejo/wrag047 (PMC13143264; doi:10.1093/ismejo/wrag047)

Supplementary Figure 4. Transcriptomic changes in cured and non-cured *R. microsporus* exposed to light. (A) Volcano plots displaying log2FC expression and *P* values for all indicated comparisons. **(B)** Heatmap indicating log2FC values for the comparison indicated above for HOG pathway-related genes.

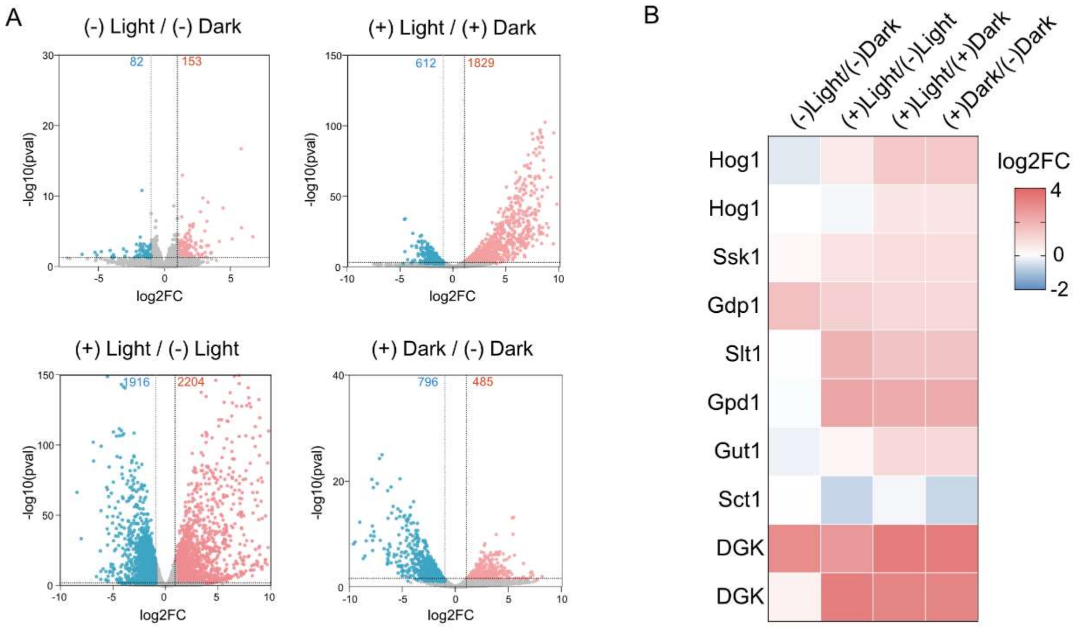

Supplement: nSupp_Fig_4_wrag047 [file nsupp_fig_4_wrag047.pdf]
