# Supplementary material for: Light restores sporulation in Rhizopus microsporus cured of its endosymbionts, unveiling their role in fitness and virulence
Source: ISME J. 2026 Apr 8;20(1):wrag047. doi: 10.1093/ismejo/wrag047 (PMC13143264; doi:10.1093/ismejo/wrag047)

Supplementary Figure 5. Impact of light on the fungal cell wall of *R. microsporus*. (**A**) Quantification of the differential ergosterol content, expressed as a percentage of mycelial dry weight, between light- and dark-incubated cultures. (**B**) Assessment of melanin content variation (absorbance at 405 nm) in response to light versus dark incubation. Bar plots represent the mean ± standard deviation of at least three replicates. Statistical significance was determined using one-way ANOVA followed by Tukey's tests (*) $P \leq 0.05$; (**) $P \leq 0.001$; ns (not statistically significant). (**C**) Total fungal cell wall thickness observed by TEM. Representative cell wall of cured and non-cured *R. microsporus*.

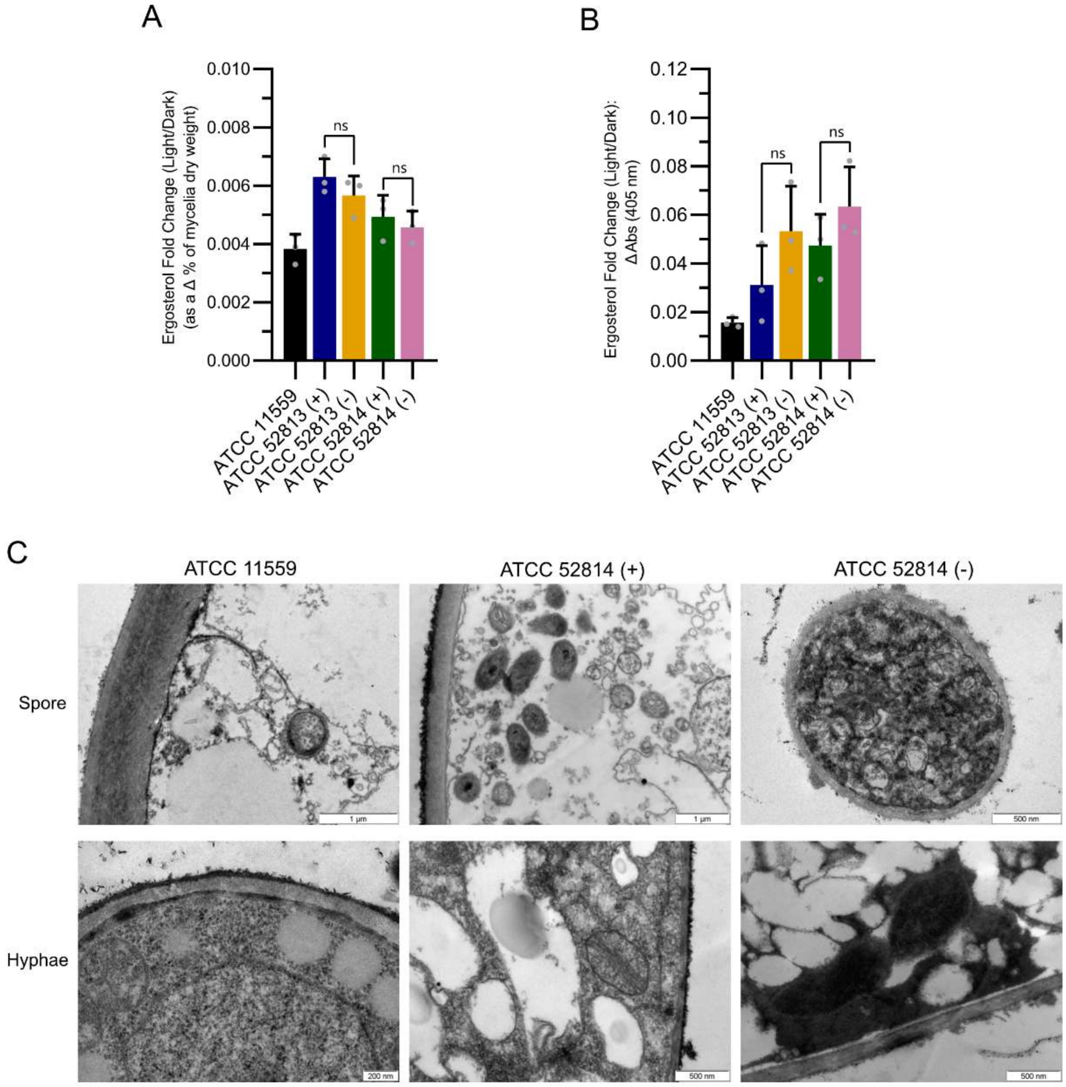

Supplement: nSupp_Fig_5_wrag047 [file nsupp_fig_5_wrag047.pdf]
