# Supplementary material for: Light restores sporulation in Rhizopus microsporus cured of its endosymbionts, unveiling their role in fitness and virulence
Source: ISME J. 2026 Apr 8;20(1):wrag047. doi: 10.1093/ismejo/wrag047 (PMC13143264; doi:10.1093/ismejo/wrag047)
Supplement: nSupp_Table_1_wrag047 [file nsupp_table_1_wrag047.docx]

**Supplementary Table S1: Taxonomic analysis of RNA-seq reads for each sample.** The table shows the total number of reads per sample, along with the number and percentage of reads assigned to *R. microsporus* relative to the total read count. Similarly, the number of reads assigned to *Mycetohabitans* is indicated, including both the percentage of total reads and the percentage relative to all reads assigned to Bacteria.

| **Sample** | Total Reads | Total *R. microsporus* Reads | % of Total Reads (Fungi) | Total *Mycetohabitans* Reads | % of Total Reads (Bact) | % of Bacterial Reads |
| --- | --- | --- | --- | --- | --- | --- |
| **(-) Dark (7)** | 33089558 | 31213697 | 94.33 | 0 | 0 | 0 |
| **(-) Dark (8)** | 21629050 | 19913717 | 92.23 | 0 | 0 | 0 |
| **(-) Dark (9)** | 33019595 | 31001817 | 93.89 | 0 | 0 | 0 |
| **(-) Light (10)** | 26562806 | 24930650 | 93.86 | 0 | 0 | 0 |
| **(-) Light (11)** | 29576263 | 27493874 | 92.96 | 0 | 0 | 0 |
| **(-) Light (12)** | 27456966 | 25393511 | 92.48 | 0 | 0 | 0 |
| **(+) Dark (1)** | 25866943 | 24210072 | 93.59 | 5259 | 0.02 | 11 |
| **(+) Dark (2)** | 28212015 | 26288196 | 93.18 | 15052 | 0.05 | 20 |
| **(+) Dark (3)** | 28139057 | 25864008 | 91.91 | 10286 | 0.04 | 14 |
| **(+) Light (4)** | 26597721 | 23578866 | 89.69 | 129378 | 0.49 | 65 |
| **(+) Light (5)** | 24739413 | 22581100 | 91.28 | 80941 | 0.33 | 60 |
| **(+) Light (6)** | 24598253 | 22633811 | 92.01 | 74447 | 0.30 | 56 |
