# Supplementary material for: Light restores sporulation in Rhizopus microsporus cured of its endosymbionts, unveiling their role in fitness and virulence
Source: ISME J. 2026 Apr 8;20(1):wrag047. doi: 10.1093/ismejo/wrag047 (PMC13143264; doi:10.1093/ismejo/wrag047)
Supplement: nSupp_Table_2_wrag047 [file nsupp_table_2_wrag047.docx]

**Supplementary Table S2. Antifungal susceptibility of cured and non-cured *R. microsporus*.** Minimal Inhibitory concentrations (MICs) of Amphotericin B (AmB), Voriconazole (VRC), Posaconazole (PSC) and Itraconazole (ITC) were determined using the CLSI broth microdilution method, inoculating 10^4^ sporangiospores/mL and 10^3^ sporangiospores/mL, respectively, for each antifungal agent.

| **Strain** | **AmB** | | **VRC** | | **PSC** | | **ITC** | |
| --- | --- | --- | --- | --- | --- | --- | --- | --- |
|  | 10^4^ spores/mL | 10^3^ spores/mL | 10^4^ spores/mL | 10^3^ spores/mL | 10^4^ spores/mL | 10^3^ spores/mL | 10^4^ spores/mL | 10^3^ spores/mL |
| **ATCC 11559** | 0.5 | 0.25 | 32 | 8 | 2 | 1 | 4 | 1 |
| **ATCC 52813 (+)** | 0.5 | 0.25 | 64 | 32 | >64 | 4 | >64 | 4 |
| **ATCC 52813 (-)** | 1 | 0.5 | >64 | 32 | >64 | 4 | >64 | 2 |
| **ATCC 52814 (+)** | 0.25 | 0.25 | 64 | 32 | 4 | 4 | 8 | 4 |
| **ATCC 52814 (-)** | 1 | 0.5 | >64 | 32 | 4 | 2 | 8 | 2 |
