# Supplementary material for: Light restores sporulation in Rhizopus microsporus cured of its endosymbionts, unveiling their role in fitness and virulence
Source: ISME J. 2026 Apr 8;20(1):wrag047. doi: 10.1093/ismejo/wrag047 (PMC13143264; doi:10.1093/ismejo/wrag047)
Supplement: Supplementary_File_1_wrag047 [file supplementary_file_1_wrag047.docx]

# **MATERIALS AND METHODS**

## **Confirmation of elimination of endobacteria in fungal strains**

## **Analysis of cured strains by PCR amplification**

Total genomic DNA was extracted from 5-day-old mycelia (0.5 cm² fragments) grown on PDA at 30 °C in darkness, following a modified protocol of Müller *et al*. [1], and quantified using a Nanodrop 2000 (Thermo Scientific, Madrid, Spain). Detection of endosymbionts was conducted by amplification of 23S rRNA gene using GlomGiGf (GGGTCCATTGCGGATTACTTC) [2] and LSU483r (GGTGCAGGAATATTAACC) primers [3]. The presence of endobacteria was confirmed by the visualization of a single ~553 bp amplicon after agarose gel electrophoresis.

## **Confocal microscopy**

The presence of endobacteria in resting and germinated sporangiospores of both non-cured and cured strains of *R. microsporus* ATCC 52814 was assessed using the Live/Dead BacLight Bacterial Viability Kit (Invitrogen, #L7012), which includes the SYTO 9 stain commonly used for endobacteria detection, following previously established protocols [4, 5]. A total of 1 × 10⁶ sporangiospores and germinated sporangiospores from cured and non-cured ATCC 52814 (grown under light exposure) were washed twice with PBS and resuspended in 50 µl of 0.85% NaCl. Samples were incubated for 5 min under light in the presence of 2 µg/ml calcofluor white (Merck, #18909) and 3 µl of SYTO 9, mounted on microscope slides, and observed under a Leica STELLARIS confocal microscope (Leica) with excitation at 483 nm and 350 nm, and emission collected at 503 nm and 432 nm, respectively. For germinated sporangiospores, spores were incubated in liquid PDB medium at 30 °C for 6 h in a rotary shaker (250 rpm) before staining and imaging as described above.

## **Rhizoxin determination**

Rhizoxin extraction and detection were performed according to previously established protocols [6, 7]. Briefly, 1 x 10^5^ sporangiospores of *R. microsporus* ATCC 11559, and cured and non-cured strains of ATCC 52814, were cultured in liquid PBD medium at 30 °C for 4 days under shaking (250 rpm). One volume of ethyl acetate (Merck, #319902) was added to each culture and vigorously mixed for 1 h. The aqueous phase was discarded, and the organic phase was collected and evaporated using a rotary evaporator (Heidolph VV2000) under reduced pressure at >40 °C. The resulting residue was dissolved in 5 ml of HPLC-grade methanol (Merck, #34860) and concentrated to 500 µl in a SpeedVac concentrator (Eppendorf). Chromatographic separation and mass spectrometric analyses were performed on an Agilent 1290 Infinity II Series HPLC system (Agilent Technologies, Santa Clara, CA, USA) coupled to and Agilent 6550 Q-TOF Mass spectrometer with an AJS-Dual ESI interface (Agilent Technologies, Santa Clara, CA, USA). System parameters were configured using MassHunter Workstation Data Acquisition software (Agilent Technologies, Rev. B.08.00). Samples (20 µl) maintained at 5 °C were injected onto a Phenomenex Luna Omega C18 (2.1x100 mm, 1.6 um) HPLC column. Absorbance was monitored at 310 nm. The Q-TOF operated in positive mode with a nebulizer pressure of 30 psi, drying gas at 16 l/min and 150°C, sheath gas at 11 l/min and 300 °C, and voltages of 4000 V (capillary), 500 V (nozzle), 360 V (fragmentor), and 750 V (octopole 1 RF Vpp). Data were acquired in centroid move over 50-1500 m/z at 4 spectra/s and 250 ms/. Reference masses at 121.0509 and 922.0098 were used for real-time mass correction. Data were processed using MassHunter Qualitative Analysis Navigator software (Agilent Technologies, Rev. B.08.00). Extracted-ion chromatograms were examined at 614.3324 m/z (Rhizoxin S1, C_34_H_47_NO_9_) and 628.3480 m/z (Rhizoxin S2, C_35_H_49_NO_9_).

## **Scanning and transmission electron microscopy**

For scanning electron microscopy (SEM), small pieces of agar containing mycelia samples (with sporangia) were collected from 7-day old PDA cultures and fixed in 2.5% glutaraldehyde in 0.1 M phosphate buffer (pH 7.4) for 24 h. Samples were post-fixed in 2% osmium tetroxide for 2 h at 4°C, dehydrated through a graded ethanol series, and infiltrated with amyl acetate. Specimens were dried using a critical point dryer (CPD 030, BAL-TEC, Balzers, Liechtenstein), sputter coated with gold, and examined using a Quanta 600 scanning electron microscope (FEI Company, Eindhoven, Netherlands).

For transmission electron microscopy (TEM) analysis, samples were prepared as described previously [8] with some modifications. Mycelia from 7-day old PDA cultures (with sporangia were carefully collected, embedded in 0.6% semisolid agar, and cut into 2 x 2 mm cubes. Samples were fixed with 2.5% glutaraldehyde - 2% paraformaldehyde in 0.1 M phosphate buffer for 24 h, post-fixed in 2% osmium tetroxide for 2 h at 4 °C, and dehydrated through a graded ethanol series (30% to 100%). After dehydration in 50% ethanol, samples were stained with 2.5% uranyl acetate in 70% ethanol for 24 h and embedded in Spurr's resin. Ultrathin sections were obtained with an ultramicrotome (Leica-Reichert Ultracut E, Leica Microsystems, Inc., Wetzlar, Germany) using a diamond knife and examined with JEM 1011 transmission electron microscope (JEOL, Japan) operating at 80 kV. Images were using a MegaView II CCD camera (analySIS). Approximately, twenty micrographs per strain were randomly selected for measurement of cell wall thickness and observation of endosymbionts using ImageJ/FIJI software. The procedure was repeated twice with independent fungal preparations.

All electron microscopy analyses were performed at Scientific and Technical Resources Service (SRCiT), Rovira i Virgili University, Spain.

# **REFERENCES**

1. Müller FMC, Werner KE, Kasai M, Francesconi A, Chanock SJ, Walsh TJ. Rapid Extraction of Genomic DNA from Medically Important Yeasts and Filamentous Fungi by High-Speed Cell Disruption. *J Clin Microbiol* 1998;36:1625.

2. Bianciotto V, Genre A, Jargeat P, Lumini E, Bécard G, Bonfante P. Vertical transmission of endobacteria in the arbuscular mycorrhizal fungus *Gigaspora margarita* through generation of vegetative spores. *Appl Environ Microbiol* 2004;70:3600–3608.

3. Mondo SJ, Toomer KH, Morton JB, Lekberg Y, Pawlowska TE. Evolutionary stability in a 400-million-year-old heritable facultative mutualism. *Evolution* 2012;66:2564–2576.

4. Liu XL, Zhao H, Wang YX, Liu XY, Jiang Y, Tao MF, et al. Detecting and characterizing new endofungal bacteria in new hosts: *Pandoraea sputorum* and *Mycetohabitans endofungorum* in *Rhizopus arrhizus*. *Front Microbiol* 2024;15:1346252.

5. Itabangi H, Sephton-Clark PCS, Tamayo DP, Zhou X, Starling GP, Mahamoud Z, et al. A bacterial endosymbiont of the fungus *Rhizopus microsporus* drives phagocyte evasion and opportunistic virulence. *Curr Biol* 2022;32:1115-1130.e6.

6. Partida-Martinez LP, Hertweck C. Pathogenic fungus harbours endosymbiotic bacteria for toxin production. *Nature* 2005;437:884–888.

7. Richter I, Radosa S, Cseresnyés Z, Ferling I, Büttner H, Niehs SP, et al. Toxin-Producing Endosymbionts Shield Pathogenic Fungus against Micropredators. *mBio* 2022;13:e0144022.

8. Glauert AM, Lewis PR. Biological Specimen Preparation for Transmission Electron Microscopy. *Princeton University Press*, 1998.
