# Supplementary material for: Light restores sporulation in Rhizopus microsporus cured of its endosymbionts, unveiling their role in fitness and virulence
Source: ISME J. 2026 Apr 8;20(1):wrag047. doi: 10.1093/ismejo/wrag047 (PMC13143264; doi:10.1093/ismejo/wrag047)
Supplement: Supplementary_File_2_wrag047 [file supplementary_file_2_wrag047.html]

Javascript must be enabled to view this page.

magnitude
magnitudeUnassigned

Report\_POS\_DA1
Report\_NEG\_LI12
Report\_NEG\_LI11
Report\_NEG\_LI10
Report\_NEG\_DA9
Report\_NEG\_DA7
Report\_POS\_LI6
Report\_POS\_LI5
Report\_POS\_LI4
Report\_POS\_DA3
Report\_POS\_DA2
Report\_NEG\_DA8

258325302736230929463173265006633294979633030607245319162464955826454818280942902816934821573160

327316993212881733515419210993482062920107020618336137378

021010101100
25861882198415642456146874510981031326228002923

100000000000
6981104478365160163195889563

111
000

111

1

3

111
000

111
000

000
111

11

1

1

000000000000
6780103478365157163194879163

25178827242
00000000000

00000000000
25178827242

1122121
0000000

2111

1111

1

14156827121
00001000000

1111

4347611

21121

11
00

00
11

11

000000000000
6575102477656149161187858661

000000000000
6575102477656149161187858661

1
0

1

000000000000
596489376448141151165675750

1111121

1

1

1

1

112

586386356347140150163675549

000000000000
611131012881022182811

611131012881022182811

1

1

17721275125310161733848439626564246820471935
021112001100

11

00
11

11
00

11
00

10
11

1

12

0
1

1
0

0
1

0
1

1

1657117411459541631768307502436234119161860
000000000000

7574778250402638411269671
000000000000

7574778250402638411269671
000000000000

000000000000
7574778250402638411269671

11

7573778250402538411269671

000000000000
46192515374493565839275

0000000
1513211

1513211
0002010

1311

111

11

1
0

0
1

1

45182414373992525436265
000000000000

0
1

1

00
11

11

000000000000
45172412373890505332234

1

45172412363890505332234

1
0

1

1
0

1

000000
221421

221421

11122
00000

112
000

11

1

1

12
00

12

111
000

111
000

000
111

111

000000000000
1536107910428561541684188406336217517931784

000000000000
1518105810308471522673177394332215317811780

000000000000
1505104910168331511661150349294213017521768

1505104910168331511661150349294213017521768

0
1

1

010100130000
95675227403121228

21313212

11

11211

11

23113263

5122112032271284

111265

0
1

1

1111
0000

1

111

1
0

1

111
000

1

11

00001001010
33676936263

1

23475612153

2

1

13

13

142112819111012316103

411151

1

2

2321
0000

2321
0000

0000
2321

2311

1

111

00000000000
985241243724

00000000000
985241243724

00000000000
985241243724

58522833511
01101002000

1

1

12

1

11

2

1111

2

1

1

311

1

11

21

2

21

11

1

1

321213
000000

11

2112

1

1

1

1

14
00

14

000000000000
787579487262737190839337

787579487262737190839337
000000000000

00000000000
371552354661

11
00

11

371551354651
00000000000

371551353651

1

111
000

00
11

1

1

0
1

1

1531257723
0000000000

257712
000000

257712

11
00

11

1
0

1

00
11

11

1121
0000

1

1

1

1

1

00
11

1

1

0000000000
43841024853

3274811643
0010000000

23713

1

1

36162

11

1
0

1

00000101
11121221

1

111

1

11

11

111

11664211472010117
000000000000

00100000
12222421

1

1

11

13

121

2

1

5
0

5

221124
000000

11

121123

000
111

1

1

1

1122
0000

2

1

1

11

1
0

1

0
1

1

1111
0000

1

1

11

0000
1111

11

11

00
11

11

43222621
43221611

1

1

00
13

11

2

00
22

22

11
00

11

000
111

111

004000
124111

1

2

11

1

771627725202214179
000000000000

000000
121731

1

1

1

631

1

1

1132111
0020000

1

2111

1

1

3221
0000

1

322

0000000
3233321

3233321

111
000

1

1

1

47221665566
0511953001

1

1

12151365

12

1

22

13112
00010

111

1

3

00
21

21

11

211
000

11

11

1
0

1

00000
87611

87511

1

514631335037332933425618
000000000000

000000000000
32211315211416151128357

22112

32191315191416141127355

511551914956143
000000001000

11

11

37431812854113

11

2112

1

111

1

00
22

22

000000000000
544644237887

212

444644216687

1

1
0

1

12231
00000

1131

1

2

00
11

11

0
1

1

01011
11111

1

1

11211
00000

111

12

00
31

1

2

1

00
21

2

1

11
00

11

5244115147
0000000000

5244115137

1

22221
00000

22221

00
11

11

1

1

1

1

134118118263421222022
000000000000

000000000000
134118118263421222022

000000000000
134118118263421222022

32513252317115
44735392517129

12

111

3

1

11211

2

111

141426246144
00000000000

111

2325166134

11118

0211000000
4423976437

3

4212946437

421412
000100

1

1

1

11

21

1

22

000000000000
14712296171113111411

000000000000
14712296171113111411

00000000000
45829123956

00000000000
45829123846

35829111546

21

1

11

1

00
11

11

0000000000
1024517910195

00
21

21

0000000000
1024316910195

1

1024315910195

0
1

1

1

12111122
00000000

12111122
00000000

00000000
12111122

00000
11111

11111

111111
000000

1111

11

1

1

1111
0000

1111

13341461341554
10000010000

12241461241554

1
0

0
1

0
1

0
1

1

21

00
21

0
1

1
0

1
0

1

1
0

0
1

0
1

0
1

1

1

0000000000
1142143132

1142112112
0000000000

1111212

13111

312

000000000000
675485556460578514115256215639600803

0000000
1211122

0000000
1211122

1211122
0000000

0000000
1211122

1211122

000000000000
675479554459578513115255215637600801

0000000000
2132126331

22211323
00000000

1
0

1

11

00
11

11

1
0

1

11111
00000

1

1

1

11

121
000

1

11

1

1
0

1

111311
000000

00
11

11

1
0

1

00000
11111

11111

000000000000
4763323311532

4763323311532
000000000000

2
0

1

1

000000000000
4761323311532

266131339232

21112

11

11111221
00000000

11111221
00000000

0000
1121

1

111

1

11111
00000

11

11

1

13
00

0
3

3
0

1

2

0
1

1
0

1

0
1

0
1

1
0

1

000000000000
668470544455573509109246200624595797

15316019015820813469135149239180118

5153103542973653754011151385415679
001000001000

000000000000
5083043482973633743310441372406676

5083043482973623743310341372405676

111

000000
111131

1

111

112

42222
10000

32

1

1

112

1
0

1

0000
1111

111

1

110
331

1

1

2

1

2212262
0000000

62

12112

11

0000000100
4231111421

111

11

3111

1111

1111

5

212030111714101810261749
000000000000

000
111

111

000000000000
20203011161491610251649

189111121328113830
000000000000

11
00

1

0
1

1

11

16910121328113830
00000000000

0000000000
1551012137112730

1551012137112730

142111
000000

12111

4

321113

011156003678512
21119101157811816

1

12411224

1111
0000

1111
0000

1111

1111
0000

1111
0000

11

11

1111
0000

1111

121221
000000

121221

0000
1111

11
00

11
00

00
11

00
11

11

11

0
1

1

13311333231

2145628462
0000000000

0000
1224

1224
0000

0000
1224

0000
1224

1224

0000
1211

1211

1142615222

27142618291761322152561
000000000000

27142618291761322152561

11
00

1
0

1
0

0
1

1
0

1

1
0

1

1

0
1

0
1

1
0

0
1

1
0

1

3

11312

4944611123911871010003297508107112167577271692473293710335600763445
245672442587584628047978253949643151941931746378232560032326244324897536263953242670703520274121

0000000
11412612

00000
113262

11142
00000

11142
00000

1

1112

12

0
1

1
0

1

1211
000

00
121

1

12

1

000
111

000
111

1

11

1412134673221

42

15

0000000
4115121

11111

1
0

0
1

1

3141
0000

0000
3141

2141
0000

2141

1
0

1

126131087111522415

00000000
2784415151

00000000
1114215141

0
1

0
1

1

1114215131
00000000

00000000
1114215131

1114215131

21

4
0

4
0

4
0

4

000
163

3

00
16

0
6

6

1

1621422111
000000000

1621422111
000000000

1621422111
000000000

1621422111
020000000

1421422111
000000000

1421422111

1

000000000
133231623

000000000
133231623

000000000
133231623

133231623
000000000

000000000
133231623

4

33231223

1

1

67671211114

299263255286289326467439499381354314
010010000000

1
0

0
1

1

394351411212
000000000

394351411212

1
0

0
1

1

1
0

1

21

010000000000
299254246280285321453425478380354311

00
11

1

1

000000000000
299253246280285321453424477380354311

7759786364706786886693154

372594113026322

63452124574

1

213184162207210246373304357304255155

000
111

111

0
3

0
3

3

6495886676086479066016137371403965445
032424127110

5094435164955278114264103611279873352
000000000000

21
00

21
00

21
00

21

000000101000
5094435164955258104264103611279873352

31

000000000000
120313020352641464111210727

117293017352637453510510327
000000000000

117293017352637453510510327

32341674
00000000

131211

3131463

000000000000
3894094864754907833843643191167766325

3614044854694867813713402901013704320
000000000000

3614044854694867813713402901013704320

000000000000
2851642132429154625

1121221

11

2751422122427152623

322

211

3112
0000

0000
3112

2100
3112

112
000

112

2
0

0
2

0
2

0
2

2

252642281714273061201119
000001022000

1

000000000000
21202422131222253912715

000000000000
21192321111222253912715

000000000000
20182220101220233711614

1

94723471

1

1

1

20818129616192911612

13

11

000000
111211

111211

00
11

1

1

111
000

111

0
1

1

0
1

1

0000
1112

112
000

112

1
0

1

000
111

111

000000
222111

222111

1

121713
000000

121713
000000

12
00

12

1
0

1

0
1

1

1153
0000

1

113

1

3

0000000000
2123111442

2123111442
0000000000

2123111442
0000000000

2123111442

1

00
11

0
1

1

1
0

1

18114211
00000000

0000000
1811321

1811321
0000000

1811321

11
00

00
11

1

1

1

21

0000
1312

1312

1211
0000

1211
0000

1211
0000

0100
1111

111

0
1

1

310131255133464326
000000000000

000000000000
310131255133464326

000000000000
310131255133464326

10010111717003
310131255133464326

116181

2

2102553121737322

1
0

0
1

0
1

0
1

1

3877706466638895186594137
1957505317201

000000040000
81568144913138516

111
000

111
000

111

000000000000
875613388107515

72251011312414
000000000000

7215101122414

1

1

1

1
0

1

000000000000
152132749511

152132749511

1
0

1

1
0

0
1

1

0
1

1

000
111

000
111

1

1

1

51112
00000

00
11

1

1

1
0

1

3
0

3

00
11

11

0
1

1

0
1

1

2

000100001000
213645373440302940402815

1
0

0
1

1

5102018111935151592
000000000000

5102018111935151592
000000000000

491918101935141392

11

1112

112625152321252319241713
000000000000

000000000000
274264366246

274264366246

9181813161722161122137
000000000000

1

11

1

11

11

918181215172115921135

1

11
00

1

1

000
312

312

0
1

0
1

1

3222
0000

000
321

321

21
00

21

1
0

0
1

1

0000
1141

00
11

1

1

1

00
31

31

00000000000
13331837112

13331837112
00000000100

1
0

1

1
0

1

1332183612
0000000000

1332183612

00
13

00
13

13

2111
0000

0000
1111

0000
1111

1111

0
1

1

512128717344410732

1
0

1

2121231323
0000000000

1000000000
2121231323

21221123
00000000

21221123

1112
0000

1

1

1

11

0
1

0
1

1
0

1

010000000000
7319244297443955403729

1112
0000

00
11

00
11

11

12
00

1

00
11

11

00000
14237

14237
04036

0
1

1

111
000

1

1

1

000
131

12
00

00
12

1

1

1

0
1

0
1

1

0
1

1
0

1

717174275423448403729
000000000000

000000000000
717174275413448403729

714164275413447403729
717174275413447403729

21

1

0
1

1

1

121
000

12

0
1

1
0

1

00
61

00
51

5

1
0

1

1
0

0
1

1

241774101219202217107
000000000000

241774101219202217107
000010100000

0
1

1

0
1

1

1111
0000

1111

0
1

1

2
0

2

000
111

111

2417649101618191697
000000000000

2417649101618191697

2

311291290320363346375369431316317225
000000000100

10011290877510113611221710810364
000000000000

10011289877510113611221710810364
000000000000

0
1

1

21181717633162528233722
10011089867510113611221710810364

1233417623231

743215251431856

11312

6782606061588459102605632

22

2132591

214221842

21112112

2

1
0

0
1

1

1
0

0
1

1

000010
212311

1
0

1
0

1
0

1

112
000

112

11
00

00
11

11
00

11

11
00

11

211176198232287244238254206204213161
000000200010

12
00

12

1
0

1
0

0
1

1

00
11

00
11

11

1433575162432
1333365111822

00000
21461

00
21

21

216
000

26

1

1
0

1

1

11
00

00
11

1

1

0
1

1
0

1

362027184127245521444233
000110000000

0
1

0
1

1

121
000

121

362026144027245521444133
000000000000

362026144027245521444133
001200110000

22362571451542

34182263822164016293731

0000
1111

11
00

00
11

11

11
00

1
0

1

0
1

1

211
000

211
001

00
11

11

0
1

1

174150165211241208207181158158167125
100000000000

000000000000
783558255593

783558255593
020000000000

753558255593

1

166142162206236200205176153153158122
000000000000

161140158206235199197173153148157119
000001000000

161140158206235198197172151148157119

12

0000000000
5241183513

5241183513

11111152
00000000

11111152

111

3

2

1111
0000

0000
1111

1111
0000

11
00

00
11

11

00
11

1
0

1

1
0

1

00000000000
13311111111

13311111111

1

00
11

11
00

11

00
11

1

0
1

1
0

1
0

1

116141376121226229

003100014000
2021181830216213177201310

1

201913142519591256019139
000000000000

1410326610200
201913142519591256019139

18135101713501144416125
000000020000

11

22

111141442

1712391312431083916125

1

121
000

121

00
11

11

000
111

111

11734325413
00000000000

11734325413

0000000000
22351351311

222132711
111011401

111
000

111

11213

3336
0010

23
00

23

000
312

312

21
00

21

21211366
00000010

0000000
2121254

00000
11132

0000
1112

1112
0000

1112

0
1

0
1

1

0
1

0
1

1

1

211122
000000

211
000

211
200

11

0000
1112

1

0000
1111

1111

00
12

0
2

2
0

0
2

2

0
1

0
1

0
1

1

1
0

0
1

1
0

1

000000000000
777522331216651

2

111137721
001000000

1121
0000

12

01
11

0
1

1

1127511
0000000

0000000
1127511

1127511
0000000

1127511

00000000000
677412257441

00000000000
677412257441

01010000000
677312256441

11
00

1

1

67632255431
0000000000

67632255431

00
11

11
00

1

1

1

000000
221462

000
111

000
111

111
001

11

00000
21461

00000
21461

2123
0110

2

21

1

231
000

231

3321655

3

000000000000
33887571326222

1

127844582311
127743582311

1
0

1

1
0

1

0000000000
2113125321

2113125321
0000000000

1

010000000
211225321

21224321

1

1
0

1

0000000000
6515516121111

6515516121111
0000000000

6515516121111

9242341

000000000000
10819101517132111311921

00000
21221

1221
0000

1221
0000

221
000

221

1
0

1

2
0

0
2

0
2

2

000000000000
1081710151712199301921

000000000000
1081710151712199301921

1081710151712199301921
000000000000

000000000000
1081710151712199301921

1081710151712199301921

11426046425875110189363030
033133236001

310735854115
0000000000

0000000090
310735854115

11
00

11

111

1973585324
0000010000

1873575324

1

1
0

0
1

1

110710513102026113
11364638365065102179361924

0
2

2
0

1

1

4256711821225135
000000000000

4256611821025135
000000000000

4256611821025135

12
00

12

216221882
000000000

000000
621154

621154

121
000

121

0000
1132

1122

1

00
11

11

00000000000
3513691415313

22366711
00000000

22366711

13133388211
00000000000

13133388211

1
0

1

2721343
0000000

0000000
2721343

2721343

0
3

3
0

3

00000000000
16515412172345

41
00

41

00000000000
16515412132145

16515412122145

1

1
0

1

000000000000
51013141213253788343

000000001000
51013141213253788343

3768115202452331

2376185133512

0
1

0
1

1

11
00

11
00

11

11

11

2678101999680127213356212828
41215910151819241107

000000
434121

000000
434121

434121
000000

433121

1

0
1

0
1

1
0

1

121025571217112
000000000000

000
121

121
000

1

1

1

1

1
0

1
0

1

11
00

00
11

11

2101247121712
0000000000

211343
210322

11

11

9123481412
000000000

9123481412

1

11
00

00
11

00
11

1

1

123230339210
133135364032539618911116

1224232724254679156793
000000000000

000000000000
1224232724254679156793

1218232724254679156793

6

1

371952414212

1432255

1221451
0000000

1221451
0000000

1221451

0000000100
284833719202

0000000000
1411112951

0000000000
1411112951

1411112951

0
1

1
0

1

14271258141
0000000000

00000000
112226121

111226121

1

00000
71112

2

7111

1
0

1

32

111
000

111
000

111

143221

159186281182

1
0

1
0

1

000000000000
3111110179162755633

0
1

1
0

1

000
113

113
000

113

11
00

00
11

1

1

000000002000
391110179152255633

0
1

1

000010
111151

1

1

1

114

29109179142247533
000000002000

1

1

1121

1

22214151

1

2

122515442

116

123244181112

2423124141

1

1

11

1

11

1111

2

245336141719221
000000000000

00000
11111

000
111

111

00
11

1

1

000000000000
234335141718121

234335141718121
000000000000

234335141718121

1

0000000000
33472261413

33472261413
0000000000

0000000000
33362121412

33362121412

00000
11111

11111

0
1

1

2
0

2

112181

000
111

111

1
0

0
1

1
0

1

12

000000000000
18102115181142255236408

112

18102115181142245134408
000000000000

3
0

0
3

3

18101815181142245134408
000000000000

11
00

11

17101715181142245134408
000000000000

17101715181142245134408

0000000000
14138335312

14138335312
0000000000

0000000000
14138335312

0
1

1
0

1

0000000000
14137335312

0
1

1

00000000
413723531

413723531

00
12

12

1432241431
0000000000

1412231131
0000000000

301
311

0
1

1

111222131
000000000

000000000
111222131

111222131

213

11114141

0
3

3

9123381843125468513
000000000000

000000000000
76186164241935757

1
0

1

75174154231935657
000020000000

75174134231935657
000000020000

1122231

1

1

1

1

1

2

622918210457

5131312822

1

3

12111
00000

000
111

1

1

1

00
11

1

1

1
0

1

00000
12111

0000
1111

1
0

0
1

1

111
000

000
111

111

00
11

11
00

1

1

141512751016
0000000000

141512751016
0000000000

0000
1311

31

11

141412441015

000100
161221

0
5

0
5

0
5

5

1111
0000

1010
1111

1
0

1

1
0

1

11
00

1
0

1
0

1

0
1

1
0

1

1

113222

8
0

0
8

0
8

8

231019621413112
000000000000

231019621413112

1
0

0
1

1
0

1

127352248231
000000001000

000000000000
117132236221

000000
121112

121112
000000

121112
000000

1

111112

14111226
00000000

0
1

1
0

1

13111226
00000000

13111226
00000000

1

1223

111113

111
000

1
0

1
0

1

00
11

11
00

11

1

1

000000
122111

122111
000000

000000
122111

122111
000000

122111

31451421

2274323516132

2

207292424027815217342630525600312304020459555254562371223597
347589744564132190274482841

16752714381513222110
000000000000

000000000000
16752711371513212110

000000000000
16752711371513212110

16752711371513212110
100003110000

1575277361413212110

1

3
0

0
3

0
3

3

11
00

11
00

00
11

11

142341
90000

00
42

0
4

4
0

4

0
1

1
0

1

0
1

1
0

1

0000
1111

11
00

11
00

11

00
11

11

1
0

0
1

0
1

1

21
00

21
00

00
21

21

0
1

0
1

1
0

1

0000
2112

2000
2112

1
0

1

000
111

10
11

1

1
0

1

01102331016920
318394393347608444250298338388207542

24223712821
00000000000

221211132
000000001

1
0

1

1
0

1

11
00

11

0001000
1111113

11113

1

1

00000000
21216151

21216151
00000000

1

2121651

13725824521537530917518921411377393
000000000000

000
111

000
111

111

000000000000
15637572435321

221211663
000000000

221211663

1222136162621
00000000000

1222136162621

1
0

1

124113
000000

1113

114

712091941883202584550592526355
13525323921236830416816517810975392

0
9

9

1
0

1

0000
2133

1

132

11

00
21

21

624243204142120101101744933
000000000000

624243204142120101101744933

1
0

1

000
111

11

1

11461391711
0000000000

11461391711

1791311451302281257197100266126148
000000000000

000000030400
1791311451302281257197100266126148

11
00

11

003010312421
2870949913173343033332220

1

2769899712570292726231718

3121

1122311

11112521

9243341282
000000000

9242341282

1

102
112

1

000000000
1311122102

1311122102

1
0

1

140594729954733586120694125
011000100200

4221123

1

6235538541555

12112

10

11

6

13

2

3

6142112416882

123364123864118324715985116

11

0
1

1

2111
0000

00
21

00
21

21
00

21

11
00

11

000000000000
654021192827374540263946

0
1

0
1

1
0

1

0000
1232

1232
0000

0001
1231

1

1

1

21

1
0

1

010000000000
623516172826323933233746

11
00

11
00

1

1

39357134688
00000000000

11
00

1

1

00000000000
18357134686

18357134686

0
1

1

11
00

1

1

211413412
000000000

000100000
211413412

1

211313411

0
1

0
1

1

0000
1481

0000
1481

1481

12
00

0
1

1

0
2

2

552313141918263317152836
000000000000

00
21

21

1

000000001000
552113141918263315142836

552113141918263314142836

0
1

1

13225121
00000000

00000000
13225121

1
0

1

322512
000000

322512

0
1

1

000
211

211
000

21

0
1

1

00
11

11
00

11
00

1

1

32
00

32
00

00
31

31

1
0

1

11

1212
0000

11
00

00
11

00
11

11

0000
1111

1111
0000

1111

000000000000
412425203515371535213618

2
0

0
1

1
0

1

0
1

1
0

1

00
11

11
00

00
11

11

1
0

1
0

1
0

1

412324162914351531203517
000000000000

412324162914351531203517
000000000000

412324162914351531193517
000000000000

412324162914351531193517

0
1

1

0
1

1
0

1
0

1

111111
000000

111111
000000

000000
111111

111111

115
010

15
00

00
15

1

5

00
21

00
21

00
21

21

000000000000
147144681013215725

147144681013215725
000000000000

137144481013215725
000000030000

31124712
0000000

31124712

123124365395625

11111
00000

11111

12
00

01
12

11

111

2
0

2
0

2
0

0
2

2

000000000000
214417711112337751

213407710102037751
000000010000

00
11

11

000000000000
213397710101937651

000000000000
213386710101836551

213386710101836551

1
0

1

1
0

1

111
110

1

11113
00000

11113
00000

11012
11113

11

4323344678116
000000000000

000000000000
4323344678116

000000000000
4323344678116

000000000000
4323344678116

4323344678116

202242365127215212512555225007306903957358760249052334522896
122725322791229124493235182324433009147413011864

0
2

2
0

0
2

2

001000000110
5565156345396126027828521000680614381

0
1

1
0

1

000000000000
263288331343312402330327448310300168

000000000000
263288331343312402330327448310300168

263288331343312402330327448310300168

5969167944322
000000000000

4869167934322
0457164312200

1

44121612122

2

000
111

111

1
0

0
1

1

121
000

11

2
0

2

107889246111591412621921309575
000000020000

0
1

1

107889246111591412601921299575
1210206101026503515104

2237311039257413578391725

141312112819183936171916

11

1

1

1

51164337413151

108444465932

4418249262152234353027

7464612121220136
00000000000

000
121

121

7463610121120136
00000000000

7463610121120136

11111312
00000000

00000000
11111312

11111312

11
00

11
10

1

169122194137165130285246339208201120
100000001300

616326121519694
000000000000

616326121519694

0000
1111

1111

59446537605614688161736438
000000000000

21

59426537605614687161736438

1
0

1

000000000000
555795767954809399828857

555795767954809399828857

131
000

131

11
00

11

0
1

1

1
0

1

000000000000
168131113522162713189

168131113522162713189

958654913151226
000000000000

958654913151226

227744512211518193
000000000000

227744512211518193

1112
0000

12
00

12

1

1
0

1

11020112
13232424

2212312
0000000

2212312

000
131

0
3

3

11
00

11

1
0

1
0

1
0

1

212427242426382133453117
000000000000

0
3

3
0

3

17151618181728815442916
000000000000

000000000000
17151618181728815442916

1

17151618181728814442916

000000000
810347710171

810347710171
000000000

810347710171

113213111
000000000

0000000
1122311

1122311

000
111

111

22
00

11
00

11

11
00

11

00
11

00
11

11

1
0

0
1

0
1

1

5211222012250
276759597892572360197863133262029835990401729584425

1
0

0
1

1

272187174132148102278292324342278221
000000001000

271182173130146101273289321341277218
000000000000

271182173130146101273289321341277218

0
1

1

1
0

1

00000000000
15122152213

15122152213

111
000

000
111

111

74471172643509371113967846
248857697704558958687758130482000235664367126724204

832113573142
000000000000

832113573142

283169201936131924333137
206113219139148762284328385232213224

1

11

1221

473554412845233346293696

3354317117563

20

13

1111234135

2

91912810146214103

1184259608867021725730114912679

1

0
1

1

0
1

1

0
2

2

1
0

1

00
71

71

1
0

1

1

0
1

1

1
0

1

3
0

3

453551364136434885555738
312123402841

403143293531313357385233

23654281526914

1

19917264211917131010
000000000000

19917264211917131010

1

1
0

1

1
0

1

1
0

1

000000000000
545452418235379284541675605697608323

545452418235379284541675605697608323

0
1

1

2102015302023363
00000000000

11

2102015302022263

1
0

1

76251252348732
000000000000

76251252348732

11
00

1

1

170165146353158117174215204236181207
000000000000

170165146353158117174215204236181207

0000
2111

2111

00000000000
10992424261114

10992424261114

1
0

1

0
1

1

0000000000
2161112431

2161112431

1
0

1

0
3

3

12526932375692176254291714
002000000000

12526732375692176253291714

1

162321211647122619272318
000000000000

13222751

152019191447121914272218

2216142012317923532020
000000000000

2216142012317923532020

00000000000
61721314743

1

51721314743

00000000000
21133122951

21113122841

1

111

103546126374451845686162113491796733376196311592980
000000201000

103546126374451845686162113471796733375196311592980

1
0

1

000000000000
7374434782912

7374434782912

1
0

1

343534205326384723344045
011101002100

273230174922323619273445

623243611266

1

1
0

1

1
0

1

0
1

1

0
1

1

1

7553127103131139
211231222122

4431746111913

1122214

257162162151339166330383509177222229
863840108234847988639982

1611029911824211621724333290108141

3111171110193350842

1

49101346925361063

32133235

1113

11
00

1

1

1

000000
121313

1
0

1

1
0

1

0
1

1

11
00

11

11112
10011

1

1

1

210020100710
449279378244333297508572643677470346

201211128210
372250317203276274469519583565391282

11
00

11

2215921813313735374024
10952704169931209811218311888

4442410561454

1111111

7134483244706847511125752

1

11293561081919157

16711415495149131271302360216175125
672441303628342544866754

151256377722

1

54631081220866

131617911268141215812

1

1

112222611324

1

1

1

23

11

755258307953197210221928444

1111

51015191181224401043

1

1321

0
1

1

1
0

1

6049755337384575561247348
251618202322111416421220

353321333253

322854301215315837805625

010001000200
343517121811324147392421

1

343216121710324147372421

21

7528614155233853601057864
15118121214101121157

592342273920293744765750
30797155468222320

2091791612182419462326

31

21111

7415117376167114

000000000000
141242565867

131242565867

1

000000000000
216147143153181138288300330224208105

13121014341321261620177
000000000000

000000000000
13121014341321261620177

1

13121014341321261619177

20313513313914712526527431420419198
3423155551164

14333017121738508228157
000000000001

14333017121738508228156

1
0

1

1
0

1

1869810111913410222221922716517086
000000000000

1869810111913410222221922716517086

0
2

0
2

2

524184253136150292334398411600479254
205011000000

402961076776203185217265466379125
341220344130

0
1

1

000000000000
37870955156183156179212447351114

37870955156183156179212447351114

1

212011141817263343182411
310021304210

186547132123283

17115912910121614158

1161
0000

1161

00
11

11

7943814339577411479865584
000003000001

0
1

1

11

313
000

313

64143431210558
7941814039547411279865480

18479661810148410

1

181417661322421835177

371955212431314837382855

0
1

1

414560263431756767484545
000000010010

0
1

1

1
0

1

152843142119211919292629
000000000000

152843142119211919292629

261617121312544648191816
000000000000

261617121312544648191816

198716991878146920431499173318662072221221991797
000000000000

198716991878146920431499173318662072221121991797
111012512111319231195

122120121911291833251816
000000000000

1

122119121911291833251816

1
0

1

1
0

1

00
11

11

000000000000
90628350738682779513310565

11

90628350737582779513310565

681621143632564055436635
275374385413542393360351353257255343

187348353393497357290291274171175288

177415331613371414

1223383433

21521131733

11
00

11

2
0

2

002000000000
544567712533623437368431495669781515

212322

1

544567710529622436366430491669779512

211

1

1
0

1

1111
0000

1111

0
1

1

000000000000
3992114295454049394619

3992114295454049394619

1
0

1

1183231
0000000

1183231

2

000000000
412644562

1

1

412444562

0
1

1

1
0

1

00
11

11

1
0

1

1332
0100

1232

3112113331
0001010200

1

1

31111211

1

1

13143
00000

13143

111
000

111

117136416797119
000000000000

117136416797119

1
0

1

11
10

1

111
000

111

1
0

1

0
2

2

1

000000000000
8323251410131244

532325141011944

323

210161142106267148136153150181168321
130807551218869096823552239

80796455475841556314511482

1

23245252

000000000000
3241131071001217292125119342297107

320113106981207289121118335296102

312341715

11

1117073448555621017910712464
167247228122164217309317381240244173

111

6272116142122324110142

625341919324818101613

3112268621

411249943441092181702341078892

0000000000
4252155951

4252155951

00
11

11

1
0

1

53866613613642
000000000000

111

53765613613641

000000
211112

211112

11
00

1

1

1
0

1

1
0

1

0
1

1

000000000000
1686560599963150178217180130142

1686560599963150178217180130142

000000000000
1175393851512114

1175393851512114

111
000

111

111
000

111

762942164829717751496450
000000000000

762942164829717751496450

000000
121751

121751

1
0

1

001000011100
1612241220811232824249

97105136671217125

7513772515156124

11
00

11

1
0

0
1

1

010000000172
45175991091269640031731934984423581338

112111
000000

112111
000000

112111

6054805349417396105128167738
000000100090

1

000000000020
69421884912123615887

69421884912123615867

22211111
00000000

22211111

0
1

1

000000000000
182513182217334130323217

182513182217334130323217

000000000000
341823161819294362594714

341823161819294362594714

113111
000000

00
11

11

00
11

11

1
0

1

111
000

111

1
0

0
1

1

1111514
0000000

0000000
1111414

1111414

0
1

1

22213
00000

21
00

11

1

1
0

1

0
1

1

0
2

2

3
0

3

11
00

00
11

11

00
11

00
11

11

1211
0000

0
1

1

0
1

1

00
21

21

0000
1111

1111
0011

1

1

3726978138516373542161962186996521284
000000000000

310010002131
50204018284611554971047539

47194018274611554951037237

1

1
0

1

000100000000
3226777738326093071011411215955771245

2123134631

315673768829603303991381145865731236

3211212

11

111

1121111316

1

00
11

11

1831345525211712185
000000000000

1

0
2

2

000001000000
1631345524201712185

1631345424201712185

1
0

1

15551885140499414111228153617721684201315071509
2824001511736224

21134111
00000000

00000000
21134111

1111111

1231

359232255189291201390426368338324192
020000021010

350222249182278197387414360289316181
000000000000

350222248182278197387414360289316181

1

9867134310749711
000000000000

9767134310749711

1

207198210160204167296286338284195150
111015611132616241777

0
1

1

1

1
0

1

00000000100
95432124331532

1

83432124331432

2

321141873332
000000000000

321141873332

000000000000
815189232412911131210

81517923249911131210

11

2

0
1

1

11121
00000

11121

00
11

11

1010751072011172154
331012545140

676595157121114

1

140128133127125102164180218191126103
453328404928504966401927

259182122542

332637271519273452442434

1

31221521

515853584844868795927332

66614752547

1
0

1

12123
00000

12123

1
0

1

100121135223
57521071081526107

152452331753

323141727731

1
0

1

00
11

11

19182561911282031271715
242031231340

1613215157241623151113

2221

11211321571

000010000000
19152211221352020141421

0
1

1

6192653108322
000000000000

6192653108322

1
0

1

10121051242599415
000000000000

10121051242599415

00000010000
32342443284

32342433284

032000120100
337932396238339332416488501668351678

000000000000
9991561514127135

9991561514127135

0
1

1

11

328920385236332326400472489660338673
000000000000

328920385236332326400472489660338673

000000000000
605506517396553513423538444670600461

211

0
1

1

000000000000
605504511395553513421534443670600459

605504511395553513421534443670600459

01000
26122

25122

100
222

000
122

122

000000000000
36681043825298066307533758

0
1

0
1

1

36681043825298066307533658
000000000000

1922
0000

1922

36681033825298057307533456
000000000000

36681033725298057307533456

1

000000000000
199108154131171132163152182194209165

0
1

1
0

1

199106150125169129160139163193209164
000000000000

000000000003
199106150125169129160139163193209164

199106150125169129160139163193209161

0
1

1
0

1

0000000000
246223121911

1
0

1

00
11

11

0
2

2

000000000
23622212171

23622212171

411431752333
311431552333

1
0

0
1

1

1
0

1
0

1

1
0

1
0

1

11111
00000

11
00

1

1
0

1

1
0

1
0

1

00
11

11
00

11

16201151141594914481253176320692595209219521263
000020000000

61318111415204476963
15061075135188513561152166319732458193818191187

020110010100
421200253163248133301332444513624248

190275820412210213015125030664

1111

231171195141205111198201293262317184

00
11

11

1
0

1

1

111335716
000000000

111335716

284130158103259175176219180259267243
000000000000

284130158103259175176219180259267243

000000
111312

111212

1

0
1

1

1
0

1

8472739410473
100000010010

111

6462739310462

2411147137151922182315
000000000000

2411147137151922182315

1131221
0000000

1131221

11
00

11

2
0

2

0
1

1

100000000000
81013678403029111211

7913678403027111111

121

510423546334503553613720829832630425
7527068835908068091099132016891110876655

221432261

504449294643148167279575121

124184230210209176265358455147124148

6653581644347175124687061

1006052458291908111713710869
000000000000

000000000000
1006052458291908111713710869

1006052458291908111713710869

000000000000
1416121981010152017257

51155251774145
000000000000

51155251774145

9571465981313112
001000010000

9561465971313112

7744625152397476110939069
000000000000

0
1

1

010
111

1

1
0

1

000
111

000
111

11

1

00000000000
12124122532

1
0

1

0
1

1

2111112
0000000

2111112

00000000
12211122

1

12112

1111

0
1

1

1
0

1

101000000140
592729302919373952545344

000000000000
411719141811122016262929

411719141811122016262929

000000
113111

113111

0
1

1

100000000000
1710915108221834271915

1610915108221834271915

171531192216353455313422
000000001000

000
111

111

1
0

1

3
0

3

1
0

1

1
0

1

141021131116292937211812
63236510521187

51116359142521

1

3483258710794

1

11

11111

2

11444349368
00100011010

1

11

11244337348

00000000010
22412213451

22412213441

21151411
00000000

21151411

10
11

1

000010000100
824656741622754621656633725874825757

2930323624285563102282316
000000001000

2830323624285563101282315
010100104000

282931352428546397282314

1

1

11
00

11

0
1

1
0

1

31225
00100

2114
0000

2114

1111
0000

1111

000000000000
522420163322444363173828

522420163322444363173828
000000000010

512420163222444362173728

111

21212223221
00000000000

0
1

1

211213221
000000000

211213221

100
121

1

1

1

494300338277349322464432465484438258
100000000000

0
1

1

493300338277348322464432465484438258
000010000000

1

493299335276347322463432465482437255

1

313

2

1

000000000000
235296338287332235797873335309452

111
000

111

000000100000
234296338287332235797772333308452

1

234296338287330235787771333308452

1

1

0
2

2

0
1

1

9511513141214165102
000000000000

000000000000
9511513141214165102

9511513141214165102

000000000000
1978890982579885491155317522010267721721894

1978890982579885491155317522009267721721894
000000000000

1
0

1

77359595015943110194121675620172
1978890982579885491155317522009267721711894

1089748800463626352134714651774190314411627

1

83791236399969288110698092

1

1

3343144329303

1
0

0
1

1

000000000
322124631

000000000
322124631

00000000
32224631

32224631

1
0

1

1891526881722532111
010010002110

1
0

0
1

1

176131478141952188
010001000010

0
1

1

1
0

1

00
11

11

12113
00000

12113

0
1

1

133912268944132
000000100000

133912258944132

1
0

1

2
0

1

1

0
1

1

0
1

1

0
1

1

112211311
000000000

112211311

0
1

1

00
11

11

0
1

1

1
0

1

229
001

1

227

00
11

1

1

1000000000
1211113123

00000
11123

1111

1

3

0
1

1

000
213

213

90208229308184169239269722937079
000000000000

11
00

11
00

1

1

000000000000
472128173331294052323618

000000000000
472128173331294052323618

1

1

472128163331294051323618

000000000
222141141

000000000
222141141

222141141

41184198291150138206229669603060
000000000000

100001032000
41184198291150138206229669603060

40184198291150137206226667603060

181143168125134181263258283221176105
000000010000

00000
11111

1

010
111

11

1
0

1

433251085510411
000000000000

1
0

1

0
1

1

00
11

11

4222597439311
000000000000

4222597439311

0
1

1

000
121

121

181011149141410821167
17413515512012717024624325919916986

21

2
0

2

999310097961211601481751229046
000000000000

999310097961211601481751229046

11
00

11

1
0

1

1111232
0000002

111122

1

1
0

1

1
0

1

0
1

1

41
00

41

1112
0000

1112

1
0

1

2
0

2

12
00

12

00000000
11133122

11133122

00
11

11

231223118451
000000000

231223118451

00
11

11

1
0

1

1
0

1

010000010100
41213871830424446384625

1

40203871829424346374524

111

0000
1112

1112

000
111

111

000000
211144

211144

812332
000000

812332

111
000

111

0
1

1
0

1

0000000000
11617812418

164510

11332418
00000000

11332415

3

00000000000
14421111671

111
000

111

111001140
143211571

1

2

3

1

243

0
1

1

2231470
000004

1213
0000

1213
0000

1213

110032
211166

002
118

5

111

0
8

7

1

110
17

3

0
1

1

5
0

5

2
0

1

1

1
0

1
0

0
1

1

000000000000
572212367612

000000000100
472211364612

00
11

11

000000000000
342211364412

11

11

34211344312

1

0
3

3

000
112

12
00

2

1

0
1

1

1
0

1
0

1

8626473345356140734160119
000000000000

000010000000
7924452837335434713658113

7924452836335434713658113
000000000000

9112978461261319

702333192925502859304594

1
0

1

722582562516
722582762516

0
2

1

1

564845243926548083635852
000000000000

1
0

1

0
1

0
1

1

1
0

1

0
2

2

11
00

1
0

1

0
1

1

554845233926537981625852
013020344101

0000
2252

2252

1
0

1

524742233724487577565549
000000000000

524642233724487577565449

11

1
0

1

112
000

1

12

000010123100
12673526372634794491380216941572212793612803188

000000000000
216210242232235201380318382307327149

4152541

7667411095564
211209241232235200375314375303325148

191923242935534138231511

111

53233491941

178181210201199161308254331266299132

1

0
1

1

0
1

1

1
0

1

1
0

1

121

1
0

1

371818152513282151342418
000000000000

371818152513282151342418
000000000000

371818152513282151342418

001000000101
1317181093221425272320

21421329124
00000000000

21421329124

1
0

1

12113213
00000000

12113213

111
000

111

000000000000
111612761161216211912

111612761161216211912

123842405325353835122531
123842405325353935142531

12
00

12

0
1

1
0

1

000000000000
163966471181471

163966471181471
000000000000

163966471181471

416
000

416
000

416

13211114312
00000000000

0
1

1

00
11

11

1121
0000

1121

1
0

1

1

0
1

1

000000
111221

111221

0
1

1

00000000001
24343126842

11
00

1

1

1
0

1

24242116841
01120100110

1

11

111221131

221

1

31

1

1

154871117692114656812747128275
000000000000

0
1

1

11
00

1

1

000000000001
153861117691114646712747128273

1

152861117691114646712747128272

00
11

1

1

00
11

11

000000000000
813314532813095406634401148108814794867442690

1
0

1

813314532813094406634401147108714794847432690
000000000000

813314532813094406634401147108714794847432690

12
00

12

11
01

1

211
000

0
1

1

0
1

1

1

0
1

1

000
111

00
11

1

1

0
1

1

00
11

11
00

11
00

11

425354365340390405573626602641572403
000000000000

424353364337390405573625594641571403
1711111421219116716518264163198132

11

0000
1111

1111

12
00

12

67110851131559085779011112458
000010000000

2

67110851131549085779010912458

000000000000
109310881416232168

109310881416232168

3611514129283901177768
000000000000

3611514129283901177768

24914213243313718785234
000000000000

1

24913213243313718775234

1

0
1

1

93931006190771641942761019290
131821141316364149191417

51345561017251095

2728432137206881122293126

114234311916946

1

373030183132394564343436

23913998223531492113
000000000000

23913998223531492113

00
11

0
1

1

1
0

1

1111
0000

0
1

1

0
1

1

1

0
1

1

217
000

217
000

217

101521
00000

101521
00000

131
000

12

11

00
81

81

1
0

1

10
11

1

0
1

1

1
0

1

000000000000
10921824620219416371100113196107257

10921824620219416371100113196107257
200200012110

00
11

11

0
1

1

00
11

11

122
020

12

0
1

1

1
0

1

9319321817818815555717218193247
000000000000

9319321817818815555717218193247

000000000000
112428215714283812139

112428215714283812139

0
1

1

312423
000000

000000
312423

2112
0000

2112

11
00

1

1

000
211

211

00
12

12

000000000000
139205817101131567

10718561061011456
000000000000

10718561061011456
000000000000

10718561061011456

21
00

20
21

1

1
0

1
0

1

00100
11311

0
1

1

00
11

11

101
111

1

0000
1112

1112
0000

1

111

1

112311
001000

0
1

1

1
0

1

0
1

1

000
131

131

0
1

1
0

1

1112
0000

0000
1112

1112
0000

1112

204144160123258143188228198179159167
000000000000

101100
711413

1
0

1

3

0
1

1

1

1
0

1

2
0

2

212
000

212

2
0

0
2

2

000000010000
136103109102215117128147145114109123

000000000000
136103109102215117128146144114109123

136103109102215117128146144114109123

1

1
0

1

4000302101702
614050214326608051614941

1
0

1

000000000000
352327142214383720292418

352327142214383720292418

1915126128141920191817
000000000000

1

3642311351126

168849713161481611

1

321016461410674
000000000000

121621

3210152588664

0000
2251

2251
0000

2251

111312
000000

111
000

0
1

1

0
1

1

1
0

1

00000
11121

00000
11121

11121

000000000000
35405125352810788155845623

2727332027229069142784314
000000000000

1

1
0

1

0
3

3

11
10

1

00
13

13

1

0
2

2

0
1

1

0
1

1

2626321727228864142733814
142014121410764912451228

44131495971084

82549376111282

1
0

1

141
000

141

0000000000
172214148363

1111552
1112672

1

111

00
11

11

0000000
62111736

62111736

11

000000001010
71311366355376

0
1

1

0
1

1

0
1

1

0
1

1

1
0

1

000000000000
6211233111261

1

1

511231241

10

111112

000
111

111

0
1

1

0
1

1

2
0

2

1022224

9515219789116976410514611178159
000000000000

0
1

1
0

1

0000010
341141511

0
1

1

0000000
341141311

341141311

1
0

0
1

1

21111
10000

0000
1111

1

11

1

1
0

1

0
1

1
0

1

0
1

0
1

1

000000000000
931491948511395588713411078159

000000000000
931491948511395588713411078159

931491948511395588713411078159

0
1

0
1

1

0000
1111

0000
1111

1111

1321
0000

1321
0000

0
3

3

0
1

1

11
00

1

1

1
0

1

1601747872730111885970178087716071590768
000000010000

1211
0101

0
1

1

11
00

1

1

2
0

2
0

1

1

1101101
1122311

1

221
000

221

0
1

1
0

1

141000112031
1601747871728111685969977787516041586767

1810841245332928355
000000000000

1810841245332928355

1

32
00

1

21

1

1
0

1

349248264355454386154166165363337199
010000000100

1

349247264355454386154166165362337198

132
000

132

0
1

1

1

0
1

1

000000000010
11782927012092112122141121141116

11782927012092112122141121140116

107838146428150436335439746810671052396
000000000000

107838146428150436335439746810671052396

831781241538501157
000000000000

831781241538501157

1

000000000
432353322

432353322

111111
000100

1

1111

1
0

1

0
1

1

1
0

1

00
11

11

000000000000
2218201014111011137939

2218201014111011137939

1
0

1

0
1

1

11
00

11

0
1

1

000
111

111

11
00

11

18468754311639439071151193198
000000000000

000000000000
18468754311639439071151193198

18468754311639439071151193198
000000000000

18468754311639439071151193198

000000000000
93964876056973563711351100118110821026648

93964376056873563711351100118010821026648
363546143430302729283223

000
312

312

1
0

1

57134118351710093109453620
000000000000

57134118351710093109453620

2
0

1

1

000000000000
4387642643514

1

4387642633514

000000000000
304156252632314043346859

304156252632314043346859

151162141146186150115153147194179148
1134451141912201714

12015212613717113178114107157152125

75212781012523

132948111510161286

425250120601
660389465357448404857781848776704384

1

643366443335369398846763839760686379

1

3

13211720716101689184

11
00

11

0
5

0
5

5

0000000
1114221

1114221
0000000

0000000
1114221

1114221

000000000000
54564834595487851331109438

000000000000
54564834595487851331109438

000000000000
54564834595487851331099438

54564834595487851331099438

1
0

1

000000000001
63394126473976107105915733

2112521
0000000

2111521
0000000

2111521

0
1

1

4
0

0
4

4

001000100000
1242241021142

00
11

11

11
01

1

11
00

1

1

0000000000
2321481112

2321481112

00
11

11

12
00

12
00

12

000000020000
6235362345356010299894929

1
0

1

000000000000
613436234534599981884929

613436234534599981884929

1

00000
111118

1117

11

1

000
111

1
0

1

00
11

1

1

1
0

0
1

1
0

1

0
1

1
0

0
1

1

001000010101
298294367228310154315398388438318315

3426399919354950825523
000000000000

1
0

1

0
1

1

1
0

1

3326399919354950785423
000000000000

3326398919354950785423

1

3

000000000000
264268327219301135280347338355263291

264268327219301135280347338355263291
000000100000

264268327219301135278347338355263291

1

0
1

0
1

1

00000000
11111112

1

1111112
0000000

1
0

1

000000
111112

111112

0000
1114

0001
1114

112
000

112

10
11

1

11
00

11
00

00
11

00
11

1

1

224221
000000

224221
000000

224221
000000

000000
224221

224221

14232329
00000000

00
11

11
00

11
00

11

4232229
0000000

0000000
4232229

4232229

00000000000
1037588161223129

1037588161223129
00000000000

0
2

2
0

2

00000000000
937368111118129

00000000000
937368111118129

937368111118129

1112
0000

1112
0000

1112

11511
00000

11511
00000

11511

1

4

2

12
00

12
00

12
00

10
12

2

1

00000000
32247582

2

12247582
00000000

111112
000000

111112

113658
000000

1

113657

21014221425152132556
035254638100

691861161015122
00000000000

2
0

2
0

1

1

428222341
000000000

428222341

271049451122
0000000000

0
1

1

271049441122
0000000000

271049441122

1162752

00000000000
21114114332

21114114332
00000000000

00000000000
21114114332

21114114332

0
1

0
1

1
0

1

1365236161
000000000

1365236161
000000000

000000000
1365236161

1365236161
000000000

1365236161

143010072100
133315111467157115071501149220431980169115061010

111
000

000
111

111
000

111
000

111

1

187542459745484667544677186210248
000000000000

122581
000000

00
11

11
00

11

000000
112571

112571
000000

112571

000000000000
187541457745482667524169186210247

000000000000
187541457745482667524169186210247

187541457745482667524169186210247
000000000000

187541457745482667524169186210247

00
11

0
1

1
0

1

1
0

1

99111616881814154351437538
84774471759576666010771508129912331021547

3
0

0
3

3
0

3

276180187139131181276241363547397124
000100205001

11122111
00000000

00
11

11

000000
111211

111211

1
0

1

0000000
2423142

0000000
2423142

2423142

8381924135603837551277750
000000000000

0
1

1

8381924135603737551277750
000000000000

8381924135603737551277750

10146111662519171534
000000000000

2
0

2

000
123

123

0
2

2

000000000000
9126111662317141534

9126111662317141534

000
121

121
000

121

1

11
00

00
11

11

000000000000
1838183848011120817828040431568

3

1

1838183848011120717828040131568
000000000000

1838183848011120717828040131568

0
1

0
1

1

829469422182622912
000000000000

111418133745
00000000000

00000000000
111418133745

111418133745

00
11

11
00

11

0
1

1
0

1

1
0

1
0

1

7192553135201557
000000000000

7192553135201557
000000000000

7192553135201557

111
000

000
111

111

0
8

0
8

8

0
1

1
0

1
0

1

72730248527357623
00000000000

72730248527357623
00000000000

72730248527357623
00000000000

72730248527357623

0
1

1
0

1

710020351628790
4563944313514533627211175828520530347

11211
00000

1121
0000

1121

1

000001011201
3653453453123763166061082706404423274

7498819711074989113613014771
000000000000

7498819711074989113613014771

0
1

1

000000000000
291246264215266241508990569272276202

291246264215266241508990569272276202

000000008200
91189155151928311514

66361111013139119
321350424333

1213543621

22133117665

000000000000
3553445672045

3553445672045

1
0

1
0

1

00
11

11
00

11

000000000000
753676305841645765778359

26192113213112027352517
753676305841645765778359

2121338252

441546143637473325365340

3172113154

34755141539111
00011200001

3474412153711
0000000000

0000000000
3474412153711

3474412153711

1
0

1
0

1

1
0

0
1

1

112
000

111
000

000
111

111

0
1

1

011167102152349427
9356751322676123213191818

11

62140351922528113311138
510171697162835334

1341212
0000000

0010000
1341212

1

12

1

1

121

1

1

1

1210839262536321
000000000000

001000000000
1210839262536321

1

129739262536321

51214211141
0000002000

1
0

1

00
11

11

3112521
0000100

1

11

1

11

111

1

1

111

2112312
0010100

11

11111

111

00000
12131

0
1

1

11131
00000

11

1112

1111310
000000

000
111

111

00000
11128

11

11116

1

1
0

1

11
00

11
00

11

151233152541
0000000000

151233152541
0000000100

111

1

11

11

1

131123142011

3

11
00

00
11

1

1

0000000
43125102

43125102
0000000

43125102

11321
00000

00000
11321

11110
11321

1

1

11

33793181626432

112
000

112

0
1

1

287183213171222143277352374251257192
0112115618101

412913412
00000000

412913412
00000000

412913412

712131710818466317147
000300310101

0000000
412261623

412261623
0000111

412151522

1

000000000000
63456234916105

63456234916105
000000000000

63456234916105

1826114192241
00000000000

1826114192241
00000000000

1

1826114192141

1112
0000

1
0

1

11
00

11

11
00

1

1

1111
0000

0000
1111

11

1

1

212037
213138

00
11

11

1

000000000000
13448461922113

1344646161611
00000000000

00000000000
1344636161611

1344636161611

1
0

1

23612

11
00

11
00

00
11

11

275167195148202128239268266230242179
0260302411011

1
0

1
0

1

271219232715555325202424
000000000000

000000000000
271219232715555325202424

271216232715555325202424

3

0
1

1
0

1

1231081139913294146136160180154120
000000000000

000000000000
1231081139913294146136160180154120

1231081139913294146136160180154120

0000
1212

00
11

11

22
00

22

00000
64114

64114
00000

1

6414

000000000000
1243452233918367567306330

000000000000
1243452233918367567306330

1243452233918367567306330

00
11

11
00

11

1
0

1

0
2

2
0

2

00000000000
227925861415

227925861415
00000000000

227925861415

335332122211931614213
1421321310006

12430171616152245327

000000000
313222641

2211411

1

1121123

14

1112
0000

1112

9334749352214521240318936

13492717967666894104148110103
000000111000

00
11

00
11

11
00

1
0

1

1
0

1

020010310010
11457373749463866581028267

13
00

00
13

13
00

12

1

000000000000
524643127639

1

000000
121313

000000
121313

1

1111

12

1

1

1

3333122126
0000000000

0000000000
3333122126

3333122126

000
111

111
000

111

0
1

0
1

1

00000
11331

00000
11331

11331

1
0

1

0
1

0
1

1
0

1

0
1

0
1

1
0

1

00
22

00
22

22
00

12

1

21221
00000

21221
00000

0
1

1

12
00

1

2

112

100
116

6

0
1

1
0

1

00
14

2
0

0
2

2

00
12

00
12

12

0
1

1
0

1
0

1

00
12

00
12

0
1

1

1
0

1

1

1
0

0
1

1
0

1

000000001000
1094732294143325742877857

0
1

0
1

1

1094732294143305538877856
000000000000

1
0

1

113
103

1

000000000000
954431284042295135786952

944431284042295135786951

1

1

00
12

12

1

1
0

1

0
1

1

141794
00000

141794

11
00

11

0
2

2

2221
1000

1
0

1

1

00
12

2

1

0
1

1

12
00

00
12

0
1

1

1
0

1

1
0

1

1
0

0
1

1
0

0
1

1

000000000000
203434411719282744462836

2137178912723515
000000000000

2137178912723515
000400006000

04312034401
111464534741

1

17151534

0
1

1

12
00

1

1

1

0000000000
1225235371

1

1215235371

221234
000000

221234

000000000000
18212622910151921402731

18212622910151921402731
000000000000

000000000000
18212622910151921402731

1815161564141513402627

61073614814

00000
12111

00000
12111

1
0

1

1211
0000

1211

000
111

000
111

111
000

1
0

1

11
00

11

1

086460188211
23455041540639035872311881887235207178

83548402834407411710411

218188626067172338604341330
611171411103891152436

06253381033241
8515638284010919136220816

02210721114000
837492415277313224615110

00000000000
351344921397984

351344921397984

0000000
24571712

23571712

1

0000000000
534675153422

534675153422

01
11

1

11339
00000

11339

111
000

1

11

521261543365792412
000000000000

521261543365792412

8591010284983335

71915102117255690928

0
1

1

8725771394210218330814924
000000000000

8725771394210218330814924
000000000000

869506236379517528412921
869506136379517528412920

11
00

11

37935782423
0000000000

37935782423
0000000000

37935782423

1968133265182115

196345210221244212382534768174179107
03810211516205085316

14727108727161618187
000000000000

1457106424131113147
000000000000

1457106424131113147
000000010100

1457106424121112147

672333554
10000000

0
1

1

66111
0000

66111

1222554
0000000

1222554

000000100000
7118101514154076735

213441011

00000000000
72212223631

1113

712212631

967129833663
000000000

43365317362

53464516301

16736214204462
115947217255262

1
0

1

31243
00000

31243
00000

31243

11114
00000

11114
00100

1

113

1

000
111

11

1

0
2

0
2

2

2

000000000000
546312423164577104744

2

531101412827394863
033223371211

312246564

5155102115252712
073600106301

42111146

1

11

151314151711

1016323
000000

25323

811

11356

895545152712

0000000000
71126410182321

21
00

21

1

0000000000
5112639172321

5112639172321

0
1

1

1101213455111

000000000
41354182845

1111237

2

3124462536
00000120

00000000
2113321713

2113321713

11113623
0000000

11113623

11211
10211

1

130115989113411519219921510512564
070100260000

111

00000000000
1642131091811

00000000000
1642131091811

1642131091811

12999887712510417016715710112056
000000000000

12999887712510417016715710112056

14
00

0
3

3

11

21343381122

1
0

1
0

1

00000000000
157446923335

154425723324
111324415114

1

43122

1

1

52

1

321211

84121734252
00000000000

84121734252
00000000000

00000000000
84121734252

84121733252

1

1
0

0
1

0
1

1

0000000000
48101414931

48101414931

11

000000000000
2620233032364985144222215

000000000000
2620233032364985144222215

2620233032364985144222215
034010003000

2528121422222219191912

113111816142663119323

21131

0
9

9

011010011100
5923737363742144386317277483016923384863099

000000
121151

121151
000000

000000
121151

121151

21819291118192149515
000000000000

21819291118192149515
001311742100

21614167109732311
000000001000

0
1

1

0000
2114

2114

000000000000
2151215697422311

1

432132292

186104221311

11414

32312151

000
113

113

000
111

111

00
12

12

22727281301
2410373101514

1
0

1

1
0

1

22111213
00000000

1111

1211112

484325233933337418730024834066242214272624
000000000000

484325233933337418730024834066242214272624
000000000000

000002000000
484325233933337418730024834066242214272624

1
0

1

482323433733320417129914643815832064252621
000000000000

482323433733320417129914643815832064252621

00000000
21222121

21222121

211265
211165

1

9109127613189

0000000010
67542861762

57542761652

111

0000000000
154334715221

154334715221
0000000000

0000000000
154334715221

154334715221

0000000000
262325101331

262325101331
0000000000

141231
000000

000000
141231

141231

00
11

00
11

11

121
000

000
121

121

0000
1111

1111
0000

1111

00000000
221261011

221261011

000000000000
92440203026541081759310

92440203026541081759310
031220525100

3913111012234078323

021205270111
24142931281623

1

1
0

1

00
13

13

00000000000
212241551312

212241551312

48125911145876314
24433492225200

0003300
312131814

21181514

1

12

135255523291
0000000000

135255523291

14
00

14

0
1

1

113
000

113
000

000
113

0
1

1

112
110

2

964321698191461152002537999855459
000000000000

000000000000
964321698191461152002537999855459

964321698191461152002537999855459
000010000000

000000000000
954321698181451151992537989855455

954321698181451151992537989855455

11114
01000

1114

33234688
00000000

33234688
00000000

00000000
33234688

33234688

121020203000
13515114693127107117154197239171126

000000000000
259141110168221817189

000000000000
259141110168221817189

000000000000
227107612720161089

000000000000
227107612720151089

227107612720151089

1

00000000000
324444122710

00000000000
324444122710

322444122710

2

77949658805676841001178372
000000000000

1

0000
1151

1151
0000

1151
0000

1151

7793955780567679991178372
000000000000

595229121241
0000000000

595229121241

000000000000
7788865278546767871138371

7788865278546767871138371
000000000000

7788865278546767871138371

3246352435353148761047045
033011001102

1
0

0
1

1
0

1

1111
0100

1
0

1
0

1

01
11

0
1

1

1
0

1
0

1
0

1

00
11

1
0

1

1

111
000

00
11

00
11

1

1

1
0

1
0

1

000000000000
14811142419171622533230

1
0

1
0

1

14811132419171622533230
000000000000

14811132419171622533230
000000000000

14811132419171621533230

1

2

61912381028388
000000000000

0000000000
11212314131

0
2

2

1112101
000000

1112101

1111231
0000000

1111231

0
1

1

00000000000
571226714257

00000000000
571126714257

571126714257

1
0

1

11
00

11
00

11
00

11

1121117775171946305
000102012100

54
00

44
00

44

0
1

1

1116116745121745305
000000000000

000000000000
1116116745111745305

1116116745111745305

1
0

1

1

0000000
10113321

0
4

0
4

4

0
4

4

0
2

0
2

2

1
0

1
0

1

0000
1111

0000
1111

1111

0000
1221

0000
1221

1221

0
1

0
1

1
0

1
0

1

000000000000
1538311852775411416818214313883

000000011000
75539538544382130137533463

0
1

1
0

1

1
0

0
1

1

1
0

1

00
11

11
00

11

244226851
000000000

243226851
000000000

243226851

1
0

1

0000
1151

00
15

15

1
0

1

1

00000000
14214221

1
0

1

1
0

0
1

1

2242
0000

1
0

1

0000
2232

2232

1
0

0
1

1

000
112

1
0

1

12
00

12

213251611102626396211
000000000000

0
1

1
0

1

11
00

0
1

1

1

1
0

0
1

1

211142
000000

211142
000000

2142

1

1

00000000000
6118379921111

00000000000
6118379921111

6118379921111

00
11

00
11

11

112346713
000000000

112346713
000000000

112346713

236331342414
00100000000

235231342414
00000000000

235231342414

0
1

1

1621631
0010000

0
2

2

0
1

1

1

11
01

1

00
21

21

11111
00000

11111

0
1

1

0
2

2

00
11

1

1

000
135

135
000

000
135

135

214912211191625
00000017401

274111991024
00000000000

00100000000
274111991024

273111991024

111
000

11
00

11

0
1

1

0
7

0
7

7

21
00

21
00

1

11

22
00

1
0

1

21
00

21

0
1

1
0

1

2
0

1
0

1

0
1

1

0
1

0
1

1

000000000000
691634122824376061432836

000000000000
661534102721376055412835

000000000000
661534102721376055412835

661534102721376055412835

00000
21211

0
1

1

1
0

1

1
0

1

0
1

1

11

1

0112
1123

0
1

1

1
0

1

1
0

1

0
1

1

000
121

21
00

21

0
1

1

11

1

00
11

11
00

11
00

11

0100
6111

00
51

51

11
00

1

1

0000000000
171127312510

12113
00000

2

1112
0000

1112

0
1

1

0000000000
16917311210

0000000000
16917311210

1691631110

12

00000
21121

0
1

0
1

1
0

1

0
1

0
1

0
1

1

0000
1121

1121
0010

0000
1111

1111

7628211323113238458710320
053101445200

1
0

0
1

0
1

1

1

2151114
0001000

12
00

00
12

12

000000
214112

00
14

14

11
00

11

111
000

111

1

1
0

0
1

1

000000000000
761611111572730318510220

000000000100
761611111572730318510220

0
1

1

761611111572630318410220
000000000000

761611111572630318410220

00
11

00
11

00
11

11

00000
13321

2
0

0
2

2

2

1
0

0
1

1

00
11

1

1
0

1

000
111

0
1

1

11
00

1

1

2123
0000

2123
0000

1
0

1

1
0

1

1122
0000

1122

0
1

1
0

0
1

1

0
2

2
0

0
2

2

000
211

211
000

00
11

11

10
11

1

0262001001000
915848403419255771779

000000000000
29242217981725523

212

29242016961725523
000000000000

00
12

12

29242016951725521

121
000

121
000

0
1

1

12
00

12

211196938202213

21

1

010000000
5341391223

2
0

2

2

54139112
0000000

54139112

1

1

13

1
0

1

1
0

1

000000000
133311212

000000000
123311212

2112
0000

0000
2112

112

1

1

1231111
0000000

122111

11
00

11

0
1

1

755735471
010000000

6
0

5

0
1

1

00
11

00
11

00
11

11

145735361

61
617

13
1

0
9

9
2

2

5

0
3

3

0
3

0
2

2
0

2

1
0

0
1

1

12222311

554254217141732677413

1

117311166

115651473825509216291228

300832288921513221322605928747270632717832446322983416520097
735120687212938861415109712831583906904521

284401223449312304346393476351282237
3204625101434412

00
33

00
33

33
00

33

000000000000
910137722101167171

910137722101167171
000000000000

151337341240
89676207837171

612513147

11

134138311161

11712333

0000
3131

00
31

31

13

0000
4221

4221
0000

01
21

2

2

11
00

11

11
00

1

1

11
00

11
00

11

00
11

11

3

0000000
11134181

000000
1134141

1134141

00
14

00
14

14

254311185222286249293321354326254226
120100113002

000000
1211313

00
11

1

1

00000
61312

42

2131

4
0

4

10
11

1

2
0

0
2

2

00
21

1

2

00000000000
21123946831

11123946831
00000000000

21

11123925831

1
0

1

235284765933
000000000000

1

235184765933
000000000000

235184765933

21558763910141
000200031000

0
2

2

1

2
0

2

232344235
000000000

232344235

103332131141
80200000000

1

1

233213113

1

000000000000
15202111303032733121515

111
000

1

11

15192011303032732121515
000000000000

15192011303032732121515

121
00

121
00

71

5

00000000000
11091041217162623

1
0

1

0
1

1

0000000000
891041217162622

891041217162622

2
0

2

0
1

1

11
01

1

142910
00000

142910

11
00

0
1

1

0
1

1

000000000000
32314134251162

32314134251162
000000000000

32314134251162

2
0

2

1721412421121
00000000000

1
0

1

00
32

32

1321412221121
00000000000

1

1321412121121

1
0

0
1

1

0
1

1

111125111
00000000

111125111

198177116141205135146206207210193175
000000000000

16614793122175103107162144152170154
000000000000

16614793122175103107162144152170154

2
0

2

323023193032394463562321
821145238792

561185171

11151121

788478161720748

1

2111131212

482454715241031

10548778771148

132193611
000000000

000000
211211

211211

00000000
11116151

11

116151

2
0

2

461112213
000000000

1
0

1

421112113
451112113

3

0
1

1

2131335211
000000000

2131335211
000000000

2131335211

233716282015553419581622
000000000000

1
0

1

000000000000
233716281915553419581622

233716281915553419581622

00000000
13113461

1
0

1

122461
000000

122461

11
10

1

1
0

1

1

000000000000
1725201915273038561198

1725201915273038561198
000000000000

48855681226413
000000000000

48855681226413

11
00

11

000
111

111

0
1

1

000000000
25434121622

1

32210912

2512271

0
1

1

000000011
715123423

221

1

4121131

1131

1

1376461915138333
000000000000

1376461915138333

111
00

31

7

1

141901
000

131901
000

000
131901

131901

1
0

1
0

1

141429351519915274912457
124218048539381015424613841537199112781715799

10105249232120897
000000000000

10105248232120887
000000000000

411
001

41

1065248232119787
000000000000

1

1055248232119787

11
00

00
11

11

000000
111111

111111
000000

111
001

1

1

00
11

11

0
1

1

00000
11311

00000
11311

11311

2
0

2

00
11

00
11

11
00

11

1616382198269268545
666107540945344011817898631174576960372

2441

0
1

1
0

1

00
56

46
00

35

11

1
0

1

02901400000000
8126114993719303711642

000
311

1

31

411335
000000

3

111335

14111111012

1
0

1

1
0

1

2

1
0

1

1

00100010
12211113

21

111112

46543353415161810602
080200000440

4574315341516186562

0
1

1

1

1
0

1

1

1

00
16

6

1

1
0

1

1

0
8

8

2

06601411114001410
2302728711010029725524225117936580

1

231

2

0000
7512

1

1

21

13

1

1

21

1

3
0

3

000
113

112

1

00000
11121

1

112

1

000000000000
1257132820111874

1

1247132820111874

7049522125710122
2491214134822921251714

112111

11111

135744492107311

3161211

211314114

1
0

1

3
0

3

58131304733134384652698731
1222366595131413179

41

1

41

11

2

1

1

913627935

12132111

3

1

1

124111624194

6636825222692

47141432

1

1

11

144

1

1

11111

11131

5926

16

21

18137197691322191810

1

423543533

1

6511111

1

111

4

13

1111

1

13542382850731721721707610931
000000000000

222142

13539362850711711681687610931

1

0
1

1

58221832323
0000000000

0
4

1

3

1

52221832223
0000000000

52221832223

11
00

11

9
0

6
0

6

3
0

3

1

000
253

253

010000000000
861609987751451842194209211772

000
311

1

1

11

1

0000000
2121261

2121261

861529987741451822174189211071
9331211134020315013177

8203224182449711149187

699955514381113115251707556

11

21

2
0

2

0000010000000
14235107531111181453

0
2

2

31
00

21

1

52212212
00000000

1111

522121

000000000000
116375258912933

1

116365258912933

0
2

2

0
3

3

000000
3317234

3317234

2
0

2

1
0

1

1

6111131
0000000

6111131
0000000

1

5111131

22112
0000

00
51

51

0
1

1

11
00

11

0
9

9

7

0
1

1

1
0

1
0

1

000000000000
7261210162017307947

7261210162016307847
000000000000

7261210162016307847

11
00

11

0000000
1111112

111112
000000

111112

0
1

1

1
0

0
1

1

000000000
5743117212

574210521
00000000

574210521

11211
00000

11211

42
00

02
42

4

1

0
1

1
0

1

11

23
00

23
00

23

1
0

0
1

1

23143151139
0000000000

0
1

1

23143151138
0000000000

23143151138

0
1

1

2111
0000

2111

00
12

1
0

1

2
0

1

1

000000000000
11421252531021

11421252531021
000000000000

1132125253102

11

000000000000
813979354645420

813979354645420

444511101725361
00100000030

0
1

1

0
1

1

0000000000
1324410162211

1324410162211

211171223

000000
411111

411111
100000

11

1

1

1

11

1

222114
000000

0
1

1

2112
0000

2112

00
21

21

2
0

2

0
1

1

23311
0000

0
1

1

2331
0330

21

31
00

00
31

31

31

195817241321123476113269
1400032000000

43726631413
0000000000

43726631413

41566552101218242
000000000000

8

2

1

21124422222

2554586101622

3

010000000010
6115743541122283

141

34333283414132

2224171781

4

61
00

1

6

0000000
4222186

4222186

00000000
141211211

141211211

3

1
0

1

2211154318
0000000000

2211154318

11214311
00000000

21

11212211

71112614
00000000

11126

1

11

1

3

1

4

3212121
1000000

11
00

11

11
00

11

0
1

1

1
0

1

12
00

12

0
2

2

96314452221
00000000000

00000000000
96314452221

94314452221

2

1131
0000

3
0

3

1

0
1

1

1
0

1

00
11

0
1

1

0
1

1

258123136106182189241207278218269165
000000000000

000000000000
258123136106182189241207278218269165

258123136106182189241207278218269165

000
411

4
0

4

0
1

1

0
1

1

000000
212221

00000
22221

2

2121

1

1
0

1

0
1

1

2

100
111

11

1
0

1

4

0000
1149

1149

00
13

13
00

13
00

13

00000000
11321351

00000000
11321351

11321351

1
0

1
0

1
0

1

21441
10410

1131

12253102

4
0

4

2

112
000

112

161651210424283127291515
000000000000

00000000
113284111

113284111

0
4

0
4

4

000000000000
1615498420202716291415

1615498420202716291415
0001073000201

1

121035510017261010106

3117311111

31195231316

11

121792312

11
00

11
00

00
11

11

1

0
4

3

1

11
00

11
10

0
1

1

1
0

0
1

1
0

1

000000000
111211512

1

1
0

1

1121512
0000000

1121512

11
00

11
00

11

3
0

3
0

0
3

3

0000000000
2812764711

2
0

2

82764711
00000000

82764711
00000000

82764711

1

3339520144932
000000000000

000000000000
3339520144932

2139520142932
000000000000

2139520142932

21
00

21

00
11

11

31122

2
0

2
0

2

1121
0000

1121

113018921
113118923

2

1
0

1

4603743353574611737395397402509592325
0000043000120

00000
13311

13311
00000

13311

000003000000
362302289289405540282264231401505266

00000000
104421421

11

2

11111

311311

9

0
1

1

52216824333
0000000000

1

52216814333

11
00

11

000
313

313

00
11

11

2
0

2

0000000000
12126714121

12126714121

357287281278403395277249223398499261
000000000000

357287281278403395277249223398499261

1311
0000

1

3

11

1
0

1

1
0

1

9555425947940101121157956951
0000062100000

1221136221531
00000000000

1

1121135221511

1

11

1
0

1

824191244451142
000000000000

824191244451142

2

15759615517141413175
51231641356110

22212711961

1

1

22130124221

1

1

1113334242

72122214

13221355344
0000000000

13221355344

000000000000
24112985325172230168

24112985325172230168

2617171911116131527251527
000011000010

1111

11

11

22111397138913101422

21

11

3212101241153

11

14

1

2241118

1
0

1

1
0

1

0
3

3

0
1

1

0
1

1

2212333
0000000

2212333

0
2

2

344612214662316
000000000000

344612214662316

0
1

1

44344121333262
000000000000

44344121333262

00000000000
141251657101623

12

131251637101623

000000000000
16332118223781

0
1

1

16322118223781
000000000000

16322118223781

000000000
1014447613

000000000
1014447613

1014447613

0000000000
21539234474

0000000000
21539234474

21539234474

5711161510132965
00000000000

5711161510132965
00000000000

5711161510132965
00000000000

452345151

1111436

2

4531225753

31

000000000
112131861

000000010
112131861

112131851

0000
3211

2211
0100

0000
1111

111

1

0
1

1

1
0

1
0

1

00
11

0
1

1

0
1

1
0

1

15223211
00000000

00111011
15223211

000
121

121

00
11

1

1

1
0

1

10
41

1

3

143541612123
00000000000

143541612123
00000000000

0
1

1

1312
1000

312

3231411

0
1

1

000000
311472

113

1

1

11

143

1

000000000
6211021111

6211021111
000000000

6211021111
000000000

6211021111

4
0

0
4

4
1

3

0000000
535810161

535810161
0000000

1

2
0

2

13581016
000000

13581016

11

00000000
121711411

121711411

000000000000
95768628671236855

000000000000
95768628671236855

03210070212512
95768628671236755

1

1

153213952522

8

1

1

822214213

621131125

1

1

12233

1

1

7119

5

1

7

3

1

1

215645582130354
14124441616001

22

11

1

11
00

00
11

11

11

2

1

0000000000
55212413123

55212413123
0000000000

55212413123

0
2

2

1
0

1

0100020000000
455143274432066881091087152

10181441712232284651218
000000000000

10181441712231284651218
0000044000000

872342125122331155

1

1231273820221

1

33

111

1

159110303421832

1
0

1

3430262325131325056504744
1000011100000

300000000110
9961145510166758

685114558136648

1

1

112

1

000000000000
155924392330261910

155924392330261910

000000000
525132423

525132423

9109516599717172323
000008000000

21

85711336944142019

221331212

114212322

0
1

1

11
00

1

1

0000000000
12324719473

0000000000
12324719473

44

1232471573

113

2222

0
1

1

1

21

419925342880229433242469228325382644418740103635
282320336111

202020112520
334715532053125419481628173618291922357632242683

000000000000
2019366505298910198447626610228121051662

153625111523224
21669913143457736

111

11

1

1

1

835118171

12

31132816511

1

2014349498279898185432586547227020951655
263739542447293023132714

1985310457223867133398552524225220621639

21115233322

1111231123

11

0
6

6

22236
00000

22236

0000000000
1111163111

1

1111153111

000
131

131

311112155101121
13041166150993710231418124911581271125011031000

212

300010100020
15142112557169

1131121

1114172345148

00000000000
17381139318

17381139318

000000000000
443848353117783366394726

25332485331

423345322913702863394425

412121212
000000000

412121212

5365604713982396671484662
000210116000

4964604513681376164474461

41121121

212

1891791323172125203015
000000000000

1891671020121920162113

1233525492

0
2

2

00000
22122

22122

000000000000
221313411826112425139

221313410826112225139

1

1

1

424263374445807782615839
000000000000

424163374445807782615839

1

000
211

211

0000001000
65314181111

1

11

1111111

1

44313158

4211227

503049254524937097574530
000000000000

106136138333557251310

422411221

321424112113552427272917

32221426222

13313121

371511741

00000
22944

22944

0000000
3112211

3112211

111031210123
104995012437617241203881845858979848792

1423114111

1

662036283022667589786557

243222412

183122117118144112104132109165166147

6146447157151224

353638115108

3232242622

572590859387312878288293253408368413

582951252811524568424417

156163165190195161344274306264186143

000000000000
222137191312394439401421

222137191312394439401421

000000000000
1613954101327217

1613954101327217
000000000000

000000000000
1613954101227217

1613954101227217

1
0

1

000000000000
84996781210281369837534693689608784944

84996781210281369837534693689608784944
1611012514100

11

2111116
0000000

2111116

4283577121611
43956129162021

1111

1114144

1

7808676989561283750409490421541703887
000000000000

7808666989561283750409490421541703887

1

010000100000
895466221630578

4522213513234

122212671512

311232242332

1112111413
0000000000

1112111413

26395121274055109142202221
000000211000

2

2435421723333996111182120

1

2113111

1

1111

482141010262

11111

1232211020
1452434621

222236

1

100000111000
284042344322315146384627

2322114941

1

6

11122

11111

92931303212153626151616

11

1

141839761313132410

4

1

16609257113755387119151452
000000000000

000000000000
16609257113755387119151452

000000000000
16609257113755387119151452

38121273357387657
16609257113755387119151452

846764410669107242436

45413557449

11211

1

1

111

1

1

613142662145
00000000000

613142662145

11
00

11

00
11

00
11

11
00

00
11

11

1
0

1
0

0
1

1
0

1

15108213161823
000000000

15108110131623
000000000

15108110131623
000000000

00000
22141

22141

0
2

2

11311
00000

11311

0
1

1

0000000
5645163

5645163

41414261
10000210

31435

111

1
0

1

1

1
0

1

11

1332
0000

0000
1332

1332
0100

1232

1

0
1

0
1

0
1

1

261542178326146423463302713
267033572149519825693544313032463700279131372058

00
11

11
00

11
00

1

1

343520513250394753294516
000000000000

343520513250394753294516
040000000000

332018312935333742274113
0000017011021

23910109826193423267

1011821191071764135

11

1
0

1

0
1

1

01000000000
111921023222

5

1

11321023222

00
18

14

1

1

2

411154781
000000001

41115478

3111
0011

1

1

2

0
1

1

2
0

2

1
0

1

1015138152
0000000

0100000
1015138142

1
0

1

11
00

1

1

0
1

1

0
2

1

1

000000
2136131

2136131

0
1

1

5

1

1111
1100

11

000
111

111

1
0

1

0
1

1

4
0

4

1
0

1
0

1

1
0

1

000000
222252

222252
000000

000000
222252

222252

000005000000
19161039165018252415159

3
0

3
0

2

1

000000000000
147930133616212312107

147930133616212312107
20125111310000

11128422213

141433

1064491391521794

561939241352
000000000000

0
1

1

010010010000
551939241352

541929231352

000102011000
695445516488611702515638620642728522

000000000000
634394472444565608491594583609685461

634394472444565608491594583609685461
423276367270409370325383354435502337

141

111

2233612225

62123521

2

1

8161463

9610381091014171417

12111

211322311

3452531112

3449333667555

10

21111421831

1115112

1171141812921

121

1114311111

6215391616182841852

1

111111

211

391110183015231832302517

632522173127283741163131

561524233825295521557137

721781113149106

613435531

111137111

118121111

1

3111

1423135354

432512781

31152421

3113621

2

1

000408000000
605144434692244336334361

111466318152663
000000000000

134522112

11121116141463

493738334366233834273758
0201013100102

212

373022263238183725153047

1251661115218979

2648241702262181213159283210
120302031011

163115125123714273016213
000000000000

07011426000100
162015124123714273016173

1

2112161221

22112

4552810141954

72551625369

11

1

1

1

1

12444125611

000
1114

1114

4
0

0
1

1

0
3

3

2
0

2
0

2

6313396566
000000000

000000100
6313396566

11

1

625396466

5

1

11113

2

00
11

11
00

1

1

00000000000
355814710124

1131
0000

1

121

1

41223123
10223123

1

3

14142561

00
11

1

1

38414218154169109522
000000000000

3743218154169109522
000000000000

3743218154169109522

15
00

15

0
6

6

0
1

1

000000
222341

000000
112241

0
1

1

21
00

21

1231
0000

1231

1
0

1
0

1

00
11

00
11

1

1

42841872791516516
000000000000

2000000300
42611412615

1
0

1

211411
000000

11411

2

0
1

1

0
2

2

000000
214825

214825

1

1102662443651
00000000000

1
0

1

41
00

1

4

00
11

11

1
0

1

00000000000
121422043521

121422043521

0000
1243

1243

0
4

4

7
0

7

64

312214
000000

1

13
00

1

12

1
0

1

0
2

2

1121
0000

1121

1263112121
00000000

1263112121
10000000

00
12

12

111
000

1

11

0
2

2

67
2

65

33
00

3

2

1

000
2811

2811

25

00000
11111

10110
11111

1

1

715212
00000

1

37
00

37
00

1

27

00000
48112

1311
0100

1

1

11

1

0
1

1

000
111

111

2

211

0
1

0
1

1

1851
0000

1
0

1

1751
0000

1251

5

125917171223157714391656166214611983141814731066
215538914165242528243512

0
1

0
1

1

1

1

000000000000
316775249871237

1
0

1

00
11

1

1

010006000100
2571215324314

241314113

223263111

431124221
100000000

211124111

1

11

2

1

000000000
111282712

1

11282712

2
0

2

121
100

21

1
0

1

1
0

1

11
00

11

110005000000
274931444362455275502221

0
1

1

00000000000
8910864512121

8910864512121

000000000
1151012654

1151012654

0
1

1

001016000000
415911122624252920107

1

112617131115

1

1

1105377192225431

11

1

2122634561

0000000000
31322331312

11112

3132122121

0000000000
4313222111

33

1

111

111221

11

000000000000
17171112191311151422411

107365711871443

710861467788

1111
0000

0
1

1

000
111

11

1

1

6757736987388117748447961069732866694
1142133714511277

27
0

27

00
11

11

0
1

1

1
0

1

00
14

13

1

112
000

112

0
1

1

00
232

2

23

1
0

1

12
00

1

1

1

0
2

2

2
0

2

3216212221018953
010000000000

1

316212221018953

0
1

1

0
1

1

00
21

21

11422131
00000000

1

11322131

1
0

1

1
0

1

0
2

2

6577026916967967478007701036716846681
918867741091394262608911044

4313133421

1

413232623

1

171

121

221

19109111212201815202510

43272336153127

142312313

133

463930112216282948445236

731245547442

2595118621151710185

325423136

1143211310134

11

615455644523

11531441313127104

11

1

5522061314169671

31

3111334542

114116356111

21224221

32112912

1392334

211

12

2

2

1

211132121

23632234351

1112221

11111

3729232521

1

421533331

1

311341391313382261385368584402505296

112231

1

31134128421

11121101342

7410493861611321871681655855228

11

12

1

115242111

26211516112941284318246

1

11

341486654263

00
12

12

458692371438476564559400476442433290
010000000000

2

6315444305179634045627132
458684370409473559555394472441433290

31322612342

1

3231

255951821323026181613

99277195613661

516310221881

4

3

278296283315351359394262320278274197

1

11

2

45142441232

111

11

1

116779151213218810

1

17731738614756

72123814

62364346

11205181219116912920

1

3

1

2113211

11

2

414341211143

41418444256

1

644337462491

211111

1

2

1311
0000

1

311

1122154
0000000

1

122154

00
251

251

31
00

1

2

1

0
2

2

1
0

1

000
111

1

1

1

000000000
2311251212

1

11

11

1

21115121

12

11

31
00

3

1

1

1
0

1

4051334524879475152876018
2341113113100

11122
00000

11122

11
00

11

1310101713401541733204
3021212307611

44721313

1

113102102

34

1112

1

953391163615173

1
0

1

161091051355519628308
040000000000

1131331116121

1

1

224631050438110145

1

35422253241

111

45351261321

000
161

161

22

4
0

4

000000000
342314285

1

342314284

0
1

1

12241111
00000000

12241111

0
1

1

0010001
11341211

1

11

12312

2
0

2

4321112431542
00000000000

4321112431542

0000
1311

1311

11

010000100000
28603518836788095156595022

1
0

1

132829282532667613446284
000000000000

3

32027211922394191512

1082461027354341272

10012407000000
143413484091616131515

3

101333274129889

321456547576

1131542232
00600000

21211

11

1

1

1

11

2

7542

11

0000000000
112112123131

112112123131

212
000

1

112

1

111
000

11

1

2121111
0000000

0000000
2121111

2121111

0
1

0
1

1

000
112

000
112

2

11

00000001000
576713131011

0000
3311

3311

1

0
1

1

111
000

111

000000000
211321121

211321121

2
0

2

11
00

11

01
11

1

2
0

2

000
216

6

1

1

1

1

3
0

3

3611

0
1

0
1

1
0

1

0001102541219000
491199228486310522442543476439537303

346313221432
00000000000

1
0

1

1
0

1

0000000000
32631322432

32531322432

1

0
1

1

5

070148010103120
428141176368225296343422324359431229

1031012141913231211119
412124171197217269323401302336411224

1318262615292327269144

2

1

113121

388103134157186221286345263313385209

111212

15

161041881719211922185
000000000000

161041881719211922185

0
1

1

52435085771668793117679270
0001015000200

0003116111000
13913273163342959202714

41

113291022246

3336963816433

31612113

6566121927173711245

1112222
0000000

1112222

1
0

1

111
010

11

643165248347133
000000000000

643165248347133

000000000000
1145141315511881815

1145131315511881815

1

12
00

1

2

00
11

1

1

223493615851
00000000000

223493615851

0
4

4

1

1
0

1

2
0

2

3111

181719132116272217172832
000000000000

181719132016272217172832

1

122312520743
0000000000

122312520743

12211122
0000000

12211122

1

0
1

0
1

1

81029522614159112
000000000000

1
0

1

4816318478651
81029522613159112

122

1

21121124621

1

11121131

42411053411
0000000000

0000000000
42411053411

42411053411

0
1

0
1

0
1

1

0
4

0
3

3

0
1

1

5
0

5

22312871271
0000000

7
0

0
7

6

1

1

2

3

2

0000000
22312721271

0010000
22312721271

24

1

1

2

464

1

4

1745512

2

334

1

1

132241

2

51
00

40
51

1
0

1

1
0

1

0000000
12103111

1253111
0000000

0000000
1253111

1253111

5
0

2
5

3

3

0000
36821467

36821467
882223

430
1584

1154

90
201

11

1

4674
74123

12

1435

1

1

113

28
0

28

32
10

2
0

2

0
2

2
0

2

24

21
00

00
21

21

1321611322
00000000

00000000
1321611322

020000000
1321611322

1121611322

3122227131
000000000

000000000
3122227131

3122227131
001000000

311226121

1

21

51
00

1

3

2

115112871659528520323423617511894
01010119040800

00
32

00
32

2

3

000100000000
132083911311418171494

30112101121
42323482132

1221138111

1
0

1

00
11

11

411
000

411

518427521678961
000000000000

1131221311

34111318415551

1216

111153

323521331
000000000

323521331

11211241
00000000

12

1121221

0
1

1

0
1

1

5029263336128133167143614950
000007000000

33103916129411492302635
451620312859129159134483746

32822216226

6241021328353810110

21233121

1

13656222

125131324

000000000
1122422121

1122422121

474262046812103
001000000000

463261746812103

1

3

51
00

51

1

1
0

0
1

1

1

0
7

0
7

7

0000
1311

000
111

111

2
0

2

1

3
0

0
3

3

00
11

11

1
0

1

000000000000
171161838848

000000000000
171161838848

171161838848

010001000000
497139271282214154

1
0

1

000000000000
1131238210473

1

1131238110473

1
0

1

0
2

2

1

000000
111131

1

11113

00000000000
123521433542

2

123521221442

11

1

1

1
0

1

000000000000
211548123561

211548123561

464242493156332740634027
000003000000

0
5

5

000100000000
8174555281051

1

5141112753

1

3344321521

0001013002000
323823281939181921442923

811116478810141110

113222

2220101511934226119

22615532113

1432223161

000200000000
6312127910611963

1

3111963847542

1

1

31116214421

131123264611
00000000000

00000000000
131123264611

121122263611

111

00000000
12112218

12112218

141
000

0
1

1

131
010

121

1
0

1

000000000
2162522445

2162522445
020000000

0
1

1

000000000
2102522445

21

1

92522435

2
0

2

0
1

1

000000
111433

111433
010000

11433

0
3

0
3

3
0

1

2

0
2

2
0

2

0
3

0
3

3
0

3

1

0
3

3

2
0

2

1
0

1

0000
3712

0200
3612

0
1

1

00
11

1

1

0
1

1

2211
0000

1

21

2

1

0000000
25425102

2422492
0000000

0000000
2422492

0000000
2422492

1421262

1

123

1211

18141510181519536811114
000000000000

18141510181519536811114
000000000000

1

18141510181518536711114
001000151000

6121332146
0000000000

1142

1

11

51122214

13422
00000

13422

576386264321
0000000000

554386253921

2114

1

1277211481620542
114010111000

21

11

111348121

1052143263532

11

2

11

1

00000000000
227167913224

227167913224
00100221111

263411
000000

263411
010000

2

21

1

1

22311

2131611113
000000000

000000000
2131611113

2131611113

798761734124
0000000000

111
000

111
000

000
111

111

797651734124
1000000000

2
0

0
2

2

332321119111
0000000000

332321119111

111223512
000000000

0
1

1

111223412
000000100

1

1

11123312

0
1

1

111
000

0
1

1

11
00

11

000000
143371

0
1

1

01
13

12

00000
13361

335

11

1

11
00

11
00

11

2181

0
3

0
3

3
0

3
0

1

2

2
0

2

156205205199214189169118131177175137
89295110879641049887136113951995998944638

000000000000
7614664665260777968977168

000000000000
577312812617121074

00000
11341

121

114

60

51529812216121074
000000000000

1

42771211451073

11

12211261

1

1

1
0

1

716961544459516256876464
000000000000

000000000000
716961544459516256876464

686347534158496148836362

28111232

12

346121411

2221112

000000000000
2330332733285288111362118

15241823202133708219148
000000000000

15241823202133708219148
14461121416103

1364581220172416133

1141012118113942212

000000000000
8615413719182917710

0030115411000
8615413719182917710

8612412614141817710

581312111619114112172
000000000000

581312111619114112172
000000000000

00000000000
2511761414430515

2511761414430515

122114411

000000000000
322331437711

1

3112133711

11217

1

632560770658739593104310981642676660413
11114513157212

5

11

1

226481010108217

111

71319431413296675818

42111148

036435577324
6215177205857095569999891498663655395

010000000000
352413132714143414283418

24149122311721992515

119413371351883

111

3631346884
0000000000

3631346884

232262132
000001000

232261132

1111
0000

1111

000000000000
352927343535523450485911

3236343415111

1

531211

322422312931463134334810

000500101010
106168265176120133387486889120101105

4

628241025238984113575029

20169814131458407922129

1412321

211421391448095237358693373865

22221111

16203039402135352514839
000000000000

15202938402132342213838

11131311

35645481611
0000000000

35645481611

314175290214367230394254340336356135
000000000000

314175290214367230394254339336356135

1

1768511152318159911
000000000000

2

164638111916133410

112332154

11221111

275234124232
000000010000

1113212

264131112112

000000000
121452661

121452661

0000000000
1112143513

1112143513

000000000000
211213996826

211213996826

36121

00000000000
41174231881

41174231881

9212

3122132522
0000000000

322132512

11

211

00000000000
521526579115

521526579115

000000000000
756046687273325770484646

756046687273325770484646

0000000000
3144517253525

3144517253525

11

758232102011

124291

11

111111

11

121

23541108

5488161211235194754

00
11

11

0
1

1

21226191410293884546
631451581511011132924111228875770

0
1

1

1
0

0
1

1
0

1

000
124

000
124

124
123

1

00000000
711861611

711861611
30000000

1

41186161

000000703000
1068616921342261011

0000011
33221661

0
2

2

11
01

1

3312144
000000

3312144

103561491218136911
001000050000

0
1

1

000000
121323

11323

2

21
00

21

92421399976911
010000000000

91421299666911

1

31

1
0

1

111
000

1

11

111112
000000

111112
001000

1
0

1

1
0

1

12
02

1

1

0
2

0
2

2

00000
31221

1
0

1
0

1

00000
21221

11
00

11

1
0

1

0
1

1

11
00

11

00
11

11

24511731641
0000000000

0000000020
24511731641

05
15

1

000
112

112

1351163921
0000000100

1

1341152721

1

11

001000100000
2201622141235661143111

00150002000
214151613123161102111

474662542727
000000000

1121

1

1

1

471652541696

1

0
1

1

1
0

1

0
1

1

0
1

1

1112347911
0000000000

1112347911

11
00

1

1

0
1

1

61
00

61

152117122
11201371

2134

2111

1
0

1

1
0

1

0000001
1411271

141127

1

11
00

11

0100000
6611261

1
0

1

0000000
5511131

3511121

11

1

0
1

1

0
1

1

2
0

2

11
00

11

0
1

1
0

1

0000
1351

1
0

1

123
135

12

0
3

0
3

3
0

3

000000
211126

211126

71933251212255281857
001000100000

00000000000
152222252011

00000000000
152222252011

1111131311

1

32112127

00000000
111165111

0000
3221

3221

0000000
1814311

1814311

00000000000
1468122111212

1
0

1

00000000000
1467122111212

1467122111212

000000
113211

113211
112211

1

1212131
0000000

1212131
0200000

213

1

11

32554451320322
000000000000

000000001000
32554451320322

32554451319322

000000000000
146454131520211

2
0

2

464237141821
0000000000

464237141821

1114121
0000000

1114121

0
2

2

0000000000
1014106813306722

00
11

1

1
0

1

2
0

2

00000000
3434717392

3434717392
00000000

3434717392

0
1

0
1

1

414634612272
000000000

24331611261
000000000

2

1

1

14241

1

13331821

11033111

1

111010101000
174847573353100138775171716

21

7336547814965
000000000000

21
00

1

11

053044401
153355451

13115

0
1

1

131

00000000
11213453

11213453

00000
32111

311

111

11
61

3

2

0
1

1

1

13261151113111
000000000000

13261151113111

1
0

0
1

1

0000000000
1111113111227426422

00
1584

1584

74556102538
00000000

1

4235692424

1

12117

2

4

1

2

0054561510161
1155561512161

12

11

00000
23254

23254

1

0
3

2

1

2373215202116134
000003030000

2
0

2

0
1

1

11321
01000

1

32

1

000
112

112

1

0
1

1

1
0

1

00
11

1

1

1112211
0000000

1112211

0
2

2

21
00

1

2

1
0

1

1
0

1

103514461
115515461

11

11

11131532
00000000

3

1

112

131112

11
00

11

1
0

1

00000000000
2221525111415375654

00000000000
2221525111415375654

2221525111415375654

00000
11112

111
001

11

111
000

111

13
00

00
13

13

337422201629152
000000000000

1
0

1

337322201629152
025222141017102

4

1

13

11

1

12

22551

1131

2
0

0
2

2

211261
000000

000000
211261

000000
211261

211261

2511854503633401714
00000000000

00000000000
7754334211514

11
00

11

7654234211514

221159111
00000000

1000
1111

1

1

1

12114811
1211489

1

1

16314223192812
00000000

000000
164122182812

164121182812

1

11
00

1

1

311
000

311

00
11

11
00

1

1

11
00

11
01

1
0

1

0
3

3

000000000000
341131011047173179258409935834

0010012211010
341131011047173179258409935834

000000000000
6623747352769100178171115

311

1

662364435276999177171115

002002112000
15202727151546565947175

86171391130302741134
000000000000

7610108926232039134

173124772

121
111

1

111

1111

21251241616193
0000000000

21251241616193

1210106661
2221116661

11

1

1

1131
0000

3

111

00000
31111

1

1

11

111

13313630213062100161292914
851182120291716234

13142481021011
2151210816183872615

121112

111

3122145191

174323216202

1

121135

1611231

11133

11
00

11

1

000000000000
112552342411

112552342411

155347610221
0000000000

155347610221

1

0000
1131

1131

10010
11112

112

11412583112
12412583112

1

00020141011
221218911212

11

1312

13111

248

1

1

1

000000
131249

131249

11
00

00
11

11
00

10
11

1

2

0000
1121

0000
1121

1121
0000

1

112

11362910241813131495
465323812201188925013359292930163378479251861931

1

11

186313441398133917892577200818032244205521841133
0821012000000

8671616169751361361709812674
000000000000

1

8356585258651261241399112270
411012023200

1

121036214

313148254161

11123713642

11

111

22418116465

343242857591

12

1

125

1111

684545383635109961057210062

111

221354128743
000000000000

221354128743

811

121116

11
00

11

000000000
1434589171

1434589171

00000
113171

00000
113171

113171

1222103521
000000000

1

122262521
000000000

1

122261521

0
4

4

591284136740382526537
177612631332127717182479187216642054195220561057

211321232523
00000000000

211321232523

00
12

12

332

00
11

11

45218713212

0
6

6

1

67065164461582114028477851088718757442
3931272717211623990744448

1416415324

211111

63341041676

247185181167297239258194240175208112

1819910913373339202819

236253111

11

2113141

1815999919341444

3441619135331

121521451

44421121351

1

1

1

271415211650356239244411

11123

6273341

11862323

237251074105

213220222122

11

1115121

1

142215212

12254388

247106881029522

32101

2111221211

121

1111

121122111

121149232221

71316134755610

8289857910416918514630519416975

1111216203331

12201917101916354916189

113

11365311376385

1111

41121121111

21141941

595433474060555153303533

21413144

883847295856212426395542

116113

11143

1

1111

1113

113312111

122121112

3

11132

131143246

12

26123155139165170736959226021

11311041

111131313

128212218454634

1162114

111728

221113072

121642

11112514211

3

1716132011105231527411913
104559367464788299497482992012041239606

41141438

121

2

21511

1

523213121

4232145588133

2

1225131451

112711112

863361610151112164

752532284440149831538111258

448239295305389290342313258562603242

2222211

21111921

181513999161824271835

19253

1611211

125184213

5388728234

31141121238

73712211371331

754036322949916165264544

11211

1

1330133422

12323

11811

11

829961217141215123

5262658710421

245105122114180127109103161239245109

12123134142

795452575414156

51711

1

1111311

1321233488562725341774

1712232

12112321645

20162267202724262799

1

7243742833126

1

2

11122522514

53317461212

1

22232612

3211451223

11

131413212

531111212741

1223

1111

1115112312

1218111121

23

1

322425

31101824752

23785175131613232115

12211

422121

222118182225232016172114

2121111

1314111

1

11112232

1431

2111

1113113221

21212211

1

22

250340854429944938861480663222782709515
0411300216300

001001000000
954070384469176126156809959

60275829334214188116527936
000000000000

60275829334214188116527936

4135591529281784
4111035941462

2456102023322

1

31128461720912111219
000000000000

31128461720912111219

764748515881108116111645129
000004000400

000000000000
2344510989852

2111621261

1

11

2

111

213343634242

1

214243112332
9141212201541536614138

551473231316871

1

111

1

11111423

156566103243121

1127611

362421292318484523252911
000000000000

362421292218474523252811

111

29611510341010131348
000000000000

29611510341010131348

0
1

1

0
1

0
1

1

233130642119334223732856035321302559426
010000000000

002004000013
230125938716129818328250626320912538405

1113223232

230025838516029517728050326120882535402

1
0

1

010004000040
203524243841363055281616

114311131

19332424383333295427915

1076357612181145
831132263835

1222135611

123241192

000000000
44516411171

44516411171

0
1

1
0

1

4
0

4
0

4

1
0

0
1

1

443512611
000000000

443512611
143512611

3

1
0

1

11
01

1
0

1

2

1

182471164174159283197290359302170191
0122325435201

211

111
000

111

1

164394147143137207172236287273154173
482913181813101821382250

1

11816310281861815115
212115205410

2262310126321

11133

111511

437491453

11118523121

51
00

41

1

41336811206678
000000010001

31215543133

112137165347

010000000100
502475964357150831321364747

91181531437719810

318012161142521

32114145191

11112

2

39121359141946824

83576761417648

719191681346619119

1

111

1

5111

1

842235977131014

337742821355

111111

1

84110321208

1

000000000000
255494143038131

255494143038131

448233484474565243655558
61156131581516104

2124774411

11

204420392843221513212640

1696382110219221613

1221211

16131111

1962448102352
00000000000

1962448102352

0
1

1

0
1

1

43744262791
00000000000

43744142791

12

151611131

22

29410888254534
00000000000

2
0

2

27410888254534
00000000000

27410888254534

11243

000000000000
6266342837215105

6266342837215105
0100000000000

311323211

5

68521281414104

1

3

6

101851373381417858
000000000000

1

000000000000
91851162871311225

91551162871311225

1

2

0000000000
1215116632

1215116632

000000000000
871148564927583879713910786

871148564927583879713910786
110000039000

1121
0000

1121

0
1

1

0
2

2

33822
00000

1

2

2382

100
314

24

1

0
2

2

0
1

1

1
0

1

00
12

12

0
2

2

00
12

12

00
11

1

1

861048054887382827713610785
000000000000

861048054887382827713610785

21212222
00000000

00000000
21212222

1
0

1

00000000
21212122

21212122

1111
0000

1111
0000

111
000

1

1

1

0
1

1

1
0

0
1

1
0

1

2431651529211
00000000000

00000000000
1431651529211

00000000000
1431651529211

1314611

1

1

1111

1

1

1

11458201

11

0
1

0
1

1

341312
000000

1
0

1

000000
341212

000000
341212

341212

371416420390582514432373416495384284
000000000000

000000000000
371416420390582514432373416495384284

100600102000
371416420390582514432373416495384284

2452114193711
100000011000

1452114182711

00000000
51252132

51252132

00000000000
32222743343

32222743343

113
000

113

101000110100
365405413378564506422359402485379278

232315307316464353339249300391299189

1

112131111

732821255249122522462350

1

445571313889486166263735

11

1115176

1142

14592682015717183

000000000000
143959219102308167132271141713053119411357415753169469428

000000000000
143959219102308167132271141713053119411357415753169469428

143959219102308167132271141713053119411357415753169469428
000000000000

143959219102308167132271141713053119411357415753169469428
666232322204404185440499528810776329

22747141217721292613

12923854494977559122221083211987107131238514030153118558

172226342242204337331712

211581314691511172517

746399373350551340580664592834791499

434419232657677381264528
000000000000

434419232657677381264528
010004011001

121211431
000000000

020000
121111

1111

1

11142
00000

111

1

131

000
121

12

1
0

1

000000000000
8421115106325

0
1

1

11

0
1

1

21211213222
00000000000

21211213222

5123312
0000000

5123312

0
1

1

00
22

2

2

1
0

1

1
2

1

644148988496
000000000000

644148988496
001000002000

1

1

1

631147862492

111243

2224915113042415092815
011512115020

00000000
11531411

11531411

12
00

12

5111

431256112
401255112

1

3

1
0

1

1

0
2

2

0
1

1

2

1011146241714157137
000000000000

1011146241714157137

00000
21152

21152

000000
124132

124132

313
100

1

113

31128131
0000000

31128131

0
1

1

1
0

1

1
0

1

11

0000
2112

2112

11

00000000
11621112

11621112

1
0

0
1

1

0
2

0
2

2

31112242
00000000

00
11

1

1

3111141

00
11

11

000000000000
5435712855751

5435712855751
202004200111

1

11532341

1

1

12

22142441152

111
000

0
1

1

1

0
1

1

42111381
00000000

00000000
42111381

42111381
00000000

2
0

2

00000
11111

11111

21128
00000

21128

1162315

000000000000
175237172545512

000000000000
175237172545512

175237172545512
000000000000

041002156000
175227172544512

32

1

122

1

12311410183552

11

11

1

11
00

11

4

1
0

1

1

0
2

2

111
000

111
000

000
111

0
1

1

0
1

1

1
0

1

451142165138240216360433532482455359
000000000000

451142165138240216360433532482455359
000000000000

451142165138240216360433532482455359
000000000000

451142165138240216360433532482455359
000000000000

451142165138240216360433532482455359

1

1

1
0

0
1

0
1

1
0

1

59321151

93037233022224569779
2332554723103

2221

0
1

0
1

1
0

1

000010001011
52332212414143341475

00
11

00
11

11

0000000
3112431

11
00

1

1

311431
000000

1

311331

0
2

0
2

2

000
221

221
000

221

4132
000

000
4132

4132

000000000000
5192112201471430422

5192112201471430422
000000000000

5192112201471430412

1

0000000
1682441

1682441
0000000

1682441

000000000
131342311

00000000
11342311

00000000
11342311

11342311

3
0

3
0

3

11

00
11

00
11

11

3122181411
000000000

3122181411
000000000

21211111
00000000

21211111

111613

0
1

1

170169312741200771741129022683867355248371

000000000000
14242549042053434888126106123130287

11
10

1
0

0
1

1

14242449042053434888125106123129287
000000000000

14242449042053434888125106123129287
000000000000

1
0

1

000000000000
14242449042053434888125105123129287

14242449042053434888125105123129287

1
0

1
0

1

48335497545847047627557425321370738103166151677593185455342500
746701773607749652730849986907860590

000000000000
1238898108130121436168819069

000000000000
1238898108130121436168819069

000000000000
1238898108130121436168819069

0
1

1

000000000000
1238897108130121436168819069

1238897108130121436168819069

00000000000
43638653321

0
1

0
1

0
1

1

25
00

5

2
0

2
0

2

4343663321
0000000000

4343663321
0000000000

4343663321
0000000000

4343663321

1
0

0
1

1

2225
2003

1
0

1

1
0

1
0

1

000
112

112
000

112

202313341830134915002073257925403288364031881372
4241144911528474324949830489086515797012145087525364841537778

000000000000
591480544353414441567501475663601394

590480544353414441567501474663601394
000000000000

590480544353414441567501474663601394
000000000000

590480544353414441567501474663601394

0
1

1
0

1

1
0

1
0

1

49141081281214201613
000000000000

0
1

1
0

1

11
00

1
0

1

0
1

1

2
0

2

16
00

00
15

15

0
1

1

0
1

1

000000000000
4812108128106201613

11

4812108127105201613
000000000000

1

481298127105201612

1

000000000000
10161042316347796

12
00

12
00

12

141231
000000

141231
000000

1

43

121

00000
12211

111
000

111

22

000000000000
810931714121586

710931714121586
000000000000

710931714121586

0
1

1

000
411

411
000

411

1
0

1

1

0
1

0
1

1

4913643252646
00000000000

499633142646
00000000000

499633142646
00000000000

499633142646

2
0

2
0

2

00
11

1

1
0

1

1
0

0
1

1

1
0

1

0
1

1
0

1

020000000000
3550663537145155101121826152

2
0

2
0

2

3447643337144155100121805751
000000000000

0000000
1121211

1

1111211

766214626577
000000000000

766214626577

264158303614014996114734943
000000000000

264158303614014996114734943

0
1

0
1

1

1121124

143
000

1
0

1
0

1

4
0

3
0

3

1
0

1

12
00

0
1

1

1
0

1

0
1

1

000000001000
221224319181214282413568645240229147

000000000000
13216425712814818625443748811413294

11
00

11

36476723284386132160242320
13116325712814818625343548811413294

1426966122426462

302538322929515496153120

32754161912111

53435238644582181160615939

396319955699136

834287101625896

12
00

12

111211
000000

111211
000000

111211

0000
3233

3233

0
1

0
1

1

0000
1111

1111

12
00

00
12

12

8339404740881341101271048046
000000000000

2
0

2

11
00

11

0
1

1

1

1
0

1

1
0

1

11
00

11

0
1

1

8239394740861311091261037946
000000000000

8239394740861311091261037946

111
000

111
000

111

112
000

00
12

12

1
0

1

1
0

0
1

1

51817620823182318164
51310512617101016113

1
0

1

1

371716713131

11111
00000

11111

1
0

1

776494521458605612915878782762777492
1377923531288982451322595287924006769195114740189281609415954

00
11

11
00

1

1

0
2

2
0

2

000
111

00
11

11

0
1

1

2

000000000000
18655581523232

11
00

11

000000000000
18655581423222

18655581323222

1

337210281271293274362419434369350292
000000010010

000000000
2714812105

2714812105

917696858673112122140948494
000000000000

917696858673112122140948494

000000000000
246132178185203193249294284270265198

246132178185203193249294284270265198

1
0

1
0

1

000000000000
67938610111251814

1
0

1

67938610111151814
000000000000

67938610111151814

1
0

0
1

1

00
21

2
0

2

1
0

1

806549561559738655749838761933914568
181732102367813221717

3576467311358488050353148
000000000000

122

3576467311258487850353146

596975757799936957825359
425660695388694344613434

21121221

7351135911781012

1010851051314413712

580300308314429392440545497659687351
254125127119182180213275202162254156

1026756597013161746617914780

127635281220676

8558117102617111510

591927353842675656884415

145778790123308110213621322084

000000000000
252727293218232633153635

252727293218232633153635

20192891830352217221610
8960735864821381101111209058

622534403745776679745343

12

7161198726221324215

157768361112591251059918217091
011110220110

001000102010
129617141904891727415114776

1

117536233742443495713313269

12888162447231418147

281411192111323125302215
000000000000

281411192111323125302215

274014269180721634313428167403041859150104813473727995991
000000000000

272814262180631633013410167163041059139104808472327955962
000000000000

272814262180631633013410167163041059139104808472327955962

1279131824811514429
000000000000

1279131824811514429

891161851088581613
000000000000

8742114664501412
100000000000

1

5531624414286

1

221152223666

27471424821
00000000000

27471424821

111281
000000

0000
1125

1125

131
000

131

11
00

11
00

11

535170446741565451797156
000000100100

000000000000
414146275432383939575539

414146275432383939575539

000000000000
12102417139171512211617

12102417139171512211617

1
0

1
0

1

0
1

1
0

1

1
0

0
1

1

620647195197453051486750466247434713898686216138
344644563453456279515421

11111
11001

1

1

440302343258367378266274237511435245
496940444512384543155933331231583130775673935391

21171410181814111426198

582211161311

172121145612212421171419

13111356254

8455747753310

1211132511

315338417441640449168215212287295256

77191928415411710899566043

11

12821215

64462236763

1

5243561136654

222363411167167193404402445195200122

112221132111

14423548457116

3162656644

3121112611

926748121025126

1235864739412630820026213911353

331433122311777174323632

3444644541251

234166216553193541363433

557391346527255164650030533734073078892

527888486886354141784684

1233715851619101310

31424633492

247121017823309142718

5581042141615217615

265758213088282410151813

128244229322631682411417621

11912515212211493618584113154102

3221134451

1261541711941025914

15670738110190140112122116125116

736101471876710

4244531

3718212526145758103504021

182416261748150015861644434509442170617532822

10380120707211813616214610810152

560336360293399726348301264580529310

8481012445511510

845383751114138

322727945977

000000000000
120262964162979976413051523150311781173725

120262964162979976413051523150311781173725

0000000000
156515714233

0000000000
156515714233

156515714233

0000000000
1212111112

00
11

11

0000000
1211112

1211112

11
00

1

1

2

473153604533
13091250132910791175124018322007215815771302948

801401771161819919626025917710161
000000000000

801401771161819919626025917710161

6236544413342
20121617275211225232514

93115153917131

5712101712533811

734777223432106116112664748
000000000000

734777223432106116112664748

113225651245
00000000000

113225651245

000000000000
744861026816113

744861026816113

000000000000
8137651515816171312

8137651515816171312

000000000000
130618570569127820037822718558

130618570569127820037822718558

252289281247232240288278315325204185
000000000000

401541521041141095968801245996

21213512914311813122921023520014589

1

1
0

1

185715171019272524176
000000000000

185715171019272524176

137181176191225194263393332170182106
700667669570762624710872888660668548

132823303932653343201719

401830242528596089484626

1102223211253

81156167881367136831079360101

25614613410816519510112298159198178

452518252121484647385420

57125811471413175

33122141

1

741248692

745683810191577

211221122

638456121217727

95539191681562

7779686911064776889515466

1312123

2

10109105591112556

000000000000
1742411918622212324245

1742411918622212324245

1

7473491442311
00000000000

1
0

1

0
1

1

53622711373
000000000

53622711373

00000000
11222411

11222411

0
1

1

0
1

1

1
0

1

00000000
39151621

39151621
00000000

39151621

2

00000010
15112258

00
22

22

13112246
00000000

13112246

254493105144126
000000000000

000000000000
254493105144126

254493105144126

5123146
0000000

5123146
0000000

5123146

00000020
11124471

132
000

132

00
11

11

11

0
1

1

1
0

1

1
0

1

111
000

1

1

1

12

1

21
00

21
00

21

11
00

11
00

11

1
0

0
1

1

000000000000
121566647618512111981576047

000000000000
121566647618512111981576047

121566647618512111981576047

000000000000
602011101131443442504224

602011101131443442504224
000000000000

602011101131443442504224

0000
1132

0
1

1

1122
0000

1122

7671507236085357219463273372605895721008
000010000000

7671507236085357119463273372605895721008
000000000000

11121

7661506236085357119463273362585895711008

0
1

1
0

1

0
1

1
0

1

1
0

0
1

1

000000000000
14413014193160106144172173223145124

0
1

1

14413014093160106144172173223145124
000000000000

14413014093160106144172173223145124

000000400000
284145137130165210246247214309214136

000000000000
332124202927364530462921

17810713918181623115

161314131618182714231816

000000000000
251124113110136183206201184263185115

251124113110136183206201184263185115

1
0

1

0103000000000
162198274183192278169174210170169110

000000000
151539121

000000000
121539121

121539121

0
3

3

000000000000
931730183652129941325910823

6313563471
0000000000

6313563471

871429153146126901255910822
000000000000

871429153146126901255910822

000000000000
691642291601532213066461095986

691642291601532212966461095986
000001000000

691642291601532202966461095986

1
0

1

00
14

1
0

1

4
0

1

3

11
00

0
1

1

1
0

1

52435511221
000000000

52435511221
000000000

52435511221

18
00

18
00

7

11
00

1

1

6333433732
0000000000

1
0

1
0

1

0
1

0
1

1

6233433722
0000000000

5233333421
0000000000

5233333421

000
111

111

0
3

3

1
0

1
0

0
1

1

31
00

0
2

0
2

2

00
11

00
11

11

213016271703155523301959200719872141216820791515
101066184910920115

000010000000
1110176821831519918

1110176721831519918
000000000000

1110176721831519918

0
1

0
1

1

11
00

1
0

1

1
0

1

010010000
132211131

0
2

2

0
1

1

0
1

1

1
0

1

1
0

1

0
1

1

0
1

1

121
000

121

0
1

1

12080955711667118152128126119109
000002001010

111
000

111

1
0

1

0
1

1

0
1

1

11915101161817201195
000000101000

1

97157105121514855

2231152524

1025868459853851038110710592
291811175124896212235

211222322

1

342424132518507157393721

381433152110252116444434

000000000000
713112661432247412

713112661432247412

2
0

0
2

2

000000000000
3215388117232138312834

3215388117232138312834
442312033267

5133513421

231123575181732252026

000000000000
293231302721505851444612

293231302721505851444612
000000000000

1

293231302721505751444612

11
00

1
0

1

0
1

1

536477510439447491426463470528485334
012321120214

2
0

2

1208311582778822114521011113355
000000000000

1208311582778822114521011113355

1

0
1

1

2
0

2

000000000000
9016116813211011864808313811670

9016116813211011864808313811670

000000000001
2616711266283624152522

756101395982

19117516525271961719

5610171411511717514
299216218211232278112199151260210183

29119319618220326480152110232202163

26225226823

1111010103253428313

25119104451536
011311100100

11
00

11

221
000

221

1
0

1

1

111
000

111

0
1

1

1114114
0000000

1114114

1
0

1

0
2

2

1
0

1

11
00

11

0
1

1

1
0

1

1
0

1

11

1
0

1

0
1

1

00
11

11

1
0

1

0
1

1

1
0

1

000000
131131

131131

0
1

1

0
1

1

000000000000
21222219138211417151615

1
0

1

000000100000
21222219117211417141515

1

22411114342

15201715104121313879

1

213127344

0
1

1

0
1

1

1
0

1

1
0

1

010000000
132421121

00
31

31

1
0

1

1
0

1

1
0

1

0
1

1

21

0
1

1

0
1

1

0
1

1

1
0

1

1
0

1

2111230121110
10498148058431418114511531024121510641082750

1
0

1

1
0

1

2111
0000

2111

0
2

2

0
1

1

1
0

1

1
0

1

0
1

1

0
3

1

2

0
1

1

0
1

1

00
11

1

1

0
1

1

0
1

1

0
1

1

1
0

1

1
0

1

1
0

1

0
3

3

0
1

1

1
0

1

1
0

1

38191641612523935403015
000000000000

38191641612523935403015

0
1

1

000
113

113

1

298420396599669781534445586429368350
000000000000

298420396599668781534445586429368350

1

0
1

1

0
2

2

327
0

327

0
1

1

20
21

1

1
0

1

0
1

1

152421523892087
000000000000

152421523892087

00000
21112

11

1112

000
111

111

1
0

1

611722123419192413424651
000000001100

611320113218162010354447

42121342624

0
1

1

1
0

1

0
1

1

0
1

1

000100000000
61611156918921191913

61611146918921191913

0
1

1

0
1

1

0
1

1

30019223490157143187219215286378133
000000000000

30019223490157143187219215286378133

0
1

1

322145114110189167334272329213229177
30312210494172151310254296199204161

12172109121971681313

3135425813592

45514234131

0000
1141

1141
0000

1141

100000000200
684639386363647557925678

000000000000
205522610231015918

205522610231015918

000000000000
332318193744404335604047

332318193744404335604047

4768612454727
000000000000

4768612454727

000000000000
10111091811048856

10111091811048856

000000000000
1571781618143112112212

11
00

11

1226371051997106
1551671518143011112212

15132321322

3253566911104

0
1

1

1111
0000

1111

1
0

0
1

1

12
01

0
1

1

1
0

1

000000000000
1424657121361741

000000000000
1424657121361741

1424657121361741

1
0

1
0

1

11354532010118218
001000000100

0
1

1

1
0

1

0
1

1

0
1

1

0
1

1

1022453208115218
000000000000

1022453208115218

0
1

1

1
0

1

0
1

1

000000000000
413861317542481

1
0

1

000000000000
411751317542481

411751317542481

11
00

11

0
1

1

1311
0300

11
00

11

0
1

1

1
0

0
1

1

002020030010
166117131012914111214

00
12

12

0
1

1

1
0

1

1
0

1

0
3

3

1
0

1

1468510101261151014
000000000000

1468510101261151014

1
0

1

1
0

1

0
1

1

0
1

1

1
0

1

1
0

1

000000000000
8410131147105676

000000000000
8410131147105676

8410131147105676

18074685412684458086164143110
201111657140

1
0

1

000000000000
1256676317416116

1256676317416116

16669614711877365875147128103
001110102100

322471211161518594325

12212217221

522331521

136561382941159

1153944378056223043697667

00
11

1
0

1

0
1

1

186515171675128717581813231123192716220922001516
460014055320

000000000000
454137242362881211161019751

000000000000
35373015184368109103939245

35373015184368109103939245

1046952201213856
000000000000

1046952201213856

117
00

117

0
1

1
0

1

11
00

00
11

11

4082622801592042537808331041572482211
001202220164

311185199107132177697744953426373119
000000000000

311185199107132177697744953426373119

1
0

1

6
0

6

00
11

11

0
1

1

2
0

2

9776795072747887871459788
000000000200

7458674252626379711297974

2318128201115816141814

1

1
0

1

000000000000
158205284145247401170188197259293335

143195268137235385155175176236279323
100101233410

136189266130223379140167164210266314

6626125135922129

0
1

1

1
0

1

000000
211121

1

211111

113265464
000000000

113264464

1

1
0

1

0
1

1

0
1

1

417123436825
000000000000

417123436825

00
11

11

00
11

11

000
111

111

1
0

1

654565748754
000000000000

654565748754

0
1

1

0
1

1

0000
1112

1112

11
01

1

1
0

1

1
0

0
1

1

1
0

1
0

1

233165240209259162342340443356376119
000000000000

11
00

11

233165240209258161342340443356376119
000000000000

233165240209258161342340443356376119

13
00

0
3

3

1
0

1

111
000

0
1

1

0
1

1

0
1

1

21
00

21
00

2

1

00
11

00
11

11

0
1

1
0

1

21217514128194042
00000000000

21217514128194042
00000000000

21217514128194042

66221871953
0000000000

111
000

111

0
1

1

1
0

1

0
1

1

0
1

1

2222551721

1
0

1

1112
0000

1112

1
0

1

00000
31111

2111

11

0000
11231

0000
11231

11231

00
11

10
11

1

3
0

3

0
1

1
0

1

100
111

1
0

1

0
1

1

0
1

1
0

1

1
0

0
1

1

7841495354551378214218511342
000000000000

107644112920112399
000000000000

107644112920112399

68344349505412573122737433
000000000000

68344349505412573122737433

000000002000
63104434391153

000000
111141

111141

000000000000
5284412121052

5284412121052

1
0

1

1

111

0
1

1

1

0
1

1

1
0

0
1

1

000000000000
208694626399193754442026

1
0

1

000000000000
198289585893153335432024

198289585893153335432024

0000000000
14545644192

14545644192

0
1

0
1

1

000000000000
103735713892257

000000000000
103735713892257

103735713892257

211

1
0

0
1

1

121
000

000
111

11

1

1
0

1

121010000013
733607564538777628485443394483643595

0
1

1

0
4

4

000000000000
195228246184193182204226185232234101

195228246184193182204226185232234101

0
1

1

0
2

2

000000
811451

811451

0
1

1

20261910171214202512227
000000000000

20261910171214202512227

508351294343566434263197183230386482
132146322631

16252911109162518162215

491323263331552419244170163208361466

1

11
00

11
00

11

1
0

1
0

1

301816272518526660364045
000000000000

000000000000
301816272518526660364045

301816272518526660364045

2
0

0
2

2

72315425535
00000000000

72315425535
00000000000

72315425535

1
0

0
1

1

112014206010
53114616101113382

1
0

1

00
11

1

1

32645118114372
000000000000

32645118114372

1

1
0

1

0
1

1

1
0

1

0
1

1

431819191619554381544623
000000000000

000000000000
411816191619523670534623

411816191619523670534623

00
31

31

23610
0000

23610

1
0

1

0
1

1

000000000000
8234322951461339881606147

8234322951461339881606147
000000000000

8234322951461339881606147

1131
0000

1
0

1

1
0

1

0
1

1

0
1

1

0
1

1

0
1

1

327621462468199726302226297631713702313030672554
172566829383340595215116867

000311001200
4527523429104

000000000000
442441341794

442441341794

0
1

1

1

0
1

1
0

1

411448246020203130403038
000000000000

000000000000
411431246020203130403038

1

411431246020193130403038

0
17

17

000000000000
139181163161208154262223276176166131

0
1

1

000000000000
789869759477143114170978546

789869759477143114170978546

557284454972
000000000000

557284454972

000000000000
5678878410672115104102707483

5678878410672115104102707483

00
11

00
11

11

11211
00000

00000
11211

11211

265196192141213183358390506296236201
643205133304

13185997094102801161219992105
131310556310

32221226821

7336871013411420

146201251051826101512

1047073497482557376566871

21423531121

000000000000
46751023237411355

46751023237411355

10292101512716166
000000000000

10292101512716166

2
0

2

000000000000
3423339125327

3423339125327

000000000000
31193156811553

31193156811553

1
0

1

104756053915721419631214511369
644336255540176159265786535

2111231041

41426242331

2

127339214113151217

241122

1121111112

111341

311384

151612222015172520282313

49334811561212
000000000000

49334811561212

0
1

1
0

1

563967464057645557544735
004000200101

502130342240474135413818
563350433251575051484426

111214211

412129798513657

141

3211

613386556538
00000000000

613386556538

2211
0000

00
11

11

1
0

1

00
21

21

100000000711
21690136112131123451492624225261172

13113191922214
1478131310276273449173512

124526511172112296

121044256237406352

5862988411049
000000000000

231

566298841739

181320111011141113141518
000000000000

181320111011141113141518

000000000000
18581381611151310616

18581381611151310616

00000000000
28663829575

28663829575

110517737506412015612010217089
000000000000

110517737506412015612010217089

1
0

1

1
0

1

4169293511143118201822
000000000000

1

4169293511143117201822

000
7405

7405

1143349333625607958978515
000000000000

1
0

1

000000000000
1143349333625607858978515

1143349333625607858978515

000000000000
11648643834921699815710013233

000000000000
11648643834921699815710013233

11648643834921699815710013233

000000000000
16795546139171716

000000000000
16795546139171716

16795546139171716

6384531107163367469804251
000000000000

000000000000
6384531107163367469804251

6384531107163367469804251

00000000
115106111

115106111
00000000

115106111

432223303123385136535835
000000000000

000000000000
432223303123385136535835

432223303123385136535835

996532668417663574615706836933866876
202338333640212020713722

1
0

1

00000000000
84793361210139

84793361210139

000000000000
521412765385

521412765385

00000000000
114213373564

114213373564

0
1

1

000000000000
1514911716181614111521

1514911716181614111521

7448532952426361801319471
000000000000

7448532952426361801319471

00000000000
22213228211

22213228111

1

343124241929331931493134
000000000000

343124241929331931493134

2269512876110145117132225107157319
000000000000

2269512876110145117132225107157319

1
0

1

523435549754
000000000000

523435549754

000001020000
13493121014212081012

2112421

11352951312176810

1332131221

000000411000
14556179131012141112

145561799911141112

21
11

1

000000000000
242315252219161827201230

242315252219161827201230

000000000000
65108354811485

1

65108354711485

0000
23110

11

239

3061111047751
290891034417863138185182202186176

28588944217762127180173192180175

213111231

1162156310972
000000000000

1162156310972

11
00

11

22
00

22

000000000000
431211363521

431211363521

892939414161294147857556
000000000000

892939414161294147857556

155954618212271810
000000000000

155954618212271810

0000000000
2132352232

2132352232

0
1

1

113
000

1

112

13414219896139108941169116916382
000000000000

13414219896139108941169116916382

000000000000
1266763981509614136

1266763981509614136
000000000000

1266763981509614136

111
000

0
1

1

00
11

11

525399323440630464220267280325411443
000000000000

000000000000
525399323440630464220267280325411443

525399323440630464220267280325411443

11
00

00
11

11

0
1

0
1

1

4
0

0
4

4

6328481626201933408010253
000000000000

6328481626201933408010253
000000000000

6328481626201933408010253

1
0

1
0

1

2
0

0
2

2

01
11

0
1

1

1
0

1
0

1

000000000000
271425112116475370383118

000200402000
271425112116475370383118

25102171812272320312714

244234163048744

382736222934555158434039
000000001000

332030112126523946243632
332430132427534449323935

4211141423

21241

5369572781114
000000000000

5369572781114

182243353284279241284237320174167133
2340202041110

422421415373
28613812121028911814329

11241371111107132

132737487771034124

722939213253645456854732
211172514831113118

2123747566

411427182634313720481710

83311218101819138

0
1

1

211
000

211

300413524526
8020429725323317511694141648792

6019729024521916410285117517280

10422865216534

7352524543102

0000
1112

1112
0000

1112

110010000000
18211211548807012599113222183184

000000000000
1469210231504110580102191148153

1469210231504110580102191148153

212211111822
351913172929201911313531

28254142011115132222

1

51661114887410117

000000
111114

000000
111114

111114

2
0

1
0

1

0
1

1

4711481321614
00000000000

4711481321614
00000000000

4711481321614

0
1

0
1

0
1

1

175021303814273112241727610344128811525415719204371990713567
000000002000

1
0

0
1

1

0
1

0
1

1

000000000000
253487444323363519257247285149188150

253487444323363519257247285149188150
17045738828830646816715819983111101

833056355751908986667749

000000000000
17249125511382910901169129825126241500615432202881971913417

515547375223434685494286535958076308678566295786
17249125511382910901169129825126241500615432202881971913417

2758782820548683522678112385931003343750

8157408726488125045207328331039923734

411523962447208723671482151419191823460640532153

659642744388711486784914996810738723

8117529034528155807689751002990937948

410354305237257210290452541438414476

16221121135412131650117614591707158113191549959

473704782675694284437535592551523539

431323379307374295815842897650610349

010000000000
732537155536266158544447

258341251120662
000000000000

1
0

1

258341251120652
000000000000

258341251120652

1
0

1

0
1

1
0

1

2112112
0000000

111112
000000

111112

00
11

11

0
1

1

000000000000
68152585023143828463544

000000000000
68152485023143828463544

68152485023143828463544

1
0

1

0
1

1
0

1

0000
1111

1
0

1

111
000

111

11112
00000

11112

1
0

1
0

1

00000
12741

0
1

1

12641
00000

12641

121113121
011013120

1
0

1

1
0

1

1

0
1

1

11
00

0
1

1

0
1

1
0

1

000
31012

31012
000

000
31012

31012

169282373451524
71769669852577548495210841201728727513

000000
123311

1
0

1

1
0

1

23211
00000

23211

7243899181362
00000000000

0
2

2

00000000000
7243799161262

7243799161262

0
1

1

1
0

1

00
12

0
1

1

0
1

1

1
0

1

11221811
0000000

2
0

2

1
0

1

0000000
11221311

11221311

0
2

2

0000000000
4455595771

0000000000
4354595771

4354595771

0
1

1

0
1

1

0
1

0
1

1

11315
00000

0000
1315

1315

0
1

1

1
0

1

152111904611675157163195144160120
394522105014587875364550

0
2

2

1913981515282321272315
111020030111

1510645618181623199

3224891025335

00000000000
5431551319753

5431551319753

000000000000
6155529514954

6155529514954

142724925713146
000000000000

142724925713146

391342325147
00000000000

391342325147

000000000000
117154523551764

117154523551764

000000000000
64338197106109

1212412

5233719510297

0
1

1

492613102624412244284522
000000000000

492613102624412244284522

11
00

11
00

11

0
1

1

000000000000
322131363432

322131363432
000000000000

322131363432

0
1

1

0
25

25

0
1

0
1

1

000000000000
565159411617183855775930

000000000000
565159411617183855775930

565159411617183855775930

0
1

1
0

1

21212818112
000000000

000000000
21212818112

21212818112

151561251511
000000000000

000000000000
151561251511

151561251511

182418124019231411112225
000000000000

00
11

11

000002101010
182418124019221411112125

182418124017211410112025

2107311103316
0000000000

2107311103316
0000000000

2107311103316

010100000000
1579327912111

1478327912111

71
00

71
00

71

0000010
43211286

431212
00000

431212

1
0

1

1164
0000

1164

000000000000
26146611110732274

1
0

1

26146611110632274
000000000000

26146611110632274

000
344

00
14

1

4

2
0

1

1

0
4

4

00
11

0
1

1

1

0
3

0
3

3

0
3

0
3

3

143162112
110010000

11
00

11

0
1

1

1

3213112
0000000

3213112

0
1

1

7122882
0000000

7122882
0000000

511661

212221

0
1

0
1

1

473219314444598152633634
000000000000

1
0

1

000000000000
473219314444598152623634

473219314444598152623634

1
0

1
0

1

1
0

0
1

1

1
0

1
0

1

000
113

0
1

1

3
0

3

1

00000000
3813181212

4
0

4

00000000
3413181212

3413181212

321
000

1
0

1

0
1

1

00
31

31

000000000000
384646401234613453242121

000000000000
384646401234613453242121

384646401234613453242121

1
0

1
0

1

3823341013461
11000144151

0
1

1

12
00

12

4
0

4

000000000
2212315929

2212315929

1
0

1

111
000

111

21
20

1
0

1

0
1

1
0

1

346331370313487262511605612324375256
000600213000

0
1

1

000000000000
307292333286446235447520523261335220

307292333286446235447520523261335220

0
1

1

0
1

1

393935204126628385634035
000000000000

393935204126628385634035

1
0

1

1
0

1

0
1

1

1
0

1

100
111

11

11
00

11
00

11

221
000

11
00

11

1
0

1

00
11

11

00000
11112

00000
11111

11
00

11

111
000

111

0
1

1
0

1

000000000000
1910185103312425171610

1
0

0
1

1

1
0

0
1

1

0
1

0
1

1

0
1

1
0

1

000000000000
1810165103312425161610

000000000000
176937226212113106

176937226212113106

000000000000
147231534364

147231534364

11
00

11
00

0
1

1

1

73316397882026957
011011100001

00
31

31
00

31

14129101
0000000

0000000
14129101

14129101

000000000000
630631432914722

1
0

0
1

1

000000000000
630631432913722

00000000
2822811673

2822811673

000000000000
624333136421

624333136421

0
1

1

00
11

00
11

11

13414322223
00000000000

13414322223
00000000000

13414322223
00000000000

13414322223

2
0

2
0

2

21112171212283763643
000000000000

0
2

2
0

1

1
0

1

000000000000
2119171212283663443

2119171212283663443
000000000000

0
1

1

2118171212283663443
000000000000

2118171212283663443

12
00

12
00

00
12

2

1

1
0

1
0

1
0

1

000000000000
220951198311586110152142214198185

000000000000
21691113761078198123114199192182

00
11

00
11

11

31215211
00000000

31215211
00000000

31215211

0
2

0
2

2

0
2

2
0

2

121
000

21
00

21

1
0

1

0000000
3112112

0000000
3112112

3112112

000
111

111
000

111

000000000000
2018410771997196120104190182162

522015201314212721362716
2018310769997194118101189181160

131117494791517156

1

11

12

17434566245811

1054253336031506148109112114

146188101510168221911

2

1

122

0000000
2223112

2223112

1
0

1

8354531137817
000000000000

000000000000
8354531137817

8354531137817

000000000000
214433813101463

214433812101462
000000000000

000000000100
214433812101462

1

214433811101362

11
00

1
0

1

0
1

1

232352416181
0000000000

0000000000
232352416181

232352416181
0000000000

232352416181

1512241011
000000000

000000000
111114911

111114911
000000000

111114911

4000
4111

1
0

1

0
1

1

1
0

1

31616311
00000000

0000000
3145311

3145311
0000000

3145311
0011101

313421

1
0

1
0

0
1

1

21
00

00
21

21
00

21

1912212619179168121510
000000000000

1912212619179168121510
000000010000

1911192418167106121410
000000000000

000000000000
1911192418167106121410

1911192418157106121410

1

1112
0000

0000
1112

1112

0
1

1
0

1

1
0

0
1

1

211321
100000

00
11

11

1132
0000

1132

0
1

1
0

1
0

1
0

1

254622162655200427921877191221882118278926602139
000000000000

211
000

00
11

11
00

11

2
0

2

254622122651200127881874190921862116278926602139
125182133332

23113322
00000000

00000000
23113322

23113322

000000000000
6857141018582857561531586551635629732

000000000000
6857141018582857561531586551635629732

6857141018582857561531586551635629732

0
1

1

000000000000
719441498371478393582677628771736668

2
0

2

719441497371478393580676626771736668
000000000000

719441497371478393580676626771736668

1

00
11

11

1
0

1

10
21

11
00

1

1

1

1141105511291045144191779491792913781292734
000000000000

1141105511291045144191779491792913781292734
000000000000

1141105511291045144191779491792913781292734

00000000
24243222

0
1

1

11
00

00
11

11

13233222
00021021

121221
000000

121221

3
0

3

10989431065805106584717151828198614301130897
234012101217131

0
1

0
1

1
0

1

1

0
1

0
1

1
0

1

0
1

1
0

1
0

1

000000000000
4814746201954311

416726181951111
000000000000

000000000000
416723181951111

416723181951111

0
3

3

7000
7112

1
0

1

1

2

2
0

0
2

2

0
1

1

00
31

0
2

2

11
00

11

1
0

1

2
0

2

111
000

11
01

1

0
1

1

00
11

11
00

1
0

1

0
1

1

92891187187561331772411528884
210232014121932528411

1
0

0
1

1

0000
1211

1
0

1

0
1

1

111
000

111

1111
0010

00
11

11

0
1

1

1
0

1
0

1

1
0

0
1

1

000
121

0
1

1

1
0

1

00
11

11

114165201

111
000

111
000

111

13111441
00000110

1

0
1

1

311133

00
11

10
11

1

1
0

1

11111
00000

11
00

11

0
1

1

0
1

1

1

100
231

1
0

1

1
0

1

10
11

1

0
1

1

10000
11542

0000
1542

1542

000
211

1
0

1

000
111

1

11

1111112
0000000

1

111112
000000

111112

00
11

1
0

1

1

0
1

1
0

1

1
0

1
0

1

00000
12152

41
00

41

0
1

1

111

11
00

11

3112
0000

0
1

1

1

11
00

11

2
0

2

0
1

1

1
0

1
0

1

1
0

1

1
0

1
0

1

392839122815385667613444
000000100000

1
0

1

1133385
0000000

1133385

1

1
0

1

0
1

1

1
0

1

382638122812345358563343
000000000000

382638122812345358563343

0
1

0
1

1

1
0

1
0

1

0
1

1
0

1

11

0
1

1
0

1

02
12

1

211001010320
1014161510421131515116

0
1

1

0
1

1

1
0

1

81214151031912151296
000000000000

81214151031912151296

0
1

1

000000000000
811912108188241394

811912108188241394
000000000000

811912108188241394

131
011

0
1

1

0
1

1

0
1

1

4

010
111

0
1

1

0
1

1

0
1

1

11
01

1
0

1

000000
212111

0
1

1

00
11

1

1

000
121

1

111

0
1

1

1
0

0
1

1

22
00

1
0

1

0
1

1

11
00

11

3

0
1

0
1

1

0
1

0
1

1

000
111

1
0

1

1

0
1

1

1
0

1
0

1

2993598224036402514
000000000000

000000000000
2993598224036402514

2993598224036402514

0
1

0
1

1

1
0

1

0000100
2111212

111

1
0

1

0
1

1

0
1

1

0
1

1

11
00

11

825560526132898893595938
000000000000

0
1

0
1

1

1
0

0
1

1

000000000000
825560526132898892585938

111
000

111

0
1

1

825560516030898892585938
139111611227163217138

39161912151240463722269

112

303030233315222623191821

1

701580633500735598117111431150870724550
5257524591032

0
1

1

000000000000
176179171165231173216221253232179124

000000000000
176179171165231173216221253232179124

176179171165231173216221253232179124

00000
11114

00000
11114

11114

1
0

1
0

1

11
00

11
00

11

612121
000000

612121
000000

612121

0
1

1

1
0

1
0

1

000000000000
401321370279432341755770703494429359

000000000000
1458978568678391417359193172146

1458978568678391417359193172146

000000000000
4842523453326293106856038

4842523453326293106856038

000000000000
208190240189293231302260238216197175

208190240189293231302260238216197175

113
000

000
113

113

0
1

1

0
1

1
0

1

1
0

1
0

1

000000000000
118758247598019014117313310963

118758247598019014117313310963
000000000000

81545534397310290112877144

37212713207885161463819

0
1

1
0

1

00000
11441

11441
00000

11441

1
0

1

0
1

0
1

1

0
1

1
0

1

0
2

2
0

2

00
11

0
1

1
0

1

0
1

1

002000001000
96110122776660129158195164139130

0
1

1

23123324511
21000020000

1
0

1

111322211
000000000

111322211

1
0

1

00000
11112

11112

2111
0000

2111
0000

2111

1
0

1

0
1

1
0

1

11532561918004
9299116715854110132149154132127

000000000000
717998554138929811913410692

717998554138929811913410692

111
000

111

201913131411121411202631
000000000000

201913131411121411202631

212
010

00
22

22

1
0

1

31293
00023

0000
3127

3127

122

1131
0000

0000
1131

1131

2

1212121011261
0000000000

1

1212121010261

0000
1122

1122
0000

1122

12
00

12
00

12

1
0

1
0

1

11
01

1
0

1

010000
111221

111

000
211

211

1123261
0000000

0000000
1123261

1123261
0000000

1123261

100798375896711715515315310473
000000000000

000000000000
813729210236815

000000000000
81272629236815

81272629236815

13
00

13

1
0

1

000010000010
9266767380651071531501479658

355435111846413
000000000000

1111121411

3

22421361124

21127212

000
111

1

11

000000010000
462419272420244228423516

111

11

18511371118151319104

271972317962614222512

495465647752
393550364735657965895437

33277464

131428142610242431341715

191214161520284423422820

000000000000
422545714101252

422545714101252

000000221020
151117131316304740251015

0000000000
2442187724

0000000000
2442187724

2442187724

0
1

0
1

1

00
11

00
11

11

1
0

1
0

1

158976101023172357
000000000000

158976101023172357
122443481361724

431251211

3222342510422

1216991
0000000

0000000
1216991

1216991

0
1

1
0

1

21532
00000

21532
21422

11

0
1

1
0

1

0000000000
1212211211

1212211211
0000000000

1212211211

11
00

00
11

11

00000254900
6610961013273724

0
1

0
1

1

000
123

1
0

1

23
00

23

1
0

0
1

1

0
1

1
0

1

1
0

1

1
0

1
0

1

0
1

0
1

1

1
0

0
1

1

00000000000
1338211151314

11231461
00000000

11231461

25111174
0000000

25111174

1
0

1

1
0

1

00000000
11212345

00000000
11212345

11212345

22214249

000000000000
4657578384738370116594535

1
0

1
0

1

203521565251564265323020
000000000000

0
2

2
0

2

000000000000
203521565251544265323020

203521565150534264322920
000000000000

203521565150534264322920

11111
00000

1

111

1

000000000000
1658584105517106

1
0

0
1

1

000000000000
1648584105517106

1648584105517106
000000000000

1

1

1647584105517105

24435981021

000000000000
10132117211281433848

221
000

221
000

1

1

21

1
0

1
0

1

00
11

11
00

11

0
1

0
1

1

151711793620113
000000000000

151711793620113
000000000000

151711793620113

0
2

2
0

2

2112
0000

0000
2112

2112

3
0

3
0

3

111
000

111
000

111

000000000000
8333535811715

000000000000
8333535811715

8333535811715

000000
231131

00
11

11
00

11

000
112

000
112

112

0000
2111

0000
2111

2111

100000202110
105158668555275056388786999811841037701

1
0

1
0

1
0

1

101856665653272353983979993211431001668
312131026200

1

000000000000
71414437464573721211399153

71414437464573711211399153
000000000100

1

71414437464573701211389153

0
1

1

1
0

0
1

1

0
1

0
1

1

0000
1111

0000
1111

1111

000000000000
361942132818464990354422

000000000000
361942132718464990354422

361942132718464990354422

0
1

1

0
1

1
0

1

11
00

11
00

11

000000000000
11275735769651211371471019292

11275735769651211371471019292
67785127711641

5146224141261675

321117161712402839131921

331825182824364653243535

14151110654812221011

221073118203626201719

1
0

1

1211221
0000000

0000000
1211221

1211221

0
1

1
0

1

1
0

1
0

1

111
000

000
111

111

0
1

1
0

1

0
1

1
0

1

279189193224240173167173149316307145
000000000000

0
1

1

000000000000
279189193223240173167173149316307145

279189193223240173167173149316307145

1111313211
0000000000

0
1

1

1111312211
0000000000

1113122

11

1

000000000000
12667774968571371019414910567

000
211

1

21

0331501023431
118647447635612988891349462

658159221521219805820

535056373641986577503341

3

632251812515115

115255968231429720
001000111000

000
111

111

105245967221328719
000000010000

105245967211328719

0
1

0
1

1

11
00

10
11

1

1

100000001021
376166194144257173282239302366352267

000000000000
355157179140250167263223293342329260

355157179140250167263223293342329260

000000000000
209154761916824216

209154761916824216

31132092121415353383432
201000010000

000000000
413131182

412131182
000000000

412131182

1

0
1

1
0

1

000000000000
226871617302722322529

226871617302722322529
000000000000

226871617302722322529

1
0

0
1

1

00000000000
6624491728311

0
1

1

00000000000
6524491728311

6524491728311

1
0

0
1

1

00
21

21
00

21

00
22

20
22

2

1

212
000

212
000

1

211

000
111

0
1

0
1

1

00
11

11

1466435116211
012401353000

21
00

00
21

21

211

0
1

0
1

1

1
0

0
1

1

12112
00000

12112
00000

12112

000000
112111

112111
000000

11211

1

1
0

1
0

1

11
00

11

1
0

0
1

1

0
1

0
1

1

1
0

0
1

1

0
1

1

1
0

1
0

1

0000
1211

211
000

000
211

211

1

0
1

1
0

0
1

1

11
00

1
0

0
1

1

0
1

1

221241
021240

0
1

1
0

1

0
2

0
2

2

1

59961025017843474960879183
000000010000

58961024917843474860879183
000000101000

58961024917843464859879183
000000000000

0
1

1

0
1

1

58961004917643464859879183
000000000000

58961004917643464859879183

1
0

1

0
1

1

0
1

1
0

0
1

1

1
0

1
0

1
0

1

78296910521022101257963170481410218501217
0030301519100

309416367539597372323357407190190374
023015013000

00000000000
11016210513173334

00000000000
11016210513173334

1815210513173334
1916210513173334

1

1

0
1

1

10335
11335

1

000000000000
1111810811101511615

000000000
364142961

364142961
000000000

364142961

1456564152
0022002120

1

1111

13245532

00000000000
17811222413

17811222413
00000000000

1

1111

66113

1211211

1

1
0

1

306392327524578350300324354184186365
000101010000

14830224444750525991961498687240
000000000000

083120101013
14830224444750525991961498687240

22213111

973646334165262724595173

11019161498263856

11

1213

2

111

2715488324362144211724813107

12321611

2236745831311

1

20928364753426112281745

12863665560741701611436574107
001110000001

111

0
2

2

000
311

311

82343217283612110293394561
12657614755681661551386572102

1211

11

517242173119549

19161619191617121561520

1

145371109966611

2

1122

11

6111417

0000000000
1113241123

1113241123

1124222331
0000000000

1124222331

010001110000
302717211316396662332518

88815810829
4451275601

1

1

1

1

1

1

4413418

1

164315411161315146
000000000000

164315411161315146

1412412741839371292
000000000000

1412412741839371292

22214421
00000000

22214421

1

1

4212261
0000000

2000000
4212261

22111

1

224

010000000000
473545677482408200303325362827659842

1963684373022093869106150537411490
031011111100

0
1

1

000000000000
718610681321451366

718610681321451366
14113291325404

1

1121211

1

12

12

11

1111

13322321

41016241114431

1

000000000000
189347430292201295584104523405484

189347430292201295584104523405484
101000002000

11

11

1

1

22221

1

1

11

1

11

11

11

1

3481245341343823

15033241128018723527495484364460

13376323221

1

277175240180199162233219210290248352
000000000000

000212
111223

11111
00000

11111

276175239180198162231217206290248352
02538113912140

4964865264563433338286152
276172226171188155208203173289243348

45246551161

1

2112211632

1236111

1

1231232321

276113

131310613433

1111151

31545396645

633758363012564938602225

1

13811537104914145

211

127222361

32242221154

111112133

11

43426921

12446534967586575466891149

122222428

114121121

186261052114
0000000000

121241811

1

652463133

1
0

1
0

1

112
000

000
112

1

12

11151

33131715111
0000000000

33131711111
33131715111

2

2

1
0

0
1

1

1

11531
00000

00
11

11
00

10
11

1

1143
0000

11
00

11

0100
1132

132

000000023010
8252719191221516641219

000000
211323

113
000

113
000

000
113

12

11

0
1

0
1

1
0

1

00
12

12
00

00
12

12

000
111

000
111

111
000

111

351167571918541
010012001000

0000000
2112114

2112114
0000000

00
11

1

1

10001
21212

1

11

1

1

1

1
0

1

0
1

1

0
1

0
1

0
1

1

00
11

00
11

0
1

1

0
1

1

1944141114431
00000000000

131
000

131
000

131

00
11

00
11

1

1

1
0

0
1

1

00000
11132

020
121

1

1

1111

1

6342106
000000

000000
6342106

6342106

1111121
0000000

0000000
1111121

1111121

00
12

12
00

12
00

11

1

00000000
11114211

10001100
11114211

1113111

41673215111335144
000000000000

000000
113411

000000
113411

000000
113411

113411

163121106522
00000000000

2
0

1
2

1

00000000000
163121104522

00000000000
163121104522

1

16312184522

1

11
00

1

1
0

1
0

1

000000000
31111229111

31111229111
000000000

0000000
311129111

311129111

111
000

1

11

1
0

1
0

1
0

1

71111
00000

00000
71111

71111
00000

6

1

1

111

1
0

1
0

0
1

1

123451

000000000000
128108551225111

1
0

0
1

0
1

1

128108551224111
000000000000

000000000000
128108551224111

000000000000
128108551224111

128108551224111

1
0

1
0

1
0

1

1

0
1

1
0

1
0

0
1

1

000000000000
1443141416183435619956054

1
0

0
1

0
1

1

1443141416083435619956054
000000000000

030000000000
1443141416083435619956054

000000000000
1442841416083435619956054

000000000000
1442841416083435619956054

1442841416083435619956054

0000000000
120355491341

0
19

19

111321

00000000
124546132

00000000
124546132

124546132
00000000

124546132
00000000

124546132

11

010000000000
13169510112191521919

1121
0000

0000
1121

0000
1121

0000
1121

1121

13159510111191419819
000000000000

000000000000
13149510111191418819

13149510111191418819
000000000000

13149510111191418819
000000000000

13149510111191418819

00
11

00
11

11
00

11

20111412610113410316820526019719397
000000000000

2

197109122951229416019124319518089
000000000000

11
00

0
1

1

1
0

1

197109121951229416019124119518089
000000000000

197109121951229416019124119518089
000001100000

000000000000
243112254372

243112254372

1

1112312
0000000

1112312

1310001461450
763857304127596974856331

272321413244

1111

1489433611171357

16

157115421771313135

1

1

512232123442

113214143

74334135516121

1151132

19927102111202222241715

11967606379629811716010611053
302100021002

1

112

1

5423202143257785130586722

212241

614133383435132523443526

12

1111431252

1111

1111

0
1

1
0

1

0010000000
33446611112

0
1

0
1

1

000
112

0
1

1

00
12

12
00

12

21123371
00000220

0020
2131

201
211

1

0
1

1

000000
111312

11
00

11

001000
111211

11

1

11

1

000000
112121

0000
1212

0
1

1

1
0

1

00
22

2

2

11
00

1
0

1

1
0

1

111121
000000

111121
000000

1

111111

000000000000
4243852841126

11
00

11

000000000000
4243851541125

4243851541125
000000000000

4243851541125

21

11
00

0
1

1

1
0

1
0

0
1

1

31112

000000101010
20310914086136124220219251171166169

194521010122313131316
100012041040

100000000200
18452981017612916

00
11

0
1

1

1
0

1

2
0

0
2

2

1
0

0
1

1

211
000

211

000
113

000
113

113

153419761559916
000000000000

153419761559916
000000000000

153419761559916

2261
0000

2261
0000

0000
2261

2261

000001040000
9657568121013103

0
1

0
1

0
1

1

000000000000
964755881013103

964755881013103
000000000000

000000000000
664755881013103

664755881013103

0
3

3

127628946896610810613096103116
000100000000

0
1

0
1

0
1

1

000000000
122111212

1221112
0000000

0000000
1221112

1221112

1
0

0
1

1

00
11

11
00

11

10347773365547376795972102
000000000000

000000000000
10347773365547376795972102

300101021450
10347773365547376795972102

731930133834322820233268

272847192719414658323534

231410102311343049373012
000000001000

1
0

0
1

1

000000000000
221310102311292948373012

0
1

1

000000000000
221310102311292948372912

221310102311292948372912

151
000

151
000

151

000000000000
483739303141907696493934

111
000

111

000000000000
332326212122574655202023

000000000000
332326212122574655202023

000000000000
332326212022574655202023

332326212022574655202023

0
1

1

000000000000
2232255920115

2232255920115
000000000000

2232255920115
000000000000

2232255920115

121210781427202128186
001000000010

000000000000
646645148111262

000000000000
646645148111262

646645148111262

1

68314913121014114
000000000000

68314913121014114
000000000000

68314913121014114

0
1

1
0

1

2111121
0000000

0
2

0
1

1
0

1

1
0

0
1

1

000
111

111
000

111

000
121

21
00

21
00

21

1
0

0
1

1

0
1

0
1

1
0

1

3531221
0100000

1

000000
232111

000000
232111

232111
000000

2

212111

0000
1112

0000
1112

12
00

12

11
00

11

243442702559383227704025251431293123851731467947228862842273738924081850261102352645338320047637
2653463753693604295538681278338235353

2012515510697108172302567382140

000000000000
243427932559108127701629251403483123558631463752228835982273412224076811261082892645181820046040

280122901934609328013759638319465795546689474264002732222673
243427932559108127701629251403483123558631463752228835982273412224076811261082892645181820046040

72840412328264170666
000000000000

72840412328264170666
000000000000

37171874122232325

42123231624141938341

436243934521424658844603487844245113516648383656
000000000000

6142932231354561121086
436243934521424658844603487844245113516648383656

233167223711

435343684485420658484580480843364947515248273646

122311141

3823938913332

230452434758489464836937227018462471215821484377
31287369312522

000000000000
225951154639477464016848210115781960211421014321

225951154639477464016848210115781960211421014321

34585965434998165331242433
711611515244982789

7515534151431245

155636591831

532142771022

2311

14996632181

11

255332132478324

426112513

133

6314466221

281056341914122

21116

111

2651864153362532

11

4282221141142469071212
287654191537011

1211

2111131

23

111

26341261525374

9710437101424

221

2313

11251432

000000000000
22021171719194772887

22021171719194772887

0011000100
210810541662

131411

3121

1734331542

111
000

111

269487121841403
139151856816591211982377125813140221277412517172501651311055

0000000000
4325113172

4325113172

12273529232535851172197
2351245411100

11

1021231817212974981786

37104168211

000000000000
138941851816537211562373125777139651266412336172171650111043

138941851816537211562373125777139651266412336172171650111043

000000000000
31489439422112

31489439422112

31420161115314521
0000000000

0000000000
31420161115314521

11191391424372

321221781

11

124212571411104012691243141913091707193316191236
000000000000

124212571411104012691243141913091707193316191236
000000000000

124212571411104012691243141913091707193316191236

000000000000
417433028224372103864

000000000000
21743302819397095764

21743302819397095764

234281
000001

23224

24

000000000000
246088796062112230361251633

756211

268911991924209
245383736062110229360251633

34133411271

31

12712896388992

4644415334333

1

418132111

81829261823481181696411

3433331112

132436152

115614121642235

1221347

3925971212

61016201314122128994
35728780790384381097510108360532024145342432308045

28151077195261987
000000000000

31

2815576185159756

2111231

1

432642132038113
000000000000

432642132038113

5203524392255101158211112
000100000010

5203223382054961521497

312156715

000000000000
32422271041088222

123212172947221

2116321411

132
000

32

1

923322122142730491066
41724161710192236321

1121111

3573327812244

21124

379586421223
00000000000

379586421223

7410667113251116
236475726712735293319021253019252579207920826844

43112172

21

231475066636729492728948240517732309203120296807

2113111

32251222211

174251691122

11828221319323496621

232021101723372950293416

1113111

2213917201

10147512141526249118

000000000000
1176111310609731541101893510421209129311091162

1176111310609731541101893510421209129311091162

1008107311769201302110116271702214416741207999
82637413436591192541249

1000104611398791268106515671580188916601202990
000200000000

1000104611398771268106515671580188916601202990

113121
000000

113121

13495870455412424846115919
242427892545155727559072249932913105860931283085227450702258179123855726258987512632405619948870

1566102707156122177277241240
000000000000

1566102707156122177277241240

011311415002
1999110998885150266496221626

147483746268121210361131415

524262225162555130929

28385409224381644150400094853975654103147176728282363140325947
242427422545134327558802249930523105840531282890227446742258110023854492258986902632401919948785

21192

51

11

70841251037586151172278738741

26192820181535233027406

31391567019717169431531619222329475335994902555834568210

323252821173463127644

1

50137162149127151239400685554550

242100722539351127493874249306503100181731213697226338112242203423578866258640082628819619913717

37571001077575145248440382337

887771822755847955110110641233602709717

21819171315203760255

518557805172416362737514033

44354717296152

63535122433

23554716244351

33

1330493220278212627412714

000000000000
2462737748515391132171621

001000114000
2462737748515391132171621

1713121959222218111110

7496058434230681106511

13

518192615172834921216
211221521359194116211399301841

213141291352446441

86182703939468616116516
000000000000

86182703939468616116516

630372728253767100978
215748992327013

1613137551717321

12314615122

2344563

1775665101221

1

17118113321

45504708827115173135894289228163738709081023551514417081945019
268349320420437423351435618575292304

0000000000
2351254141

11

221121141

13232

000000000000
22981088674114227461727381533

3413613142

21871047865111217427693381528

111148421203

1123402524315318025921819
000000000000

1123402524315318025921819

45762137285713

1111581

711227910294160468

0
1

1

000000000000
791718162333701171155

791718162333701171155

141186111021193891613
000000000000

141186111021193891613

00000
11321

11321

111311131
00000000

111311131

000000000000
31281077101432133

31281077101432133

3623154571542725751071301743205417261611910061396955998233966
2585101731122610079991611466181192904749921503132585068

214346213358214

31312117517183913

2764512182062472

816

1473767273721

134352090020326221402960327328144301181813637180601749113543

3154

18101410917287073

44618172522

513252417132112411978

1223638961

525224672

220199711243269452

20212901878146512151518270348887801434258576

1

1

1745040393449105103169236058734

11413596334688646

13124547322212

281110147162139332

2

11213871118253974

1

1512626824

46817649101414

8853428181

311221243631511

1

1

29108462439101626

16585567227

1

2221

31

1131351211

3527034221218922741173711529341102

211112221

11

1111

66938112834226

1

152116191832214

3511767143223

1805077646446101148310470189824

4131716131361021149286

1

1213445111

11

134

1527424122376712222114513

85787631042112

121023881323533

41411131192424496514

1356288284335

33587

121132

4342412264212

412215112

31419111310112853821

115679614336109185385411931

2323328312960101189105

91618171115421021651135

314134117246313123

1713788132338211

3514471019215

31322221814521011938516

241156722397131

7791435131651521

1

1111

3234784349416086120302816

1946921366197412300029884332801743613390152551086323729514382

112

133

000000000000
57487216253590154

57487216253590154

09916514121530644
101822301926318114929159

981111111111377823105

112331829411

19557577595411416535121934

172411611781610174

4281071011384111113

1372133611
0000000000

11231

136113131

00000000000
1202233101214183353

1

2215

1202231101212172843

181611711122240417

00000000000
11122413203134

11122413203134

4371351113211
00000000000

4371351113211

88561558716097147691737517128846273548283108661040310552
000000000000

88561558716097147691737517128846273548283108661040310552

0281748913116
58164971715207616

5683239676510

97565833858123279415221816
000100000120

66451763337102219299171112

3111465212160116454

31132215925132

239163101524312

1112126

11222222

212111262

112201312982931252

245

12109786215034

9341432115816431994316499965861112501051929
000000000000

9341432115816431994316499965861112501051929
000000000000

111111211
000000000

111111211
000000000

111111211

9331431115716421993316499865661012491051929
000000000000

011011124110
9321427115416411992316399364359412491049929

1

1111

2

211581

51

471012576

9321421114516291988315798762957412431046929

143111513162

3247363543439386146504235
000000000000

000000000000
3247363543439386146504235

3247363543439386146504235
227122255411

3
0

3

000000000000
12910131512121714312615

12910131512121714312615

11

00000000
2636682315

2636682315

0
7

7

151412121621613593151318
000000000000

151412121621613593151318

00000000000
186642941521

6211

113631721421

1

11

22

111
000

111

0
1

1

63145292928285283769

11

000000000000
55038563222357013111723

000000000000
55038563222357013111723

2141626866121612
00000000000

1

13431

32101351

21014133368111

1100021137011
114652692431323

0000000000
1255237112332

9542275181

11

3116412

00000
11111

111

11

1

222162522102034847518

512182998172764937
21170355551435438078911702124245194204

0000000000
281462754122

18136274492

1113
0000

0000
1113

111

3

1823183873772832926099141593200135164
000000000000

1823183873772832926099141593200135164
123973624346106184308121627

1

511

1

111

000000000000
12047415581702052083291128372

1

1

321222143442311

1

2

121

335104634721062

1134134427261153991771078360

11111311

1

00
11

11

000000000000
5201917813244577633

1161615612183970312

443221667321

1
0

1

13345161326

21068411114779623
000000000000

555494294342

2513271836221

1

73117511892

34168212192110130208319607472245

000010000000
5192823251535811539712

1411

361410119101731333

1

19473482033113

1

1

136492154187535

11

1

1

1

1

34423461

127191564521182352
000000000100

000000
223441

000000
223441

1

1

22343

0
1

1
0

1

00000000000
126772231115341

0000
1124

11

21

2

1

2676223191341

12132116911

17547665324485124258261323
120000000000

0000
1113

2

1111

21

624393712172839831027
01141671136212

15112112312

2419787141937411
000000000000

1

1

241778712173731

1

2

111

31418142467732

1

3814581217316922
00000000100

3714571116296121
00100001000

793791012311

11

3321616302

1111271

72023201115395310216914
57112621936641661

000
111

1

1

1

0000000000
15421454165

121

14321452155

1

14713471210207

3122233
0000000

2

322213

1

1
0

1

0
1

1

1

1

000000000000
340512222256880179886

020020116000
340512222256880179886

000000000000
326441816195867151686

325431716185867150586

11

1

11

1

11

117446912212

1

2

112122131310
303721862631177824281828215926322614283931822219

3
1

0
1

1
0

0
1

1

1
0

1
0

1
0

1

008011454253
150110831196867106886394311161128145315081154

386152186110196112163228246432439240
000000000000

100000010010
386152186110196112163228246432439240

000000000000
9639423060234263579511248

9639423060234263579511248

5620301461243
25352728203692120602718

2334211431892

18262224153571311301413

24142561317304757153518
000000000000

24142561317304755153518

1

1

240649246103368296112262264156
561481322152083423951

1774480326718486994212221100

7641143141915845

11
00

00
11

11
00

11

0
1

1
0

1

348283368188254202239271259273331376
000000000000

348283368188254202239271259273331376
000000000000

18
00

18

347282365187251199229255233273330369
331010011014

2122153

228108124127142124141155175200227146

114170240581087387995273102216

1
0

1

00000000000
1131226131511

1131226131511

00000
14236

14136

1

1
0

0
1

1

575457444418432425314366343521560347
000000000000

2
0

2
0

2

121113311
000000000

0
1

1

111113311
000000000

11113311

1

575456439416429419312362340518560346
000000010000

8670695678509410595829786
000000000000

8670695678509410595829786

489386370359350369218256245436463259
000000000000

489386370359350369218256245436463259

0
1

1

0
1

1

1

0
1

0
1

1

225112
000000

125112
000000

125112

1
0

1

0
1

0
1

0
1

1

0000
3314

1
0

0
1

1

3313
0000

21
00

21

311
000

311

0
2

2

133313481021
00000000000

0
1

0
1

1

100
111

0
1

1

1

12231348911
00000000000

00000000000
12231348911

1

1

1223134881

2
0

2
0

0
2

2

1211
0000

1211

000
114

0
1

0
1

1

0
3

0
3

2

1

11
00

11
00

11

000000000000
62748074714396115107795768

000000000000
405139624522474132302741

000000000000
405139624522474132302741

405139624522474132302741

000000000000
222341112621497475493027

000000000000
222341112621497475493027

222341112621497475493027

1
0

0
1

1

000000000000
125106956410271110104131141113116

125106956410271110104131141113116
000000002000

000000000000
393442334932534456694160

393442334932534456694160

000000000000
725333243528273446555733

725333243528273446555733

2
0

2

14172071810302522171521
000000000000

14172071810302522171521

00000
21132

21132

0000
1231

000
131

0
3

3

1
0

1

0
1

1

11

000000000000
3510864111614412

111
000

11
00

1

1

0
1

1

349864111514412
000000000000

000000000000
249764111414412

249764111414412

000
111

1

1

1

0
1

1
0

1
0

0
1

1

004000044100
1441101013398511240887111814101362126915861005

131113121
010101110

2
0

1
0

1

0
1

1

0
1

1
0

1

11
00

00
11

11

1
0

0
1

1

1
0

1
0

1

00
11

11
00

11

1
0

0
1

1

150100128748593141173178129158103
000000000000

150100128748592141172178129158102
000000000000

150100128748592141172178129158102

000
111

11

1

9741449393913
000000001000

1
0

1
0

1

0
1

1
0

1

00000
12122

121
000

121

21
00

11

1

0
1

1

000000000000
9541446263713

300002010223
9541446263713

1

1

6531425161510

01
11

1

00
11

1
0

0
1

1

1
0

1

11
00

00
11

11

846604829558800503368624597732943578
000000000000

846604829558800503368624597732943578
000000000000

846604829558800503368624597732943578

763043446867265235434334
000000000000

000000000000
763043446867265235434334

1

763043446867265234434334

1
0

1

00
11

1
0

1

0
1

1

11
00

00
11

00
11

11

11
00

00
11

11
00

11

000000000000
744147283935665887606138

1

00
21

1
0

1

0
2

2

000000000000
744144283935665786606138

744144283935665786606138
200030000100

1

21

102101044710118116

112

623832183131594674495032

1
0

1

1274571983237
000000000000

0
1

0
1

1

1
0

0
1

1

9611758226
0000000000

9611758226
0000000000

9611758226

0000000
1221111

0000000
1221111

1221111

11
00

11
00

11

12
00

00
12

12

0
1

0
1

1

1
0

0
1

1

00
21

1
0

1

11
00

11

1

1
0

1

000
113

000
113

113
000

113

1
0

0
1

1

5479481071012114
000000000000

000000000000
5479481071012114

5479481071012114
000000000000

5479481071012114

000000
111111

11
00

11

000
111

111

0
1

1

000000000000
83657963974511984999711594

000000000000
83657963974511984999711594

83657963974511984999711594

146111211
001000000

2
0

2
0

2

1
0

0
1

1

14311
14211

0
1

1

0
1

0
1

1

00
11

1
0

1

0
1

1

1
0

1
0

1

0
1

1
0

1

0000000000
283543516311

283543516311
0000000000

283543516311
0000000000

283543516311

163110148491161089713899167229116
000000000000

1
0

1
0

1

003000000000
163110148491161089713899166229116

000000000000
163110145491161089713899166229116

163110145491161089713899166229116

18181310111423921016719912
000000000000

0
1

1
0

1

000000000000
1817139111423921016619812

1
0

1

1816139111423921016619812
000000010000

1816139111423920916619812

0
1

0
1

1

00
11

1
0

1

1
0

1

518443262532114
00000000000

518443262532114
00000000000

518443262532114

000000000000
92216513141015232555

002000000000
92216513141015232555

1
0

0
1

1

0
3

0
3

3

1
0

0
1

1

92210512141015222455
000000000000

000000000000
92210512141015222455

92210512141015222455

0
1

0
1

1

1
0

0
1

1

000000000000
85697554105628788100898255

1
0

0
1

1
0

1

00000
11111

1
0

0
1

1

111
000

00
11

11

0
1

1

1
0

0
1

1

0
1

1
0

0
1

1

000000001000
8267745310562848897868155

0
1

0
1

1

000000000010
8267745310562848896858155

8267745310562848896858055
363325223022485669504625

16475193111219894

30304226563725208272526

2113
0000

12
00

00
12

11

1

000
211

211
000

211

0
3

3
0

0
1

1

2
0

2

261710449113033
00000000000

000
115

000
115

115

00000000000
251610449112533

111
000

111
000

000
111

111

16312242
16412252

1

1

02000000000
1410534782013

1110534662013

1
0

1
0

1

00
12

00
12

2

1

000000000
4352872251

324

00000000
45287211

1
0

1
0

1

0
2

2

421
010

000
411

411

1

21265111
00000000

1

2265111

1112
0000

1112

11
00

1
0

1

1
0

1
0

0
1

1

2

151010333521

00
11

11
00

00
11

00
11

0
1

1

1
0

1

442443344144517094351724
000000000000

11

000
111

000
111

111

1
0

1
0

1

1111
0000

1
0

1

111
000

111

1
0

1
0

1

00
12

12
00

12

000000000000
431735202928384364261321

00000000
12112511

00000000
12112411

12112411

1

431633202827384159251320
000000000000

431633202827384159251320
224451155002

1

11

1

1

39718121824343138231218

1

13103313310

12

1

1

1111321

111
000

1
0

1

0
1

1
0

1

0
1

1

11232732

000000000000
14712912101722723

14712912101722723
001000000000

312
000

1

32

146991161520723

1

1
0

1

000
211

211

2

36514121

0264000612000
224248270211215155274355420229237180

22329261311264837364
001000000000

1

1
0

0
1

1
0

1

000000000000
2172312710244327263

2172012710234327263
000000000000

000000000000
2172012710234327263

2142012710234327163

31

1
0

1

3
0

3

00
11

0
1

0
1

1

0
1

0
1

1

01000100
21621511

262311
110001

321
521

2

13
00

1

3

00
11

00
11

1

1

00000000
43831234

0
2

2
0

2

00000000
23811123

1271123
1271113

1

1111
0000

1111

111
000

000
111

111

0
1

1

0
1

0
1

1

274212111

3121

1

000000011000
222217227177202142247297369225231175

021833671627001
221212225169200140241293357218230173

1
0

1
0

1

1
0

1
0

1

0
1

1
0

1

11
00

00
11

11

00000000
146621511

0
2

2

00000000
116621511

1

11562131

1

11

1
0

1

24552464823
00000000000

0
4

4

111
000

111

11
00

11

2211342421
0000000000

2111342421

1

1
0

1

000000000
211111231

211111231

103
233

10
13

3

1131264
0000000

0000010
1131264

11253

1

3

1

1111111
0000000

1
0

1

1
0

1

1111
0000

1

111

1
0

1

00000
23151

23121
13121

1

3
0

3

0
1

0
1

1

000000000000
8103523531463

000000000000
8103523531463

8103523531463

21
00

21
00

21

0
1

0
1

1

0
3

3
0

3

000000000000
595252267141536069587354

000000000000
595252267141536069587354

323325174128383638223525

27192793013152431363829

3661259131
000000000

3661259131
000000000

3661259131

3211
0000

1
0

1

000
211

11

1

1

2
0

2

0
1

1
0

1

010000010000
43422828278333647604728

43412828278333547604728
000000000000

43412828278333547604728

0
1

1
0

1

0
1

1
0

1

3
0

2

0
1

1

121
000

2
0

2

11
00

11

000000000000
1047075717869113120137799379

0
1

1

2
0

1

1

1
0

1

61
00

61

1

0000000
4443422

4443422

00
11

11

000000100110
1046570677866113113128789277

12

181412151113913178914

943146113611135

1112

11

7546555062479197103576758

1

00000000
13126691

13126691
00000000

13126691

0
1

0
1

1

1
0

1
0

1

0000
4622

0000
4622

4622

000000000
2761116152

276116152
00000000

276116152

0
1

1

00
11

00
11

11

00
11

00
11

11

232222
000000

232222

21
00

1
0

1

0
1

1

1
0

1

3744327911
1502203410

123112451

0
1

1

1

0
1

1

111311
000000

000000
111311

112

1111

21
00

21

1
0

0
1

1
0

1

1
0

1
0

1
0

1

1
0

0
1

1
0

1

114211
000000

000
111

111
000

1

11

10
11

0
1

1

41
00

0
2

2

00
21

21

00000000000
141621211072

00
11

11
00

11

00000000000
13152121771

13152121771
00000000000

13152121771

00
31

3
0

3

1
0

1

32615161

111

135321277111

3673712413388104212317565894354
4153149356663756589239272659670049927677757434487

1

00000000
11122111

11122111
00000000

11122111
00000000

11122111
00111000

1111111

4

1167674534443145197390371411

111

2

31

1

11
00

11
00

11
00

11

1

1

3

0000
11211

0000
11211

0
2

0
2

2

00
21

21
00

21

181
150

2
0

2

00
11

1

1

1

2794352012011
2530754443302136759821613

0
6

0
4

4

2
0

2
0

2

1
0

1

4
0

4

03000000000
112733126341

18733126341
00000000000

18733126341
02020000000

1

111

1271211633

4

1

1471
030

0
23

1

22

0
2

2

1
0

1

0
3

3

0
3

3

6

161
000

1

1

51

31
00

31

26
0

14
26

2

8

1

1

3
0

3

1

0
7

0
4

0
4

4

3
0

0
3

2

1

0000000
81311111

11

030000
8121111

030000
891111

73

2

11111

1

000
211

1

1

11

1028753237211732679116510
010000000000

1

0140000100100
1028643237211732679116510

91
00

1

1

2

1

1

4

82618303419132336551439
03410000000000

29

2

1

2652311212

1

8

111222411451

9

3

710185

12951413

3

1

2

2

21937

1

23

2

6

1

473151456203048517

050000000000
2223232473136121

1

1210232363136121

211

3

2

1

9

4211

11112

355344114655111512
097560000000

00000
62211

62211
00200

1

11

5

2

00000000000
159221211217

1
0

1

00000000000
149221211217

1

121121127

0000
1161

1161

83
85

2

1

1218314337113
000000000000

158314337113

16
0

16
0

16

1

6333

0000000000
1756311221

00000000
15552221

15552221
00110000

1

111221

1

1

2341

00000
21111

21111
00000

1

2

1

11

1
0

0
1

1
0

1

11

211

2454

1

1

24171614269101315253713
000000000000

24171614269101315253713
000000000000

000000000000
24171614269101315253713

000000000000
24171614269101315253713

24171614269101315253713

1
0

1
0

0
1

0
1

1

236372551863
000000000000

236372551863
000000000000

236372551863
000000000000

236372551863
000000000000

236372551863

3331365441536239100684340
000000000000

000000000000
3331365441536239100684340

000000000000
3331365441536239100684340

001000000000
3331365441536239100684340

12114213332

322934533751613697654140

21

224126112714474279304114
010000000000

1

000010000000
223926112714474279304114

111
000

111
101

1

223926112613464179304114
032011154200

3
1

2

01000013000
7103828734122

1

11111

1

37382852922

3

11
00

1

1

000000000000
22251481610372941273912

3

22221481610372941273912

000000
541331

000000
541331

000000
541331

0
2

2

41

3133
0000

312

13

1

2181831279813614121928833123422377
010000000000

6

51
00

51
00

00
51

1

1

4

2
0

2
0

2
0

2

2181691279813614021928833123422377
070000203010

00000000000
1551411610721

1551411610721

003001001000
556053385063657493404727

1075311131086962
000000000000

11

21

2425321221

1

633269875641

100110012120
433745353949536386303925

1

1

23521133

1766567162614

32511211233

3

3112112

11

28132413202715211917259

1131

6853651277755

128712

11324951314

11

0
1

1

0
1

1

00000
22222

22222

0
13

13

1

5348453367588498109474629
000000000000

5348453367588498109474629
100000000000

1

5247453267588498108474629

1

1

00
11

1
0

1

0
1

1

41

001
121

2

1

000000000000
60134363164947116987

000000000000
60134363164947116987

60134363164947116987

1115
0000

0
1

1

1
0

1

000
113

1

11

2

1

1

12
00

00
12

1

2

42121110311
1000000000

1
0

1

22121181
00000000

22121181

0
1

1

10
22

12

0
1

1

0000000
71333111

00
11

11

7332111
000000

5

2332111

4814175711424452282911
000000000000

000000000000
4814175711424452282911

4814175711424452282911

0
4

4

275561025185634676527861440546576540475140483550
5517612212485792344426861606330

1121114181
000000000

0
1

1

1121114171
000000000

000000000
1121114171

1

1

12114171

2

00
12

12
00

12

11
00

11
00

0
1

1

1

664418461415558514378337341586649492
000000000000

664418461415558514378337341586649492
000000000000

0
1

1

664418461414558513378337337586649492
000000000000

261318181

12231191436612

663414455411555509358315283580648489

00
14

14

000000001000
4533232220181541341511806511

0
1

1
0

1

333451644451
000000000000

3132
0000

21

1122

333141612451
000000000001

2311316145

1212

000
232

231
000

21

2

1

0
1

1

0
1

1
0

1

265113110777314545
0000000000

00000341113
265113110777314545

52242314425

3112398

2118

1716136457731

1

4174475

1
0

0
1

1

00000000000
13438101835133

13438101835133
00000000000

13438101835133

818127451821322051
3100007221220

0001
1111

1

1

1

212962591225221
122201013120

1

12

1

1

1

11111

1

1

1

13113413

1

611242

1

11

1111

1

1

1

1

111

233114
000010

3

211

11

13

1

1

2

112341

1
0

1

0000
1112

1112
0000

1112

11
00

11

5226276145776
000000000000

000000000000
5226276145776

1

5226276145676

4231621711233569754
000000000000

21513123331
00000000000

21513123331
00000000000

21513123331

222151658223366423
000100000001

111
000

1

1

1

1
0

1

1

00
11

11

0
1

1

1

0
1

1

00000000
522199241

11111

41119923

0
1

1

21
00

1

2

1
0

1

8108378153322
0000000000

8108378153322

211162
000000

211162

00000
62423

62423

1
0

1

1

00
11

01
11

1

0
1

0
1

1

3111

196462397419444748275291516393265447
176799172763222

1346231532
0000000000

1346231532
0000000000

1

1246231532

000000000000
147266421647

000000000000
147266421647

147266421647

1
0

1
0

1

000000
3433517

3433517
000000

3433517

22
00

22
00

22

272255731
000000000

000000200
272255731

272255431

1

1
0

0
1

1

5431616716413
000000000000

00000000000
32216233413

32216132413

1

1

2114413
000000

2

14413

1

0
2

2

000000000000
1389511518132626157

1
0

1

21
00

1

1

1

342221311
00000000

33122121

1111

000
331

331

3
0

3

0100
2111

1

21

000000000000
136411021310922127

136411021310922127

12
00

12
00

1

11

2040384331317582152532711
61000019773060

000000000
231521247

231521247

0
1

1

34121

000000100010
4825241110221075

1

1

31

11122331243

1

1

2181

111

1

4

1312137622

11
00

11

1
0

1

78143141291234433
000000000000

269896720112

21

5233633514221

000000
113451

113451

12
00

2

1

33633519224122
00000000000

33633519224122

1
0

1

1215
0010

1

5

2

16247
00000

16247

111
000

11

1

0
2

2

00000000000
85104792324211

85104792324211

213
000

1

113

1

0
1

1
0

1

000
131

00
21

1

2

00
11

11

121
000

000
121

121

0000000000
1112111831

1112111831
0000000000

1112111831

000000000000
25711561528322

0
1

1

01
12

11

00000000
374151122

11

111

1

25414112

0
1

1

1
0

1

1
0

1

1
0

1

111581512
00000000

111581512

11321
00000

11321

000000000000
6675431034131452397124916594

0
1

1

112358
000000

112358

000000000000
6674431023931449346224916594

6673431023931449346224916594

1

00
11

11

17321212
00000000

1

00110100
16321212

16211112

1
0

1

4911778161811
0000000000

0000000000
11222210911

11222210911

00000000
37955669

11

1

37955468

1
0

1

14251233
0000000

0000
32211

22210

11

00
11

1

1

12133
02133

1

000000100000
772922632323223597570912838319

742892602313223577164842433315
000000000000

211111

4

742872602263223567163822432314

1

1

111347242
000000000

111347242

1
0

1

00000000
22122211

22122211

2
0

2

0
1

1
0

1
0

1

000000000000
311812610111441312

311812610111441312
000000002000

111
000

1

1

1

000000000000
31181259111239312

31181259111239312

1

16713522262660123
00000000000

00000000000
16713522262660123

1225210
000000

1124210

11

1

00
11

11

1
0

1

324122213112
00000000000

124122213112

2

30000000
937415172333

4373971725

2161068

00
11

1

1

1

12
00

1

11

2111

193209179203167197286286356231197168
010100101110

000000000000
124122222211

124122222211
002000000000

122122222211

0
1

1
0

1

00
11

1

1

0000000
1111211

0000000
1111211

1111211

00
11

1
0

1

1
0

1

12
00

12
00

1

1

1

11212
00000

11212
00000

1112

1

1

1441362
0000000

0000000
1441361

1441361

1
0

1

236787171931663
000000000000

111
000

111

23234221641
00000000000

23234221641

3355314172912
0000000000

3355314172912

4312211

1
0

0
1

1

000000000000
1012975357771115

0
1

1

0
1

1

000000000000
1012975347671115

1012975347671115

915138512121541362
204425302228638214828166

0
1

1

2432510202
00000000

1422510202

1

1

0000
1222

1222

1

0
1

1

0
1

1

11
00

11

00
11

1

1

1
0

1

00
11

1

1

4

00010000000
14451413172881

7

1

2211491711

2

1

124136811

0
1

1

00
11

11

0
1

1

1

1411423717721
000000000100

1

1

211322716111

1113

12

1

224271211
00000000

224271211

0
1

1

00
13

3

1

6221410535
00000000

2121

11

421129434

0
1

1

0000001000
15311241312

1

2

431231011

1

111

0
1

1

1
0

1

72343
00000

1

71333

1

41212642
00000000

1

31212642

159144131148125155195164154181162142
000000000000

720282019132747581146
159144131148125155195164154181162142

413934745041242628605230

9879604945841337851999999

12122122

2114412231

1

2134221221

41121111

1

42224564114

13152264

000000000000
22120404638296574289282513

08001
178131

16313
01002

14

111

16

22

1

5

2

2644648925112
000000000000

0000
4112

12

3

11

000000000000
2243638923112

1

11241261911

113341

2

1121111

2

1

000000100000
418232010111922461882

1131111237113021
00000000000

1

1

1131011237103021

001000000100
35129881111161681

1

124222112

1112

2112431

1

12111246

1

236434241161

1

1

1

113121
000000

11312

1

000
112

112

0000000090030
13141222211435352097158

00000000000
5552613958265

1

31

1

332141

135149720112

1

2

11111412

3525635419331
000000009000

1

351363342331

2

3

5

11

238445181611

1
0

1

57249112426121
000000000000

57249112426121

12
00

1
0

1

11
00

1

1

21133
00000

21133
00000

21131

1

1

3124793112136417
000000000000

3124793112136417
000000000000

3124793112136417
101000013100

212

1

82111

311312

211

132816172414

1111111

1

11

154244232235677812344318
020100000000

000000101010
21011157413482

11121
00000

11121

1
0

1

111
000

1

11

000000001
171342636

11134145

1

1

2

11

4

2

212142
000110

11212

1

11

08126910111430401
123034192030567210839236

00000000010
252139915121

121

1112211

111

132137610

4131322
0000000

4131322

1
0

1

00
22

1

1

2

000000000
511113817128

1

111

413816128

1

23212353575
01000010001

22212343574

0000
6111

6

111

3711913181522
0000000000

27113101112

1

636132

11

0
1

1

33438516631
0000000000

13321

333851343

0
1

1

1

0
3

3

12219
00000

11219

1

0
1

1

0
2

2

0000
1333

1333

1
0

1

1
0

1

1
0

1

11
00

1
0

1

0
1

1

000
821

821

1

00
11

00
11

11

0
1

1

0
1

1

1111
0000

1111

000000000000
11397513133258222

11397513133258222
000000000000

11397513133157222
000000000000

1

1

11297513123157222

1

1
0

1

1

513113310152649332
000000000000

000000000000
513113310152649332

112111
000000

112111

00000
32111

32111

0000
1111

1111

199229132448322
000000000000

199229132448322

154145363848501762776158283828463717310527332361
20121971319577156281437212563

110131241012200
108053554439831392471557

7494445352543851631135
11189253512101

21331131551

1

484171311

21

121

1

12

1

11

1

11111391

12432

28158106273764611

2122

1

1

1

2

256534613333

532411516

1

1

144

1

1

1

1
0

1

00000000
173371213222

11

223422

431479182

11

12

24548524315021
00120028800

6

132114691

11232121711

1115210516

51310514101540567410
000000000000

0000000000
92112361411

212

7351

14

2122331

000000
111226

1

1

11225

42252373532
00000000000

42252373532

112311
000000

1111

221

00
11

11

121738233016

2111
0000

2111

2
0

2

000000000
16823611824

16823611824

000000000000
7141614814252978467

0
1

1

1

000000000
99116111116393

1222245

11

11

47949911333

31

2000020110
61127610443

41127310333

1

00100000000
147221772811

11216

1

125121562211

1

2112221
0011000

10000
21211

11

1

12

0
1

1

1
0

1

000000000000
31319171615334587867

114
000

1

11

3

1
0

1

111121
000000

1111

111

000000000
2124326102

2124326102

0000000
1122122

1122122

00000000000
24221101425212

111

11124

1

2122411

1

11161016111

26111296141639233
000000000000

1144632391

157833121330223

21343101
0000000

21343101

00000000
82533231

1
0

1

1

7253322
0000000

1

1

63321

12211

1
0

1

1291531161542121
56112441025110

0000000000
33316141511

1

3321614141

1

1

112
002

11

1
0

1

1

0
4

4

202921181821305955121512
000000000000

000000000000
1236623353477

1236623353477

000000000000
52214101114245049455

61242461011

3129268122822215

1446148161612

1

11

000
211

211

0
1

1

000
111

1

11

0
1

1

3323431143
0000000000

3323431143

000000000000
3131011138133245541

00000
11112

1111

11

331323881
000000000

331323871

1

00000000000
28797310213432

1

121131101

11

125112682

241

1

1511

2223108

11

1

3222

111
000

1

11

0
1

1

00000
11111

11111

1

11
00

1

1

0
1

1

0
1

1

00
11

11

0000
2121

2121
0000

2121

1132102413120
514141610133132847411

11111

0
2

2

121121091711
0000000000

121121091711

1
0

1

0000000000
33211251512

33211251512

000
111

111

0
1

1

0
1

1

13211
00000

13211

34697912832314
011100020000

1

1

11114391

21251

3121

1

1

2

12334861423

1

1

000000
123231

123231

16426452034127
00000000000

20
42

22

1112221
0000000

11

12121

1

000000000
93413152615

311

1

1

1

2

1

4

32214202

5111

1

1

111

113

00000
22211

22211

0
1

1

311
001

0
3

3

1

00000000000
24231111929113

00000000000
24231111929113

24231111929113

030101100300
129339233328451858205737211716391897281124491979

215311411191
0000000000

1

1111127121

1

132247

0
1

1

21894718224375612
000000000000

1104148817311

22126912

16332881732611

11151263
00000000

111

1

131163

1

128839013314450858125716208515831801279524451977
010000000000

3161144

128738953313450258115715208415811799278024411976

1112211

11

0
1

1

1

000000000000
7171713872130449155

130300121251
7171713872130449155

2323191721

1

61114884191926594

0
1

1
0

1

000000000000
5035644770415270541007199

5034644570415064541007199
000010000000

1

502758406340405829997098

1

11

1

124

1221121

111

552393161

126
000

115

11

2

0
4

4
0

4

000000000000
372627214830364539142620

7352321
0000000

4

7312321

371924164830344237132620
000000000000

12111

11343

11111332

112216

1

361822144327273424132518

1646313522384897129332221
011010131100

422695112311
0000000000

422595112311

1

11

0000000
3931611

3931611

2
0

2

1111
0000

1

1

1

1

0000
1111

11

1

1

1

11163
00000

62

1111

2112
0000

2112

1191414611225679611
000000000000

1181314611214871611

1

1

17

18

000020000000
1116109812171421251918

1115107412161220231918

2222

1

1

1

5101819136141237815
000000000000

0
2

2

1
0

1

00000000000
2242221071333

2242221071333

11

0000
2222

1212

1

1

1385113239212
137593239212

12

36114
00000

331

314

31
00

31

226141
000000

211

1413

2

1

124221
003201

1211

1

2113
0000

1

1
0

1

113
000

113

111

000000000000
382021112311111816184673

38192111231181514184671
110020001000

1

1

1

1

2431486

3

37132071810375184671

13322
00000

13322

17112888111322

2
0

0
2

2

16552581713523
000000000000

2342310111
00000000

234239111

1

0
1

1

0
1

1

00
11

11

1
0

1

21
00

1

2

0
1

1

111123441522
010000000000

11123441522

718335962422235
000000000000

0000000
311121115

311121115

000000000000
71532484137235

71532484137235

124212299
000000000

000000000
121211277

121211264

1

3

3122
0000

3121

1

1

00000
11111

11

111

0000000
2111221

00
11

11

112
000

112

0000
1111

11

11

00000000
33313671

1
0

1

0000
1335

1335

131332
000000

1

13332

0
1

1

0
3

3
0

3

46212277244
00000000000

0
1

1

26212167144
00000000000

26212167144

0000
1111

1111

000000000000
325232212194669113387

1111
0000

1111

324232112184669112387
02012291629120

11

815312

1

31

22151611

1642145153

1311

11

11671111

1

1

1

13

1

136

14

1111

1

21113

23551271611

1

11

1

11

111

111

1

424331181

11

31223

42217151121375781638
000000000000

0000
2111

1

1

11

1

42217131120375681538
000000000000

11214

42117131119355577538

00
161

10
161

14

11

000
111

0
1

1

00
11

11

0000000100
7369311325

0000
6214

6214
0000

6214

1

237112

0000000
1131111

1131111
0000000

113111

1

000000001
1108241061021

00000000
41115345

11
00

11

00000000
31115344

31115344

000000
611125

5

111125
000000

1

11124

1

000000
124238

124238
000000

13231

217

151112
000000

15111

1

1

000000000000
1752221814145761741294745

000000000000
1744221814145761741284745

000010000000
1743221814145760631284645

11

1742211813145760631284645
000000000412

123247345335

122211

1023137545152561113938

1

34564111141

4121

1

1

181111

1

1

1111

1

81

525225172529663368464027
010010021000

00000
21211

00000
21211

01000
21211

2

2

11

2
0

0
2

2
0

2

1245234454994
000000000000

1245234454994
000000000000

0
1

1

1245234444994
000000000000

1245234444994

1111

000000000000
323612111217471749261816

6304445575357
000000000000

301
00

301

00000000000
64445475357

64445475357

000000000000
2657681240104423139

3
0

3

000000000000
2627681240104423139

2627681240104423139

1112
0000

0000
1112

11

1

2

0100
1431

1331
0000

1331

324141627371
000000001000

000000000000
324141626371

324141626371
000000000000

324141626371

000000000000
323237776554

1122535152
0000000000

1122535152

0000000000
2221412142

2111412142

11

21
01

2

000
111

000
111

111

812335677159170181412181083146311491073512
02910010000000

000000000000
385826384831423451434254

384426374831423251434154
050001000000

00000
11613

163

11

000000000000
6521113563110171

21

652111156310171

100000000600
182522222317111736201944

1

11

172422222317101736131944

000000100000
148241122512131059

1

1211121

13223102131211849

21

1

29

0000
13121

0
10

10

00
21

21

31
00

31

1

1

1815111

025034120310030
73829667175416397731084942132010101002453

1321
0000

1321
1000

31

11

5

2194331121
000000000

5
0

4

1

0
1

1

030000000
2134331121

4

1

254331121

211
000

211
000

211

1

000000000000
8472821141750781521359

1

11

1

19943718295211
00000000000

14712618284311

5231119

7361917111032491001238
050000000000

1

1

1

481413510144179412

4

2

2

1

311214174

4

1

12

1

1

334331344722

1

6474995304195285869277531031870886386
121301420220

27161042317824422166
410307356281343389598480675598567245

11211

1

1

301204231190215299495363547419452163

2

5214221721

131111

11112

111114

1

7370897991578264881459071

212311

5313123

11942972323323

41

1

12112122

436132125306
236190173135185196325271356270317141

1141141332

111231231

164141424531

332341214631

13121023

151116333915136

20112612911114415628323128620325592

2657155314101

1612233

510235810514843

1520107151711182482227

11
00

00
11

11

219971312163048223
000000000000

3
1

1

1

1
3

1

1

111111

020
131

111

000000000000
19961212153047113

19961212153047113

1992121
0000

1
0

1

5000
1991121

1

2

3

1947111

1

101

7

1

1

4

2

7

19121
00000

19121
00000

12

7

2

1

1

76124139848215685737712310553
020000010000

0
9

9

1
0

1

211
000

1

1

2

76112137848115585727712310553
000000000000

76112137848115585727712310553

611311

000000000000
425169128114649225

32277247192922
02000000000

000
111

1

1

1

0000000000
2811111112

2311111112

1

4

1

0000000000
133613518261

133613518261

831
000

1

3

2

2

1

1

2

0000000000
1392104427205

00
11

11

24
00

1

23

1292104424165

31117559419327
00000000

00
11

00
11

11

3117558419327
0000000

1
0

1

317458409327
317558419327

11

000
111

000
111

11

1

000000000000
14017116712611215392167167188115110

000000000000
14016916612611215391166165187115110

010310011302
13916116212611214782154138185113106

000000000000
1171182881826854

51132431

16412121323

1

5567813231

1

000000000000
25212143315427

25212143315427

000000000
213111511131

11

11311151131

1
0

1

22263444223
00000000000

22263444223

120100064010
128101124787812042723716010079

11

121

1249312177751173961301529576

1241

1112

111

141312181

2211111211
1110000000

1111121

1

1

28736712363938
00000000000

28736712363938

121

0
1

1

2123336111821322
000000000000

2123336111821322

000000000
54691227223

100000000
54691227223

44691227223

13
00

00
13

3

1

1

000000
211121

1112
0000

1112
0000

1

112

21
00

21
00

11

1

000000000
31936161010115

2

3000000001
31934161010115

2
0

1
0

1

0
1

1

33

7
0

1
7

5

1

0
239

0
26

4

22

1

120
0

116

4

1
92

91

834161010114
000000000

000011000
33416910114

1

1341587114

13

01
51

5

11
00

00
11

00
11

1
0

1

0
1

1

12015654233557313

1491041310010
267218281983234232771401133214801476309029642194

000000010
334743610175

1
0

1
0

1
0

1

1333435782
000000000

1332412351
000000000

162412251
000000000

162412251

0
27

27

0
1

1

00000
23431

00000
23231

23231

0
2

2

0
1

0
1

1

0000000
1131372

0000000
1131272

0000000
1131272

1131272

0
1

0
1

1

11
00

11
00

11
00

11

1

560643651144118235456214955821188899467
000000000000

11

1
0

0
1

1
0

1

493121102922251217472932
000000000000

11
00

0
1

1

1
0

1

493020102922241116462931
000000000000

111
000

1

1

1

4929169272121911442830
000000000000

4929169272121911442830

1312325111
0000000000

1312325111

1
0

0
1

1

1111
0000

1
0

1

1
0

1

00
11

11

0
3

0
3

3
0

3

000000000000
511609625143117925235934835631140869435

000000000000
511609625143117925235934835631140869435

000000000000
511609625143117925235934835631140869435

511609625143117925235934835631140869435

1
0

0
1

0
1

1

00
11

0
1

0
1

1

1
0

1

000
111

11
00

00
11

11

1
0

1
0

1

1
0

1
0

0
1

1

11
00

00
11

1
0

1

0
1

1

2
0

2
0

0
2

2

11
00

00
11

00
11

1

1

023000010000
514279810182842469

113
000

0
1

1

00
13

3
0

3

1
0

1

0000
1222

11

1211
0000

1211

01000010000
58263101023121

21211
00000

1
0

1

00
21

21

1
0

1

00
11

11

001
111

11
00

11

0
1

0
1

1

0
1

1

1111441411
000000000

1111441411
000000000

1111441411

1
0

0
1

1

11
00

11
00

11

0
1

1
0

1

0
1

0
1

1

1
0

0
1

1

12
00

12

00000
11123

00000
11123

11123

1
0

0
1

1

11221
00000

0000
1221

1221

0
1

1

1111
0000

0000
1111

1111

0000000000
13723271213

000
161

11
61

5

1
0

1

1272327612
0000000000

0010001000
1272327612

1262326612

1
0

0
1

1

0
1

1
0

1
0

1

13111
01101

1
0

1
0

1

0
1

1
0

1

0
1

1
0

1

0
1

0
1

1

00
11

1
0

0
1

1

0
1

1

00000000000
43732345323

0000
1112

00
11

1

1

000
111

1

11

0
1

1
0

1

00000000000
32632211323

00
11

11

123111112
000000000

1

1

2311112

0000000
2221213

2221213

14
00

14
00

14

2103113312888861446839686944825189320581718
000000000000

1311121
0000000

0000000
1311121

111
000

111

121111
000000

121111

0
1

1
0

1
0

1

000000000000
1381051487684102192157207551351175

1381051487684102192157207551351175
000000000000

0
1

1

100000010000
1381051487684102192157206551351175

1

75432659111186

1

9887112646386152103131494304147

32133291910344464463921

1

1956102111348091356731490783611133417021541
000000000000

133335112111
000000000000

000000000000
133335112111

133335112111

0
1

1
0

1

1955101211198041349721488779601133316961535
000000000000

000000000000
1955101211198041349721488779601133316961535

1955101211198041349721488779601133316961535

12111
12011

0
1

1

1
0

0
1

1

1
0

1

0
1

0
1

1

51012313544
0000000000

51012313544

0
1

1
0

1

0
2

2
0

2

96355344752
00000000000

1
0

1
0

1

0
1

0
1

1

96355334652
00000000000

00000000000
96355334652

1

96255334652

00
11

11
00

11

112433101624710
4840543954485470110714533

000000000000
121727182017252857182014

000000000000
121727182017252856182014

9162317181620264913159
010010000010

26213322

814201510132025457106

1

11

1121132

1
0

1

2111525234
0000300210

21111214

2

1

13

112111321
000000000

112111321

1
0

1

0
1

0
1

1

411

97343104381776
000000000000

000000000100
97343104381776

97343104381676
000000000000

97343104381676

0
2

2
0

2
0

2

000000000000
261518132817152320291513

261518132817152320291513
000000000000

010001100100
241011112511151911221413

235881571412119118

143310371235

2572364971
0000000000

313321

254121361

1214

4743386371012
000000000000

4743386371012
000000000000

000000000000
4743386371012

000000000000
4743386371012

4743386371012
000000000000

4743386371012

149101141418001
13211812696135102179201241116114106

711123885
000000000

000000100
711123885

51113574

211211

0000
2112

212

1
0

1

000
111

000
111

111

1
0

0
1

1

000000000000
41110111181723314

11

121

11

1

411811118521114

2111639
0100436

21123
00000

21123

000
111

1
0

1

11

00000000
362257113

00000000
362257113

362257113

000000000
221121256

221121256

272545133930345349522237
000000000000

000210000
1126610112

1

1

11245102

000000000000
272444123726284739512135

1

262241113625254638462034

122111311511

122
000

122

2
0

0
2

2

213211
000100

121

1

11

1

1

00
11

11
00

11

4753111730233

11

1
0

1
0

1

0
1

0
1

1

1
0

0
1

1

89634453695911394106487641
000000000000

89634453695911394106487641
002010388001

1

8763425368581098597467538

1111111

111

255166220951239976919172527734274320256470

1

294148257929644469251972
100000200000

11

1

11
00

1
0

1
0

1

0
1

1

1111693221
0000000000

1111693221
0000000000

11371221
00000000

1

1

16121

1211

11322

2

132

441721
000000

441721
000000

441721
341721

1

000000000000
283442227329493363231769

283442227329493362231769
000000000000

000000000000
283242217328483362231769

283242217328483362231769

2111
0000

2111

1
0

0
1

1

1333
0000

0
1

1
0

1
0

1

0000
1233

1233
0000

1233
0000

1233

131519221210132

6

067101042615402
120135152130112139104126187156141111

12111
00000

0000
1111

1111
0011

0
1

1

1

1
0

1
0

1
0

1

1
0

1
0

0
1

1

1112112
0000000

1112112
0100000

0
1

0
1

1

00000
11211

11211
00000

11211

1
0

0
1

1

267103661117222

000000000000
11710111575841118893121147132105

000000000
585116811

000000100
575115811

000000000
575115711

575115711

1
0

1
0

1

1
0

0
1

1

000000000000
223626283233274251402943

000000000000
213324232930264144362743

000010000000
213324232930264144362743

1

213321232628264043352743

1211

211

00000000000
13253311742

13253311742

123211
000000

11

12311
00000

12311

95597942527756436110710161
000000000000

95597942527756426110710161
000000000000

010000000000
95597942527756426110710161

95587942527756426110710161

1
0

0
1

1

1
0

1
0

1
0

1

112
000

0
1

0
1

0
1

1

11
00

00
11

1
0

1

1

0
1

1
0

1
0

1

77162122
00000000

00000
47111

00000
47111

47111
00000

47111

36212
00000

00000
36212

36212
00000

36212

00100
11212

0000
1112

12
00

12
00

12

00
11

11
00

11

1
0

1
0

0
1

1

137155444112
000000000

000000000
137155444112

000000000
137155444112

137155444112
000000000

137155444112

00000001010
1761167121322

11144612

1

3142313
0000000

3142313
0000100

0000000
2142211

1

2132211

2
0

2

1

0000
2112

22
00

22
00

2

2

11
00

11
00

1

1

0000
2361

11
00

11
00

11

00
12

00
12

12

00
14

00
14

14

11

0
1

1
0

1
0

0
1

1

6
0

6
0

0
6

0
6

6

00000000000
21013129717437833

1345537102031
01011220200

21111
00000

1

211
200

1

1

1
0

1

12243159173
0000000000

12243139162
11203135110

1

1345

11

1
0

1

000
111

111

1797441033582
0001001000

0000000000
164643832582

0
1

1

12

0
1

1

153443831582
151443831582

2

1
0

1
0

1

00
11

1

1

0
2

0
2

2

1
0

1
0

1

2

0
1

1

000000000000
51119161712174685431

0
1

1
0

1
0

0
1

1

51118161712174685431
000010234100

311534101512
0100000000

000
111

110
111

1

0000
1355

0000
1355

1355

1
0

1
0

1

214314911
000000000

000000000
214314911

214314911

011010010000
21017111312113366211

1
0

0
1

1

00000000000
3253511419111

00000000000
3253511419111

3252511419111

1

14

1142248923
000000000

000000000
1142248923

1142248923

000
112

000
112

112

285225181
00000000

00000000
285225181

285225181

10
11

0
1

1

1
0

0
1

1

000000
214111

000000
214111

214111

000000000000
12182017151920224714725

68109151714412110
20021025000

12521010624217

3655476123
000000000

3654466113

11

1

000000000000
94742437312614

000000000000
94742437312614

94742437312614

20
22

2

33534111

3
0

3

10487887911011031523516611744
000000000000

0
1

1
0

1

10487877911011031523516611744
000000000000

10487877911011031523516611744

111
000

000
111

111

1111

426519022938144420341437352040474230516249663581
575364050291583350794734040026520975657263261756146588458471

1786325118364
000000000000

000000000000
1786325118364

000000001000
1786325118364

00000
32521

32521

000000000000
146132596364

146131596364

1

134698861937417415221317312696
806045445353

115546653836815612919115411689
000000000000

622466734672
115546653836815612919115411689

75314028525311785135996666

34212421259324152494321

3232411441
0000000000

0000000000
3232411441

3232411441

000000000000
813136211318131244

002000000000
813136211318131244

813116211318131244

141726131112192126302027
000000000000

000000000000
141726131112192126302027

215101531625
141726131112192126302027

000000000000
613142957141510814

613142957141510814

6371026741014108
000000000000

6371026741014108

10257898916339047446528169295918551961
000000000000

11236522
00000000

11136522
00000000

00000000
11136522

1

11135522

1
0

1

10127778756278937306267829005618401955
000000000000

164221142220223134
000000000000

164221142220223134
000000000000

164221142220223134

000000000000
9967738736258827266047628785588271951

000000000000
9967738736258827266047628785588271951

9967738736258827266047628785588271951

131116591120292730136
000000000000

48621428411
00000000000

48621428411
00000000000

48621428411

000000000000
93938718212330125

1
0

1

93937718212330125
000000000000

93937718212330125

1
0

1
0

1

1

457338323301372358267271270508459373
890158666796524072725243100371106212173130531072114273

21
00

21
00

21
00

21

10455106711873
00100000100

00000000000
73339468672

73339468672
00000000000

73339468672

3112121311
0000000000

0000000000
3112121311

3112121311

000000020000
113152013341346897116515348

10710141226941846813614027
000000000000

000000001000
10710141226941846813614027

10710141226941846713614027

000000000000
656184533291321

656184533291321
000000000000

656184533291321

11121321
00000000

11121321
00000000

11121221
00000000

11121221

0
1

1

0000000
2112212

2112212
0000000

2112212
0000000

2112212

000000000000
1781611165292134152414

000000000000
1781611165292134152414

1269914318193081914
1781611165292134152414

311161123

111

1161211

1121141

111

27596641553397105117443161
000000000000

000000000000
27596641553397105117443161

000000000000
27596641553397105117443161

27596641553397105117443161

23185106559715790786711614467
000000000000

23185106559715790786711614467
000000000000

23185106559715790786711614467
000000000000

23185106559715790786711614467

200010022100
443376452558790692494394435333349320

00000000000
68253161025726

00000000000
68253161025726

68253161025726

1
0

1
0

1

101022125453
441370444556784689478382408325346314

183283305458664559130936294157185
153279295456657549126785473125163

21214213362

27381384134182619

1111

000000000000
1494363375659859250985971

1494363375659859250985971

000
156

156

000000000000
251215142510232430483613

251215142510232430483613

131544382862
00000000000

131544382862

11
00

11

000000000000
703049433755236162253738340

703049433755236162253738340

12121391759111433145
010100000010

111511
000000

000000
111511

111511

211343295
000000000

00000000
21343295

21343295

1
0

1

000000000000
10912714458121974

000000000000
10912714458121974

10912714458121974

197228137285134312010
000000000000

197228137285134312010
000000000000

000000000000
197228137285134312010

197228137285134312010

312241
000000

000000
312241

312241
000000

312241

000
211

211
000

000
211

211

000000000000
3142118166203557442

3142118166203557442
001000100000

111
000

111

000000000000
2131818156183356322

2131818156183356322

00000000
11211111

11211111

51536410714331
00000000000

00000233000
51536410714231

00000000000
4133437411221

4133437411221

122111
000000

11

12111

1
0

1
0

1

7243246172426508711813
000000000000

000000000000
7243246172426508711813

000000000000
7243246172426508711813

7243246172426508711813

000000000000
10356827941135

000000000000
10356827941135

10356827941135
000000000000

421241141

614442684734

400000140530
597547660454999460555608637726591843

11517198362
0000000000

0000000000
11517198362

11517198362

262026151810262417343317
588540652449995454545584617713576837

312362409311767299243255255394287376
000000000100

312362409311767299242255254393287376

11

00000000000
1248432279810

1248432279810

2122431038
000000000

2122431038

61245106451
0101010110

3124532

311143141

000000000000
1914201039141278142428

1914201039141278142428

55858610131114104
000000000000

55858610131114104

1
0

1

206134179102154123245264317234206393
000000000000

206134179102154123245264317234206393

4635362112564
000000000000

4635362112564
000000000000

4635362112564

000
132

132
000

132
000

132

402733115021201940332829
1806117616381158152512649581036982191619572405

0
1

1

12221241
00000000

12221241
00000000

12221241

3411873543429
000000000000

000000000000
3411873543429

3411873543429

1

000000000000
41011338710715

000000000000
41011338710715

41011338710715

011010110101
433141643543

4111522112
0000000000

4111522112

00000000
12121133

12121133

3391513171060746610558110
542030243325851029114590123

00000000
52211121

52211121

00000000
12113321

12113321

34212210412232
000000000000

34212210412232

0000101100
2313674452

1112442222

12113113

000000000000
3223731511596

3223731511596

1211341
0000000

1211341

000000000000
8126553638112

8126553638112

000010011610
11998138938169280239289332256150

251331143017263322232227
002200021110

11372

251329122916262821151927

8353561612304107
000000000000

8353561612304107

000000000000
8682102764546238193236299223116

8682102764546238193236299223116

000000001110
291821181321101827202651

25171815122181122152349
000000000000

25171815122181122152349

41331264422
00000000000

41331264422

1
0

1

126282011578771155976172269173110012741869
003000110100

000000000000
126080911468701149972151247151109312661866

126080911468701149972151247151109312661866

04010021221
16223352542

1112222

111131111

156531152120141
000000000000

156531152120141

9286151614161292
000000000000

9286151614161292
000000000000

9286151614161292

1012020130102
12362516102462

22111
00000

22111

0000111000
2223146136

222235125

111

2
0

0
2

2

000000000000
91143210944131

000000000000
91143210924131

91143210924131

2
0

2

334231441
331010110

1
0

1

0
1

1

0000000
3211321

3211321

36242610198343239232617
761169111712145

100000011000
1729165129111097

51211416353

111764874744

32121573221
00000000000

32121573221

8134321535682
100000000030

412114231

3122211133342

111168432
100161000

232

115112

000000000010
885053435439728468759147

000000000000
10246321219764

10246321219764

784849375137608359688443
000000000000

784849375137608359688443

1212222
0000000

1212222
0000000

1212222

31311152425762
00000000000

00000000000
31311152425762

31311152425762

000000000000
7353113586964829

000000000000
7353113586964829

7353113586964829

0
1

1

00000000
11111211

0000000
1111211

1111211

1
0

1

115320353230
312632122019464140333820

000000000
112122123

112122123

000000000000
18810578171521131712

18810578171521131712

7734119139957
00000000000

7734119139957

000000000000
41681795667101

41681795667101

000000000100
26202613915212022111613

0000000000
4182644422

4182644422

2119181391214151661410
000000000000

2119181391214151661410

111121
000000

111121

503362264447664949675742
000000000000

503362264447654849675742
493060244039594645655337

11

1121541222

1311111

212212

1

11
00

1

1

000000000000
159133722216101768

159133722216101768
000000000000

159133722216101768

3611441141
000000000

0000000
2113231

2113231

0000000
1613191

1613191
0000000

1613191

000000000
411324521

000000000
411324521

000000000
411324521

411324521

315116193625183425282221
000000000000

32131225252
00000000000

00000000000
32131225252

32131225252

284915163523162923232021
1214510301851086615

852358779
000000000

852358779

000000000000
7333442283615

7333442283615

0000000000
1211223331

1211223331

121211
100211

2

1

11111
00000

00000
11111

11111
00000

11111

812540194028458385728040
000000000000

812540194028458385728040
9431225151517104

00130022000
632434614267

111114123

532348144

000000
121133

121133

000000000000
3581721912203930313815

3571721912203930313815

1

00
11

11

000000000000
257514138571514208

257514138571514208

1
0

1

4211122111610536
000000000000

1

11

4211112101610535

000000000000
121567043726134414811010774

121567043726134414811010774
000000000000

000000000000
121567043726134414811010774

121567043726134414811010774

000000000000
44133113205573646513839

246186111381331292619
000000000000

246186111381331292619
000000000000

246186111381331292619

00000000
11112211

00000000
11112211

11112211

0
1

0
1

1

20612783172314201119
000000000000

20612783172314201119
000000000000

20612783172314201119

100000000010
402405474288333287414441496426402373

162292130251924159821
368388457260316262381386430385377360

1
0

1

289323377211240181320312389324323310
000000000000

289323377211240181320312389324323310

1511212
0000000

1511211

1

624366274556414825524429
000000000000

624366274556414825524429

00000000
21114341

21114341
00000000

21114341

27171427132027506138229
000000000000

000000000000
27171427132027506138229

27171427132027506138229

00000000000
42134221224

00000000000
42134221224

42134221224

418924512527204025981613637872858101810560729393
000000000000

418924512527204025981613637872858101810560729393
000000000000

418924512527204025981613637872858101810560729393
351314481253333813914

415424382513203525881612635372508068802260319378

122221

3217257259452764283614
000000000000

3217257259452764283614
010010000000

000000000000
1131221441571

1131221441571

3115226227442360132913
000000000000

3115226227442360132913

000000000000
1551221458111712730726035317811987

1551221458111712730726035317811987
000000000000

000000000000
1551221458111712730726035317811987

1551221458111712730726035317811987

00000
13312

00000
13312

00000
13312

13312

222029191110522541501615
000000000000

114100212411
222029191110522541501615

0
1

1

12141754734121825811
000000000000

12141754734121825811

0
1

1

3113335512
0000000000

3113335512

1
0

1

0
1

1

000000000000
6551243139151561

6551243139151561

22335
00000

22335
00000

00000
22335

22335

001000000000
19786365131719815

000000000001
146222439121359

0000
1112

1112

135222339121158
000000000000

135222339121158

515412245636
000000000000

000000000000
515412244636

515412244636

0
1

1

814376171526362
000000000000

814376171526362
000000000000

000000000000
814376171526362

814376171526362

35182618311912139453123
000000000000

35182618311912139453123
000000000000

000000000000
35182618311912139453123

35182618311912139453123

1111
0000

0000
1111

1111
0000

0000
1111

1111

702631194138656574596443
363193260148264239223261270316335271

000000000000
44223573618262826252237

000000000000
44223573618262826252237

44223573618262826252237

2761681468912341612
111000000000

000000000000
153524227722109

153524227722109

0
1

1

000010001411
1121061046251253

612473222522

5182214232

000000000000
6313453310131351

6313453310131341
110000000300

212113324

3111343371081

0
1

1

14678996811111653695269134127
2489915159165142526

963340216052323520337168
000000000000

963340216052323520337168

263750383649121827223833
000000000000

263750383649121827223833

373325223331222428555733
000000000000

100000000000
373325223331222428555733

363325223331222428555733

000000000000
1117217101912212627148

1117217101912212627148
000000000000

1117217101912212627148

2054544261002
2282013148343539342210

946142614101103
000000000000

946142614101103

343532171717844
000000000000

343532171717844

00000000
14311311

14311311

1232311
0000000

1232311

0000000
62212116

62212116

0
1

0
1

1

20152714143232543322522
000000000000

000000000000
20152714143232543322522

20152714143232543322522
102220212042

121979239618135
000000000000

111

111978239617135

000000000000
7141553118153514815

7141553118153514815

0
1

1

000000000000
275917412258142820711539164019932108275827021801

492532242934382940253924
275917412258142820711539164019932108275827021801

532165195147324112224351309429521312
564187233167366150257375351483548339

109846412813141112
000000000000

109846412813141112

0
1

1

9256652232587
000000000000

9256652232587

1311251030291913261588
102110100001

77196272613821747

54432355584

520012011310
221014161210122042322110

1
0

1

1
0

1

2352232241
000000000

2352232241

000000000000
148111666101519251910

148111666101519251910

122233298814125
000000000000

0
1

1

0
1

1

0
1

1

122232288814115
000000000000

122232288814115

9688146106989396898715610471
000000001000

000000000000
594779445755433826805634

594779445755433826805634

00
21

1

1

1

11111
00000

11111

1

0
1

1

0
1

1

0
1

1

000000000000
364062614137525057764837

11

364062614137525057754737

1
0

1

0
1

1

0
2

1
0

1

1

401932242413508368525417
000000000000

003101101400
401932242413508368525417

1

24

30152415176306346333713

1

94586619171715174

1

1
0

0
1

1

000000000000
1391145312161415107

1391145312151415107
100000011100

1

1

119114531214131297

1

1

1
0

1

15112211989200115941411051137976
020000021010

21121211
00000000

21121211

000000000000
7266646013345398048392631

7266646013345398048392631

795254296668545954735244
000000100000

11

1

775254296668535954735144

1229644151279177
000000000000

000000000000
1229644151279177

1229644151279177

0
1

1
0

1

000000000000
9135988134975134423354614891110992652

000000000000
770538726460383381263392387981854567

770538726460383381263392387981854567

1
0

1

000000000000
14360873712961726910212913885

14360873712961726910212913885

000000000000
15372853412588174141188145152122

000000000000
15372853412588174141188145152122

106505024887315410615610410589

472235103715203532414733

214161671016163015174
000000000000

000000000000
214161671016163015174

214161671016163015174

0000
1111

11
00

11

1
0

1

1

0000
1211

0000
1211

1211

000000000000
423453248577

1
0

1

422453248577
000000000000

422453248577

000000000000
91295161019193915138

91165161019193914128
000000000000

91165161019193914128

1
0

1

0
1

1

0
1

1

1
0

1

0
1

1

0
1

1

000000000000
3918224810243143112167149554755

3918224810243143112167149554755
000000000000

3918224810243143112167149554755

258223307175366213282267357215256253
100210112200

1
0

1

320220124210
981101247720010863103656092178

5231451389451

90106121741941034993525486177

0
1

1

934444771110113
000000000000

934444771110113

1
0

1

1491101799216110021115627914315371
000000000000

1491101799216110021115627914315371

000000000000
39121692012291133212214

39121692012291133212214
000000000000

39121692012291133212214

1
0

0
1

1

1
0

0
1

1

0
1

0
1

1

000000000000
3631701601462341938613290275312128

000001000000
3631701601462341938613290275312128

3631701601462341928613290275312128

11
00

10
11

1

0
1

0
1

1

1
0

0
1

1

1
0

0
1

1

5662384812132492624845706741040781209
163001220014522114041548714419140311535816949207931822914101

12134301815572728216887103
497157297173179226231303360391408499

20911111319161928221811
001000000000

20910111319161928221711
000000000000

20910111319161928221711

1
0

1

101111353221
31994227126123115135212272266266368

0
1

1

1
0

1

431115181062061
10732665152316341311

51115136879610

1310115351

000000000000
295761731098594106180242211228331

295761731098594106180242211228331

000000000000
13112110301511411182325

13112110301511411182325

000000000000
372029182835534439353717

000000000000
372029182835534439353717

372029182835534439353717

211110100031
659533665494573493785683874737799473

1214172206382447123612
000000000000

0
1

1

1214172206382347123612
000000000000

1214172206382347123612

1505471326631908812513314895
010000110400

000000000000
42899119303043153635

42899119303043153635

1

993542214519374255979954
000000000000

993542214519374255979954

000000000000
91020210322142717136

91020210322142717136

166194146202120312214
000000000000

000000000000
156194146192120312214

156194146192120312214

1
0

1

1
0

1

000000000000
148216219217175171193157140177224109

145215215217175170191153138175222108
000000000000

145215215217175170191153138175222108

3141242221
0000000000

3141242221

000000000000
542438312548764257593924

542438312548764257593924
361625251738602636461916

14211431

187948915121812208

6373365103570
277218300207272231367351485325327218

887188771068510766831119468
000000000000

887188771068510766831119468

2315231431215963118283716
11072115921059818117729712313266

815383716269118110170898249

5387106437571

111221216

395169254318406075324165
000000000000

395169254318406075314165

1

0000000000
3135414413

3135414413

312018101519303723505216
961348812112191

5643316811153

16611479111315262410

12111234122

13122

000030000000
3556523548224383665614870

000000000000
3556523447924383665614870

3556513147924383665614870
512591436138871113

2411282334812

3775243562123217

253338166511162341433438

0
3

3

0
1

1

0
1

1
0

1

2667212513312423123
000000000000

000000000000
2667212513312423123

000000000000
2667212513312423123

2667211513312422123

11

90495521442872611091388948
10000100058121

000
111

111
000

111

482930132314352253314025
1971138219627111414

1319108851012189168
000000000000

1319108851012189168

000000000000
163827764811103

163827764811103

0
1

1

000000000000
1448514419161912165

1448514419161912165
000000000000

1448514419161912165

000000000000
271516379182337362116

11
10

1

00
11

11

000000000000
271515369182337352115

271515369182337352115

0
1

0
1

1

16
00

1
0

0
1

1

0
3

0
3

3

3
0

3
0

3

001000000000
1010135211012151916711

1010125211012151916711
000000000000

1010125211012151916711
000000000000

1010125211012151916711

000000000000
553172282827486153516244

0
1

0
1

1

0
3

0
3

3

553169282827486152516244
000000000000

532643232424435749516240
38131910412312925212812

3111325

121223132012112821282928

0
1

1

25265435424
0000000000

25265422424

13

000000000000
131034121319202219169

131034121319202219169
000000000000

131024121319202219159
000000000000

131024121319202219159

0
1

1

0
1

1

1
0

1
0

1

1
0

0
1

0
1

1

000000000000
513551335340462847716141

000000000000
513551335340462847716141

513551335340462847716141

000000000000
1098121213611776178421913763994499551018977

0
1

0
1

1

001000000000
98711321248166316692088227256284814880868

98711321247166316692088227256284814880868
000000000000

98711321247166316692088227256284814880868

11180113113115103149143164141138109
9372519138733

1
0

1

000000000000
2511141412515104552378

2511141412515104552378

776692979897125119111829898
000000000000

776692979897125119111829898

252150112133173162250230315208202176
000000000000

252150111133173162250230315208202176
011160296205

238143108117154148234204288184178160
5324871220404639343826

16810286101121109161127227126121117

1513881613252012201316

24615681110461

310021210330
1462151314141721222411

5391011881312115

113

522612388776

0
1

0
1

1

000000000000
1112199128975171220

1112199128975171220
000000000000

1112199128975171220
000000000000

1112199128975171220

000000000000
610165913119941119

000000000000
610165913119941119

610105913117741119

622

3512237179192115252920
000000000000

000000000000
3512237179192115252920

3512237179192115252920
000000000000

3512237179192115252920

604860253432497776857451
000000000000

604860253432497776857451
000000000000

604860253432497776857451

1
0

0
1

1
0

1

000000000010
2020870941720990807152019201980255220281762

000000000000
14489128638413420118529020115561

14489128638413420118529020115561
000000000000

14489128638413420118529020115561

1

000000000000
1876781813657906673131817351690235118721701

118863132186802234341
1876781813657906673131817351690235118721701

19141731131242420231210

1846679733522709562129217091667232418571650

14860114401296768787710913299
000000000000

1
0

0
1

1

14860113401296768787710913299
101100221020

6

0010
1111

11

1

223111

811935147910101216497170
000000000000

811935147910101216497170

000
111

111

623965244956556460575828
000000000000

623965244956556460575828

00
11

11

0
1

1

1
0

1

100000000000
514913128470571218419588671378164412811176

000000000000
3437409345215941205303339424648606802

000000000000
3437409335215941205303339424648606802

3437409335215941205303339424648606802

0
1

1

170173243184118636655528954996675374
000000000000

1

000000000000
170173243184118636655528954996675373

170173243184118636655528954996675373

0
107

107
1

106

0
1

0
1

1

1011735829546814859650727726972956943
36627928122025740913212576344296339

000000000000
87858243119514253836074166

000000000000
87858243119514253836074166

87858243119514253836074166

000000000000
961281507111810967109125101115135

000000000530
961281507111810967109125101115135

96128149711181096710912595112135

11

6522471976461391631609111758
000000000000

6522471976461391631609111758
000000000000

6522471976461391631609111758

1
0

1
0

1

010100040001
298171191153178194207208211270254167

000000000000
23967167242229291414

23967167242229291414

226129149118121162126131116176190121
275161185145162187183182181241240152

645333671362

432831243825544558524429

0
1

1

1
0

0
1

1

000000000000
341840122415192733413034

1

4281134791438
341840112415192733413034

11729512829138719

1993511413111119207

000000000000
1914131718179812102216

000000000000
1914131718179812102216

1914131718179812102216

461823112318343326554827
000000000000

461823112318343326554827
000000000000

461823112318343326554827

0000
1111

0000
1111

1111

000000000000
26101651110142615211215

26101651110142615211215
000000000000

000000000000
26101651110142615211215

26101651110142615211215

000000000000
372389460296516303778784978422398323

372389460296516303778784978422398323
222430171918503845332419

50500315295053
504953654661668968625428

841137168914684

18912101520263133122411

193625522422172016441710

137213245145334137315315424106155185
022110332611

11104964121439774

12620123913532713330029838393147180

17897101021172320138
1369811860977819018127019814979

2218251115317294427278

53131467614494

10473731613181493

491446101321173099

674734274642775594866137

1051568435335515198

14111475222

275149209157161171231612
000000000000

275149209157161171231612

832366231293
00000000000

00000000000
832366231293

832366231293
00000000000

832366231293

0
1

1
0

0
1

1

000000000000
4312510913211217137

000000000000
4312510913211217137

4312510913211217137

536528574487749756686600697708713552
002000000000

0
1

1

961091038010111087729015812192
000000000000

0
3

3

000000000000
961091008010111087729015812192

961091008010111087729015812192

440419468407648646599528607550592459
11011414816133631815889108136203219

000000000000
9121716131313161512167

9121716131313161512167

0
1

1

00000000000
85127532145

85127532145

80639453567910812596916063
000000000000

80639453567910812596916063

109211771212101379
000000000000

109211771212101379

13121561311121742222012
000000000000

13121561311121742222012

613342152611
241731142722354661543716

8591851519271485

7545362141711144

361451291781523146

1

000000000000
895531345331

895531345331

0
1

1

200011011321
2213116611141824321713

555335610111052

15863258712191010

000000000100
303925252525215042544645

192717121818103833383436

1112813771112915129

1261269911815515422014820312917874
000000000000

1261269911815515422014820312917874

1
0

0
1

1

000000000000
435548285349906675543743

0
1

1
0

1

435546285248906675543743
000000000000

000000000000
435546285248906675543743

435546285248906675543743

0
1

0
1

1

0
1

0
1

1

1
0

1
0

1

9281075983938143896645367752511301073742
000000000000

873951394335647561596739
9281075983938143896645367752511301073742

1132
0000

1132

000000000000
933435298814

933435298814

1
0

1

1
0

1

000000000000
17175131722131515181011

17175131722131515181011

1
0

1

0
1

1

000000000
121111222

121111222

792100590586513498833515444211020974668
515366479471638370515252

7369528368151254811286460347964916611

121

43311214364

00
11

11

0000000000
95945713888

95945713888

241819104617813
000000000000

241819104617813

000000000
121331227

121331227

1
0

1

0
1

1

000000000
612211313

612211313

00000000
21335664

21335664

1
0

1

1
4

0
3

2
0

2

0
1

1

1814291361127152815124
000000000000

1
0

0
1

1

000000000000
1814281361127152815124

1

1814271361127152815124
000000000000

1814271361127152815124

8030411361112174
367181198249327181143209281430456185

00000000000
26431368376

26431368376

000000000000
357175191246323179127197262415432175

357175191246323179127197262415432175

1
0

1
0

1
0

1

3257513036375010358222824
000000000000

3257513036375010357222824
000000000000

000000000000
3257513036375010357222824

3257513036375010357222824

0
1

1
0

1

00
21

00
21

00
21

2

1

0
2

1
2

0
1

1

189168215127242140236244268248195148
010000000100

303455297725395071612739
000000000000

000000000000
303455297725395071612739

303455297725394971602639

1

11

242122142121272545383028
000000000000

0
1

1

232122142121272545383028
000000000000

232122142121272545383028

000000000000
135112138841449417016915214813881

000000000000
135112138841449417016915214813881

135112138841449417016915214813881

202132415211
625053265836717794675437

15142314171715202718135
000000000000

15142314171715202718135

000000000000
191584178242934121614

191584178242934121614

000000000000
2621207219282728352417

2621207219282728352417

000000000000
8665325993063

8665325993063
000000000000

8665325993063
000000000000

7665325993063

1

306121862515200523842255264431353213422636482681
897144636310915011616017515951

000000000000
88066295373282776611731423136213131079938

88066295373282776611731423136113131079938
251211122516131628674519

1036856510725156

84564793671279774411551397132612211019913

0
1

1

613634740656685693408401447691724576
533564666603623662285299261583662534

60614440482511292168994727
000000000000

60614440482511292168994727

000000000000
209301314611101891515

209301314611101891515

25515422124962745548
7881823740645210175997479

1
0

1

321
000

321

1715111024154341
0000000000

1715111024154341

000000000000
332216102513253447372028

332216102513253447372028

252111571611
000000000000

252111571611

0000000000
2541377862

2541377862

1
0

1

693053154039506161625476
234444222333

48233473026313437423349
000000000000

48233473026313437423349

00000
11111

11111

000000000000
19414469162421171724

19414469162421171724

000000000000
5382502041182091381692612641007783473

1
0

1

5382502041182091381682612641007783473
2941267724463872116112784542323

745944365532389379889453

1706583581086858527313514797

537259252190243184473564606658546274
242324272013554775453422

000000000000
17810121571782234157

17810121571782234157

19662952592118186
000000000000

19662952592118186

2382630193227841339719518859
000000000000

2382630193227841339719518859

2
0

2

1335710315111915153
146150117819989196190259222190101

5179502425275243889910240

51425425563

7767584859551271311471027058

0
6

3

3

103343375540
281023152011314833452214

8744741528171238

41522151274

15211673812921116

0
1

1

11664339201620139
000000000000

11664339201620139

0
3

2

1

5430253045295510983796656
000000000000

5430253045295510983796656

3
0

3
0

3

14193947810211613115520710912385
320022451711

00
11

11

75726331274106
000000000000

75726331274106

000000000000
153317141734232816242031

153317141734232816242031

000000000000
1165369627777100110183749247

1165369627777100110183749247

000000000000
11610690116175146385331112106129

11610690116175146385331112106129
000000000000

11610690116175146385331112106129

902021030300
8361925111664709592977190

111

71011413722233621199
000000000000

0
1

1

71011413722233620199
000000000000

71011413722233620199

0
1

1

000000000000
6751774710156486856725281

6751774710156486856725281
313555316436244428283030

341420143617232120362051

22221313882

0
1

0
1

1
0

1

3840443442428260116834456
000000000000

1
0

1
0

1

201616192724252448382729
3840443442418260116834456

115199210112518710
1018261271271410271419

51112142334

42623122639

000000001000
8623855022581838

8623855022571838

223239305518422124422537
000000000000

000000000000
223239305518422124422537

000000000000
223239305518422124422537

223239305518422124422537

0
1

1
0

1
0

1

2173843366154281185193959995
188913121584121818521377194419401974233119301431

38201761810424246523621
000000000000

38201761810424246523621
000000000000

38201761810424246523621

197135158132216185254232219294208136
000000000000

88217413541579
000000000000

88217413541579

7363687196851371121161199636
1279011410114913218817016621715456

1481710135111712461

1

1281762320131413291410

28111214172227272465389

000000000000
623742306049535749624771

623742306049535749624771

431618212333151316275220
112010001004

00000000000
41325694729

41325694729

12
00

12

2
0

2

000000000000
371413171727698234316

371413171727698234316

564038294721354954385427
000000000000

000000000000
564038294721354954385427

1

564038294721344954385427

000000000000
98124109781101213810352175103105

1
0

1

000000000000
98124109781101213810351175103105

98124109781101213810351175103105

591396498471691472371459457620579451
100000001100

0
1

1

264184211209243158196275251406311186
000000000000

264184211209243158196275251406311186

000000000000
326212287262448313175184205213268265

326212287262448313175184205213268265

000000000000
358358507316452379555442575582480324

358358507316452379555442575582480324
000000000000

358358507316452379555442575582480324

1
0

0
1

1

91242116131614271313
000000000000

91242116131614271313
000000000000

91242116131614271313

000000000000
36232418184213652773421

36232418184213652773421
000000000000

36232418184213652773421

000000000000
7101037913661153

000000000000
7101037913661153

7101037913661153

000000000000
6442671911571291268

6442671911571291268
000000000000

6442671911571291268

1
0

0
1

1

000000000000
191118135781596369153106271223177

000000000000
191118135781596369153106271223177

191118135781596369153106271223177

0000
2111

2
0

1

1

0
1

1

1
0

1

1
0

1

411816253313454755493830
13331765141011962

2131344824
0000000000

2131344824

000101031020
16977137171624171320

644343142637

10533931692111813

21001010110
106511111171615174

1

1

2131548111071

1

6415783493

1357264171619261816
000000000000

1357264171619261816
000000000000

1
0

1

210000000000
1356264171619261816

1146264171619261816

000000000000
10079965474826370729482106

10079965474826370729482106
000000000000

000000000000
10079965474826370729482106

10079965474826370729482106

000000000000
3960591002071225334471129858

1
0

1

000
111

111

3960581002061225333451129858
000000000000

3960581002061225233451129858

1

0
1

1

00000
11311

11311
00000

0000
1311

1311

0
1

1

914467307471108861351388769
000000000000

120110311120
914467307471108861351388769

7524491442547763931077048
000000000000

7524491442547763931077048

0000
1111

1111

000000000000
53831246751216

53831246751216

000000000000
91410111913221536171415

91410111913221536171415

000000000000
435242276248354275199240221428429254

435242276248354275199240221428429254
100000001020

000000000000
372739404025254628305330

372739404025254628305330

633135557914
262923172954301230352721

18141312174412618181314

212749513158133

0
2

2

000000000000
371186212191285196144182162363347203

371186212191285196144182162363347203

000000001000
1416993901159510210410018214469

00
11

11
00

11

000000000000
8844676679574756711259447

00000
11121

11121

1

8744676679574755701239247
1761011211241514221917

592441454740433451665623

111416101156535177

532526243638554827574922
000000000000

000000000000
532526243638554827574922

532526243638554827574922

423220397943
215177171143173176106128140280215216

000000000000
19516915012916016487105125251194194

000000000000
19516915012916016487105125251194194

19516915012916016487105125251194194

001010000000
1661812111216148201719

000000000000
1661712101216148201719

1

1

2

1661511101216138201719

11
00

0
1

1
0

1

1
0

0
1

1

000000000000
261834332113192819251324

0
1

1
0

1

741131261041342
000000000000

741131261041342
000000000000

741131261041342

000000000000
19142330201113181412922

000000000000
19142330201113181412922

12

18142330201113181412722

344150247154215188314367388359334233
331225122140372835576111

11
00

00
11

11

141638359745712815816711512187
000000000000

141638359745712815816711512187
000000000000

141638359745712815816711512187

000000000000
461622202919374147413354

000000000000
461622202919374147413354

461620202919374147413354

2

481634282219445644685725
722121553643

000
111

111

726557823319135
000000000000

726557823319135

2232125351
0000000000

2232125351

000000000000
1216761636772

1216761636772

7
0

7

10108151110613186
13951289242022292414

31211768843

73364648825

74112544
00000000

74112544

692068263228627780685645
000000000000

0
1

1

692068253228627780685645
83176631619171056

23414686202225231911

381337131819263638353228

000000000000
7231493725661510611

7231493725661510611
000000000000

7231493725661510611

91
00

6
0

6
0

6

0
1

1
0

1

21
00

0
2

2

0
1

1

002000000010
613165272014375567745625

004000000000
54255314128325052524024

0
1

1

53251814118324951514024
16157352152224888

233133321079710

1471083314161933236

11112

41
00

41

000
1511

1511

0
1

1

8
0

8

13
00

13

4
0

0
4

4

000000000000
7661386551522151

000000000000
7661386551522151

7661386551522151

1
0

0
1

1

633230195128645145536148
000000000000

633230195128645145536148
000000000000

000000000000
633230195128645145536148

423213

632830195128624845516045

00208000001100
242612931900125517293921237217662318429439571675

53232311431
0000000000

100
311

2
0

1

1

1
0

1

0
1

1

1
0

0
1

1

121241
000000

000000
121241

1

111241

0
1

1
0

1

3211632
0000000

0000000
3211632

3151

21131

0
1

1
0

1

62749273745265910835775806731068921540
34103017119710

121513172914272130232011
000000000000

0
1

1

000000010000
121312142610211917232011

121312142610211817232011

0
1

1

1
0

1

0000000
13245213

13245213

0
1

1

2
0

2

191914101817282234302121
000000000000

011000000000
191914101817282234302121

191813101817282234302121

00
21

0
2

2

1
0

1

000000000000
645377344925616786816934

1
0

1

645376344925616786816934
102000110520

33122671912242746303214

304148273013363940463520

0
1

0
1

1

912185749111717103
004000000000

000000000000
912125748101717103

912125748101717103

1
0

1

1
0

1

00
11

1

1

000000000000
634213047294459871411096344

0
1

1

000000000000
634113047294459871411096344

634113047294459871411096344

951529828233213107
000000000000

000000100000
951529828233213107

951529827233213107

22121533
00000001

11131
10020

1

1

1

1

000
112

112

0
1

1

0
1

1

0
1

1

11
10

1

1
0

1

0
1

1

12
00

12
00

1

11

3111
0000

0
1

1

0
1

1

00
31

31

11214
00000

00000
11214

11214

00
21

21
00

21

0
2

2

355288343288448898289247235676624359
000000000000

597983671091217170691017075
000000000000

597983671091217170691017075

296209260221339777218177166575554284
000000000000

296209260221339777218177166575554284

223377263043212827241027
000000000000

1322
0000

1322

3030101101820
21337726304321282721825

22833217221

16316623264119112311624

1

11
00

00
11

1

1

000000000000
701930153425475655878829

000000000000
701930153425475655878829

701930153425475655878829

00
21

00
11

1

1

1
0

1

1
0

1
0

1

001110011201
179479895380310672838179311831633322130331134

177077490478510542819175111311559317130091115
000000000000

177077490478510542819175111311559317130091115
000000000000

177077490478510542819175111311559317130091115

000000000000
1062287718102626129

106228771892626129
000000000000

106228771892626129

0
1

1

141826951224414722129
000000000000

141826951224414722129
000000000000

141826951224414722129

5722314951154
76748864742644543065574866616101078304

6667892685132
000000000000

000000000000
6667892685132

2
0

2

6667892685112
000000000000

6667892685112

000000000000
167141112921292333248

167141112921292333248
122322496230

00000000000
45121244171

45121244171

000000000000
115778514151329147

115778514151329147

1111
0000

111

1

00
41

41
00

00
41

41

2816149169282025251212
000000000000

2816149169282025251212
000000000000

2816149169282025251212
000000000000

2816149169282025251212

323131613411
785192245984967612369320666

17965714111018323114
000000000000

000000000000
17965714111018323114

17965714111018323114

0
1

0
1

1

2817467102237414661113622
52324218131921125

001000000000
21171289152255311211

21161289152255311211

2143644121013537126
000000000000

2143644121013537126

000000000000
302337113947412456463829

302337113947412456463829
000000000000

302337113947412456463829

000000000000
5839574653421019589998448

000000000000
5839574653421019589998448

000000000000
5839574653421019589998448

5839574653421019589998448

12
00

12
00

00
12

1

11

558360444322287274398509379728728152
255111115210

3581892821515069394619
000000000000

000000000000
3581892821515069374619

3581892821515069374619

2

000000000000
521347421312258252346458305687681133

000
111

111

000000000000
521347421312258252346458304686680133

521347421312258252346458304686680133

18214472541416510
000000000000

18211371541415510
000000000000

18211371541415510
001000000000

18210371541415510

3111
2000

0
1

1

00
11

11

0
1

1

000000000000
235218164454187171349412031

0
1

1
0

1

000000000000
235218064454187171349412031

235218064454187171349412031
000000000000

235218064454187171349412031

70288030448359167666637878081007932756
204381582260981129131689311880185522004022878266532272120249

324327408285368365250311369276247280
002000000000

18914720093159160103146158128159125
004000000000

000
111

1

1

1

2142303133204
564593496340377678695549

424282435739285566604340

12274316896125

1

13310210344961206570795810476
000000000000

13310210344961206570795810476

11717217317920119410410815712574140
003000000000

111170157176199193949815012172137
000000000000

111170157176199193949815012172137

0
2

2

000000000000
421332110107423

421332110107423

000000000000
1883313811435754231415

1314
0000

1314

1873013811435753191415
000000000000

1873013811435753191415

1929294138121418222816
000000000000

1929294138121418222816
000000000000

1929294138121418222816
000000000000

109165344141358

9201348581049238

000000000000
281223133766375887827

000000000000
281223133766375887827

000000000000
281223133766375887827

281223133766375887827

9457548181824131617
000000000000

9457548181824131617
000000000000

000000000000
9457548181824131617

42484361791281114

5291121912553

0
1

0
1

0
1

1

00000000000
951624252763

00000000000
951624252763

951624252763

000000000000
6757669131781085

1520103101022
6757669131781085

000000000000
211135210106651

211135210106651

3126214271412
000000000000

3126214271412

000000000000
91128191317927169127

11121211
00000000

11121211
00000000

11121211

000000000000
81127191217726149116

81127191217726149116
000000000000

81127191217726149116

113010110100
530317902050152428621372237123342452422944572946

521617321908145227951289219922122307411043382853
000000000000

521617321908145227951289219922122307411043382853
935574626557137122113677273

512016761811138727271230205320792187403342602774

11

11214232

27112652545

142122311

29632151518855671443817
865713972668317112114511811993

0000000000
832912128164

832912128164

494878565165846472666572
000000000000

494878565165846472666572

580645356042394253304411569159116869762962535700
384295252956352649655144

1
0

0
1

1

000000000000
119321013192350288108

000000000000
119321013192350288108

119321013192350288108

000000000000
403950202416323835535536

000000000000
403950202416323835535536

403950202416323835535536

742672168822183
220851659611296479379484336226151

11
00

11

583138164521394450514434
44922133213282433282524

112

81914194711617117

6312434911483

1
0

1

319673412857
00000000000

319673412857

000000000000
1444361625469429318418238154101

1444361625469429318418238154101

000000000000
114205421751756

114205421751756

611081149524676104132928592122
000000000000

611081149524676104132928592122
000000000000

611081149524676104132928592122

1913257178191623111216
000000000000

1913257178191623111216
000000000000

1913257178191623111216

00000
21112

21112
00000

21112

4812411531175353522066736
455323482216379258607615690809533389

14122691516412735403517
216000552344

7122431551216154

23635810711843

168333436885

214132774541

000000000000
43122772517644931193247

43122772517644931193247

43901157688794947431118955
000000000000

43901157688794947431118955

2391401457717596335352465351240190
000000000000

2391401457717596335352465351240190

7411293369784
000000000000

7411293369784

00000000000
120242112422

120242112422

7311224735371
010000100210

1262233111

65223451

1
0

1

000000000000
828313398172

828313398172

00
11

11

0
1

1

000000000000
333768212215242812462734

333768212215242812462734

000000000000
11111027626402820181

11111027626402820181

001100322101
136711971540102915211214139015311835165514781607

000000000000
11861041132790412881019102912331351147112911408

11861041132790412881019102912331351147112911408

170139193113221183343280470164175174
181156212124233195358296482183187198

111719111212151612191224

000000000000
582177212111381012

002000000000
582177212111381012

581977211111381012

1

000000000000
421229152411273937313733

000000000000
421229152411273937313733

421229152411273937313733

0012001200010
2618102151722544326302318

000000000000
562527314115763

562527314115763

211265131018383221231615
027101131300

1985310916352618161313

225211232432

352026813385240729412633290930303557453737263262
493785223026192030543251

4115378334414
000000000000

4112378334414

3

014243113639
12278143578586106101115176161121

1056912848765378848914311699

178117530271623274213

511321756553
000000100000

1

511321656552

329324933005228027762436272428493336421734713040
1286102143704

1

4

328624842989227027712429268128323309419734593030

1342331151185

5442141089576

42023378914117
00000000000

42023378914117

000000000000
135863312456511101738

135863312456511101738

722035510444132
000000000000

722035510444132

0000000000
4351212431

4351212431

711944710101115106
000000000000

711944710101115106

1279243171930271619
000000000000

1279243171930271619

67149241721141394871638172160
200136064633278435621981178620842113218622972601

16111383262
00000000000

16111383262
00000000000

16111383262

52422366951062
000000000000

000000000000
52422366951062

52422366951062

732736436393554627512110335
22723471411523

102011244461
3146121256791

2411123

1313

00000000000
642688476910107

642688476910107

572036222822383345958331
000000000000

572036222822383342958331

3

5632111342
0000000000

5632111342

0
1

1

000000000000
33383628541182

000000000000
33383628541182

33383628541182

142221
000000

000000
142221

142221

11
00

1
0

1

0
1

1

6576117257516714161452
19015022097138176149182196122147271

16476136681019412015716889116198
381832132625385954152723

9535654442545068713659148

329291119132367

28213092415211130152420

20172312127131814171721
000000000000

20172312127131814171721

523449535437489351587642645817695541
116289531215109168

14622712201111
00000000000

14622712201111

000000000000
2318351217161824149

2318351217161824149

21113
00000

21113

421131120221
152522142814222735162014

716224234784

42215112391922317109

000000010000
204162196147167121158190163322255186

204162196147167121158189163322255186

000000000000
174134132139161136205187247241255105

174134132139161136205187247241255105

11611313012312157160186160201133214
43112307112775

22101121

543431971375

45513950521916103423968

10121611147181928191513

50415156493010913811212666128

000000
311221

311221

1784081627242542172924
000000000000

1784081627242542172924
004020001011

1672971225211637152621

141136111

132133211

109322264240
1084276330362092270612238361015100791611551511

000000000000
2231351529264327452749

2231351529264327452749

000000000000
10612732299220742675119583097799686911241462

10612732299220742675119583097799686911241462

000000000
181313212

000000000
181313212

181313212

322635124141713103
002000000000

22121246461
0000000000

22121246461

112341221011942
00000000000

112341221011942

304456233615424347584639
122042431330

24293818299283136503333
000000000000

24293818299283136503333

11112
00000

11112

244212664173
000000000000

244212664173

2221222
0000000

2221222

1331121
0000000

1331121

257211311
000000000

1232111

134211

2896522871110
02000002010

2112
0000

2112

00000000000
26765216798

26765216798

0
1

1
0

1

181131781
000000010

111211
000000

111211

000000
812156

812156

171020613617151220236
000000000000

000000000000
171020613617151220236

000000000000
171020613617151220236

171020613617151220236

111
000

000
111

111
000

111

000000000000
312124101522534573433317

312124101522524573433217
3111261012104510

14121316726195320175
000000000000

14121316726195320175

0
1

1

000000000000
137987916141019102

137987916141019102

1
0

1

0
1

1

11
00

00
11

11

4553186612713742879399501063570482367
523552233120616986645532

000000000000
641455686111323

000000000000
641455686111323

641455686111323

1131346620101011235
682963323626927385767851

00000100000
24451233411

21132

43513211

000000000000
7384822712201063

7384822712201063

00000000
21211131

21211131

8485611023101995
001200000000

8473611022101995

1

00000000
42243354

1

41243354

8311136372114
18815935221216122210

112112

141121

11111

34251514743

51632853133

232214511938
00000004100

2112

21221456736

14687587398815
000000001000

14687587388815

892359955611920
4574130597648325373431585767

00000000
12311321

12311321

31017382215428
000000000000

31017382215428

11

233211175685
163440253221112316171821

49123232122

2172120228267213

8543510472773

11111

211023426681
00000000000

211023426681

000000000000
641015992943313721096

641015992943313721096

000000000
151122222

151122222

000000000000
91222714766226911

91222714766226911

9933108245757738191947747
61180414113432

000000000000
852142204347516065746134

852142204347516065746134

103120421230
6811262107139105

3831215411574

252111121

23372478310736
000000000000

23372478310736

28132221
00000000

00000000
28132221

28132221

7366104527848245235235616976
0013000215331

000000000000
8613447382579

8613447382579

221
000

221

11741111
00000000

11741111

000000000000
53103122676102

53103122676102

595659417139238219220464863
116951081426211162

13151211711231915191414

353538255420201174184162847

473389262530624161325032
133414615695101

0
1

1

1453526620212410166
000000000000

1453526620212410166

202549231518271428172425
1323202314521

2121111

551454165156106

121711161113178951117

131243202315101811222019
311221019149114151410

00000000
13112312

13112312

416631163446
000000000000

416631163446

0000000
51231211

51231211

5032502941376052511487138
000000000000

5032502941376052511487138
000000000000

5032502941376052511487138

11111
00000

0000
1111

1111

1
0

1

2712216714392437263121
000000000000

1
0

1
0

1

000000000000
2712206714392437263121

000000000000
2712206714392437263121

2712206714392437263121

1193512147313939381022
000000000000

000000000000
1193512147313939381022

1193512147313939381022

4294362942568268326192052580062917809904365776741
2912094568187249136235243262385273348

956035414171291110
000000000000

956035414171291110
000000000000

11

95592441316128810

1112

11

000000000000
122389101723141516131324

000000000000
122389101723141516131323

122389101723141516131323

1

3041305291048636662703817745468573
33101722142018304

000001000000
271716271510322329333916

27171627159322329333916

001000000000
267103175806220603644757690413542

267103174806220603644757690413542

2165843911416105
000000000000

2165843911416105

000000000000
5524231459346

5524231459346

1

000
112

1

12

0
1

1
0

1

6125012187182430222228
000000000000

000000000000
6125012187182430222228

6125012187182430222228

154113656345
00000000000

00000000000
154013636343

154013636343

122
000

122

2641481289128175142267379375360291275
7437299310924181416

0000000
26324134

26324134

0000000000
836721141610

836721141610

000000000000
3443237332642

3443237332642

35115322
0000000

111

35114221

62238344
00000000

2111

11

41227333

617096814413763
100100010000

316135515332

264733928431

0000000000
52672334111

1

1

526723341

0000000
12252512

12252512

0118000100000
25113531353414

21331125211

362521313

000000000000
19125411410172015161520

111

19125311310172015151520

20511538095132110213322300293241211
3243410124532

1721012057711597192277269248205183

1121122434413

333222114154

26879111210153622312823

34118110425002
453159016218487755694260

000000000000
292527910122264236512929

292527910122264236512929

311033141113
0010000000

311013141113

1

10190583172913171226
000000000000

10190583172913171226

11643358414
00000000000

11633358414
00000000000

1421135212

121122321

11

0
1

1

511612222732
00000000000

511612222732
00000000000

511612222732

0000
3111

1
0

1

00
11

11

11
00

11

1

000000000
2463425514

000000000
2463425514

2463425514

000000000000
11103451899171324245

11103451899171324245
000000000000

11103451899171324245

2102196821175184245224252323276228387
3343305435247221220201682451648076207739654245402

000000000000
5419512141614264150353340

5319509141614264150353339

131

11143130113142
12873751788966173153214145120108

1257148976846316715020814211099

21119123523267

0014000000000
2647260326506179715681273396842005481663347674755

2647260326492179715681273396842005481663347674755

30414065714816384125161139307276112
4628219433429292835336326

6280411027824

25211035810112855861319726621182

2520399219173920142513
000000000000

2520399219173920142513
5112042535324

000000000000
171720514772796195

171720514772796195

32743596544
00000000000

32743596544

399238537211313235341342376446429261
2085111284105151817

8779136101863236685210511858
000000000000

82731159674293054419211257
8779136101863236685210511858

3214532289122

24781462141

1

1
0

0
1

1

166104001000
331674171925414829394021

0
1

1

264479815312815312215
4011033831343

151231221

1

12622286626

1615515171041093

414313221322

16112273

000000000000
45112536972135

45112536972135

11
00

11

2195634106551
000000001000

2195634105551

00000000000
531221732771

00000000000
531221732771

531221732771

8640892949351077310216313474
41100024441534

1
0

1

11
00

11

0
1

1

00000000000
221011215331

221011215331

910151212819525191815
000000000000

910151212819525191815

46171782619453238998234
5623401428225442441139243

7514527511176

31913255333

14413283282024131711
000000000000

14413283282024131711

0
1

1
0

1

11465753211082757588725663
103000102111

9357572910261546255524141
9554629517566

82111107847

755247239557274626422734

1311844111

000000000000
208153821201331191421

208153821201331191421

000000000000
261643211136544877193317

261643211136544877193317
1814321993549375792816

6231115916751

2811243

11325485878864
000000000000

11325485878864
000000000000

11324485878864

1

97153107611692
00000000000

00000000000
97153107611692

97153107611692

1

711636112421184
000000000000

000000000000
711636112421184

711636112421184

000000000000
703749324340707884695049

33116136915474
703749324340707884695049

0000
1111

1111

000000000000
361914143522392317212826

361914143522392317212826

000000000000
30152312715254551441518

30152312715254551441518

0
1

1

100111111221
1171191981031261726083808077138

96343412910769
000000000000

0
1

1

96343412910768
000000000000

96343412910768

000000000000
22822355121484

000000000000
22822355121484

22822355121484

6162130297111128211392374
000000000000

000000000000
6162130297111128211392374

6162130297111128211392374

000000000000
444957674953144744483850

444957674953144744483850
000000000000

444957674953144744483850

000000000000
551726142622394536352746

551726142622394536352746
000000000000

551726142622394536352746
000000000000

551726142622394536352746

5436217282902114432325068952873
49941596769136413114664349852

32175711166222123351817
1117210305211

2111314341946
000000000000

1

2110314341946

000000000000
2915296142161717241310

2915296142161717241310

000000000000
12456452581718111124

12456452581718111124
5017111147461

6117215616673341

132232111422

000000000000
15110320647972688148191154138122

000000000000
15110320647972688148191154138122

15110320647972688148191154138122
4410220311340

62505111791371

1419714645902574140181138127121

272038102219363441312615
01726210175212

1711186101419132318127
000000000000

000000000000
1711186101419132318127

1711186101419132318127

10813263741311136
000000000000

10813263741311136
000000000000

10813263741311136

000000000000
361629112414435054526127

000000000000
361629112414435054526127

000000000000
361629112414435054526127

9484931183787
361629112414435054526127

646272111795101

14611585142126293217

7244741611112

3111112
0001001

00000
11111

1111
0000

1010
1111

11

0
1

1
0

1

21
00

21
01

2

4

171415118717144213

000000000010
182239191910374062201610

00
11

0
1

0
1

1
0

1

0
1

1
0

1

1574547142452
00000000000

1574547142452
00000000000

1543447122132
00000000000

1543447122132

000000
111111

00000
11111

11111

0
1

1

0
1

0
1

1

1
0

1

211
000

21
00

21

0
1

1

17173115136302638151310
000000004000

1
0

1
0

1

1
0

1
0

1

17172915136302634151310
000000000000

17172915136302634141310
0387514811221

0
1

1

0
4

4

31
00

1

3

000000000000
17141387526182312119

1711987526182312119

34

0
1

1
0

1

11

27212738141848225

00000000000
1220257671824144

121
000

0
2

2
0

2

11
00

00
11

0
1

1

0
1

1

00000000
22411112

22411112
20010000

14111

000
111

111
000

1

1

1

1018216551622142
00000000000

1765119711

91115143715131

0
1

1

112216613
00000000

112216613
00000000

112216613
00000000

00000000
112216613

112216613

1

000000000000
245249282224244203172197200232250158

245249282224244203172197200232249158
000000000000

245249282224244203172197200232249158

0
1

1

2311399

2129436683231
00000000000

00
11

00
11

00
11

00
11

1

1

2522324911

000000000
7641344222

7641344222
000000000

11
00

1
0

1

0
1

1

54344202
0000000

54344202
0000000

54343191

111

40
72

00
11

1

1

0
2

2

1
0

1

000000000000
27160285148194117851532022221110

000000000
512458213555

00000000
52458213555

1125
0000

1125

00000000
52457203350

1

1

32457203350

0
1

1
0

1

22156282144188109641171462219110

31111

0
2

0
2

2
0

2

1
0

1

158167921361910

36321652326

2

531

1267824123169884679154169710356489902301

167890111494021239892274555361208347413832824
92242673027716305774276726749171811775120382283721878921578

0
1

1

1
0

0
1

1

191411366819189821

000000000
4211112102

4211112102
000000000

421112102

1

11

121344317

00000000000
533242236415

1
0

1
0

0
1

0
1

1

221121
000000

221121
000000

221121
000000

1
0

1

00000
21121

1

1

1

11

1

1

1

511142124315
00000000000

00000000000
511142124315

00000000000
511142124315

511142124215

1

3

11

263211381335
131110110102

1

1

111111

1
0

1

116121
000000

116121

11111

1

1

000000000000
15873438315385161933613108266915592512829383542418

15873436315185151933513105266315552509828783472418
000000000000

5132433247541496
000000100000

312131

4132133234441465

0
1

1

000000000000
15823423312785121933213081265615502505827383382412

15823423312785121933213081265615502505827383382412
100020400010

1

1

1311

15803423312685121933013081264915482505827083362412

1

2

2211364367
1000110000

12

1122

23

1

1
0

1

1111224

12

1

71050661010320629556256712161219258210455036

000000000000
291636293121304834322336

291636293121304834322336
000000000000

291636293121304834322336
000000000000

111
000

0
1

1

0
1

1

1
0

1

000000000000
8917171088211315916

00000000
11114113

111113

41

12121333
0000000

11

12121221

11

000000000000
232225164637

1112211

1

231213142626

111
000

111

2112172255625
000000000000

2112162255625

1

1
0

1

0
1

1

0000000
2211121

2211121

0
2

2

00
11

1

1

1
0

1

21619122113212720171420

1

200141772130257083931029164530111684713517006912
000000000100

000000000
313211432

000000000
313211432

0
1

0
1

1

31221322
00000000

2221322
0000000

222132

2

1

1

1

00
11

0
1

1

1
0

1

244731686310190160281192161214401
199541692129256983871026163630041674712616916908

402629124125416754633964
000000001000

16

000
112

00
12

12

1

000000000000
17191112257112415321845

00
11

11

16191112257112415321745
000000000000

1619111225792414321745

1

1

1

17314138273833101613
00000000000

17313138273832101613
00000000000

17313138273832101613

00
11

11

0
1

1

1

1211

1

1

543273341433
00000000000

0
3

3

1
0

1

1

0000000000
3221322353

3211221243

111

11

1
0

1

0
1

1

0
6

6

000
111

111

0000
1211

1211

000
112

1

12

1

17074066192924908242907143326451424689614326425
000000000000

000000000101
17074066192924908242907143326441424689614326425

1413

17004057191224868238905142926301413687114216406
001310110100

2566152657613

1

16984052190424778236900142626231408686314156393

2

5754213961489
001000000100

5744213961389

1

2182154736
0000000000

132142313

14322

211

11

1

1

0
1

1
0

1

000000000000
44343421146618

11

000000000000
34343421146617

224332725213
00000000000

24321622211

211132

4

1

11141144
00000000

11141144

371133769462
000000000000

12111
00000

12111
00000

1

1111
0000

1

11

1

1

000000000000
271131659362

271131659362
000000000000

000000000000
271131459362

2325231

1

22

1

311142

1

2

211

1

2
0

2

58456350323945142738360106
000000000000

000000000000
2527503618332210641463668

21104020827129532312748
000000000000

211016576215510928
000000000000

00
12

2

1

11
00

1

1

000
121

11

1

1

1

000000000000
21915553214510926

21915553214510926

00
11

1
0

1

1

000000000
241520108027211820

000000000
241520108027211820

231520108027211820

1

00000000000
13915868868614

00000100001
1131112513213

00000000000
1131112413212

1131112413212

1

254743736511
00000000000

254743736511
00000000000

254743736511

00000001000
44112233736

211
000

1

1
0

1

00
11

11

000000
111111

1

1

1111
0000

1111

00
11

11
00

1

1

2212112534
0000000000

0
1

1

2211112534
0000000000

1

1211112534

33171314136233532372438
000000000000

33171314136233532372438
000000000000

33171314136233532372438
000000000000

131011553183125251515
000000000000

131011553183125251515

000000000000
10424423355611

1

831342335559

21112

1

1

1

923311126311
00000000000

1

922311126311

2111
0000

1

211

000
111

000
111

1

11
00

11

0
1

1

0
1

1

111113

4462759121691510

82624254143118

11

12
00

00
12

12

2

1

1

1

61617138425721110617445144

000000000000
311839965248089236671342832299910242350608842

2211433521
0000000000

2211433421
0000000000

2211433421
0000000000

1

21

211332421
000000000

211332421

1
0

1
0

0
1

1

5743314332322
000000000000

5743314332322
000000000000

5743314332322
000000221000

0
1

1

0000000
42211214

411114

11

12

15232111228

304839065198068235971332828299010235343603819
000000000000

1

304838965198068235971332828299010235343603819
000000000000

304838965198068235971332828299010235343603819
000000000000

1281176107912575069598379922604169210250

14671895401679818286155198119467605132370510

302439132519105226422359
000000000000

302339132519105226422359

1

000
1731

1731
000

000
1731

1

1
0

1

21

11
0

11

5

11

0
1

0
1

1

211513

1

60482163746371092791236044101
000000000000

0
1

1
0

1

4216418362
000000

4216418362
000000

4216418362
000000

00
11

11

000
121

121

1

216418062

000000000000
131115898624341916918

131115898624341916918
000000000000

1111
0000

1111
0000

1111

1
0

1

1

11114242
00000000

11114242
00000000

11

1

1

11112231

000000000000
5836422021152514

5735421819141212
000000000000

3734421719131212

2111

11221132

531232262723
000000000000

000000000000
421222252623

411212252513

1111

111

1

1
0

1

111

111
000

111
000

1

1

1

115111
0000

0000
115111

151

1

1

1

423758263731216242443583
000000000000

423758263731216242443583
000000000000

1271951111421817928
000000000000

0
1

1

000000000000
12717589420713826

11

11717589420613826

0000000
1321412

1321412

1
0

1

00000000000
165141732314

165141732314

11
00

11
00

11

623181234244
000000000000

11

4272133
0000000

4272133

11111
00000

111

11

111

2

1
0

1

11

11
00

1

1

1

000000000000
20212720171442014211837

000000000000
20212720171442014211837

20212720171442014211837

000000
210101211

000000
210101211

210101211

311111
000000

31
00

31

1

11
00

11

0
1

1

112111331

13123423

12312

1

123175222

00000000
34311722

34311722
00000000

00000000
34311722

34311722
00000100

21
00

21

11

3421411

1
0

1

1

11

000000000000
2224321325261215651128

1
0

1
0

1
0

1

000
111

000
111

111
000

0
1

1

1

0
1

1

000000000000
2223321324261114651128

2223321324261114651128
100000000000

31628610206133
0000000000

0000000000
31627610206121

1

21627610204111

12

112
000

11

1

1

000000000000
1874713551335198

130
00

1

30

1
0

1

1774713451332198
000000000000

1774713451332198

1

1

1

1212

00000
11112

00000
11112

11112
00000

11112
00000

11112

722157983262
010010100011

11

11411

121

1

5112563131

111

1

11

31

1

0000000
1221262

21
00

00
21

00
21

1

1
0

1

0
1

1

000000
122161

000000
122161

122161
001000

1

0
1

1

0
1

1

00
13

3

1

23

11111

000000000
111111411

1

111131
000000

000000
111131

11131
00000

1

1131
1000

1

1

3

0
1

1

000
111

11
00

11
00

11

1
0

0
1

1

21

11

131

9257523272559017111814395121
000000000000

69483824594772131941197077
000000002000

2113961013103717301326
000000000000

11112222
00000000

1001
1111

11

1

1122

1

000000010000
20138591383515281326

0
1

1

1

000000100000
18138591383315281326

1

1

18138491373215281326

1

109125225284441191628
000000000000

000000000100
10894224254137131227

3121
0101

1

1

21

11212

55331812332307525
55331812333317525

11

11111322

1

1
0

1

00000
11112

12

111

11

0000
1111

1111

0
1

1

1

11

1
0

1

111211
011111

1

1

1311324641
0000000000

0
1

1

1311224641

0
1

0
1

1

372412132729344834684122

000000
125221

125221
000000

21
00

1

1

1

1

12

3

21
00

21

1211

238148118174023242544
000000000000

000000000000
207148107143219232444

000000100010
207148107143219232444

12
00

12

0
1

1

215141

1

185845562312131334
000000000000

1

185845562212131334

1

226231624968
000000000000

226231624968

2
0

2

1

000000000
311138411

000000000
311138411

000000000
311138411

311138411

1

198424092513309126193119522916961774205320922383
000000000000

198424092513309126193119522916961774205320922383
5635362221391008351574986

0
1

1

1
0

1

6155243861
0000000

5135
0001

5134

5224335

1

00
11

11

1
0

1

1

1

0
1

1

00
11

11

1211112

1

22

1

4212155313

1

4215

1

1

111

1

2

0
2

2

11

1

1

0
1

1

3613717184171812746723

1

1

00000100000
52212362476

2

1

0
1

1

000
112

112

1

1

1111
0001

111

311115144

0010
1111

111

1
0

1

1

21115223
00000000

13

1

000000
211142

21132

11

0
1

1

42113311
00000000

42113311

1

1

11233121

0
1

1

1

1

0000
2111

2111

1

1

000000000000
2071814151026222220819

2071814151026222220819

1

11

11

1

1
0

1
0

0
1

1

1

1

1

1

1111

2
0

2

1

2

1

00
11

1

1
0

1

143

0
1

1

01
11

1

1

1

1

1

1

1

1

1

0
1

1

1

00
11

11

13886

0
2

2
0

2

6

1

1

0
1

1

6

1

122131328

62

32141911
00001000

1

1

2

2

4

3

4

1

1

1

1

000
241

21

4

1

1

3

1

1

1
0

1

481416122013232032284420

1
0

1

1

1

21421123

268114147171219372117

5432585635517
000000000000

5432585635517
000000000000

5432585635517

43134391054954

2

211312

41123
00000

41123

0010000
3132339

0
2

2

212139
000000

212139

1

00
11

11

0000000
1141111

1141111

000000000000
5352341622310

000000000000
134111131214

134111131214

311222114
000000000

311222114

00000
11112

11112

932341191359
0001000000

1
0

1

2
0

2

632131071349
0000000000

632131071349

31
00

31

0
1

1

1

1
0

1

1

1

11

00000010
24211122

142112

0
1

1

11

1

1
0

1

11

1

111

4

2518157411122621192029
000000000000

12154216

1
0

1

1

122

1

000
111

111

1

211

0
1

1

211
000

211

2121159615

000000000000
18161143101138111222

18161143101138111222

1

1
0

1

11

1
0

1

1214

1
0

1

1211514145

5254342425513

1

0
1

1

1
0

1

000000000000
242427152221133714172551

111322113

232426142218113514162448

0
2

2
0

2

1

0
1

0
1

1

1015411341626
815410341626

21

1
0

1

2231

00
22

22

1

2112

11

16402231230829642450295995813271427167817751989

1
0

1

1

1

1

101013

1

0
1

1

0
1

1
0

1

1

1

1

0
1

1

1

23714263282210101338
000000000000

00000
11122

11122

000000000000
23614263272110101136

23614263272110101136

1
0

1

8
0

4

4

1

1

1

1

1

1

1

1111

2

111

00000000000
1534129617585

1534129617585
00000000000

00000000000
1534129617585

00000000000
1534129617585

00000000000
1534129617585

1534129617585

15312721

1111

554203616235619756311271109817120539783235548
000000000000

000000000000
554203616235619756311271109817120539783235548

11
00

00
11

00
11

1

1

000000000000
554203616235619756311271108816120539783235548

1871211528562831214
00000000000

1871211528562831214
00000000000

1871211528562831214

000000000000
536202916115619755211221080760117739473214544

00
41

1

4

002010012110
536202916075619755211221080759117639473214544

1

11

510201615855611754411131069730115239083189490

1131

261220879102821342254

1

0
1

1
0

1

1

1

1

1

11

1

1

1044424516739355732355047

12893444121171413

11

56135124612211

1

1

8514

119888011620461330456102189213369671204932108847311705671230114158446813618721231363

17511211

4991427779632464335603184129130134082133986197614759827416933408
180959449179923527806271304984191399045644665524

1

6146454451273

5

199278229180199183246223314199207199
0112001102010

105113867078531007610892115100
100001010000

27142392921141240213347
000000000000

25132372821141240203347
000000000000

11

4021000215010
25132262821141240203347

11314

14982141166682036

211

1111

1312

1

1

541992782

1

1

131111

1

1

13

2

1

112

511

1

1

1112221

0000
2121

211
000

1

2

1

11
00

11

1

000000000000
576142463212433238465129

516140453011402833404829
000000000000

011020000112
516140453011402833404829

5111

1

13106151025121098

1

610131041311574

1

1

11

1

224153333

1

1

111

1222

12122

1

2011

1113

2

82621161010101

1

11

1

1

11

1

446392234415

1

5122112

3122148212

1

1

111

1

1

1

11

1

6212134563
0000000000

1
0

1

0000000000
6212134553

322

3212134333

1

000000000000
193821141618433130243124

000000000000
193821141618433130243124

000000000000
9911131213241821192421

9811131213241821192421

1

2712111
1110000

2

3

1

1

101

1

1

1

0
1

1

92914414128473

0
1

1

12
00

12

111

1

7111
7000

0
1

1
0

1
0

1

11

313640102516304654472821
18159611913111613913

010000355100
122030314617343434198

1251112
000000

1111

1

251

1213321
1000000

231

2

1

1

11

11183211513252731188
030040020142

82215919162063

1

1

1111

51433511

21

1

1

24115631

3

1

13

0000000
1111114

0
1

1

0000
1111

1111

00
14

13

1

6311991999511111197142586377

2244811

2

1

21

000000000000
725157144136159225201285148

725157144136159225201285148

6

2

13

1

2

48170161214424332442364

311

1

1

144

1

128

67151462577133
000000000000

120100010000
67151462577133

45131462457122

11211

1

2

17161464816714201313
000000000000

000000000
1211116211

000000000
1211116211

1211116211

5111

17112

7113245

000000000000
91182337163514412

91182337163514412
000000000000

1

91182337162514412
030300000000

2
0

2

0100000000
9223416251235

14

9341624735

71

14

912317
00000

1

912316

2

1

85259684532100756977474039
1330586193438455504277260746025273542514287130334784

111

3131834583756443026082333382639394639485234533048
395848961421929332943314425

1523
000

000
1523

4
0

4

1123

5945111330252
86422128917538328706116386621056819997

123
000

123
000

123

23461671710584334473013
000000000000

0
1

1

35110110931171
23451671710584334473013

1

1

3

1074136937567

211

2

1312826192511

3311

11111

311111

111

11

11

1

1

2

131

1211

1

6111

11

12

1

1

12513

7

11

0000000
5415313

5415313
0000000

31

321

21

21213

187678300144209181142132139191231154
121402421410910141

122
000

12

2

40197170477739574147475329
2696110532230

3131

1

611113

3

11

1

41

211

11

171

11

14

1

111

1112

17152116211116197221317

61

353123115134844

11

3112

621124921221

4

3

121

121

11

1

1

3

2

2111

1

3921

11

120111

3

1111

21

1

1112

2111

11

11111

1111

3262511125442325

1

111161

1

1

11

111

11

5

11

1

1

1421211

11

511

213

421

1421

195351164599967
13227312490124139807882129164121

1

16

311

1111

1112

131

1

11

1

23221213223

1

1

21

1

1

153122834

83465784107212

8

13

1

1

1

11

1

113

5

1

11

221

1

11

11

11

115

12

4

76333

712211

1

14

263262434647192831325443

1

112

3

11

1

2

1

1

2

14

1211

3

11

112

811

414122226

116

13811

1

13

12111

2

2111

224375233

1

1111

113

20311

115114343

2

611

1

21

11

411

1

1

421924124567161615456826

11

001
6211

62

1

00000000100
35224213143

12

1

4112

31232141

000000000000
6308929161981181

6308929161981181
000000000000

6308929161981181

0
1

1

1113
0000

1

113

5724766551222715
000000000000

4522664451119615
5724766551222715

41

12131

2

6

6

1

3

1

1342000000200
100537118799687102131115138114119

1

3

814
000

7

114

5117
0000

2

7

2

111

3334045362941647665733641
49611123685132073

11

7

2

211

1

31

3

52110348521013

21

4

1

1

6

1

11

742312

2

11

1

5

31

1

3315253322

1

12

25126432112

361061

14

12

1

1

1

1

111

3

1

2

1221133

311

112130161

11

4

311

1

1

4

1

2331122236

1310391231721251919

1

11

10911

15111

222

31

2

452933203817243736365539
000000000000

7

452233203817243736365539

2111738222829141814261635
0184300301010

3

113

1

1

211

3

3

4

4324331355

1111

111

111

1

41

111

3211

1

12

1

1

18

2

2

1

1

8111710121755410517

2

1

11

5

2111

21

111

1

374421323

91111

2

11

21

1

11111

10

21

1

111

111

11

11

2

4

3

111111

124

00000
41111

41111
00000

41111

537743435506499574280301353638423690
040000000000

537739435506499574280301353638423690
6154812158161225362125

11

1929112236212

1

22

222

213

1

11371

121

111

25

1

1

12

2

11

11111

11

13

1

14

2

114

2311

1121

1

91212

31112

11

223571

1

11122221

131

11

111

21

427111

9111

114

1

1

122111

1111

1

2111

133122813

33111111

121

111

31

13

416125159921159818

1031

21

144

1

1

1111

2

1

12

1353916364435

171115337

33

71

81

1

41

111

312113422

111

6

11

21253

2068310114316322717112316915812881

1111

1

2

22

2

11

1

2231

278316272300248280235023332197478

1115

11

1

212111

127321218153164

1

121

1262132722116

1

2131342232

11

2

3

1112131033

11

11

2

314

1

26

1

1

1

2

1347639152913

1

6172058646524601515744770837774667502
000000000000

2

12393200100121
6172056646524601515744770837774667502

318725192610293429355030
0422011000010

21111

1

221

113

2

141

3

11

111

22269154202010193122

42

4515473710136143

8

1

1

211

1

92

1

1113

11

11

4

1

1111

1

1

5811711612501570503705724800733611467
10076512310611212811912411015612492

6

111

21

211332235

11

1

11

311

11

1

11

2

1111

21

11

11

2

311

2

11

2521211215

211

131

2111

2

1

111

161148333

42244431513

2111

21151919421

1

111

111

261

1

1211

1

1

1311

101111

3

21

4121

2

1821117

3

3

111

1

1

141

211

1

11

111

11

21

1

1

321111

1112

111

71132763

1

1

11

1

11

1513113

111

11

3177817222714117149

1833254142115141310

11

1111

112

11

111

1

2

11

11

21

1

1111

11

1

11

11

1

232131121

221775322

21121

1

21

11

1

4

1

2

11

1

2

1

31

11

111

2

1

643727123512111810313971

1

4

3

211

2413291414172312193

3

111

1

12

1132

1

1

121115

1

2

1

1

2

1

1

21111

1

1

1

1

1

211172

1

11

4

42

11

111121

1

1

11

1

1

1

152032465241573659201336

3

4111

1

1

111

1

1

1

1111

1

2

1

11126122

21

31

1111

1

1

1221

4

21

11

2

2

1

141

3

193

111

1

1111111

1512

1

1

14

11

21

1

1

11

1123

13

21111

12

11

2

16

12485811473678

4

11

211

1

11

1

2

11

1

1

21211

1

1

4

642222

1111

1111

1

1

1

14281181613

41175821

16112213

11

23341

111

11

31

11

11

115

1

1

11

21311

1251361

11

2

11

211

111

1

3214

18

11223

1

211

1132361

11212

1

1

221

1

31132312112

11

11

161465252

1211

11

4285112024825257164218

1111211

33

11

11

2112

1

11

111

1

21

111

1

1

1

22112121

11

1

2

1

1

11

111017131

1

111

1

11

1

1

2

11

1111

112

11

1312114

1

11

112

11

1112

4

111

12

1

1

1

1

2

111111421

2

1

31

14

1

1

1

1

2716312014814241752315

1

1

91213211

14222131

11

1

1

11211

1

1114

11

311

1

2

1

112432

1

132

1

211

1

1

1

1121

1

1111

2

3122112

21

1

1

11

11

1

14

112

1

143121

1

212

2

11111

7

15135724311

11

11

11

1

22144211

111

1

31

1

2

111

3447172144473423

2

1

11

1

41

1

6511

1

4

1

1111

1

11

11

1

2

11

1

11

11221211

1

1342211737

112

11

1

11

21

4121

11

11

1

1113223

2

67821257270541171371921467737

1

2

11

111

42

12

11

1

1111

61

2513141

24182435171211341327225

41111281

1

113111

1

2

3

12

2241221125

3

2

1

1

1111

1

11

1

1

1

1

1121311332

1

11

2

11

1

1

211

1

11

12271101

12

11

1

21

1

1

5

111

2

11

12

11

1

211

211

1

11

7111

11

1

1

111

11

51

2121

1

1

1

222

2211

2111

1

1

121

31286611

11

1

2

1

1

2

1

11

7

1

12

1

2121

113

7

1

1

1

1151

1

1

1

151

41512265435

12

1

111

13

1112

111

1

2

111

2

1

2131

1

11

12

715122121222383251111

111

111

1

1

2519347163

3

1

1

3

11

31

1

11

111

211

1

11161

1554221311

2111

1132

1

1

11

1

1

11

1

1

5

10131

1

1

1

2211

1

22112

1

11

1

1111

12

1

1241

1

1

1

11

4111211

121

211

32

11

1

11

71

111

1

6241641522

1

1111

1

11

11

11

1

2122

11

18132
00000

111

8121

33351794422
02000000000

1211

3151783422

2

111
000

11

1

0
4

4

00
11

11

21113111
00000000

2131

1111

111

1121
0000

1121
0000

0000
1121

1121

0000
114151311

114151311
0000

16

43
0

43

2

34107

295

75140611
22223400

3

2481061

1

3

2

271651

11

1

2637415111024

2

1

1

000000000000
3325776432634548852455581

2

1

1

6

2

281

2

3316876402634528852455581
03126000000030

4
2

1

1

0000000000
1623181444

1623181444

13
00

1

3

49
00

49

71
00

6

11

167118291229356834333460
4110101071521

11

1

2

21111

1211

11

111

1

1

1

114

131121165

2

122284

15

61

2

1223

322

62681711418451310125

23236

5786712685622

12

2

111112

11

1

5

1

121

112

921112
200000

721111

1

8
4

4

495238327
030000000

1

11

11

1

111

1

2422376

21

1

111
000

111

102112
30000

1

1

2

1

11

2

111

1

2

961248410932102
9612484119331010

11

8

2
0

2

1
0

1

4422

1

1

1

000
1731

1731
000

1
0

1

1721
000

1721

1921111
0000

1921111
0000

2

427000
1919111

37511

779

131

77

4

234

10

68

2112123112
000000000

000000000
2112123112

0400000
2811111

1

2

211

1111

11
00

11

0000
3221

3221

23142
0000

0000
23142

23142
03101

241

000
192

000
172

000
172

152

2

0
2

2
0

2

1

00000000000
5584531051551310527

0206913271100255
5584531051551310527

1
168

167

12

4372213

195915732510
9560330105

4

918142144

1

2

3

5610111

6

101

1

2

00030
21421423

2512

4

1

3

141

1106371

0128921022
210688732139

641

1

1

1786694

133

6941111

2

1312

2

13

7

1

1

17221736142574119132200148181272284154
000000000000

0205524400000073
17221736142574119132200148181272284154

5773615033461095311617012049
1190000001000

1

2

24111111

411

2

1

2

4

5432584931411065111016812047

1

3

1

3

113

11

12

1

23324211240142
3552833275034545031474457

23252131113547

1

1221

11111

1

2

4

1

11

1

112

1131

2

2211

41111

1

11521

14011

1

11

1

1

41111

111

1

3111

12211

31

11

1

21

312

21321

281220173319271913283043

1

134111

1

00
21

11

1

0000
235111

232111

3

3691
000

3691

0498072000
117464194241641

116913193521641

4

3

3

2

20

15

6

111
000

111

62234137121118146
010000000000

3832151191373

3

1

3

1

111

32211453

2

11

21

11

5

28561753141415
28275500020312

41

8

4

2

112

51

1

1

1212

5

51

10

3

1

341

1

1521

1

1

21

2

1

3

3

2

1

19

111

2

9

2

1

0100000
2421133

211133

211

645254119101458
000000000000

635254119101458

1

2
0

2

13111
0000

4

2111

2

5

0
2

2

060000000000
11592434215232

171222115231

21

144111211

19212124
15001013

11

11111

4

246311281
0000000

2

2211241

1

1

3

4544

41
00

1

31

11112
00000

1112

1

338381117281515271510
0380400000000

221

127132

2

1

3

1

3211871626151251410

2

000000000000
25852725332325332120943

25842725332325332120943
000000000100

113111
100000

13111

8000001
121251125

3

119

11111

51

4

1
0

1

8159351291995710
25702725322223301517817

4

11

32315112

1

111

11

1

1

8

1

2

2

1

111

112

1010141825966282

1

1

1

1121

1

1

3

11

112

1

153842512141311229131019
010000000000

1

31

01990000000000
153662512141310229131019

00000
167151

167151

00
31

31

050000000000
12892011127916411819

1

2

1

561

1

1

9

12142010127916311819

2852611421
0000000000

2852611421

10

3

000
7711

7711
000

411
000

2

1

21

73
1

72

1

80024451573605539413110910011231690461476
341408915113291332

3556782571330172211
38620229199329212103123180187145109118

000
191

191

1

289211423133
67101300032

2

11

1

1

5

1

1

21

312

7

11

2

3

164

11

11

2

1

2

2

2

1

0
1

1

0000
149111

147111

2

0000000
43511112

43

511112

00
23

23

00
115

2

17

6

1

1901211
1870210

21

1

1

4080634302025194544321823
72166960464843467588765140

11

1

1

1

385

1

21176

611

1131561

11

6

2

334132131

711

1

22

22112

5

1

1

11

2

2

1

2

1

331

171111

32

3

1

1

1

88111372161

12212

1

231

2

1121

2

1011421

2

4

1

2

1

13

21

1381232

1

1

2

1

1

481

1

291

11

1

111

31

4

2

1

3

1

3

115121

1

1

2

111

131

1

1

1

51

1

2

1

412

1

1

1

1

11

92111

21

1

23

733423232

12

11

1

11

126111

17514

1

1

4

12

1

7

12

2

1135151

1

1

4151

2

121

1

12

112121

1

121

175

12

211147212

272

18

3

6

5611
000

1

331

2

201

2731511
010000

245211

2713

1

1

00
41

1

4

2506520025510
413211944896615

51

349151

212

1113663

1

1

1

211

2121

1

4111

2231
201

9

9

1

1

1

1

1

115424211
00000

115424211

600000
1523111

11

313

611

8777510625232010103
0320310000000

711

1

11514181

1

7

11

111

1

2

63375692981

1

3

41

321

2

21

121

1

41

13

31
0

4

1

26

3720012
45724213

420421

1

38212
000

38212

160
3241

111

1

5

21

0000
2211

2211

0
5

5

000
43311

43311

040000100
21002511251

1

4911

81

1

1

1

3

11

324

1

2114

0
1

1

3
0

3

1

9155323554260200210
1917215691097929313748351842

1

1

2

3

6

156

1

56

292

20212

43532484127253147261541

56

62

20

25

11

1

11111

16

5

1

2

1

4

3

511

1

27111

1

19

12

2

4

2

2

1

4

1

34

521

75

1

1

316111

11

2

1

21

217

11

3

2516132

1011

1

1

11

10534341111

121

8

180000000
2612116251

11122

342

1

4

31

4135

1

1

3

2

31
00

31

161
191

3

6

1853
0000

1853

10410511111113
016000000000

3

5

2113

47

1

5251

4

21111

15

10263

00
61

61

2

11
00

11

84111
46000

9

2

1

1

31

151

4

2

11

110
2121

181

3

114513514107942147
71112513107942146

23

15

3

11

13

7

1

31

1

1
440

1

2

423

8

5

00
141

4

21

2

3

3

100
1322

10

222

808
00

808

1

1

646151113
100001

63014

1112

51

9

1

38010000
103115122

711
000

1

1

1

6

0000
4111

31

111

000
841

2

4

121

12

461
40

1

14

1

151

4

1

6

00000
1143272

111

142172

000
521

200
521

32

1

1
0

1

3

10

11174014311258144
260100000000

5
0

5

000
121

21

1

1081381321132144
2223611000023

10841

67

3

1

6

1

1

2691312

31

35728

21

1

2

3

1

2

1

4

1

3

71

202

1

1

11

3113
0000

11

213

11112
00000

11112

1021
00

981
00

3

1

1

94

4

13
0

13
0

13

0000000
44311213

44311213
0001000

253

8

1

31

1

31

4

211

181128
000000

181128
000000

161127

1

2

276706217159248260193182238397255200
95616021656831

2

2

15030613610618218897949619217674
2686553521435

1

1

231121115

2121613291952225

21031243

21314111

1141

11267827012013335453614713036

21

1

1

2

121

1

111

111

1111

111

1

2

1

21151181216322940161712

1

1

5

1

111

2

2

1

11

1

2

1

1

12

12

7

11

1424137510102

121

111

112131

11

1

8

1

1

711

1

1

111

11

11

2

1

4

1

1

18311

1

311

17111

812

1

3

1

1

1

111

111

1111

1

11

1

1

1

1

1

114

1

2

32111

1
0

1

510592314131726
043220121002

4

16

1

151111

2

91121

1

1

12

2

2

3

1

1

5

1

2

211

7

2

4222971

32

000
111

111

0
1

1

100
121

1

2

3

0
1

1

7
0

6

1

102432022213668614
1594932283768941136

13

2

72

1

1

1

14

12113912

111

1

1

1

2

1

1

120127717413

1

2

1

15

2

2121

241

3

1

2

1325321

010000000010
32322313262813117252630

21214513161314

3088512121144212113

1

1

51

111

2221

2

11112

111131

1

060001000000
61535422952314622

1

1211063

1

1112

13285216

225120211

21

1

1

127614

00011000100
214214737393

222327272

2

11

31

11

11

1

3

1

311

1

1
0

1

1

1412111
0300000

1

1

1111

1

1

814421235811
03200020000

1

3

11

211

12

11

1

1

1211

1

1

1

11

311

1

315

4545291723276149253027
0815000000000

4533141723276149223027

43

221172
000000

221172

1

11
00

11

1

371261618195716545699192215
000000100000

37581617195715544699192215
961000662505631221

1

11

2

3

2326532521

21

1

1

2

2

1

2

1

1

12

11

3

2

813

2

111

821619

261061014346306511172

6711
000

6711

1
0

1

373131124
00000000

4231124
3120123

1

11

11

3311
000

3311

113253323111081610
000000000000

1028521228961610
000000000000

1421

24

241

1

11

101122875126

111

00000000
14121312

11

21

1

111312

0210000000000
103771061181056916

2111
0000

2111

5639410363448
000000000000

5639410363448

1
0

1

1412
0000

1412

0000000000
1111143225

1111143225

010100
142232

1

1

11

1232

000000
1011211

0
1

1

41121
00000

41121

51

2

7022272
900040

55222
0000

55222

6212
0000

6212

120000000021
41021283610261742447

394118368231536244
136320201161504

112317

13111

21

32211

33

4111

1

15121910

113111141

113

1

7126

2

221

53212

32111113

1

1

7126

211
000

211

0
1

1

3113252
0103001

11

1251

1

1

154331
00000

173

2121
000

11

202

7
0

7

0
1

1

000
181

181

0180000000001
399053343520453651312462

42
00

4

2

000
142

141

1

000000000000
15915215426193491415

1

15815215426193491415

1

00
21

2

1

22473732181519161619946
040100521600

144156410511854

6

22

311

921

614342512112533442

1

1

1

3

32

1

1
0

1

0
3

2

1

000
111

1

11

121
000

121

1

3732
00

00
3732

3732
10

3722

7

4

3481515164947727916067284111071801929607
01623110025001

7151
0000

7151
0000

7151

2

0000000000
29181453757313016

29181453757313016
1902000200

12

11

254569127

111

2111

1

1

1

11

1

1

114

11111

1111

3

14957811012040
191401920618014253498473711217

671
00

671

111
000

111

0
1

1

042909812001213371
14121051941469213287432410610

1677111

11112

199713311114

16122131

57141

234111

851

1

13613

3

312

18522

461

7

22

119

2

79322115

305121

1

1

23312

541122

2

1

2

1311251

6313

1

763115

2

2

23122

562941020206796

1

1

2131

511

3

23

1

640116

329141

2

11

72016

481

901131

1

1

25

21

25816

1

2

472

1

8

211

171

1

181114

217

1

1181

1

331

1

77

634

1

1511

12

27851

3

5961

221

1

1411

2

1

3711

1

221481112

83231723

3483

1

9111

11811

73

30417

43111

2

02718100003
458424214187

3

363111154

21

4

3

141

95

6

0
4

3

1

241

2112
0000

2

112

47521

000000
77412121

2

77212121

1323
1103

1

12

30094343929626013463373810481186686573
0810000200010

0120000000000
82556641652014412

111

1

2

78334453161238

11

11

11221014213

6001
6211

1

1

1

3
0

3

0500000000
3921222123

11

1

2212113

112

3

1272135

0
1

1

1
0

1

151022163011212729192026
000000000000

141022163011212628111926

11181

2409162625205
166600030

113

1101572

2

1138454629

00
11

11

00
211

101

1

10

100
1021

21

9

2725013142102171175886999771150650529
197326294015474574139728

31038114151012191619

110311

11111417

249417250170173100535642877988561494

7

1
0

1

00000000
731212121

731212121
00000000

00000000
731212121

42122

1

31211

10231247715765748575607743
060000000000

48054534113651
060100000000

00000000
81232121

411

41211

1121

061100
1521323

4

1

3

12

1

1

1

1

1

1

10

11

8

3

13

1

2

7

2411
0000

2411

0000000000
11041148122

3

11221

17314611

091000000000
8711039635058676772476841

0000
1113

1113

0
1

1

11
00

1

1

1
0

1

121021301210
837133604848646465446130

2811

111

1

2

11211

113

823628584445576062405430

1

1

1112

2

11111

1

000102011
1132213321

2

1

1

5121

1

111

3

2

1

2
0

2

0210010001
1831734176

3

121724175

1

1

1

1311
0000

1311

022000000000
11116342437741

00
41

11

1

2

0
4

3

1

0000000
2112111

11

211111

02700000000
66813221453

2

11111

1

11

1

1

1

1

24

111

11

331112

1

11

1

11

21

1

1111

1611211
0000000

1

511

1121

45
43

2

5

000000000000
101645601328070288272392806396

101645601328070288272392806396
031012910110111

010
311

1

3

5517933334440267240360432764
41102155121055824

1

1611

1

21

51

111

1

1

112312133114

11

1131

1

1

1

23

1

1

1

11212

2

11

112

5

2

1

1

1

11

1111123

1

486911613283221

61

31121

1

211

2

1

14111

3

11117

1

4

1

1

1

1

111

111

3

1

3767193191081323

1

1131

1

1

31

23178746314

11

111

11

3

21

1

21

1121
0000

1121

713149211
000000000

11

613149111

1022
000

1022

1012
1112

1

0
4

4

1

22

000000000000
38328941455181519

121111

3711794145481519

000000000000
498323891351036

498323891351036

25329431211082
0182331001000

251

11

1

11

3

1

1311112

1174

1111

1

3111

17211
00000

7

11

111

171233612362
01000010001

1121

21411

1

111

214714

1

1211

2

3911
000

3911

0
1

1

19112
19000

112

000000000000
505258223936415140392032

3038461520282326248624
000000000000

3038461520282326248624
000000000000

1
0

1

16131
00000

111

2

3

31

000
111

111

112
000

112

01000
11222

1

1122

0
1

1

11
00

1

1

13133211742
00000000000

11123211742

21

272744101624211919220
00000000000

272744101624211919220

201412719818251631148
000000000000

23

1773386818131982
1022362711111082

00
12

12

51513

3
0

3

00000
12126

2

4

1212

0
1

1

31
00

1

21

000010111013
3794927731266

21

00
12

12

11

2473826621243
011000000001

223311

34231352441

12171

4131
0000

00
21

21
00

1
0

1

2

12
00

12
00

00
12

12

000
111

111
000

111
000

111

10111232311
0000000000

10111232311
0000000000

1

0000000000
9111232311

1000000000
6111232311

1

112111

21

31131

3

00000000000
45212337121318

114

12100101200
3261233741218

5
0

5
0

4

1

811121

24111321

000000
611621

200
311

1

11

0
1

1

315
000

315

00
11

11

000
116

116
000

116

3
0

0
3

3

741

18

0160600000000
100352357499104815941366541203655933395181656

100332306499104015941366541203655933395161656
996478648869515901306431201647932394951653

289781221
100000

11039010
20058121

23

6719111

882011
0000

882011

401

1255701
0000

0200
1255701

2

1251701

4
0

0
2

1

1

2

00000
28557119

28557119
00000

28557119

000000000000
685758127351123772

685758127351123772
316633100010010

59851035421

146114241

7

3

21872111

46227114

000000
72312232

3300000
72312232

213511

187221

22811

6

000
111

111

1

4

1

1

0
1

0
1

0
1

0
1

0
1

1

196844911221079
30000000000

11

6564391122569

00000
91215

91215

000000003000
2841801951572042141209386250246282

121171

2831801951572042121199283250246211
000000000000

00000000000
48247481415107

111
000

111
000

1

11

00000000000
47247481414106

000
221

1

1

12

1

00000000000
1524745111495

1524745111495

0000
3211

3

211

000000000000
2791721931531972081117868240246204

000000000000
2791721931531972081117868240246204

000000000000
2791721931531972071117868239244204

2791721931531972071117868239244204

112
000

112

45624134251342
000000000000

45624134251342
000000000000

45624134251342
000000000000

21
00

00
21

21

45624134251132
000000000000

43624134251132

2
0

2

23

1

1

3

1

0
1

1

0
1

0
1

1

11321
00000

1

11

11111

112113843101
00000000000

1121138435

51

1

1
0

1

2

2

2

1
0

1

0
1

1

1

8

321514215224231287729691971848507200
189218713814013136841390411302100952104887160123325463596714372

457174354121

187848143836339198
000000000000

1

186848143836339198
000000000000

186848143836339198
000000000000

010000100000
186848143836339198

1

184848143736339198

30929230334612111320255
3078734433323565820842637219423242474338338692737

10411743204026222113303868

1
0

0
1

0
1

1

0000
6121

0000
6121

000
211

11

11

2

000
211

211

8

2132

7372262634293557871357154
000000000000

030000000220
621822161016741

2103000
3114121

111

1

1

1

1

676281221
44013000

45

11

1

1

16

2

21

5

1

1

00
21

21

21

00000
11211

11211

1
0

1

000
211

211

111
000

111

1

11

32111

61421
00000

0
2

2

100
111

11

51111
40000

1111

1

6741171631282947681276653
020000001000

221111321
000001000

1

11

1

1111

11

12

01001
13112

1

1

1

111

113
000

113

1

0200321298501
6333161631272845631246449

312113343121

1

1

1

1

32613124466123216

1215131

1411671215162320151611

111

1

1

1

1

1

1

1

1

1

1

128633139177719

1

1

1

3221111
1200000

1
0

0
1

1

211111
000000

211111
100000

111

111

2

11

111200063348644558289107867148
33111500002020

8181471200206131
7018285894282532606573455037

1211
0000

1211

2
0

2

00000000
14542853618224

14542853618224

00
11

11

0
1

1

2
0

2

1121413133
1000001

1

11

1

2

1

22

4413112

051000000000
29173446136195192114

142911

288446136185192014

1
3

2

1
0

1

000
411

11

31

2200011
11321211

3

2

1

1

1

1

21111

16134311915116863

151221233
010000000

1

22133

1

12

1

2

0000
2221

2221

1249771100015001
8777173353321161571012

1527143222

1

611111

5

5290832313126477

5019

1172111

2

2

22

1

57111

15621

31

000
111

111

111
000

111

000
222

222

00
11

11

2

2
0

1

1

000000
714111

714111

1
0

1

01100
251111

25

11

1
0

1

000000
212431

212431

14
0

3

9

2

7000100
2052211141

175211141

231

00
91

9

1

11
00

11

1
0

1

0
1

1

8371
001

837

322

0
1

0
1

1

111
000

000
111

111

440164229762
0000000000

321
000

321

1215131
000000

511

213

10

11

271
000

271

912

21431

1

00000000
41221111

41221111

00000
11111

11111

331
000

331
100

1

1

13

37232121122
10000000000

1111

3232111
2020110

1

11

1

1

11

11

000
231

231

000000
311331

0
1

1

333

00
11

11

2
0

0
2

2

12

0000000
1211223

0000100
1211223

1111

11113

73613165107105944

000
111

000
111

111

1

20448354104101962
020000000000

1
0

1

121
000

121

1
0

1

14231100422910
173361429471342

111

113

1111

4111

11

2

2

111

111

122

1

1

3

21

1

1501001000
3722121151

11

1

1

1

1112151

000
113

000
113

113

111
000

111
000

111

1

1

1

2

34583131121

1752951411343310

1

2

2202142212
000000000

00000000
21112212

00000000
21112212

21112212

191211
00000

1
0

1

000
1811

1811

111
100

11

42154548128
00000000000

42154548128
00000000000

00000000000
42154548128

42154548128

1

00000000000
39414223712

00000000000
39414223712

26413112512
00000000100

1111

1

142121112

11

212

000
112

112

1

21
00

21

1

0
1

1

2052497831290449313991299138312431384245429352069
1316351617873173257125456

000
112

112
000

112

000
6214

6214

000000000000
8260544860344645376229134

8260544860344645376229134
040000100000

11

8155544760344545366229134

11

2933520117161511142218
000000000000

2933520117161511142218
120120401120

1

2

311

122131

111

1

111

1

31

3

111121

3

12121

101126778961816

1

11

111

1

111

11

1

11148

511
000

000
511

311

2

0
85

0
85

85

155279818922382210713014
000000000000

000000000000
155279818922382210713014

11

155279718922382210613014

1687201413892820202113
2100000030011

431111066376869
000000000000

341

1711956366869

1

16

1

11

2

1111
0001

1

1

1

94584626181412143
0100200045420

1

1

1

11

1

2123

1

41

511

314331

71

12

1

1

3

532131656453

1

1

141121

0135000000000
424153311596103

0000000
1546331

11

154533

068000010090
31011335563102

1

5211

512

11

41

311

1

112

1111

112325

11

41

00
564

33
00

33

1
0

1

00
521

521

000000000
441196493

441196493

00000
14112

14112

2441832161020362613247
1470100050040

0790400060120
113182134810251211124

1

11

112

101113

63155106523

13

11

11

11

112

41112

112

3145423

911

31111

121

5

31

1

612

1

511

3
0

3

4111
0000

4111

62113163322
1800000000

812

1

1

54126332

00000
64334

64334

21414741

00000000000
1139244132221

4111
0000

21

1

111

513000000
820241111

1

111

311

322

1

2

1

21

000
1131

1111

2

111
000

111

11
00

11

111
000

1

11

172435383751192313
0110000000000

21411123114
000000000

21371123114

4

6141112321
020000000

2122

3611

1

24112

3591242132144
01000000000

54111

34124212234

1215

00
21

1

2

662333424

37018
0000

37018
37008

1

4
0

4

000
811

000
811

811

1

123211091914
10000000

14111091911
00000000

14111091911

10813
8603

1

22

839938113819182
00000000000

839938113819182
016200001220

1121

2

1

1

2

2

221

1

13

11

11212

1613

1

213

31

2311212

1

1

3241451152

0000
14114

14114
3000

11114

12111
00000

12111
00000

12111

2120135125241346
325770255221261304432308372390401267

111221
000000

1

11

1111

1

448919335442712644363042
259408212190219283397269313340339206

4

11

5

75313453371

51

11

11

1

1111

1

1

1

3262

2

1

432

11

1

11

312

21121

131121

1

1

1

13114

211

12

1

1

2143

1

19211414311411716929721523326025295

1

2

1

1

1212

6512952741225242

3

11131

11

21

214262332638236160

104

1

1

1

111

1

11

21

1

111

31

0
7

1

6

00
11

11

61112

14000001000
538223314323

251

112

511

131111111

3112113

1030113454154
01200000000

732124151

21111233

11

111

2

100012121111
181314719917148152318

1127210873101814

31

1411111

362145441221

21211

11111

1

11

11

1

11

11

1

0
2

1

1

00000
12111

12111

3
0

3

24231211813
23001000300

3211

11

111142

8471671025122261419
2950200341314

21

11

4675215911472

121

1412111

11

11

1

6

42421

1

1

22

1

1

1

000000000
172411115

21

1

161211114

1

2

00
11

11

1315221
1100001

2122

14

000000
311111

1

11

11

1

2

0
1

1

4492272731883782365967412874421106
000000000000

0180000000010
4492272731883782365967412874421106

11912

4471902731873782355967412874391106

11

114
00

114
51

13

2

2

1

425168161681924171916
000000000000

000000000000
425168161681924171916

425168161681924171916

411133
000000

4133
0000

4133

11

2811123
0000000

13

121
000

121

15112
04000

1

1111

1

111
000

0
1

1

00
11

11

150112721007512541515417910469
143210001001

11603000000000
100876186671093245391067052

2

99616156671093245381067052

31

5

00000000000
1475144399114

1

13

174144399114

111
000

11

1

1
0

1

000000
371322

271222

11

4471471111632312
000000000000

1

435137111632312

1

21

21121
00000

21

1

21

387568297357355400448351445778681252
17347106403196

1712
0000

2

111

4

2

1410100101
71011111131

1

1

661

11

1

1

1511
0000

1511

247430229267335237204199299349311191
346744306257214058433448

9998921201849879548712210761

1

4

193162221113

58495113484112210

12

112

1

13

1

1

3

1

121

614141315727386930234

3113211

21

1

1

2

19

1

1

313

111

1

41

223

1

1

1112

22

11

2231225311

2

312

6931351441072

3

1

21

11

1

111

1211

22

2

3

1

11123

315

1

22

112

11223241

1

24132

51131

11111

1

1

118111

11

12

51

111

3

102202661517191919288

11

5321221

2

712

6

211

111

1

131

1

3

4

1

4

11

221

1

1

11

1121

683930503337261815727038

1144556881915723814914242535753
000000000200

1

111

1

163

1

2

1103756881915523714813841734951

4311441

212311
00000

31

21

1

1

1

16

1

1

1

12

2

1

7921112711186822610
000000000000

43473443111
00110010000

223634111

221

211

131112
00000

121112

1

12
00

12

371753713381857
0160000000300

11411

336334321514

62241

4

121

94174

23

1211

4
0

4

0
2

2
0

2

45014111

0000
28111

0000
28111

28111

323538611513
0000000000

00
11

11

511
000

511

11

11
00

11

942741310

71111
00000

71111

1111
0000

1111

292000000100
278121192231404141382832

15311191011323335222011
2664411316529

1

11

2111

5

11

111

21

1

11

1

1

11

1

111

11

1

1

211213173

1

1

11

1

11

1

1

1

1

11

111

2

11

23

31111

31121

1

111

1

12520121

71

1111

14

1

11

5535610232838
55356132421038

3

12

00
41

3

11

000
311

311

0
1

1

1

29211325
00000000

8

11

115

1

11131

000000000000
3183457544227

2334654316

1

1

1

121111

11

2121

121025331312732
0140000000000

000
311

311

00000
102231

102231

22113
00000

22113

2921
100

4

1

42

21

14

3

6821
0000

5411

141

481

2

160001
262132

51

1

21

1

31

1

1

00000
72121

711

22

187601103638714612227414
26944423196327013394144161151340640184

20

013310200200060
416614313416681

4

6

1

161111231

1

111

1211

122214

42

3051

3421898012447396472661179868
7581335461413781316

34

357

3

417071114678131118258

186841373121293540603835

71

5

751

164

1

5451530651311827219

18

1

1

1

3

2133294610227807745628267195460101
1261378246751482020354010427370

1

8

1

14

1

26441

2

34

2511225

1

4

4

1

1

121

1

6

3

1

526121

14221343

49891384211582

1

3163264732172

1

1

3

3

4

1

3

5

3

1

5

230

1

2

2

1

61141

2

4

391121

1

1

4

68223

2

2

11

5

7837

11

191

18573562134

1

3

1

12

3

1

228

1

16

1

2

1

12

3

5

6231918122671211161310

2490224

1

1

2911112341

4711211

6511

2

2

9

5

1242311751246173

1

1

561123111131

1

1

2

13

1

1

3

2931212211

7

12622162121

5

44219212239

23855

1

31

1

1

1

6

1

2

3

1

11

1

3

6

2

9

1

1

51

46522357127152

1

1

4850217444301

668599831111

2

1

1

6

2

2

31215517628429294

16989102

3

4

1

4

778191117

248211912121

2

121

00000
91111

91111

221
201

2

00000000
37111113

00000000
35111113

1

4

11001000
21111113

11

1

1

1

12

2

1

1

1

413222311
10000000

000000
211211

1
0

1

111

1
0

1

11
00

11

1

11
00

11
00

11

000000
1111121

000
121

121

000
721

721

00
21

21

19924015401021152
464191868864853351063375599518340500267

1331000001222
11911177372262309671132452308184157

435318942
15000000

111

4

31

1

1

111

1

1

5

11

121

43

11

21

2

1312

1

000000000
5401212211

2111

11

1

1

229

11

1

811

2

00
118

1

117

3151131
000000

3

21

1

141

11

1
0

1

21

0000000
2141211

1

21

22

1111

89490721218219961942292186766149
000000000000

134353113621

76486718213219958932282156164148

739810544713482
000000000000

11

3213

1

11

11

2

388111

1

2242111244

343121112

1

1

1400
72211

3

61511

00000
182133

12223

11

1

2

3

243113
00000

61

2151

2212

11
00

11

00000
1118212

1118212
01000

131

11422

23

46265811409037613393
3211693214924210094258353283252397105

42

731
000

731

3435515171413201316353218
1410101110331

11

3

1

11

1

22

3

111

11

522234551

112242

11

1

1

1723

1

12

1

23251114122

1

1

11

1

251

1

1

161688431377181911

18

1

1

111

111

126

3

443

2

1

531

3

1

18

26

1

1

3

28141
10001

131

54

2621
0000

5

11

1

1

11

3

31311
0000

31311

617310654435
0300000000

52334425

6

5664

1

11

11

8939132773833262293
471110000000100

3

1522241842

11

2

1321

2511

1111

161

2551

123628222

21

1927311

412

1541

19

121

1043

24

1

54

11

111
000

111

144314
0000

144314

3
0

3

731218654146352639545611221840
23437717407521343437

1

1

12

612

2

21112

1

3

112

3

1

1

1332

883

2611

19311

251

4

1

711

195221

4

1

89884162768512

2

52221

25111112

1506

11

22242

1521

1523

1

2453131

1

1

4

7

181

1

1112

3

2151

1

2

1111312

1

311

1

821

1

212

113321

33

111

143116431152

1

301

1

1319173120

1

1

1012

11

910

1

1941131

1133242

166222214

3

1928131

2

4

1

31612211

11

1

661

2

191

13121

1

4

2

1

1

56115

518811

4

1

11111

1121461

1

1

1

2

242915112

5513312

1865221112

62211

2

7

291

1

12

2

28

124421537992243

1

22131

3222

14

171135

1

22

42112

11

1

1112

3

22

1

2

812

13101

117421

1

6041

31

1

611

8411

1

4

2

1

12

12

265441230

81

1

311

4

119621116

5231113

1372

14

5112

3

1211

1

110000
8102111

1

2211

4111

7

824301006
827312127

1811

22

1011

0
22

22

1

150
2232

11

12

7

1

4917256232439150240173617335
01151400030120

1136

1

11

13112

11

14

31

1

13

1

1323771439832

1

2

11

43344582232729274576734

1

2

1

1

6

11

145112

67223
2000

122

5323

1024
00

1024

5
0

5

1

01300000100
29221312641

100200
101211

11

1

1

00
12

11

1

00000000
146111131

911

171

120113

21122
0000

21122

22

1

1

1102133435216
000000000000

1102133435216
000000000000

000000
161111

161111

11

3112143526

2

1

3

5121
0000

5121
0001

512
000

1

112

3

000000000000
51274802150256784901075769

0220100040210
51274802150256784901075769

0130000000000
6531184231351

1212

45118211

1124

1

11

32111

0
2

2

1013001200
1640213363

11

6

211

1

10

31

2

361162

1

1642465812445465692329
200001000200

15312112

1

4

111

141

341111121

31

111

2

1

121112

1

2

111740157425262572028

2342220093532
131103113317181724332439

1

1771

31

111

61

26311

2121151414

3

102

11

212

341021649217813

12

1

1

14525442342

1

11

3113

1211

2

21131

41

4

11

4

10

51211

11

111

3

1

61

2160010101000
14942511068147116189186222192148126

1

5

2740011001302
3928333264539445776794357

123122611
000000000

11

11312261

0
1

1

111
000

111

1
0

1

000
221

121

1

11
00

11

11
00

11

2

21

11

11121
00000

1

1

121

31
00

1

3

0
1

1

40
44

2

2

111
000

111

00000000000
14393437132

14393437132

000000000000
59283134141393

2

5428313411393

34

21

1
0

1

0
1

1

1

1

5
0

5

00
12

2

1

1111

0
1

1

211112
000000

212

1

11

000
111

111

12
00

12

1
0

1

1123322
0000000

1123322

1
0

1

1
0

1

0
2

2

21
00

21

09300108321872
121341191812263333531933

12

2

1111

3121

22

1111

1

3

11

43

21

51

2113315

411

3

111

2121

115

11

1

141112524

2

112

1157

1

1

516361210311181137

12

1311

1

3

1

211111

1913113
0000000

6

213

11

113

0000000
2136611

12

11

113631

310122332

211211
001010

1

12

2

111
000

111

11
00

1

1

1
0

1

1
0

1

23
00

23

263132118

10812177421017714412914411310569
010100200010

0000000
2121253

11

12

21251

768881081012121612
000000001000

1

2122

645476710871511

2

11

1141211

1

1

1

00000000
8711161212

1

1

1

111

2

5761111

2121
0000

21

111

0000
1112

12

11

00
14

14

000000000000
371210162320121321444012

37119132319111320443912

11

1

1311

113
000

13

1

000021000000
131164112621

111411

2

12

2124

11

1

2266266321555
011023300124

11

1

1

2

11

112

1

12311

111

11

1

121

21

3592165129
000000100

5616419

2211

12

1

11
00

11

2
0

2

911544239178126
10010002031

11

1

1

12

1

3111

1

1

633420812252

1

1

1

1

1

1212

1

11

31

1

00
11

11

1
0

1

00000
11211

1

211

1

00
22

2

2

00
11

1

1

3113112
0000000

1

313112

0
1

1

115
000

115

00
11

11

200000000
543336421

343336411

1

00
21

1

1

1

35301462218826471252710
000000000000

35301462218826471252710

00010
31623

3312

131

0
2

2

221
000

111

11

0
1

1

111
000

111

191

231

524262531329
000000000000

524262531329
000000000000

000000000000
422262431318

422262431318

00
11

11

00
21

21

1
0

1

1122
0000

0000
1122

1122
0012

11

1

1211233
000000

000000
1211233

000000
1211233

1211233
000000

1111233

1

804498768951645210037721286488840912710025112079935
225332941151346346150041950841593

28111
00000

28111
00100

1
0

1

1

0
2

2

00
13

13

1111
0000

1111

11

51204963013051371037318

4211
0000

21

221

1

00
21

2

1

7265724581541181151401558271
000000000000

000000010010
6753674173491061051271487465

55436437694899961231366760
000000000000

113

55426437694897911231326760

13

111

000000
222221

222221

4121
1001

12

3

0000
1111

1

1

1

1

0
1

1

00000
11152

11152

1
0

1

94143411941
00000100400

1

1

941332541

1

1

3453641088575
000000000000

111125311
000001000

1

11124311

100000000020
224244557565

11322

1

112

2111

1

11122124

112

1

1211211

1

17121125211
04000000000

11
00

11

111

1
0

1

10010000
11111111

1

11

11

1

1
0

1

000
113

113

1

11
00

11
00

11

000
111

111
000

111
000

111

000000000
111131332

000000000
111131332

000001010
111131332

1

1

1

1111322

1

342422223923244637842728
000000010000

373174101571325
000000010000

1112312
0000000

11211

1211

1
0

1

1211
0000

1211

000
211

211

00
11

11

00
11

11

411133313
200000100

1

111

11

122212

1

000000000
121116547

115345

1121

11

11

111
000

1

11

000000000000
271318183219142526682022

271318183219142526682022
010000000000

1

1

251

123

11211

21

1

1911141427161199111615

1232782

1

1

2

612443

12

1

111

11

12411
00000

1

0
1

1

11

311

21113321
00000000

0000
1132

11

32

0
1

1

12
00

12

111

0000
1222

1

12
00

12

12
00

12

0000
111333

2

111331
0000

111326

5

0130010425233100
189815919037931209920321233303166301149112677

0
1

1
0

1

7281665982546181891359139
000000000000

6873645976505475881318837
7281665982546181891359139

11

2

1131

1

111

411

1

1

1

1

2

1

22332111

1

1

1

11

12111122

15719114625915884644843431
181314888367311112864309331703028285448032637

611

92214321056
0000000100

922322955

11

11

0
1

1

9102159241301611
0101000001900

98259241111611

1

0000
2141

2141

622550267197415246699714660713811541
159911878247121063825245425272505227243882598

1

1

1

1

1

1

1111

1

1

1

11

1

1211

1

1

1435251171516794

2

1

3132324

41

4123311473

1

1

1111111

242146141117314

78248937035963885421281455

212

111213

11

1

1

1

1

1

117136112

31

2

113111

2111

1

1

1711121

11

11

2

11

1

11

12

11

1

2

1111

11

12

1

1

11

1

1

11312114

1

1

1

2

1

1111

1

121

119179119

1121

31411

2

211

111

614311021619188116

1

1

233142231717144

212

31

21

1

11

111

1

1

1

1

211

1

4916111157

2

1

4

11121

111

1

21

2111

11

111

1

1

173

3251220134193711

1

31211237

1

112

1

1

1

1

1

11

1

11722

1

1111

24311142811

1

23

111

1

1

1

11114

1

1

1

1

21011111

1

1

1

3

1

4332431451

11

111

11

2

1

1

21131

1

1

1

118114

1161110187636294719879452

11

1743645454

1

1222

15

11

11

221321

132

193176227220253254273220192212274137

41

212313

23

1

11

4421221

1113

2231

11

1

1

11121

2

94111515117575

11

1

1

4

1

1

1

22132

1

2481881907719611612

2

127

1

11

1

11

14

11

2

12

12172

1

111121

2

3116141

1

2

11

13

1111

5324237232

1

124

11

12896

6111

1

2222

134113149746616

11

11

1

1

2

11

1

11

2

4121212

52

12

113

11

1

21

1

121

8162217671114

11

112

1111

2

11

11

2

11

1

51

1

33

42

12

5121117151816820171925

2

11

1

1

11

1112

12

2

1

1

121

1

1

211

11

151121

11

1

23

1

11

108197171131146119116124118266168183

1

11

21

1

1

1

12

1

221

111

1

11111

11

31

21252

1

1

758884699988536872

1

12

111

111

1

2

122222811

212111

1

12612

1

1

1

2

11

12

1

1

111

1

1

13131

1

11

11

1131

11

41243

1

11

1

11

11

21

112

111

11111

1

13

2

211

1

1

11

1

21

1

11

2

1

1145152

21

443766423

13

1

1

1

1261471

411

11

11

11113

15

1

1

11

1

1

12

1033952829217

11

2

21

111

11

111

081110010201
12622146461133

1

311

1

11

311

11

110112225932

3897103524354446454518
000010100000

112

21

1

1

211

388793024304044424518

1

11

1

1

1

11

53

2

4212861981181

8515187887
0000000000

0000000000
8515187887

6332

8515127555

205422161610333536411916
010000000300

1136961142014162256
000000000000

00000
14221

14221

1
0

1

1
0

1

8853531010101142
000000001000

1

1123221

8743337107841

0000
12015

1

5

20

1

32
00

3

2

11
00

1

1

144221414313

000
131

11

3

0000001000
1245132522

1

0000
1131

1131

11
00

11

00
12

1

11

113311111
000000000

113311111

1
0

1

221
000

100
221

1

1

1

1

1
0

0
1

1

11411

211271
000000

21271
00000

21271

1
0

1

813654671212898
000000000000

020000
121211

1

1111

1

0000
1211

1

1111

421638352
000011000

12122422

34331

2

412131111
000001000

2

4113111

0
1

1

00
51

1

5

00
11

11

31213
00000

1

1

1

2122

1
0

1

121
000

121

000
111

111

22
00

22

0
1

1

4
0

4

0
1

1
0

1

11
00

1

1

1

1

20853927511710511211597144189158

233344548722
000000000000

1121
0000

1121
0000

1121

133344446622

82575786649994054025030524785210812385
130000000000

11
00

11
00

11

00000000
21114522

00000
11352

11352

0
2

2

000
211

1

111

000000000100
203116123334112924292139

000000000000
5781844855511

54643375456

11

1

11

131

21

15

0000000000
2111322432

11321

1

1112322

000000
111111

1

11

111

000000000000
610581416511138711

1111

125

131

1

154310124545511

1

1111212

11

3

1

4

1

11

11

11

00
14

14

1
0

1

221133
000000

2

2113

21

00000
11111

11111

00000000
21112121

21112121

21511
00000

5

211

1

0
2

2

0000000000
29256211111

1

11

292552111

732233118
000000000

000000000
732233118

11

1

11

1

53213116

1

1

000000
211111

211111
000000

211111

54111

112152561431226
00000000000

112152561431226
0200000001200

110152563325

111

79170783048389849622926220969210542328
131110201221

0
2

2

000
111

111

145231
001000

11

13423

111131
001110

1

1

12

0
3

3

0
1

1

411
200

211

0
1

1

42241232
00001000

1

41313

1

21

1

1

1
0

1

0
1

1

78269782647889349221525120267910452324
132212949032

2211111

121

1

1

112

122

11

1

111

1

1

1564684101566173

1

21

1

11

1

1

1

222111

704154329252798188809159

1311

1

1

1

2

1

11

11

1

1

1

1

11

6

8231422262

1

11524

11

113

679635758424785431104140865779162253

1

21

1

11

0
1

1

1212
0000

1

1

1

21

1000020
2111231

1

11

1

1

1

11

000
122

122
000

122

0000000
2114141

1113

11
00

11

0000
1111

1111

2
0

2

41291071
0000000

41291071

2

00000000000
42643132213

20000110100
42643132213

11

21342113
00000000

1242113

1

2

11
00

11

1111

0
1

1

1

131152112
000000000

111
000

1
0

1

11
01

1

00000000
12151112

3112
0000

311

2

000
111

111

0000
1111

1111

52054313316251841

00000000000
119338453483

01000000000
119338453483

111
000

111

10004
21114

11

11

00000000000
106238343333

106238343333

00
3111

3111
00

3111
00

3111

0000000000
343191021112

343191021112

1
0

0
1

0
1

1

0
1

1
0

1
0

1

0
1

1

1

2

0
1

1

0
1

0
1

1

27367171414234435472527
000000000000

27357101314224133432527
000000000000

00
11

11

2734581214214032392326
3300223301112

121114121

1

1

121124481113971220

1151

21

133

21

1

11

111

12121

3

1

112

21212

6211112551043

111

1

161217612

0000
1121

112

1

2111
0000

11

21

111
000

1

11

1

0000004
1711324

0000
1132

1132

00
17

17

31351361

382320231518372937403178
000000000000

21

00
11

00
11

11

000000000000
382119231418362937403178

1
0

1

731241925343
000000000000

721231825323

1112

0000
1111

111

1

1111
0000

11

11

0000
2751

2751

000000000
41214615632

41214615632

1
0

1

111011111423
26151721715101210272373

1

11

1114

11

111

1

11

3

213111

2212141651069981568

21

12

00
11

11

00
13

1

3

01550000010010
44113775556341487618638539621527447571

115125961117115109

1

1903145322301
4218805406251473609622511597513435560

020011320011
385930233131394232514449

412

311620122321212813292635

1

212121312

1

1

31621356754

1

91336

1

1

1242311231

2

3

11114

111

11

121111

111

1241011

1

11

11

1

11

1

11

11

1

000000
111314

1

122

11

12

0000
8111

8111

1

0420000000000
37912897843364

111647671112

18

311

11121111

1

1

11421124

311

11

21
00

21

3125824295541372504503407490355296459
1910549572246472944782930

1

9

2

1

315755622358537172813825

11

1112

1

221

1

1

1

1

1

111

1211113

11

1

11

2

1

1

2

2553963064291321394397294368192218394

1

12212

1

21

1

1

5

312111

1

31

3372117131

372141474292
010000000000

3

332141474292

645514656451
604660335157624458977740

1

1

2122123

11

111

1221

272118112618402133362720

1

13

111

1

1

11

1

24111

111

1132

25133610213112159533715

110011021002
341221253234

11

1

11

111121

11121

1

111

1

31

9336345351013912
1340000000000

1217331212
0175000000

5111

4

4

15111

3111

1111

11

07000000
327211746

1

2

2

1

2

163

221

1

1112

111

3

1161221111
170000000

11

211111

1

11

3

1

1

1

1122
000

1

1112

1
0

1

000
411

2

1

1

2

113

141
000

141

000
2212

1

2112

000000000000
131676567537727031323617410374

131676567537727031323617410374
224000662931

11243751
11200041

112

1

2251

000000000000
3722223119125

11

11213

1211

1411

1321236612

1
0

1

2332
0000

11

1

223

00000100
11223121

121

2

112

11

2111121
0000000

2111121

171221
050000

1

1

2122

00000
11122

11122

4213732
0000000

1

4212732

0000000
1111223

2

1111221

11

01111001
11211212

1

1

12

1

11

0
1

1

21253
00000

21253

11
00

11

11243566044702452752051488561
150000010120

12

1111

113151

3

18342224

17132725167252625181215

922429352760212244174976840

000
212

000
212

111

11

10761157972944123092749358864012171093808
000000000000

10761157972944123092749358864012171093808
060300000401

1

21618783212132852
9623630136414156692147

1

11

1123721

272

2

523816352230271433

14223

1

1

1

427

1

121

1

11

11311

7

2113111

10551081923906120990642750855311031035741
100297485487411638683554314731010984688

111

1

197712

1

211

2

211212

11

2

1

1

3

271681117137

11

1

21

1

1112828445

1

1111

11

8141571013341112

326

1

11

1

1

1

12122

1

1

3312111

1431

1

1521125921

2111

111

17

1

1111

111221112

2131

1

122

1

211

12111

112

111111211

1

11

262136751134

12

1

111

12111422311

1

53544101413582234

11

1

1

31111

12

1121

1

211

1

1

1325

1

13

223112253311918

8242244161617155
128135815253930403719

1

11

3381

1

11

1

23112

2

21

1

1

1

1

4163610131812131711

11

0
1

1

00
11

11

2

1

000000000000
1844207811182618191711

1

132917751014211012128
000000000000

132917751014211012128
050000010020

1

41

112

2

11

1

2122211

23252312112

11121

115721

22

1

1

3165762744

2

211111

1

2

101

1

11

06000000000
514331458753

11
00

1

1

1001
2111

1

1

1

4312233442

11212431
00000000

123

1

1111

1

21

2

878722223435242019386076
140427253390183232741521151114981628137412341891

822162522041283249611247277447125106121284
102040000000

000000
113212

1112

21

2

00000
11111

11111

0
1

1

00
11

11

643167354712548899737834
751155621611022240010776456406164025351218

1641

11

63115032048979231710305595184742403761157

21

13

1

11

11

11

11

111

121111

47142762018282427787316

961617101287510

1

11

6

1

0105030000
841157876116

111

13421

65352495

1511

3

4637261386332415142735033
58644025975437295871016555

41164112

11

1

111

51522323213

2

1

13162

361112214831011

9

2111

1

1121582

21123528931

1

91276943

11211

1111

112253

1242

1

2

000
313

313

64122968814754807791637169
2126570021010

11
00

1

1

010120002000
599141018920101512

12343366

1

211

131

31

214111

31412314132

1

233

11

141242

1

14

1

11131

21
00

21

00100
11111

11

1

1

293552767522
000000000000

293552767522

581131550483
307144427916503529123237

1

121

1

111

51223163216

1

1

21212

1

11314

1111

1321

11

1124

1

1821112923622

1

1

41

361318715121233

11222

1

11

151238156236721218

241734224634152534351917
000000000000

241734224634152534351917

000
112

112
000

1

12

00000000
11111134

0
1

1

1111134
0100102

111

122

201323348278303048472829
217704902288285148375366524349258239

3

1

000
111

111

0
1

1

1

21422111552
00000000000

21422111552

00000
45112

452

11

143111122014121917232814
2365584301395

1

22111114

111

1

1

1

11

11111

4226133111

1

22112431

1

1

49111124367

1

1

2

121122361

21222

1

1211

11

1

11

0
3

3

1
0

1

32511213
0000000

32511213

1
0

1

11

1
0

1

0
1

1

783144210115
00000000000

783144210115

2

0013
1113

11

131343471212586
001000000100

1

1

11

13

1

1111111

1

13121451110273

1

1

11111
00000

11111

1

1

11
00

11

1

43642399851

21

000001111200
33491214171011190168434016

3149111417910688167403716

214113

0000000000
12412411212

111

2

1221241

21

00
11

11

010100001100
77261516245431369

1

1

1

414312962

36251415201227

1

64922315421167316
3321220380816005

11

1

2222

2454422531

1346

93

211

00
11

11

1122

1

1

51124402649288575112705937
200310201100

2211

37115322030216963102645323

21

1

2

12583187121082614

2412311
0000000

4111

3

211

11221
00000

11221

00
11

11

113522
001210

112312

1
0

1

11144111
00000000

11311

1411

15
00

15

221807051004716555775683070
572515620577129231

16234121

222111

13111311

1

1

13121113

943219137447

111

1

128464232146292845491511

222

112321334235

13121131

1111
0000

111

1

00
11

11

1

1

41

1
0

1

3322152
0010000

1111

1

3

1

1

13

111

1
0

1

1
0

1

6230706261526
183829142611333931352623

61616814715191381510

1

12

1

27321998951

1

3

1

1

2

2113

1

11

1122124113

221

1

1

12

1

211

13244114117
00000000001

12142114116

1

11

1

1

00010000
21121122

2

111122

1

2

122

31121112
00001000

312112

1

00001100
11122213

1

1

1112

1111

121441
000000

100000
121441

2

43

11

1

040000210000
7255442583331221401162627943

4311794541
1000121000

1

132123

123121

122

11

1

11

1

00000010000
169454486252226353

12

111111

131321

1

104144424230

234441113

178

21231

121

22832

2

111235621
000000000

1

2

1

113311

251

112244212
000000000

112244212

8425135101315685
211576288312425131817

1

44582154744

1

1

75151613426

13

22

1331

1

1

302329164819273527151922
1785294131145912

1

21

13

311

1342

11

3211315512

4102010301171013759

1

5123223111

1

2

1

000000000000
735152626558755756789571

1
0

1

2535438141710223423
000000000010

16251277148152713

913117327610

762061741317
484847586250614046556148

111341

414145585549533341516041

6877696316368107891047054109
001020001400

001000000000
4869575614751755375444287

281915161822624360413810

211

18504140128291310153475

1

1201000000
3222452722

11

1351

11

1311211

11

1

0
1

1

1
0

1

11
00

1

1

111341001312
1681051214283026151020

1112

1

1

1

1

1

14692771818188616

1

1

1

111

11

11577521

712
000

712
000

712
000

712

1
0

0
1

1

020001201010
498349314324497273272366302419439430

00000000000
3163218174842

00000000000
3163218174842

1

1

363218163732

111

225214265132125201921
000000000000

000000000000
225214265132125201921

225214265122125191921

1

1

0000000000
23255138221

0000000000
23255138221

121

324511721

22

2121134121
000001000

212123121
10001020

7211

5

1

11

1

11
00

11

000200000000
15811011812613411758685513414795

2131
1100

31

1

1111
0000

11

11

11

00
11

11

000000000000
535147621231

1

535137621231

13
00

13

0000
1111

1111

21
00

21

0
1

1

711254321292
00000000000

42

2

1

111

611412129

202222012032
141991061181219744584810813290

1

111

1

1

11

1211

2314141

12592941121159040484110212678

74533525227

1

2

111

1

331115
000000

15

23111

42311311
00000000

411111

12121

0
1

1

1
0

1

00100
21231

1

131

2

000000000000
434237394431131816261567

00000
11212

11212

111
000

111

00000000000
14253432843

1

1

11133421522

1

1111

3112

00000010000
81223475522

1

121

11

3

2111211

1

21

1

2

1

111

1

111

1311

333732333923671111762
23533851308413

93127283016262346

1

11221234512

000001001002
23414810914125886141192159195226216

23314810814125585141192158195225214
361575912613814178

1

11

126113233

1

222

1

11939515161771814

11

1

111

1

1

111

111

11

121

132111213

1611028410722859117142117145171165

1

1111

2

11

1111

1

1111

11

3

11

1

1

231

1

8311321

1124

111

5123115611

111

1

1

1

111

113

1

13332

1

11

4114323

111

21

1

1

1131
0000

1131

342622113223183632342428
000000000000

775140473851
342622113122173631342328

1

14

1

1

1

1

211261123

212

628314341437

11121

11

1

11

2212121

3423734129138

1

111

1

112

1

1

21

553851535

1

111

222

1

31

311323513

1

1

0000
1111

1111

1
0

1

000000000200
1038110514631009864200810768861362931996572

102101123510
1038110514631009864200810768861362929996572

001000000000
501023201213775144262721

11112

50921191113765044262719

11

010000001000
36065310726643391532509375791313422168

31

4

11

1

289627050602448735274428738092

4

695899996112781506172336234076

21113

1

1

3

1

11

1

112267
000000

1215

113

122

11
00

11

1

11
00

11

231
010

1

2

11

111113
000000

1113

11

625437364324511461486449515584546379
253631343326143545322618

1

55

1

1

12323214

2141

1

1

312

111111

111111

124111

655123163233495917

2132111

111

1

1

11211

7111116112

1

11

14298841081071711811181671189178

11

1

11

2151534235

11

1

5311

34661410161816223

4344121713251151

111

12

1

11

112

25

721

1

1

1

2814216113172038253913

1

12

11

111

1131

1

11

1

111

1

111

21

1

1

12111

11

112

145

1717211889304314322641

11

21

1310812171221131272214

1

11

1

4223112

1

1114

1

1121

31

11

11111113

11

14131

11121

342812132424466252828

111

1

47914216136

111

11436621

1

1

1

142316311823813

11

1122

31

11

11

2136

1

938988781047562647411612368

11

1

6112

346851557142018268

1

903019134260688041937526

11121

23111

1

1

1

11

913853961712155223924763287
745531531507134115351147893339298814751518269203601438

0022620
32581018962

2254710892

1

11125

201

1511109823
000000000

1511109823

1

000
113

113

00000
12212

00000
12212

12212
00000

12212

001001010003
1921255913649067382326

00000000
1219382274

00000000
1219382274

1219382274

22111122627
10000000000

000
114

114

1
0

1

000000
112112

112112

0000000
1112411

1

1

1

111

1

2

11

0000000000
57312191717105

21
00

21

00000
22112

11

11

112

12425

0001130
1311331

11121

2

000010000
12111681234

11571224

21

1

11

000000002000
111120468353226151216

1
0

1

0000000000
1122122112

11112

11

1

11

121

1242728933
0000000600

324

1

1

11

12112

223

2

11

1

111

0000
7121

7121

1113
0000

1

113

0
1

1

88132551823135613
000000000000

88132551823135613

1

1

229198140156174170205222268300285181
020001220900

11

000000000100
1912422557132114

5131
4000

3

111

1211226
0200002

11

121

113

1
0

1

000
111

1

11

01100100200
1772254371314

2

1

11111

11

113

15412412464

1

1

11

414222345453
00000022100

11
00

1

1

0000
2211

2211

0
2

2

2000010
21311232

121122

111

00
11

11

12112
10000

11

112

112111
000000

11
00

11

1211
0000

1211

1251008712600
205170133151172162193208247265278174

21114
00000

21114

116
000

6

11

0000
3111

3111

175134113106127133156170199210235148
534731624261212520536741

2214

111

1

1

12111

1

2

1056758326565878210212514269

1

261662338553

52442

1

1214

1

111

148145122254134121726

555

1

1351671

3741

1

1

1

1

1

1

0000000000
1242115212

111

113112

2

12

1111

221
000

221

122273224241110191119
000000000000

4

121873224241110191119

001000
111131

1121

11

000100
311441

311

11231

1

00000
22561

22551

1

1

000000000500
9468113981631293

9468113981626293

00
11

11

622

4126212

1

1

1

000000000000
139511359269523

128511244249523

1114

112

1

0001
1111

111

70222630123010031133771871339094614456016951195781095
2711376982973865085777711

415185432713911
146229158151150100226233305517171155

32
00

32

000000000001
217114913302223231421

207104913302223231416

114

121142312
000011001

1

1111111

2

2

1

1

11121
00000

11121

2111131355025651
0200001000

293035492544

1

111

1

2

0000
1222

1222

1
0

1

020011021020
3171463104355611224

12121

2

1

1

1

2

1

1

1

1121

1

1211

52112332

1

15103438515551

1

0000
1113

1113

00000000030
231173513348

11

2

11

221

2843116

21

1111

1111

21321217232

000000000000
1210131134711728217

1210131134711728217

148233124
000000000

2

128233124

1
3

2

03011
23423

2

4

1

2

17812645842626392214733
3210202201100

1

11

1221

1

12

1

221212

1

141161

122

1

1

41232111

83524377822203016429

1121

1

1111

1211

000000000000
37551696731385498

37551696731385498

111

1

15

1

000
331

331

0000000000
612171215733

36

69111215733

1311112
0000000

1

1211112

010000000000
1152538192029111110

11

1311821

41

412

111

1111

4112411520596

1121112

111

111

1

1

1

101021495271238281812
210100232203

1

1

33

111

111121

654458101895

1

1

12

1

1

12131

2211

1

324

412

4

111431122

1

111

1131

0000
1212

1111

1

1

5131215102589121422
3878321778216

2

11

23

241121126

121117

1113113

1

1

3231316391591611913
2910122207028

1

1

22

111

1

1

12111

11

1

1

1

14645463

2

11

64124221

111

12

3221

2118

0
1

1

14232
00000

11

1

14112

12566

28113310977936295113102460260123
377102324130

13321
00000

13321

0020000000
1851234535

8111

1

21222

11111144

00000000
72111111

72111111

2619684688751819383271248122
323121100420

111313142

131145226

4312312

12222122122

242342413359

5361213

121828271215342928792512

2366441236324344346162196106

112322

00
31

21

1

4321412132
0000000000

4321412132

323222272
010000000

21112

1321172

13316215
12004100

1

1311115

00000000
41411331

21411

21321

1
0

1

212170
0000

212170

242121926419101
00000000000

2142
0000

2142

1
0

1

1231
0000

121

111

00000268010
23212142538571

212121421

12

2

212172632

22415

000000000
311242143

311242143

010
111

11

2111
1000

1

11

1

1000000000
22095242210312

1209424112

1

21103

0000
4141

4141

6292160554642157229386104897921430861400618668410
469307331614168515520795141747267012

000
161

5

111

1

3304262211617363
11130100120000

1812

1

6354

92411413

3521

51

1

1911

11

22

51

46

31

433300043162
2230241465514065212515

111

1

1111

2233

1

1921263

1

21

45211913191

1173614773857

1

12

12

71143731

122114

2

1

151267141

1111

1

1

11

1
0

1

231

5581

1

1

5
0

5

215122837

0000000
62183437205

62183437205

21873512006131511050
123834161211260648204349419

1112221

1

2451

352

1111

134718117

1

1

2

1

2231

63244225372

791812228121632195

154844

1351542

134443

1

32105102

1

12415011273597121515125419513883
2905374333515112379097611111647513338

3

11

1

11

22411

8

2

1

3131

14

49162641244948

1

12

1

1111

1

63

614216829651617

2

1263

16211485541

1121

1

413

13311

1

1

1

24

1381

173651471728274345603715

1

1

11

211

311

1

132

1

1

1

11

1

1121

3815

1

7221

21

122

1

1122

1

8118213578

2

221

221322233910111

31279932

1411213

1

2

21

1141

2

21412113

1113

1225

113

1

1

3

38412

1

311142

1

1

11

22

11

1

26431182527861

2112132133

13

1

621375927346439814320862101153

11

4221

76141

3141

3431

1

31

1

111

1

12321

4

1

21

41

1

1

131141413411

13222

4210919018973861161021201859478

1

11

29

4

11

151

1

11

42111

41031303855

11

114

6

4

21

111

1111

3312

010000031000
910427418242024422184

544272254271

3

21

1

33176406236863

121

1

1

121

11

1

2

3

1

11212

2

00044641
12184108192

217913

11511

1

111

12041420
445204372

2443

141

120122242

62120240124222737961
108349101312799911184273015

4113311

1

15231

21915

4

111

251131

12743

152

61415354

1

642

1113241

11

11

111

1151

4211

2

121

911

251

11

154423

2251133211

31

12

1

123

182141

1232

232241

1

111

422311

17111744121

1

11

4312121

51

2

1227312

3311611211

121

2213101063

1221321153

0240202
4364415

1123113

311

8104188214157191612
13001051330550

2

122111719413

1123212

1

111

1

2

132

11

1212

11

1

1

12

1

11

11

1

211332

11

1

311

111

21

1123

1

3317942391
00000000

1

3316832381

111

141

890138315102157167267
5353175845824691315681045415322

310132711

5259174447809411293781028615052

2122

4342

000000000
11164241912

1

1121182

2232

22

111152

1121

1

3185
0000

3185

117138531911122951
118268187182106139284268343351202116

1342313461652
0800000700

2117

1132121

251035231

72362243127329132
040000212020

111

11

33241

1

1212

13215913222

1

6752

113112

111521

1

111

211115213

2

111

00000
13211

13211

1

322
100

222

1111212184
0000000000

111211184

11

110001
612123

212

52

1823212
0001010

1

812

122

31
00

31

11111

111312
000000

111212

1

236546412
000000100

131444311

15121

11426
0000

1

726

16

262220162117142027141731
010100212001

1

32225734

121

1

11

111

2515191219154171561329

11

14

1

1

1

2781210141171010123
000000000000

41

238121014117910123

01010
12111

1111

0000
2121

2121

11
00

11

213333261931394754432823
72124136136599318018324224314568

313

111211

1

1

1

17191093360609769315

2354699224556265631076436

11

11

61115623451071

1

11112

131

1

1

311

6222471

1

1211112

21

2

2416

1

1

1

122145943

2543201023527815
14925220715619816490112151486183261

1

000000
111121

111121

3301110272231481618
43311122823717101719

11

1

11

11111

11

211111

11121
00000

11121

1

21
00

21

14
00

1

4

000000000000
69891241241088592719113122176

69891241231088592719113122176

1

31221
00000

111

2

1

12

000000001501
2333341718151342

1133911

2

1111

1272321

1

111

1111222

114711

1

1

1131

000000
122222

11

111

11211

1255891151627111012
0001220012021

13135228735

1

131

1

1

11

21

141

1

21

2

111

11

81241561544

111

0000
1223

12

23

10210100000
668427512132

11

11121

11

21

13522431011

1

2111

2331221422
0100000000

1

1131

1121211

1112

11112171
0000000

11112171

11

27595064136263455341944
81400120071012

21231

11022

1

21

54136213833

1

11

12

211

111111

1

2114428521

11

11

61746434201720314636

1111

111

1131

31

1

1

2121

1

1

00000
14213

14213

00000
121112

1

211

111

11

1

1

111611322
00000000

111611322

100000000200
6718089165192179977387323104105

1

00
11

11
10

1

121105101233
0000001170

0000
5332

5222

11

00
11

11

000
213

213

11
00

1

1

212162
000000

21

211

2131

1
0

1

100000641000
1623162912342313251102728

32533

2
0

2

010000000003
1421162812328412742428

61111

141415281232517692323

1

1

12232

121

00000020
11263733

1143

12231

11

112

070010031100
4814569132179142624840857277

135
010

1

23

1

1

1
0

1

31333445111
10001201010

21

13122

121

1321

2111
2010

1

1

000000000000
4310263128169142584131807076

11211

1141

1111

11

1

4310062128168141553727796776

23232
0000

23222

1

000000
731126

00100
73126

3

111

45

1

2

0
1

1

1111
0000

000
111

111

1
0

1

0000000100
1211214621

00010
11111

11

1

1

11241
00000

11241

21
00

21

000
111

11

1

000000
111112

111112
000000

111112

11122
00000

11122
00000

11122
01120

12

71627766954924

1

21

1322113

00000000
42253261

00000000
42253261

00000000
42253261

42253261
20111120

12324

1

111

1

1

1

000000000000
25341911271244294127915

224331283413

2332158241142213823812
000000000000

030000000000
21301481893421312085

1121231
0000000

111122

111
000

1

11

1121

000000000000
21251281683020311775

1
0

1

21251281683020301775

221628737

3

4

7

1

13

1

5

1

0000000
12511123

12511123

2

319630221990164922341798305130213335368632682192
4161512279743

000000000000
270492237199234191395338395369329218

270492237199234191395338395369329218
090200100010

00000000000
86344974716

0000000000
3414471315

234421315

1115

000000
327131

327131

00000
22221

22221

00000000
36116411

15541
00000

15541

000000
211111

1

1

21

11

121

122421
000000

122421
000000

122421

000000000000
70538646826910378989010048

1

21211
00000

21211

2

555083458067957389779446
000000100010

341

121

11

554982427867907189769346

0000000
10135514

10135514

2111421122

1111

000000000000
251511841267492649324622

000000000000
24321213712314

24321213712314

744451256745

00000000000
472241158596

472241158596

016013000100140
1213693316317132017327

61

2

61

3711

312121

7

232

3422

2111742222

3312111

1112

12431

1

111161

2214211

2121216523

341144

11

51124353

213

1111

6688461816149125
000000000000

000000000000
6688461614148115

1

1432

641236121011795

275113

0000
2211

2211

677640464033997388825340
020000000000

00000000
22123141

21

2122141

0000000000
41441521011

211

111

2341529

00010
11111

1111

4822
0000

1

3622

2

504633342629846163714635

717351037911564
040000000000

3321311435

11

1114212

121

21

112685

11111

1

61

1112
0000

0000
1112

112

1

00000000
21122142

00
11

11

000
111

111

1000000
2112121

111

11112

000000000000
1877392622241410

000000000000
1876382612241410

11113

177637151224147

000
111

111

0000000000
1121214217

1121214217
0000003000

1121211

214

1

2

000000
212776

212776
000000

212776

000000000
111412121

0000
1111

1111

1111
0000

1111

000
311

311

0
1

1

5313843324135638093615659
000000003000

04710121113002
2111512121717365345212612

1

111

1

421

32

11

345112

121

4311

1

2522132

22111

1

2

11

11

121

1

11

11122

21

21

1

1

1

12

143648726461911193

1

2

13

121

000000000000
322029202117272644392946

11

11

291528172017262340392842

1112143

1212

1

11

00
11

1

1

1

000
111

111

000000
212111

1111

22

000000000000
123116142425363733262315

00000000000
11314151817613

1214131011

11286613

51553651510615101

000
112

112

0000
1111

1111

00001000001
51478121957419

11

1

111

1111

1127611123637

11711

0000000
1121151

1121151

01000000
11521131

132111

212

000000000
124112332

000000000
124112332

2

1

141322

111

0000000
37233146

37233146
0000000

27233145

11

00
11

11
00

11

000000000000
111372663319248105

000000000000
111372663319248105

710030615041
111172663319227105

000
211

21

1

2141121910111
00000000000

111111

1412081011

273126697543

21
00

00
21

1

11

2
0

2
0

2

5222222423

389349176186174176204233242254332238
000000000000

389349176186174176204233242254332238
000000000000

389349176186174176204233242254332238
4400010000020

13

31111
00000

31111

31262342
0000000

31262342

0000
10111

3

31

411

4772817871639596134739564
050000000000

4765817871639596134739463

211

0000
4121

4121

0900000
11012211

11

12211

000
421

421

1190000000
1301312145

11

71311

13

31122

13

41013141131
0000000000

51

1111

3513331

152
010

22

12

3

375274466433
331110001002

44164454221

11

111

3261
0000

3261

106436261021930149
000000000000

106436261021930149

12211
00000

12211

27112

000
121

121

132321494123
000000000000

11137411

1213111213

2884554746459606151116189125
31310677948177776876134208145

42536713344

1267723339494

131311

15

111

321

611

312

3

6131

1

117

15

111

11

688758124246

1124115

3711

125975467656176648911281189115414441267730
000000000000

1

000000000000
194212610119362517

194212610119362517

210000131260
124075067456076348311181178114514081242713

000000000000
2059911379965010916116226123698

2059911379965010916116226123698
1510565739123

1

135

2

111

2049310374904410215514825423495

1

000000000
242313322

00
21

21

242313121
000000000

24231221

11

000000000000
20516171483092581513

20516171483092581513
000000000000

20516171483092581513

306122106451117517722519535329681
340400020121

11

0000
1111

111

1

112217
010215

11

1

1

1

283978740966512617016633927773
18192110201192621121711

151

12121

1212

2333521

1

4323114411

111

341614102623301419683314

18551421331523521016812

1

11132489314

31

1111

121

2

121

221

112

111

21112

22

3

1

1111

27837141525

11

211

3412

1

122

1576117172930112314

51716659135

1

11

11

211

61326121

3227

322111354

121

11

53112

112

1

1121

1

111

126

111

1311

3532

423

13

3113

12111

1

1

1

11111

00
11

11

12
00

12

23111
00000

23111

21348611
00000000

11

2146511

211

21121
00100

1

11

111

19924252140
712158627339592

133421341

1

2

1

11

523322123511

23121454121
00000100000

11311

1

112

1

11

11

11111

111

000
131

31

1

00
12

12

421022111
00000000

11111

32922

4754423411492
000000000000

4754423411492

111
000

11
00

11

0
1

1

7
0

7

1112112

21

1112

000
112

112

698508432413535348796774736773676513
1914773469215131

1
0

1

0000000
1122113

113

1122

11214141222

1
0

1

11111
00000

1111

1

00
11

11

240111125000
311591064113015302912

1113221

3

11

11

4

111

21111314212263

211411

21

1

122

14122101

4578111612

9152742179358
000040000010

21112

1

1

1313

6131242136125

1

1

11

11
00

11

1
0

1

1
0

1

2141221278512294256214257200194147
22273021357483960342425

1311

111

1

1

11653

11111

111

1111

234951

11

311

11

11

1

11351

121313

111111

1

22

2

922144241171212

162322

11

114

11

2411123

3211

11

133

131

31

1

1

8623115410546

1132

131521211

1

132

218131934175

1

11

11111

312112

11

21321

12

1

1215

11

1

211

2698357973585

1113121

1112

1131

1

1

11

1

111

31215112

115

111

11111

11

12132273

3111

11

22110613

1

31

1

112

1

11111

1

2

111

1411

21

111

1

22

2321169724131851617

112121

1

1111

21

8832372934391209490978055

41

114

6161226416

21
00

21

2
0

2

000000000
511521442

511521442

3111111
0000000

1111

11

1

11

000000000
273214411

273214411

011
111

1

1411
0000

1411

2121111
0000001

21211

1

612938139564
00000000000

612838129554

111

1

41211
00000

41211

1921116136111214351410
831130333001

1

141

12431

3111

1

1

11

121

1111

23412111

1

22112

414311

111

11

1

1111

131

12191

1

12113

2111

2121133124

111121

010102004300
89695159944012179110789055

3211434

1

313

91146554379

584837528625995975586131

11

11

1111

171284341411236136

21111

13112213

11

1

111

0000
1213

2

1211

11111221
00011001

1

1

122

0
3

3

91033241449417
000012100002

211111

1121

111

1

4931442

111322

11

000100000000
273758222511292212

224232152

1

11

1

1

1

11121215

2323123823215

1
0

1

684111251081191219
201210001323

1

483811598106816

12

1122
0000

1

1

2

2

00000000
21211212

11111

22111

115211421
000000000

115211421

000000000000
612027253650835738814030

612027253650835738814030

00
11

11

12232
00000

12232

000
121

121

131
000

131

214135
000000

214135

00
11

11

295527665220266931462936
030000000000

1121

112122

22

54782461168210

7

244520574914194913382325

21
00

21

0
1

1

1

00
33

33

110000001000
152926262327131713141832

11111

1124

142624262127121611141432

00000000000
23223316323

213522

1

13221113

21117111
01007111

2

11

211124
000000

21

21113

641616496161373
000000000000

11

1

641615395161373

2191291510193218181510
000000000000

11121

12

923212179864

51162121

68127117597934

142118101913121930242919
000010002000

11

1

11

411

1117149141371212172013

11121

24412515165

121

51111111
00000000

51111111

1114
0000

1114

00000000000
1322319861067

132239861067

1

261714121910776050263522
000000000000

214427610194

2

2316148158675439252516

1

1

112

1
0

1

711111
000000

711111

00
11

11

000000010000
223120203121471226483725

212819192820461026473523

11111

1

111

112

111

1

169126133172717302518
712151681411118

11

41

1

1111

7795821116214139

1

11

00000000000
361144233362

11

111

1

36934232351

4111334633

00
11

11

0
1

1

1111
0000

1111

12

122
000

122

1123121
0000000

2

111

121

2

0000
1112

111

11

178652421

1
0

1

0
2

2

1

11
00

11
00

11

000001010000
6496745173535484169508781174849682530

000000000000
349249219161267199672563781431385218

138142107821597349840357120818973
000000000000

136142106821597122914623220418572
000000000000

136142106821597122914623220418572

00000
22413

22413

0000
2682523382

2682523382

11211

1
0

1

4212144523523
000000000000

1000012
1122122

2

111

11

000000000000
3211122321323

221111137111

11114212

1

20710511177107122170155187218194142
001001100011

5312515533
0000000000

5312515533

121
000

121

252018141214402946323319
000000000000

11312

241917131213372845293319

11111

11
00

11

17781916190106124119140185156119

292418290190274216277307390409292300
3321336772062

51113113
10001000

3111113
0000100

21

1

112

11

111
000

111

122

366348531067
00000000000

0001000011
3462273647

1

3361273636

311
000

311

000
111

1

11

00021
12231

22

1

1

171623332311821719522233
01310000000020

0
1

1

000
111

111

12
00

1

2

8

000000000000
16283331291811715522032

16283331291811715522032

11131

00000000000
344341438138

344341438127
00000000000

21

342341437127

11
00

1

1

000000000000
18121010135131913251429

00
11

11

11111

31

000000000
8341105976

834195655

1

21

11

1

00000000000
17651043134181423

211

17651023124171423

111
000

111
000

111

1

00000000000
588151486735

00
41

41

544141285635
00000000000

34112125511

11

2

11

11

144

1

11

11

11
00

11

00
11

11

4

1021541538212190137163210193156103
000040002324

445652293842405056937138
000100120000

445649283742354855927138

1

4

111

2

3257442035276975104464639
58979952774696112152958160

12

121

111

111

2211214512

111111

11611

11

12111

11231

15112

391591937121465

1

1

211113

111

141934171710161115302014

1112

1

1

1

21

2

1111212

1

212211221

1113118131914113271210
000000000000

1011118121614103171110
001000000103

111211

4324113312

311564761

4111

2111

12334157318233

11

111

0000000
1213111

1111

213

1083543395545465661525487
500000000100

4212139192210121452022
000010000010

4212139182210121451922

0000000100
21153821613

1

141221211

11

12632

512030143314313342292453
011211000206

212113928

2

1112

111

2374

11

1111

1261041761451397

1

1

31

2

11112131

1

1722225251917669

4472212117

12412

13111

122

31

11111

1

811

0000000
31375118

31375118

000
112

112

11119
01000

1119

00
21

21

0
1

1

00000200
32124561

00000
22331

22321

1

3116

11

121

1

1618151119339222319518

0000
1313

1313

878271739412

1

174

609704376341503516334350329750642465
000000000000

000000000000
609704376341503516334350329750642465

4

11

00000000000
178121311151032

178121311151032
00000000000

178121311151032

1211111

23

609681367324501511332339324739638462
39301772422553642623331

137824294029151716291120
071000000010

411

123942721

4313321411

1

1

6

13

11

1

1

11

11113

11

121321

1113

2

11

212

21422313

111

4121211

1

12

1111

711

14014172521182110

32212

00000
12121

1121

11

11
00

11

12
00

12

0440013220030
1151434635501854584411597109

5

131111131

1

3

11

212

1

11

3

13

94403220376112412946795

1510566626202219175

2

4123

1

6

331

1121

111

1

211

111

1

1

732297413

1

22

1

3213

1

11

1

1

1211
0000

121

1

0
1

1

3213276624316857416920616163
441426279253387441206228218531495300

11511

215

41

1

32131

112

36

12

162113

3

311

3123

12111

1511

111

2121122

121

1

338164164161302248141174125251289188

32111

7

11

13111

1321

12

544335273917368653247

000000000000
533141135214

533141135214
000000000000

000000000000
523141135214

0
2

2
0

2

22314322
00000000

22314322
00000000

22314322

0000
1131

0000
1131

1131

2
0

2
0

2

0
3

0
3

3

1

0
1

1
0

0
1

1
0

1

3199212
00000

00000
258112

219112

39
0

39
0

39

11411

000000
115421

1153

121

1

1

2
0

0
2

2

1

2311
0000

2311

00000
11211

1
0

0
1

0
1

1
0

1

11111

01030000010
650319154964011

3112

1011111
000000

000000
711111

711111
000000

711111
000000

711111

0
3

3
0

0
3

3

1121

000
122

000
122

122
000

122
000

122

4510143211

2313142389
00000000

00000000
2313142389

29

0000000
231314236

000001
17314133

33115

141327

6113

5

00000000000
61353221341

31332111141
00000000000

31332111141

0
1

0
1

0
1

1
0

1

221112

1

1

1

010000100000
1866141598151920502010

112

2011

000000
223112

000000
223112

000000
223112

000000
223112

11

21

221

1

122081035714151567
010000000000

1

133121233

000000000000
11138723514151034

0000000000
22115912114

0100021001
22115912114

54912

11

1

111321

1

91176235292
3100000000

000
214

14

2

1
0

1

231
000

2

31

12
00

12

00000000
11111141

11111141

461133231
000000010

11

2

11

111

261311

00
33

0
3

3

3
0

3
0

3

111

0
1

1

421342362331123
130000000000

2

00000000000
31533342225122

1

111121

11

0200000000
2133323125102

15
02

1

2

1

121
000

121

000
111

111

1217

1

0
1

1

000000
111311

111311

221
000

221

000
311

311

0000000
1122161

00
11

11
00

1

1

124
000

124

11
00

11

111
000

11
00

11

1
0

1

000
311

0
2

2
0

2
0

2

010
111

11

12111
00000

12111
00000

12111
00000

0
1

1
0

1

2111
0000

0000
2111

2111

2

1

3
0

3

31

341482387290318286139313071724603414358
01052210001210

1

91912

11

341367384288317286139313071713600411357
0506502011120

1
0

0
1

0
1

1

23272228197138231440014070115
000000000000

23272228197138231440014070115
000000000000

23272228197138231440014070115
000000000100

1

1111

23252127197038131240013970114

211

81116625139202062
000100100020

1

00
22

22

0
1

0
1

1

0000000000
2311341153

121141
000000

11

1214

1112152
0000000

1112152

1
0

1

000000000000
6101141288171412

12121161
00000000

11

5

1

12111

1

2
0

2

00
12

12

100
154

2

52

0
1

1

000000000000
5581117251311

5581117251311

000
111

111

15810821117714986171012
000000000000

000000000000
14788219587165151011

100000000000
14788219587165151011

111

1278821958706515911

112219782121
000000000

112219782121
001100000

111119782121

111211
000000

111211
000000

111211
000000

111211

8235373811
0000000000

821537381
000000000

0
3

3

821237381
000000000

821237381

21
00

00
21

21

000000000000
133444212121

0000000000
1334111111

0000000000
1334111111

113411

2111

1

00000000
12131111

00000000
12131111

12131111

00000
144211

00000
44211

3
0

3

00000
41211

41211

0
10

10
0

10

000000000000
131566539764

000000000000
121564532753

121564532753
000000000000

111

121554432653

0000
1211

1211
0000

2

111

7

8673764979572001652411619773
020000005100

00
11

0
1

1

1

000000000
236112635

236112635
000000000

1111

2262535

11
00

11
00

11

4135382245285530641085144
012001303111

3930342244265129571024641
110051001617

1111

1111

12

1032014171222111812717

421526141

11

111

1

1013377553470206

22

11

12121

11

67182171026378

211111

11

111

12321

112

12

1

0
2

2

00
11

1

1

1111
0000

111

1

2
0

2

00000
21131

11131

1

000000
111121

111

1111

11
00

11

000000000000
22101077121129214321127

22101077121129214321127
000000000000

1111111

22

83111110883130235

111141

132211

10254543612281

215

000000000000
186189196223319252018

185178196203319241918
186189196223319252018

1

1111

11

11211211
0000000

12
0

12

1

00000
11121

11121

2114122

000420000002
344436223254

000000
121322

1122

113

0000
1111

1111

1
0

1

001002
112122

1

11

12

131111
000011

131

1

0
1

1

135121
000000

0000
1252

1252

0
1

1

11
00

11

1271
0000

1271
0000

1271

000000000000
523548154249115

000000000000
3204359413552

1
0

1

000
111

111

0000000
13212121

2211

4

21

3111

21

0
1

1

37131711222
00000000000

37131711222

111112
000000

111112

00000000000
23113611463

00000000
13111111

13111111

00000000
11126352

11126352

0000000000
3113524823136

0
1

0
1

1

0000000000
2113524823136

10100216111
2113524823136

3

1112111

11111653014

1

000000000000
482621113125445737433933

2211103101320171631410
000000000000

000000
112211

112211

00000000000
996352761051

996352761051

132341111114388
00000000000

132341111114388

000000000000
432244431121

321222230111
432244431121

11

1

111221

201275147173419191820
000000003000

12010111
22111121

1

11

1

00000
21212

21212

181033126132814151515
000022000000

32111161

411

1

11

11

1531292112114141514

211341132
000000000

1313

2112112

22112311851
0000000000

212
000

1

22

11831
0000

11831

0000
1111

1111

00
22

22

00000
12121

12121
00000

12121

0
1

0
1

0
1

1

11
00

11
00

00
11

11

000000000000
13717422616211984655591809169164103

000000000
313211234

001000010
313211234

1

312211214

12179491683213915
000000000000

3212
0000

1

121

11

1

021000000001
12149471683113913

31271211337611

11

211

3

54331531

131

1

2111
0000

111
000

111

11
00

11

000000000000
1201552141561106764558680415015588

0100002212000
1201552141561106764458680415015587

1

1

1

11

324141612106

7428353659371481141837310138

391223235020332613463746

4

1

1110154891844542858012112

1

11

1

1

11

1

1

1

111221

1

11
00

11

1
0

0
1

0
1

0
1

0
1

1

2

9121

211

4

2

1

1111141122

11

1

2

1

1

611219201024214811
000000000000

11

611219201013214811
000000000000

611219201013214811
000000000000

61019919813214811

0000
1212

1212

1

000
111

000
111

111
000

111
000

1
0

1

11
00

11

4

25

1

3

00
11

11

00000
11211

11211

000000
1055911146

1055911146

4859

1

0
1

1

321

21

2

1

030000000000
260258237191247192243282255319302294

000000000000
119170154154150135143174124153136165

001000000000
119154153151150134143174123152134165

113146149146146128127166116145124162
000000000000

113146149146146128127166116145124162

312
000

312

1111313
0000000

1111313

2

002001300020
6224451556483

624441256463

000
121

121
000

121

1611111
000000

1611111
000000

1611111

0000000
2153212

2153212
0000000

2153212
0000000

2153212

0
2

2
0

2
0

2

1388276359355100107125162165128
000000000000

621730814533244910410554

111011831023
766546277950678376586074

11111

4111111
0000000

4111111
0000100

11

11

21

11

11

00
11

00
11

11

1

1

272715193927181621331933
010000000200

00
11

11

411234731
000000010

222451

1

12

2

12

272115183825151214271932
030000002101

1

11

111

121

11

1

261513173725131112251930

33141241

45292763821376150223336
000000000100

2131111
0000000

2131111

0000
2323

2323

000000000000
39663712223413173024

39663712223413173024

42319228614273738
04000100000

41719227513273627

1111

21

122115211

2

0
1

1

1

19133711

111

1

2

1

1

1
0

1

1

2

1

1

3

2597554544306559427677110088731052761719
000000001000

1

000000000000
2597551544306559427677110088631032761719

000000000001
2592547538302554423669109688030982750713

000000000000
16109102217153019211816

000000000000
1555102014152819201816

11

1545101914142619201815

121

1542321

543245164332666662544346
10111011919100

2081981712241313202221
001000100100

136225

1651671389105121421

1121

1

211

1

32

1135

11

1145

001000000000
239135144151711181116

64211893

1

1

111111

1111213

1651039247171110

132122311
000000000

1

1211

2

1112

111

56566255933

000000000000
265248111911656

21

21227147111

11

54226453344

2121
0000

1

2111

118187124123159190704437210132126
000000000000

118187124123159190704437210132126
000000000110

1

118186124123157190704335209130126

11

1

1

11

863237265124586695976943
000000000000

13454352381982
782630203621445275865438

11

1211

143

111

2811125111261224292422

31

23

1

12

1

32313313

3249820231334028189

1

1

1

11
00

11

55331012105111

31461233110644
000000000000

1

31361233110644

2128205210471487927665647623602242327
000000000000

010020296120
2128205210471487927665647623602242327

2

2119199208451447727364446723562240325

2

411112

1

1

2

2

11

311111

11

11

000000000000
189811138013080183229189355245153

3

189781138013080183229189355245153
528302115921103

2

32

11

143111

112

3477816222056162128

1111514

4

1

75125523179139

21113

2

1

362313

11

1111

1

86335450854349545021612874

3

15

31161

44202121658315285736017

1

12

12111

1

218911510415

11

1112112

2

1

1

11

1

1

111152111
000000000

111152111
000000000

1111

111

1

5

1

5464548465116
000000000000

545243426476
000000000000

101010015040
544241416476

44323141436

000
121

11

11

00000000
12114214

11213
00000

11

1113

00000
12131

111

212

2

00
11

11
00

00
11

11
00

11

1

89110441995355863701349586
000000000000

15

020000000000
8995441995355863701349586

7822181355154655481087633
000000000000

7822181355154655481087633
000000000000

6111
0000

1

1

511

7813181252154652461027426
000000000000

7813181252154652461027426

3322427
0000000

1131

13

1

1211

2113

1
0

1

2813
0000

0000
2813

613
000

613

22

962266402012821261950
000000000000

962266402012821261950
000000000000

190010002312
962266402012821261950

1131113

1617431152334

1

113

1

21111

133

4113

16621

1113

2

23121

1

133

1

15

1111

251

11

1

1121466

161

1113

241111

1

1

1

1

1

2

000000000
111311112

111311112

15061358486753901061078410668
050000000000

10354216151519443429394527
000000000000

112111555

835251091116222717102111
040000000000

13
00

00
13

13

411

1240000000010
825171091116212614102111

1612777121818115168
000000000000

121

12

1625413

1264176181484143

2113211

3945012111
43200000

1

1537911

20331

5111

1911

0
1

1

1

000000
311122

311122

31
00

21

1

2

822
000

1

2

8

1

0
10

10

0000000
1211211

1211211

000000
111113

113

11

1

24141
00000

24141

000000000000
191754422167241916

1311

3
0

0
3

3

151121757231616

3422112

11321
0100

1311
0100

111

11

91

11

341338214330264251222926
000000000000

2

341138214330264148222726
000000000000

100000000000
341138214330264148222724

841411188681365
00000000000

841310167671344

1121121

2471872520203335202019
000000000000

1

1

1

2461872519193335202019

163221
000000

163221

2

2
0

2
0

0
2

2

13
00

00
13

13
00

13

1311
0000

3
0

0
3

3
0

3

111

000000000
514432451

312312

22122431
00000000

1

21112411

000
111

000
111

11

1

1292843132219172613

136113421

00000
21122

21122
21002

2

1

11

3

00000000000
41844551111412

0000000000
314354111312

0000003000
313354101311

11
00

11

3335371311
000000000

3335371311

111
000

000
111

111

3

1141

00
11

11

1

61

1

3

3632726728396974509636131041248858185

00
11

1
0

0
1

1
0

1
0

1

1
0

1

1111
0000

0000
1111

1111
0000

1111
0000

0000
1111

1111

2

000000
145141

1511

0
1

0
1

1
0

1
0

1

313
000

000
313

313
000

000
313

313

00
11

00
11

11

000000000
321112231

1

00000000
22112131

22112131
00000000

22112131
00000000

11

000
111

111

0000000
1112111

1112111

11

1

1

0000000000
433331310545

433331310545
0000000000

000
122

000
122

122
000

122

0000000000
43233138543

33233126442

1121
0000

1121

1

1

1

1

4

00000
13521

13521

1

753325157146141231213
000000000000

000000000000
753325157146141231213

10742128
020000

711

111

000000
2432117

011000
2432117

1

2

1266

1

1

11

3

11

6125251171442431213
500000000000

4000000
6331314

21
00

1

11

2111314

1
0

1

0000000000
119241021411410

0000000000
119241021411410

5314711311410

6610311

1221
000

1221
000

1221

1100000000
231111221243

00000
22212

12212

1

51

420000
1271113

712

15111

111
000

111

32

4
0

0
4

4

41

1

1

2

7

2

1

1

5891757262116127650695575923558369931068297647154
6594131615174740618510530

0000
1111

1111

457163735248542055068711457042415350914183785992
10421116821549443

21258422

10451337127310471374893768908814111611701917
1100011001000

1

000010000001
339247240160331148205281248336350359

677770468249196051939073
4611566422438

11

1

21

1

111142342

1

61696842763985026838361

11213122

1

27117016811324899186221196243260284
431013953373520595320

116638652152465571599384111

1

1

1

1426825610561712

31

1

11

118

2141125

1

1

111

1

1

3327555313

1211141419517121612175

21

11

1133

112322

2

11

7

1241612

216

1211111

13

31

1152

1013641211171023141828

1

2

2812

14

1

1

2223231

121225117

11

1

94113436

1

3

1

4

212332

1610931272423409812

113

1111

6

1

3113752

3010992610461162011

12321

1

12423

23112

131415

327311224441

211

112

21
00

21

11

1

01000000000
33111148141027

111

1
0

1

00
11

11

111
000

111

211

21611310913
0030008000

1

12113

1231

25

12

2
0

1

1

0000
1342

1342

1
0

1

1611126437122512117
010000000000

1211

00
51

51

4341211721
0000000010

4341111711

1

00000000
31421433

31421433

11121
00000

1

111

1

1

1

2131468731
0000001010

2

1

213143641

12

3

000
211

1

111

000
111

111

3313115
0000000

315

3311

5826226934876084003553963406306951229
163411000000

0000000000
221412158264

31221

111

11231113

13

111111

516

70141872324162130138214126
000000000000

21

11

13112

21141

1111

11

1321557655369487

121

5651221716957461163118

1111
0000

11

1

1

31
00

31

001100000000
122111331421

21

1

1

1

121111141

1

0000000
3227475

11112

111

116373

4044725704085002937197725035521092
000000001000

1

1520671218714119610

1

3894525644014882746383604945461082

000000
111111

111

111

100000000000
702847283648547247525956

11145414131

1

1

1321

1

1

1

132

672732233047476643515555

12
00

2

1

00
11

11

000
111

111

11112
00000

11112

21123121
00000000

21123121

0001000
1151111

1

1

111

131

9411616811113621191821
110000031510

2

22

23122114

1

1111

11

3251114535

1213125272

11

2113

1

21

8122132111

1101111

11

14122

342123

3

1522

1

111

11

111

2

1211233213

1

000001000100
598353667235

1

277352667135

11

12

40000000
91211212

11

1

21

1

321

2

213654
000000

21654

3

0
1

1

0000
3647

3647

112000000000
111912122292112151855

11

31

8169111691810141045

1111

1

11

1

57

1

3000000
6311111

1

1

11

3

211

00000
12111

21

1

1

1

222148114
010010000

1

1

122

112

11

1

5

1

111

000100000001
104443317393429341197211186128112315

716912492193119843421
644143083623973091851971608471286

1

1

11

1

2121231235642

111424

3812224410

111

111531

616347146214310

11

2121

1

12145714

1

111

383722843413352746657524237237

1111

11

11111

11

11111

1122131

1111

1121

331992828279916393923
000000000000

331992828279916393923

00000000
11211111

11111

111

1

00001000000
69224349415

11

111252

41111

211

11

1

12

1

1

1

1

51

1122

1

00
11

11

1

2354111719133261341
351449903962435741247816378233214523802172014070

237335363053368930247117276020823162644560833103
31243134251331052

6

29262912186211719403820
010000000500

1112

724571723101

2220227106181015302817

2111

1

1111

303937343631476236424159
000000000000

1

293835293624385430413956

111424

11

124521121

12112

3

020000040130
4171452214124181411

1

1

411135221473151011

1

1

111

1

2

1120111013
2121224123

1111

113

147731600712599828112971387597386
000000000000

111001376019371117713

111

11

314132132223

51141771023474355123215

32211215

111111

12

1

11

11121

111

211121

21

11123

11

736124376395644296341724950349

86688455146376366691087495
82427225461091715749

22124

1

673751236524334833705136

1215123

1013151961919107

12922112

0
1

1

1212
0000

1212

00
32

32

6413214736311
00000000000

1

1

1111

1

2411363511

311111

926989621169842302610780139
42641304255109

1

886782581039838282110269130

1

111601710101
8767331251225

211

21

141121323

44111

31

1

15181048848374431197
119545536221725153

1116521

11

1

121312422

1112

441143

12

254161

1

121132112
000000000

1212212

111

0
1

1

000
421

421

00
11

11

321113351
000000000

11131

112

12112

11

000000000000
43315534104212

4131441274112

2112231

9177141445187251618311860
100000010011

576127381963129111922

1111

1

11

1

2961312923125527537

2321111

31111

0000000
1431112

1431112

42112252825511
01000105101

1

1124281921

11

21

11

1

1

0
1

1

000000000100
546822694365

1

1

12

436822691265

00000000
31211211

1

11111

2211

11224
00000

113

1211

000
211

211

116112
000000

1611

111

30814510158231118918
045122011010

1

21

1

1

292928138221018618

1

311211
000000

311211

000000000
331433531

2

321133431

111

1018241523131181231116
000000100000

12433

1017241421134483816

11

112

11

0001000010
2413162124

11112

211412

1

12

11

1

17045738828830646816715819983111101
223907441327376503236229294149165157

3

1312

121

21

2

16

1

1

923114225

1

2

1

5

1121

1

1

1

21112142

2

102811012646168

11

11

1

11

1

1

11

1

11

1

12211

5

2

11

143115

1

3113

1

11

1

30161313111

411

3

1

116954131112

1

1

1

6120125353715710

121

3111451318

11

1

41922

23212531

1441294311224

11

2111

1

1

1

1

1

113

1

11

41311

1

211121

2

11831233212

1

12111111

3111211322

121

4433152536631

1

1

1

1

1

121

1112

2

1

11111

162

2211

21

2811

6

11

1

14112131

1

2

262353201029222

1

1

151

11

1

123

17
06

1

1

15731224149923551493542615969981926572554212022
000000000000

13653511119314124

15601218149423521488541515859891923571154092018

0
2

2

11

0000
1112

11

111

000
323

323

1

000000000
181192181209421

11192181209121

3

8

772158917168710
010000000100

533771415669

1

132122211

111

1

421142211137182256
2412123187225164197102526

857724102346

1

2

11113

1111

1

21213

1

361

1111

211212122

1

1211

121332

2

1

612322145

12

211

1

1236331
0000000

1236331

111
000

111

0
1

1

211
000

11

2

0101101
1722813

11

1

12111

2

161

1

1111
0000

1111

1
0

1

381719103714263828201829
000000000000

371719103714263828201729

11

0
1

1

00000
11112

11112

21
00

21

11
00

11

101000
114121

12121

1

0000000
1121117

114

211

1

12

000
121

121

131121
000000

0000
1111

1111

311
000

11

3

040000000000
636232213750475095594421

000
111

111

458433644752
307423534642

211

311

111

310
311

1

000000
132512

132512

3
0

3

0
1

1

10211112

000000000000
1453132915143025203

1453132915143025203

2211
0000

1

1211

11010001010
452152211142

1221211111

2111

2111

11

43421259654
10211001111

12

1

1

11

111

1131

1

31

111111

121

1

11

11

000002101010
234391011122324431585

322323512

28211

311212

111

222

106515811110632

21

1

111

111

1

1

62422156234

12

0120000000000
479600419245431330577699800586431350

0170000000000
916039225549120137132945938

1

11111

64323316363997109104834727

2665618923282881211

42

13633214878143143216259287205168150
5610976565580575971657764

1

321111

2131513

42341531553

2

1

11411

311113211

1

21

2

108252

119485

1

1315212

1

111

111

21223

1

21

1

1

1

112

1

1121

311

1

1

11

1

1

2

11

1141

1

33918567101791

1

11

211

1

413742361

1111

1

9511258211226689

311181139543

111081

111

1

111

21132

3

2631116113

11

11113

1

1141

11

211

111

1

1111

23111027

22

24615141096212

35

11

1

1

1

421221

11

11

1

21

1

1111

1

1522111212

11

11

2

1

21

63

13

39

2121

11111

123

11

1

2125

1121

1

2112

1

31

1

12

4

11

611

1211

3142552

1

3121

11

1

1

1

11111613

1

211314112

1

1212

111

21

11

2

21717411054

111

1

151

2

1

1

51

121

12

8721621826364138

11

521

1

21

2313

1111

612134369435

21
00

21

245186227141229131239281361273193160
554283101658159

1013436254038343325785430

611211143

1

1

51224432222

53134223423

1

1

1

12214218010817480188226320174117114

11322

1111

1111

0
1

1

101000002520
7754472221813102

114

66344721816882

1

11
00

11

0
1

1
0

1

77358485108105
000000000000

100100010200
77358485108105

1112

1

4624312214

1

12123

11

111121

1141124

111

1111

2

1
0

1
0

1

000000000000
331529124526312013253235

001001000000
331529124526312013253235

1

331528124525312013243235

397
00

397
20

197

000000000
232311122

11
00

1

1

1
0

1

11
00

11

1111

11
00

11

0000
1212

1212

15810513712918012978957618817992
550400399361550273351460435861586442

000000
113362

3352

111

0000
1111

11

11

6211152982
000000000

6111152882

11

291015576525201616
000300000000

11

11

1257265347512

11

16581113114

0100000000
111127102033

11127102033

1154526593420152720136
355281246225362136256349323618383328

19258663611121

1

2

3

1

1

223

274117112313413442743711

34421101582268

227821065010145118177156354239127

121

1

11

112131

1

2112

62125211185

42122174091114612815

2111124

111

354364232525

43111214

111121

51587491931161232

111

1

1

1

11

1

1

2

121

1

633933

1511

1151181012551

2016111316161217624611

44193373148158

1

111

9776661299101420

1

11

22

1

7691428112434239

1

11

1

211212

111

233232211122
000000000000

1121
0020

111

001
111

11

00
11

11

1
0

1

11321122
00000000

12

11

1111

1

1

1

1

1

3513212117711
1100000001

1

2

21

8

1

2211121655

1113

1

11

000000000000
1757411151012225912

000000000000
1757411151012225912

212101021111
1757411151012225912

1

1235241198194510

1

2631131

11

1

11

123621

2111
0000

2111

101
00

00
101

101
00

00
101

101

000000000000
22281515196272451362850

000000000000
19281415146252350312848

00000000
42112131

42112131
00000000

42112131

11211314121212243261746
000000000000

1141161
0000000

1141161

00000210020
2751153142914

211

1

21223

1

43111710

1

1121

0
4

4

0000010
36211510

2215

2115

1

223

33241232
00000000

341131

3211

00
31

31

00000000000
5467116172411319

5467116172411319

21
00

11
00

11

1
0

1

1111
0000

1

111
000

111

2

112132
000000

000000
112132

112132

00000000
53121161

4211
0000

4211

1112115
0000000

111

111114

11
00

11

2152152
0000000

133

111

2211

11

6674706072682047461297640
96391876154878265711251097134412111064865

250000602001
502291353253345328589528726638630373

3
0

3

1231491465
0000000000

1231491465

1

1

145554984103

000
111

111
000

11

1

000000000000
4993514321134

00000000
21111111

1

111

2111

24112122
00000000

1111

11

1

111112

1

471214203
471214213

1

22
00

22
00

22

152114511

12

1
0

1

1
0

0
1

1

235157180122171135295310395270258188
000000000000

000000000000
218101017692311536

11

218101017652310436

31

12596111618490162177240187175131
35204292331313453303826

115248159139913

111

121

142121

111121

1

11111

2

221

1311

41

11331

11

121

122

211113

1

1121

22132311

1

211113

1

11

21313311

61791879545

4131121

31

111131

3765421111174

121

1

2321115

1

21112121

21113122

2113131

11221

1131111

11111

441231427123

11112

11

13

3

271725161643788495898262

112

1111

1

111

1211283

1113

211

3141151

22

365553111023415

1

0
1

1

0000000
1221312

11

11

12311

1

1

1151222

0
1

1

000000000000
823143242751

823143242751

132111

62526272531314223513

000000000000
92162521383110685116646535

92162521383110685116646535

2212111
0000000

2111
0000

2111

11
00

1

1

11

1
0

1

0000000
2221113

1

0000000
2121113

2121113

000000010100
1465062477510719510422126727475

1

23612111
00000000

11

11

1161111

1

1
0

1

13

312122211
101020000

11

1112

11

1

1

0
1

1

0
1

1

2312032411
100000010

1

11

1211

201

1

212

0000
2111

2111

1
0

1

12112

000000000000
4771319151530173318716927

162

4771319151430172718716825

1

1

000000000000
18446144897131818

1

18446144896131818

11
00

11

21

11
00

11

00000
11121

11

1111

00000000
12513212

12513212

2112
0000

2112

11
00

1

1

000000
111111

111111

4311585151

0
1

1

62152514305614369158527226

1
0

1

000000
111121

111121

16

1
0

1

00
12

12

121

1

11

1
0

1

361332273531122020322625
000000000000

0000000
1211232

1211232

11136165
00000000

1116165

12

1

1
0

1

361230253430121713281717
000001000000

121

2

21

351228253427111713281516

000
111

000
111

111

2
0

2

323242656142
000000000000

0
1

1

121253211
000000000

121253211

1

0000
1111

1111

000000
121321

121321

000
111

111

111
000

1

11

121
000

000
111

111

0
1

1

0
1

0
1

1

2
1

1
0

1

00000000000
71131671762

71131671762

00000000000
1718432112117

1718432111117
00000000000

3

1418432111117

0
1

1

11132265132
00000000000

0000000
1111341

111331

11

112113132
000000000

112113132

000000000000
40919143014343435242923

40919143014343435242923
000000000000

40919143014343435242923

000000000
395112121

2641111
2530110

111

1

1311111
0000000

11

121111

365122288151044

95635112646814
000000000000

000000
144112

144112

8563511225712
00000000000

8563511225712

1111111
0000000

11

11111

00
12

12

3

1

000000000000
76737456924691768711659123

76737456924691758711659121
000000000000

1111
0000

1111

76737355924691748711659120
000000000000

76737355924691748711659120

00
12

12
00

12

377771418133123135243218
000000000000

000000000000
377771418133123135243218

367771418133123133233218

121
000

1

1

2

000000000000
13111611175145861029211511095180

00000000000
91325342369

1111
0000

1

111

12141227

81131132
00000000

8121122

11

1

0
2

2

1
0

1

765864376123736792746160
100100000000

000000000000
2319266177161320341538

2218266176131018301337

111332421

51241211223
00000000000

51241211223

131711121041217241171
1630251618817213215126

31314484538335

112

1331
0000

1331

11121181233
01000020000

213

11

112114111

2341
0000

1

234

11422192434

000000000000
2321041638111115209

1819415349814188

1

5111422121

0001110010
3111113241

2112131

1

1

1

000000000000
1423913149898251212

0000
3121

3121

1420913149777251212
0170000000000

123910139576231012

2122

3111

923562443174
00000000000

00000000000
923562443174

923562443174

000
121

121
000

121

0
1

1
0

1

00
11

11
00

11

1211
0000

000
121

121

1
0

1

23313116584912910594
00000000000

23313116584912910594
00000000000

23313116584912910594

18112811

00
21

0
1

1
0

1

00
11

00
11

11

0
1

1

000000000000
120105113829887165192203164127102

000000000000
431744312034656469313124

431744312034656469313124
000000000000

431744312034656469313124

000000100000
5335682017221996

1

12
00

12

211112

000000000000
2224671817201976

111

1224661817201876

403441458653
6575624565457194991058171

421

1
0

1

000000
112122

112122

25342112322

1
0

1

13413976561
00110120110

111

1

122

111

11122

216451

1

1

16465751310554
000000000000

14234631362

11221

111

2111

111441

111

1111161
0000000

1111161

131423442
000001000

1111

11

12131331

1

1

000
111

111

111

00
11

11

1
0

1

21411
00000

1

11

41

1

21
00

21

21111262865
00000000000

212214

111

1114625

61111851311341216
000000000000

61111851311341216

31112646
00000000

211445

11221

821132312

1
0

1

1111
0000

1111

122

211959216581125913

123212131
000000000

112

11

12

1111

12

000000000000
10457911218111059

10447911017101057

11

111

11

211
000

11

2

1111
0000

1111

1
0

1

0
1

1

000
251

251

000
111

1

11

0000000000
3111111511

3111111511

1221243156
000000000

1

2111136

1

22221

1

11

2615331
0000000

123

1432

1111

1

1
0

1

11

111111
000000

111111

1
0

1

0000
1122

1122

111
000

111

000000000
3211351012

1

111

11111

4812

1111

1
0

1

000000
125211

1242

111

000
214

210
214

4

7841651713961
00000000000

7841651713961
07000000000

7131551713961

11

8582113
0000000

858212
000000

848112
100000

1

47112

5

1

1

00
11

11

11
00

11
00

11

151816668713710196

1
0

1

1

511
000

511

67252723106811
010000000000

511141
000000

000
111

11

1

00
11

11

44
00

44

0
1

1

21
00

21
00

21

6125271262611
5114170110410

1111511
0000000

1111511

00
11

11

00
12

12

000000000000
820494786121014169

0
16

0
16

16

000000000000
818894786121014169

000000
123124

123124

5
0

5

169111
0000

169111

21
00

21

11
00

11

41264445810793
000000131000

1

1

4106444459793

301101421
311111651

1

1

122

0000
1311

1311
0000

1311
0000

0000
1311

1311

1808410086111979111296150142109
000000000000

1808410086111979111296150142109
131170862605

000000
111132

111132
001022

11

11

010000001300
223233302522193436363126

0
2

2

255511122
000000000

235511122

2

12145
00000

12145

11

0
1

1

0
1

1

1

1

2111

32
00

32

72363635661

917221781782516231315

000
111

111

1

22
00

12

1

1112
0000

1112

134741333114
000000000000

1

111111

23531334

1

1

2

156496654797563725710810876
1243946525968474025908851

1741252881461010
00000000000

174952771261010

3112

1211112

00000000010
13571228975712

12571228975612

1

235311
000000

235311

111112541

0
1

1

1

1

11

1233
0000

0000
1233

0000
1233

0000
1233

33

12

714467265954515065503389
000000000000

000000000000
8139815412141213411

000000000000
7987112910109210

000
111

111

1313
1013

3

100011100
121228423

1

1

1112

2161

11

1

1

34211112215

314271422
000000000

314271422

000000000000
141142342421

1113421
0000000

1221

11

1

1

1

11

121
000

121

10000
12112

2112

111

00
11

11

000000000000
602756164350383452332776

602756164350383452332776
000000000000

000000000000
411641143045251835141760

411641143045251835141760

1

79101642521356
1510152124131517191016

132

6565814535

1

1

1131115

4111
0000

211

2

1

0000000
4111221

4111221
0000000

1
0

1

000000
311121

1111

111

11

11

3111141

3

2251148221
030000000

2

6

1115

11

1

1
0

1

211111

1

2

00
11

00
11

00
11

11

1

1
0

0
1

1
0

0
1

1

2

1

4510384138254140435778475187
001000000000

000000000000
4510383138254140435778475187

4510383138254140435778475187
000000000000

211
000

1

1

1
0

1

0
1

1

439883136252136415675455086
000000000000

21
00

21

439683136252136415575455086
000000000000

2

439683136252136415575435086

13112131
00000000

211

0
1

1

000000
111113

111112

1

00
11

00
11

11

00
11

00
11

11

1111
0000

0000
1111

1111

1

1117121
0000000

0000000
1117121

1117121
0000000

0000000
1117121

0000000
1117121

21

1115111

21541146327
0000000000

0000000000
11441144327

11441144327
0000000000

11441143327
0100000000

133221
00000

11

131

221

11
00

1

1

0021001
1121316

1

11

1

34

1
0

1

0
1

1
0

1

112

00000000
24242211

1

24232211

2

1

2

201312

11121

00000000
72314265

72314265

1

11

1

400000
511141

14

1
0

1
0

1
0

1

111

000000000
3171112123

3171112123
000000000

3171112123
060000000

10111

2211
0000

11
00

11

21

0
1

1

0
1

1
0

1

0000
1112

0000
1112

1112

1

41

1

2

1

1

1

1

1

12111

1

1

12222231
00000000

1

11222231

1

1

512

11

1

70654531565412411012218312455
020001004101

1215223123

70624330515112210711718012154

1

1

3

214416896814181042
000000000000

214416896814181042
000000000000

0000000
112011111

0000000
112011111

0000000
112011111

112011111

1696443105411
00000011000

61214241
00000000

00011101
61213241

1

5211

1113

0
1

1

1086422521
1000000000

4542210
5542212

2

1

3141
1110

231

122

1
0

0
1

1
0

1

176232241351
00000000000

176232241351
00000000000

3612111640
3612121741

11

1

0
1

1

11132
00000

11132

312

1
0

1

11
00

11

2

421121
000000

000000
421121

000000
421121

421121
000000

000000
421121

212

2211

95324381666
00000000000

95324381666

1

1

000000010000
20316127721252625203

8

11111
00000

11111
00000

1
0

0
1

1

1111
0000

1111
0001

11

1

1923574518212624172
000000000000

000000000
431196645

000000000
431196645

431196645
000000000

431196645

152354349152020122
000200000000

710513234375
00000000000

00100100
12211314

1211214

1

6831121261
1000000100

51

21

211

1

31

3111

3

11

1111

151

8121151091162

11132

1142123121

1

766354294620547080695475
000000000000

2

746354294620547080695475
000000000000

746354294620547080695475
000000000000

121792
000000

000000
121792

121792

53714343148179618
000000000000

33513343128127414
0301210010010

1111

63

2111

21

32515

233

1

11145235

11

1121115

1111
0000

1111

1
0

1

1112
0000

1112

000000
113212

113212

692639264217386156514657
000000000000

66124113443
0000000000

66124113443

111231443641
00000000000

7112163

411324211

188191418682325131216
522531263814304650413853

43212331114

111

1976495710105516

12133111

12

1

21321

3113312

41

1

1115

222215229142

111

32321215212

3

02380000003000
2371058217143182187225253239250257299

0000000000
3614411425

13123134
00000000

12133
00000

00000
12133

12133

1121
0000

1121
0000

1121

21

231121
000000

000000
231121

000000
231121

231121

21

2
0

2

27215333

00000000000
62232964633

00000000000
62232964633

62232964633
00000110000

116211

000
111

111

51231233522

253345242532
0000000000

000
12481

12481
000

000
12481

12481

0000000000
128535242532

0000000
285313133

285313133
282000100

231312

121

0000000
1511122

0000000
1511122

111

141112

202225881315162522298
000000000000

202225881315162522298
000000000000

000000000000
202225881315162522298

000000000000
202225881315162522298

202225881315162522298

6567594148649111085456464
000000000000

252

1

474843342343658564364427
000000000000

000000000000
474441312040617254304023

1
0

1

11
00

1

1

00
31

31

00
11

11

473641312039607153304023

1

2

4233341310644

000000000000
1819157232126202172037

1819157232126202172037

1

1111111112

2121
0000

2121
0000

0000
2121

11

22
00

2

2

1411391258611210591111112155151222
070000210100

3411214531

1401211218411010081106107145148221

010000000
171136621

621

16113411
00000000

1633
0300

133

3

00001
11111

111

1

11
00

11
00

00
11

11

0000
8121

000
611

611

00
22

22
00

22
00

22

1

2

1

2

33

3

1

11

41131
00000

41131

1

15910012713713613512710711119014289
010000000200

4627851181251

15593125130128130116999918314288

3

1111

000000000000
167161117694411752562391804574463

71354121815514106
000000000000

000000000000
71354121815514106

00000
12111

01000
12111

11

111

61144121814513106
000000000000

040000000000
573412141351275

1

1112

522411131351075

14141131
00000000

14141121

1

139127105584241672142041531642440
000000000000

010000000000
139127105584241672142041531642440

139126104584241672132041531642440
320000000100

00000000
38142311

2211311

1641

9

133107103584241632112041501541439
230001000000

1

1211

12996102584221612112011491540438

1

111

51211

11

000000000000
212177164232022152117

0
12

8

4
0

4

21977164232022152117
000000000010

000000000000
21947164232022152017

0
2

2

020000000000
1694714423202251217

2

1674714423202051217

0000
32108

32108

3
0

3

211

9

1

000000000000
29923117681157137305341371368291192

512619161445356821584326

2

010000000000
2482031576514392270273350310248166

1215106862529211149
000000000000

000000000000
1215106862529211149

000
111

111

1503006103000
1214105862529211049

111

112211

1

242117691

1211

11

2113

12

11

1

16

111

81122578413

11

11

1

111

135578433845416713119111012990

000000000000
681185220361752721001799953

137416610271116191411
000000000000

13
00

13

210010201746
4741154173418109

1111

1

111

1111

65

1

2

7111

121

11

11

112

2

211

1

11

1

1

1

21

85161812112

11111
00000

11111
00000

11111

851121165111036
000000000010

7144211911

1462123114
0000000000

116121114

3111

1

000
111

111

1

18137448111621101210
000000001000

11111

00000
11111

11111

111
000

111

1
0

1

1
0

1

212
000

212

128617814179108
00000000000

12841781417998

21

2211121
0000000

2112

211

00
21

1

11

28261882062740521386926
000000000000

011100110000
27251371452639481316123

19161161131431351205319

1

1

11

1

1

3145643

112

23132542

17251

2

115161114783
015150102552

1

111111

1

11

2

11
00

1

0
1

1
0

1

000000000000
32121161515264138101514

32121161515264138101514
000000000000

040000020000
32121161515264138101514

2

327115141323313381112

11138513

1

1

11

1

542586488307491375404565517545567515
121010110210

000000000000
87119785880631161201471178051

010000003000
87119785880631161201471178051

34124211774
0000000000

0000000000
34124211774

11111

11

211241573

3

030000310000
84114785778591141191271107651

0
1

1

282130182822424046303317
020000001000

231826172519403941273217

2131141

11

1211

1

11

1

1

1

11

11
00

11

00
11

11

204926323127362948542119
030000000000

204626323127362948542019

1

15712284627766
010000000000

15510284527766

121

112
000

112

00000000000
191410323392313135

11

1

19149323392312125

62131131
30000000

1

21

1

1

1

11

111

21

00
11

11

223112123

0000
26111

26111

12
00

12

11
00

00
11

00
11

00
11

11

1111

1220811162438131616
000000000000

000000000000
1220811162438131616

482762318
000000000

482762318
010100000

321

4426638

8126410223513158

0000000000
1321231142

111212
000000

111212
000000

111212
000000

111212

2

000000
211312

000000
211312

111
000

111

0000
2122

2112

1

000000000000
57947944714888112132845852

5174644062468396125764938
060000000000

1172213221
0000000000

0000
1111

1111

171212211
000000000

112111

7211

12
00

12
00

1

2

0
1

1

000000000000
6712675122759826

6712675122759826
000000000000

6712675122759826

12168245121510756

304135304733565149543425
000000000000

111211
000000

211

111

545228883455

0
1

1

11
00

11

11221
00000

11221

253528284425474146462519
000000000000

1

1

253528284425474145462518

22221132454
00000000000

113
000

113

1222132154
0000000010

111

11

12121223

111

6201549251678914
000000000000

6201549251678914

00
31

00
31

00
31

00
31

31

982548165328607768797768
000000000000

801736124421335246716059
000000000000

801736124421335246716059
000000000000

781728104018314543705655

00000000000
28243273144

28243273144

188124972725228179

31
00

00
31

31
00

00
31

31

524333306236243546373771
000000000000

482524235827172939253260
000000000000

482524235827172939253260
020000001000

00
11

11

0
1

1

452222225724172635243152
000000000020

11

1111

442221215624172534242952

111215
000000

111215

0000000
2111332

23

11

1

1

1

1

1

1

418974976712511
000000000000

233344657349
000000000000

233344657348

1
0

1

0000000000
11554311912

92131
00000

2131

9

163311911
020000000

31

1

21811

11

1

1

11

11

0
1

0
1

1

0
1

1
0

1

000000000000
221516111499915111625

221516111499915111625
000000000000

43272112

000000000000
21111311927814101522

21111311927814101522

13111
00000

13111
00000

11

1211

222
000

000
222

000
222

222

207259217137193187509960196271229
000000000000

000000001000
207259217137193187509960196271229

10234351254351520106210457
000000000000

1

002000000010
1012834105230131376010056

1

33321101432

111

5

982428850207137569653

00
31

31

1
0

1

00
11

11

010000000000
151223143131

12112111

13112221

12

0
1

1

000000000000
105225182125139152357949134167172

000000000000
103217176125138147347739125166170

103217176125138147347739125166170

000
111

111

0000000
5414152

5414152

000
213

11

13

0000
2151

11

1141

11
00

11

14

3424310838451
000000000

000000000
3424310838451

3424210738451
020000000

112
000

112

1
0

1

2401043832
000000

2401043832

1121311
0000000

1

1

1211

3

11
00

11
00

11

1

00000
22111

22111
00000

00000
22111

22111
00000

00000
22111

22111

1

1

000000000
2711210814

16111213

000
111

111

11177

1
0

1
0

1

55484445476711138142798142
000000000000

24511

55464045476710638141788142

114
000

114

000
111

111
000

000
111

000
111

000
111

1

11

518211
00000

00000
518211

71
00

00
71

71
00

71

0000
5721

5721
0000

0000
5721

5721

4

1

1

1

1

1

1

1

2

1

000000000000
2178171181122131017

2178171181122131017

000000000000
114385022222437945859957239910

114385022222437945859957239910

1

1

313467431281334317210262296334326248
051000000000

64413669568
00000000000

64413669568
00000000000

00000000000
64413669568

00000000000
64413669568

64413669568

12

112
000

000
112

000
112

12
00

12

1
0

1

2

25632979131066

21

14221

481722089779130314039302365
000000010000

0000
181112

181112
0000

181112
0000

181112

1

000000010000
441472019275127222330241957

29131111295141813131413
000000000000

2912811295141812121413

11
00

11

2

11
00

11

151341908146122841711544

000000000000
465543856435

355421254322

1000
1111

0
1

1

11

12252112
00000000

0000
1221

1221

00000
11512

11512

1111113

201231156110160116104151131208202109
000000010000

1

000000000000
1391411187911683741097911412672

1038171210561101510

3

5

020000001000
1291321076210473691037810411162

0
4

4

1111

00
11

11

431144003000
9572893816115

11

11

11112272

11

111111

11

2244212

11112

11121
00000

111

12

5

000000000000
772117194414285127425418

772117194414285127425418

000
111

111

14111
0000

131

111

22
00

22

1
0

1

000000000000
427178395047374131535338

427178395047374131535338

00000
12111

12111

0
1

1

0
1

1

131
000

131

111111
000000

111111

1

293521142219101417544123
000000000000

010000000000
293521142219101417544123

13510
000

13510

11

000
111

111

12
00

12

0
1

1

00000000
201411221

201411221

200000000000
28132013211571015152621

23132013211371013142518

1

32213

00000
11212

11212

3

000000000010
313416172013192326382614

2531132231
0000000000

2531132121

00
11

11

000000000000
146267277520117

000000000010
144267167417115

14426716741795

1

1
0

1

0
1

1

1111
0000

1111

000
121

121

1523148121012131916116
000000000000

121
000

121

101122736811955
352141243820

76132438135

1

1111
0000

1111

13111
00000

1311

1

0000
1222

11

1121

000000
223131

2212

2111

1111
0000

11

11

1
0

1

0000010
6822321

6822311

1
0

1

311211
000000

211111

11

21121
00000

111

11

11

22012114629
0000000000

22012114629
0400000000

00
15

15

1111
0000

1111

13111
00000

13111

46

52322
50001

131

12

21
00

21

11

5345567091555856102778759

111

2

1217
0000

1217

3361

2

1

1

5

931222774413
000000000000

931222774413

11

1

1

1

1

2
